# A Comprehensive DNA Barcode Library for the Looper Moths (Lepidoptera: Geometridae) of British Columbia, Canada

**DOI:** 10.1371/journal.pone.0018290

**Published:** 2011-03-28

**Authors:** Jeremy R. deWaard, Paul D. N. Hebert, Leland M. Humble

**Affiliations:** 1 Department of Forest Sciences, University of British Columbia, Vancouver, British Columbia, Canada; 2 Entomology, Royal British Columbia Museum, Victoria, British Columbia, Canada; 3 Biodiversity Institute of Ontario, University of Guelph, Guelph, Ontario, Canada; 4 Canadian Forest Service, Natural Resources Canada, Victoria, British Columbia, Canada; American Museum of Natural History, United States of America

## Abstract

**Background:**

The construction of comprehensive reference libraries is essential to foster the development of DNA barcoding as a tool for monitoring biodiversity and detecting invasive species. The looper moths of British Columbia (BC), Canada present a challenging case for species discrimination via DNA barcoding due to their considerable diversity and limited taxonomic maturity.

**Methodology/Principal Findings:**

By analyzing specimens held in national and regional natural history collections, we assemble barcode records from representatives of 400 species from BC and surrounding provinces, territories and states. Sequence variation in the barcode region unambiguously discriminates over 93% of these 400 geometrid species. However, a final estimate of resolution success awaits detailed taxonomic analysis of 48 species where patterns of barcode variation suggest cases of cryptic species, unrecognized synonymy as well as young species.

**Conclusions/Significance:**

A catalog of these taxa meriting further taxonomic investigation is presented as well as the supplemental information needed to facilitate these investigations.

## Introduction

For monitoring biodiversity and detecting invasive species, knowing what species exist in a given location is paramount. However, the subtle morphological characters that separate closely related species often demand expert interpretation (e.g. [1]), forcing studies to either limit their taxonomic scope, or to only identify specimens to a higher taxonomic category (e.g. family, genus). DNA barcoding can circumvent these limits by transforming the often lengthy chore of identifying specimens to a rapid, accurate and unbiased task [2]–[4]. For the identification of arthropods in particular, where high diversity and low access to taxonomic expertise complicate the job, DNA barcoding has proven capable of the task, in numerous groups including collembolans [5], spiders [6], tephritid fruit flies [2], mosquitoes [7], tachinid flies [8], aphids [9], ants [10], wood wasps [11], black flies [12], and mayflies, stoneflies and caddisflies [13]. Lepidoptera has seen the most studies of barcode performance to date, and results suggest barcodes permit correct identification in >90% of previously recognized taxa [14]–[17].

To continue the development of DNA barcoding as a tool for biodiversity monitoring and invasive species detection, it is necessary to both construct complete reference libraries and assess their efficacy for discriminating species. The taxa for which barcoding delivers results that are discordant with current taxonomy are of particular interest — they generally warrant further investigation as they may represent overlooked species [18], [19], species that are hybridizing, cases of synonymy or situations that require a secondary barcode marker for species diagnosis. It is also worthwhile to explore the effect of sampling on estimates of genetic variation, both in terms of number [20], [21] and geographic coverage [16], [17].

The loopers or inchworm moths (Lepidoptera: Geometridae) are one of the largest insect families, composed of nearly 23,000 species worldwide [22] and roughly 1400 in North America [23]. They are an abundant, diverse component of most forest ecosystems — this, along with their weak flight ability and low propensity to migrate [24], [25], make them excellent indicators of environmental quality [26]. A large proportion of the species are also important defoliators, including native species such as the fall cankerworm (*Alsophila pometaria* (Harris)) and invasive pests such as the winter moth (*Operophtera brumata* (L.)). Because most larvae and adults possess cryptic coloration, they are a notoriously tough group in which to discriminate species. To further complicate matters, most North American geometrid genera are in need of revision. This latter gap is now being addressed through an ‘integrative approach’ [27] that employs DNA barcodes to accelerate the revisionary process in both North America (e.g., [28], [29]) and elsewhere (e.g., [30], [31]).

The geometrids of British Columbia (BC), Canada present a challenging case for DNA barcoding. There are presently 349 species known from the province — a large fauna with varying levels of taxonomic maturity (JRD unpublished). In this study, we assemble representatives of nearly all these species, from BC and the surrounding region, and from geographically separated individuals, to examine patterns of barcode divergence. We test the hypothesis that the barcode region is able to reliably discriminate geometrid species as demonstrated in other taxa (see [32]–[35]) and determine which species merit further investigation of their taxonomic status. The result is a reliable identification library with immediate application for monitoring looper moth biodiversity and detecting invasive species in BC.

## Materials and Methods

### Sampling

We chose the province of British Columbia as the primary scope of our library; its boundary does not correspond with the limits of particular biomes. However, this regional focus was chosen to maximize the development of a barcode library that would have high value for biodiversity monitoring and invasive species detection in the province. Although BC is primarily in the Western Cordillera biome, it includes some plains, maritime and subarctic ecosystems [36] so the fauna has considerable overlap with surrounding provinces, territories and states. Since the ranges of many geometrid moths are poorly known, and many will shift with climate change, we also sampled selected taxa from adjacent regions, including Alaska, Yukon Territory, Alberta, Washington State, and Idaho, but did not attempt to sample their entire faunas. We also included *Callizzia amorata* Packard (Epipleminae), the sole BC representative of the Uraniidae, sister group to the Geometridae (e.g., [37]) within the Geometroidea.

We selected specimens from eight regional and national insect collections: Canadian National Collection of Insects and Arachnids (Ottawa, ON), Royal BC Museum (Victoria, BC), Canadian Forest Service (Victoria, BC), University of British Columbia's Spencer Collection (Vancouver, BC), Washington State University's James Entomological Collection (Pullman, WA), University of Idaho's WFBARR Collection (Moscow, ID), Northern Forestry Centre (Edmonton, AB) and University of Alberta's Strickland Collection (Edmonton, AB). An effort was made to sample at least five geographically distinct specimens for each species, to best appraise the genetic variation across its range. Specimens less than 30 years old were chosen when possible to avoid problems associated with DNA degradation. Some of the specimens may have been misidentified due to the difficulty of the group and lack of an expert curator in most of the collections. Where availability of taxonomic literature and time permitted, species identifications were corrected prior to or following DNA analysis by examining genitalia and external morphology of the vouchered specimens. In addition, a few specimens were freshly collected on targeted collecting trips, or by making requests to entomologists in the region. All specimens were labeled, databased and imaged and made publicly available on the Barcode of Life Data Systems (BOLD) [38] in the project ‘GOBCL – Geometridae of BC Library’. The institution storing each vouchered specimen is listed in the BOLD project and Table S1.

### DNA analysis

One or two legs were removed from each dried specimen and stored in an individual tube of a 96-tube sample box (Matrix Technologies) or an individual well of a microplate. DNA extraction, amplification, and sequencing of the barcode region of the mitochondrial cytochrome *c* oxidase I (COI) gene followed a variety of high-throughput techniques recently developed at the Canadian Centre for DNA Barcoding ([39]–[41]; www.barcodinglife.ca). The full-length primers LepF1 and LepR1 [42] were attempted first, but amplification and sequencing using the ‘Lep mini primers’ (MLepF1, MLepR1) [14] was necessary for most of the older material. The electropherograms were edited and aligned in Seqscape v. 2.5 (Applied Biosystems), then deposited along with the edited sequences to BOLD and GenBank (accessions are listed in Table S1). In the 61 instances where we were unable to successfully sequence a desired species from BC, sequences were obtained from specimens collected in other regions.

### Data analysis

To investigate the efficacy of barcodes to differentiate geometrid species, sequence divergence within and between species was calculated using the Kimura 2-parameter model [43] and the neighbour-joining algorithm [44], as implemented in BOLD and MEGA4 [45]. We first tallied the proportion of species that could successfully be distinguished by DNA barcoding to calculate an overall success rate. The successful differentiation of a species required that its barcodes formed monophyletic clusters and were not shared with other species. We also determined which species displayed sequence diversity >3%, an arbitrary threshold that generally falls within the so-called ‘barcode gap’ (i.e. the lack of overlap between intra- and inter-specific divergence, *sensu* [46]). And lastly, to ascertain the potential of sampling bias, we tested the significance of the relationship between mean intra-specific divergence and the number of individuals analyzed by performing a linear regression in SPSS v17 (IBM).

## Results and Discussion

A total of 2392 COI sequences were generated in this study, providing coverage for 400 species and 125 genera. Most sequences were derived from specimens from BC (N = 1390) or surrounding provinces, territories and states (N = 966). The remainder was collected in other North American regions (N = 35) and from a single German specimen (of the biological control agent *Minoa murinata* (Scopoli)). Of the 349 species listed for BC (JRD unpublished), only *Hydrelia brunneifasciata* (Packard) was not successfully barcoded. Most species were represented by multiple samples (mean  =  6.0 individuals/species; maximum  =  46), but 62 species had only a single COI barcode. All but nine sequences were greater than 500 bp (mean  =  648 bp, range  =  238 to 658 bp) and therefore meet the ‘BARCODE data standard’ (see [20]). The assembly of this comprehensive dataset reveals the important role that natural history collections possess for barcode library construction, both in terms of access to entire regional faunas and to specimens conducive to DNA analysis.

The neighbour-joining analysis resulted in a tree with most species forming distinct, cohesive units displaying minimal sequence variation (Figure S1). We found 27 species (6.8%) with undifferentiated or overlapping barcodes (Table 1), whereas the remaining 373 (93.2%) formed non-overlapping monophyletic clusters. Taxa that have undergone recent taxonomic revision appeared to have a higher proportion of species with diagnostic barcodes e.g. *Eupithecia* spp. (revised in [47]) – 55 of 55 species formed non-overlapping monophyletic clusters; species of Macariini [48] – 53/54; and *Tetracis* spp. [28] – 6/6. Conversely, taxa known to be in need of revision were often comprised of several species that could not be differentiated by barcodes, such as *Lobophora* (noted in [29]) where 3 of 5 species lacked diagnostic barcodes. There was also a single case, the species pair of *Probole alienaria* and *amicaria* Herrich-Schäffer, [1855], where the COI data were unable to differentiate the two taxa, corroborating unpublished revisionary work by Tomon [49] who considers it a single, highly variable species. The rate of species-level identification in the present dataset is slightly lower than in most previous barcoding studies on Lepidoptera [14]–[17], but it is likely to increase with re-examination of potentially misidentified specimens and further taxonomic investigation of this fauna.

**Table 1:** Geometrid species not distinguishable by DNA barcodes.

Taxon	Condition	Congener involved
*Caripeta divisata* Walker	paraphyletic	*angustiorata* Walker
*Eufidonia discospilata* (Walker)	paraphyletic	*convergaria* (Walker)
*Hydriomena edenata ^*Swett	paraphyletic	*crokeri* Swett
*Macaria signaria* (Hübner)	paraphyletic	*oweni* (Swett)
*Hydriomena furcata* (Thunberg)	paraphyletic	*quinquefasciata* (Packard)
*Dysstroma hersiliata* (Guenée)	paraphyletic	*rutlandia* McDunnough
*Eustroma semiatrata* (Hulst)	paraphyletic	*fasciata* Barnes & McDunnough
*Epirrita autumnata* (Borkhausen),	paraphyletic	*undulata* (Harrison)
*Lobophora magnoliatoidata* (Dyar)	polyphyletic	*nivigerata* Walker, *simsata* Swett
*Dysstroma colvillei* Blackmore	polyphyletic	*formosa (Hulst), hersiliata* (Guenée), *rutlandia* (McDunnough)
*Xanthorhoe ramaria* Swett & Cassino	polyphyletic	*lagganata* Swett & Cassino, *baffinensis* McDunnough
*Lobophora simsata* Swett	identical barcodes	*nivigerata* Walker
*Lobophora nivigerata* Walker	identical barcodes	*simsata* Swett
*Cabera exanthemata* (Scopoli)	identical barcodes	*erythemaria* Guenée
*Cabera erythemaria* Guenée	identical barcodes	*exanthemata* (Scopoli)
*Drepanulatrix falcataria* (Packard)	identical barcodes	*carnearia* (Hulst)
*Drepanulatrix carnearia* (Hulst)	identical barcodes	*falcataria* (Packard)
*Xanthotype urticaria* Swett	identical barcodes	*sospeta* (Drury)
*Xanthotype sospeta* (Drury)	identical barcodes	*urticaria* Swett
*Orthofidonia exornata* (Walker)	identical barcodes	*tinctaria* (Walker)
*Orthofidonia tinctaria* (Walker)	identical barcodes	*exornata* (Walker)
*Probole amicaria* (Herrich-Schäffer)	overlapping barcodes	*alienaria* Herrich-Schäffer
*Probole alienaria* Herrich-Schäffer	overlapping barcodes	*amicaria* (Herrich-Schäffer)
*Chlorosea banksaria* Sperry	overlapping barcodes	*nevadaria* Packard
*Chlorosea nevadaria* Packard	overlapping barcodes	*banksaria* Sperry
*Rheumaptera subhastata* (Nolcken)	identical and overlapping barcodes	*hastata* (Linnaeus)
*Rheumaptera hastata* (Linnaeus)	identical and overlapping barcodes	*subhastata* (Nolcken)

The 27 taxa in the left column cannot be diagnosed by COI based on one of five conditions: paraphyletic with respect to one congener; polyphyletic with two or three congeners; share an identical COI haplotype with a congener; haplotypes of one taxon do not form distinct clusters and overlap with haplotypes of congeners; or a combination of the latter two conditions.

**Figure 1:**
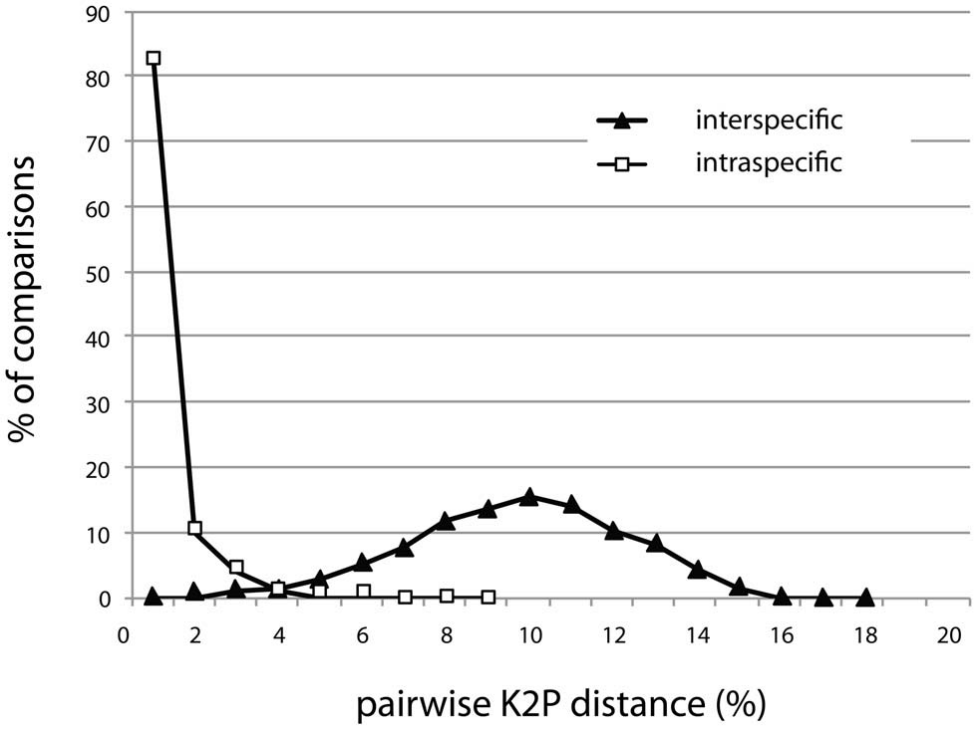
Combined histograms of pairwise Kimura 2-Parameter (K2P) sequence variation. Solid triangles indicate interspecific divergences between 116 congeneric taxa (70,580 comparisons) while the open squares indicate intraspecific divergences in the 339 species with multiple samples (11,949 comparisons).

**Figure 2:**
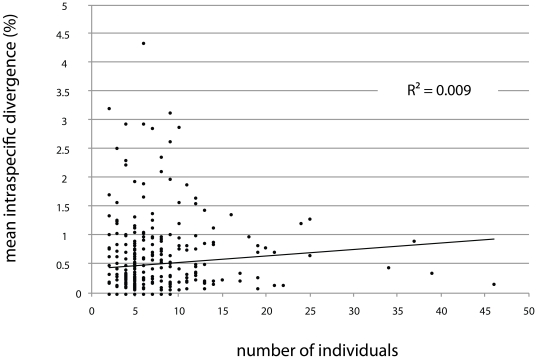
The relationship between mean intra-specific divergence and the number of individuals analyzed. The linear regression is not significant (P = 0.07).

As the mean interspecific divergence between congeneric taxa (9.17%; range  =  0 to 17.27%) was 16-fold higher than mean intraspecific variation (0.56%; range  =  0 to 8.73%), the distributions of intra- and interspecific divergences showed limited overlap (Figure 1). There was no association between mean intra-specific distance and sample size (Figure 2, linear regression, R^2^ = 0.09, P = 0.07) suggesting our sampling strategy was representative for all taxa. There were 26 instances of high intra-specific divergence (>3%) among the 338 species with multiple samples (Table 2). Of these, 22 cases involved two distinct clusters and 4 involved three clusters. These discrete clusters may indicate the presence of cryptic species, as barcoding has proven invaluable for flagging species that have gone previously unrecognized (e.g. [19], [42], [50]–[52]). Conversely, one or more instances may be attributable to misidentifications. Five of these 26 taxa demonstrating high intraspecific variation are also listed in Table 1 as taxa indistinguishable by barcodes, so the total number of BC geometrid species that require re-examination of specimens and further taxonomic scrutiny is 48.

**Table 2:** Geometrid species with high intraspecific COI variation.

Taxon	Individuals per lineage	Mean sequence divergence (%)
*Aethalura intertexta* (Walker)	1/9	5.31
*Dichorda rectaria* (Grote)	1/2	3.37
*Digrammia irrorata* (Packard)	4/3	4.87
*Dysstroma colvillei* (Guenée)	2/2	3.37
*Ectropis crepuscularia* (Denis & Schiffermüller)	1/20	4.61
*Eupithecia annulata* (Hulst)	2/12	3.13
*Eupithecia lachrymosa* (Hulst)	3/9	3.71
*Eupithecia longipalpata* Packard	1/2	4.08
*Eustroma atrifasciata* (Hulst)	1/1	3.20
*Eustroma semiatrata* (Hulst)	2/4/3	4.28
*Hydriomena perfracta* (Swett)	1/3	4.43
*Macaria colata* (Grote)	1/7/1	5.04
*Macaria decorata* (Hulst)	2/8	7.56
*Mesoleuca gratulata* (Walker)	8/1	4.39
*Nemoria unitaria* (Packard)	1/1/7	4.45
*Plataea trilinearia* (Packard)	4/4	3.59
*Plemyria georgii* Hulst	1/5	7.31
*Probole alienaria* Herrich-Schäffer	1/5	3.63
*Rheumaptera hastata* (Linnaeus)	1/2/3	5.73
*Rheumaptera subhastata* (Nolcken)	1/7	5.05
*Sicya macularia* (Harris)	5/6	3.25
*Spodolepis danbyi* (Hulst)	1/11	3.94
*Synchlora aerata* (Fabricius)	2/8	3.64
*Synchlora bistriaria* (Packard)	1/36	5.75
*Triphosa haesitata* (Guenée)	1/9	3.64
*Xanthorhoe lacustrata* (Guenée)	1/6	4.58

The number of specimens per cluster is separated by a forward slash (/) with two numbers indicating cases with two distinct clusters and three numbers indicating three clusters. The mean sequence diverence calculated for each species was caluculated using the Kimura 2-parameter distance model.

In summary, two tangible products have arisen from the current study. First, a comprehensive reference library was constructed for the Geometridae of British Columbia that can be employed immediately for biodiversity monitoring and invasive species detection. This library provides species-level resolution in over 93% of cases, and resolution to a congeneric species pair or group in the remaining cases. This small proportion of recognized taxa that apparently do not possess diagnostic barcodes, as well the fraction of species potentially housing cryptic species, constitutes the second product — a catalog of taxa that require taxonomic investigation. Moreover, this catalog includes the materials necessary to facilitate the investigations — a database of specimens vouchered in permanent collections, each linked to publicly available genetic and collateral data. Used in combination, these components can accelerate integrative taxonomic studies [51], [53] and define the ‘taxonomy of the future’ [54].

## Supporting Information

Figure S1 Neighbour-joining tree for 400 species of Geometridae and Uraniidae from British Columbia, Canada and surrounding provinces, territories and states. BOLD process IDs and collection localities are provided for each sequence.
(PDF)

Table S1 List of specimens analyzed in the present study. Specimen accessions, BOLD process IDs, GenBank accessions, collection localities, and the storing institution are provided for each specimen.
(PDF)
