# Supplementary material for: A Comprehensive DNA Barcode Library for the Looper Moths (Lepidoptera: Geometridae) of British Columbia, Canada
Source: PLoS One. 2011 Mar 28;6(3):e18290. doi: 10.1371/journal.pone.0018290 (PMC3065486; doi:10.1371/journal.pone.0018290)
Supplement: Figure S1 — Neighbour-joining tree for 400 species of Geometridae and Uraniidae from British Columbia, Canada and surrounding provinces, territories and states. BOLD process IDs and collection localities are provided for each sequence. (PDF) [file pone.0018290.s001.pdf]

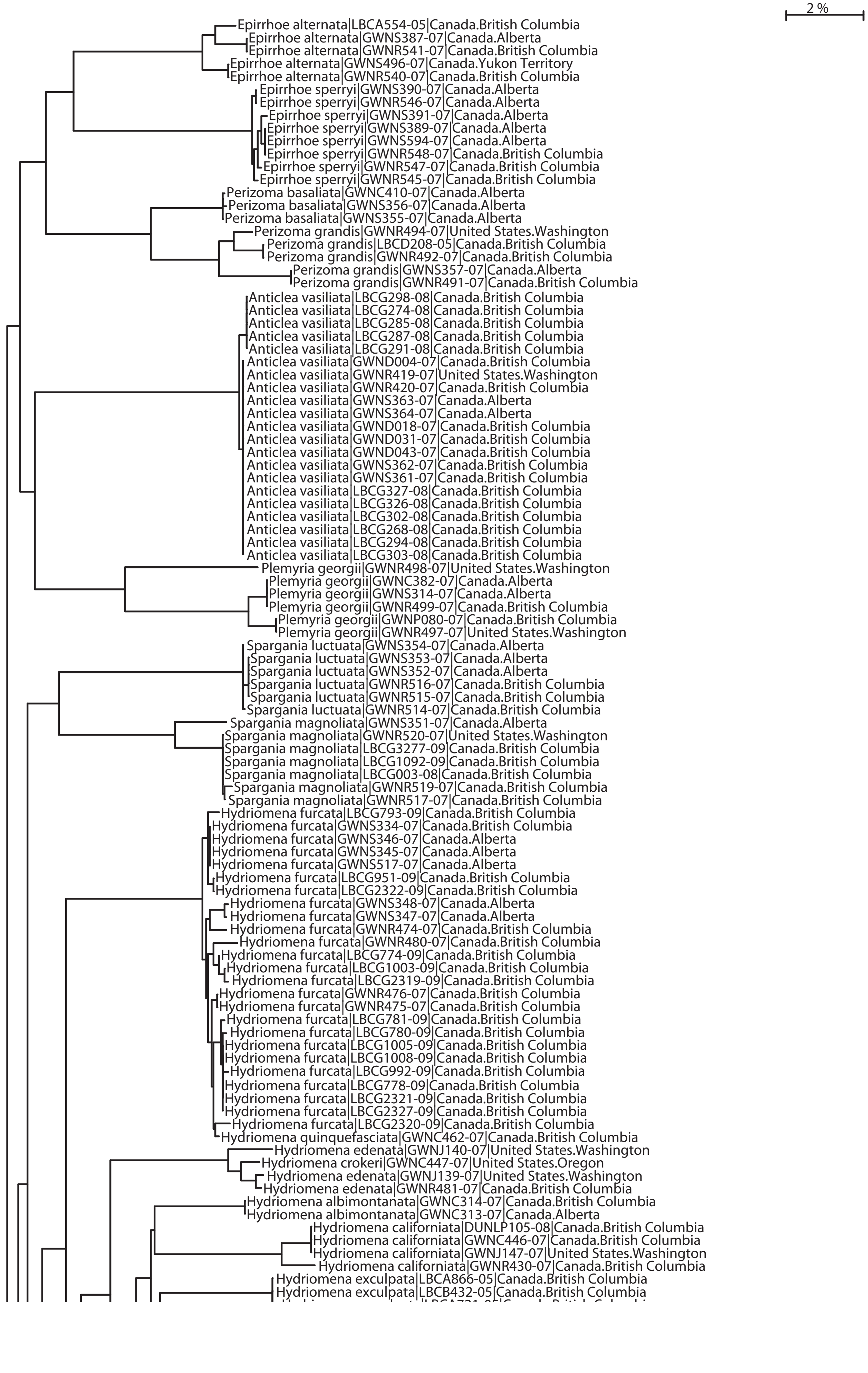

2 %
Epirrhoe alternata|LBCA554-05|Canada.British Columbia
Epirrhoe alternata|GWNS387-07|Canada.Alberta
Epirrhoe alternata|GWNR541-07|Canada.British Columbia
Epirrhoe alternata|GWNS496-07|Canada.Yukon Territory
Epirrhoe alternata|GWNR540-07|Canada.British Columbia
Epirrhoe sperryi|GWNS390-07|Canada.Alberta
Epirrhoe sperryi|GWNR546-07|Canada.Alberta
Epirrhoe sperryi|GWNS391-07|Canada.Alberta
Epirrhoe sperryi|GWNS389-07|Canada.Alberta
Epirrhoe sperryi|GWNS594-07|Canada.Alberta
Epirrhoe sperryi|GWNR548-07|Canada.British Columbia
Epirrhoe sperryi|GWNR547-07|Canada.British Columbia
Epirrhoe sperryi|GWNR545-07|Canada.British Columbia
Perizoma basaliata|GWNC410-07|Canada.Alberta
Perizoma basaliata|GWNS356-07|Canada.Alberta
Perizoma basaliata|GWNS355-07|Canada.Alberta
Perizoma grandis|GWNR494-07|United States.Washington
Perizoma grandis|LBCD208-05|Canada.British Columbia
Perizoma grandis|GWNR492-07|Canada.British Columbia
Perizoma grandis|GWNS357-07|Canada.Alberta
Perizoma grandis|GWNR491-07|Canada.British Columbia
Anticlea vasiliata|LBCG298-08|Canada.British Columbia
Anticlea vasiliata|LBCG274-08|Canada.British Columbia
Anticlea vasiliata|LBCG285-08|Canada.British Columbia
Anticlea vasiliata|LBCG287-08|Canada.British Columbia
Anticlea vasiliata|LBCG291-08|Canada.British Columbia
Anticlea vasiliata|GWND004-07|Canada.British Columbia
Anticlea vasiliata|GWNR419-07|United States.Washington
Anticlea vasiliata|GWNR420-07|Canada.British Columbia
Anticlea vasiliata|GWNS363-07|Canada.Alberta
Anticlea vasiliata|GWNS364-07|Canada.Alberta
Anticlea vasiliata|GWND018-07|Canada.British Columbia
Anticlea vasiliata|GWND031-07|Canada.British Columbia
Anticlea vasiliata|GWND043-07|Canada.British Columbia
Anticlea vasiliata|GWNS362-07|Canada.British Columbia
Anticlea vasiliata|GWNS361-07|Canada.British Columbia
Anticlea vasiliata|LBCG327-08|Canada.British Columbia
Anticlea vasiliata|LBCG326-08|Canada.British Columbia
Anticlea vasiliata|LBCG302-08|Canada.British Columbia
Anticlea vasiliata|LBCG268-08|Canada.British Columbia
Anticlea vasiliata|LBCG294-08|Canada.British Columbia
Anticlea vasiliata|LBCG303-08|Canada.British Columbia
Plemyria georgii|GWNR498-07|United States.Washington
Plemyria georgii|GWNC382-07|Canada.Alberta
Plemyria georgii|GWNS314-07|Canada.Alberta
Plemyria georgii|GWNR499-07|Canada.British Columbia
Plemyria georgii|GWNP080-07|Canada.British Columbia
Plemyria georgii|GWNR497-07|United States.Washington
Spargania luctuata|GWNS354-07|Canada.Alberta
Spargania luctuata|GWNS353-07|Canada.Alberta
Spargania luctuata|GWNS352-07|Canada.Alberta
Spargania luctuata|GWNR516-07|Canada.British Columbia
Spargania luctuata|GWNR515-07|Canada.British Columbia
Spargania luctuata|GWNR514-07|Canada.British Columbia
Spargania magnoliata|GWNS351-07|Canada.Alberta
Spargania magnoliata|GWNR520-07|United States.Washington
Spargania magnoliata|LBCG3277-09|Canada.British Columbia
Spargania magnoliata|LBCG1092-09|Canada.British Columbia
Spargania magnoliata|LBCG003-08|Canada.British Columbia
Spargania magnoliata|GWNR519-07|Canada.British Columbia
Spargania magnoliata|GWNR517-07|Canada.British Columbia
Hydriomena furcata|LBCG793-09|Canada.British Columbia
Hydriomena furcata|GWNS334-07|Canada.British Columbia
Hydriomena furcata|GWNS346-07|Canada.Alberta
Hydriomena furcata|GWNS345-07|Canada.Alberta
Hydriomena furcata|GWNS517-07|Canada.Alberta
Hydriomena furcata|LBCG951-09|Canada.British Columbia
Hydriomena furcata|LBCG2322-09|Canada.British Columbia
Hydriomena furcata|GWNS348-07|Canada.Alberta
Hydriomena furcata|GWNS347-07|Canada.Alberta
Hydriomena furcata|GWNR474-07|Canada.British Columbia
Hydriomena furcata|GWNR480-07|Canada.British Columbia
Hydriomena furcata|LBCG774-09|Canada.British Columbia
Hydriomena furcata|LBCG1003-09|Canada.British Columbia
Hydriomena furcata|LBCG2319-09|Canada.British Columbia
Hydriomena furcata|GWNR476-07|Canada.British Columbia
Hydriomena furcata|GWNR475-07|Canada.British Columbia
Hydriomena furcata|LBCG781-09|Canada.British Columbia
Hydriomena furcata|LBCG780-09|Canada.British Columbia
Hydriomena furcata|LBCG1005-09|Canada.British Columbia
Hydriomena furcata|LBCG1008-09|Canada.British Columbia
Hydriomena furcata|LBCG992-09|Canada.British Columbia
Hydriomena furcata|LBCG778-09|Canada.British Columbia
Hydriomena furcata|LBCG2321-09|Canada.British Columbia
Hydriomena furcata|LBCG2327-09|Canada.British Columbia
Hydriomena furcata|LBCG2320-09|Canada.British Columbia
Hydriomena quinquefasciata|GWNC462-07|Canada.British Columbia
Hydriomena edenata|GWNJ140-07|United States.Washington
Hydriomena crokeri|GWNC447-07|United States.Oregon
Hydriomena edenata|GWNJ139-07|United States.Washington
Hydriomena edenata|GWNR481-07|Canada.British Columbia
Hydriomena albimontanata|GWNC314-07|Canada.British Columbia
Hydriomena albimontanata|GWNC313-07|Canada.Alberta
Hydriomena californiata|DUNLP105-08|Canada.British Columbia
Hydriomena californiata|GWNC446-07|Canada.British Columbia
Hydriomena californiata|GWNJ147-07|United States.Washington
Hydriomena californiata|GWNR430-07|Canada.British Columbia
Hydriomena exculpata|LBCA866-05|Canada.British Columbia
Hydriomena exculpata|LBCB432-05|Canada.British Columbia

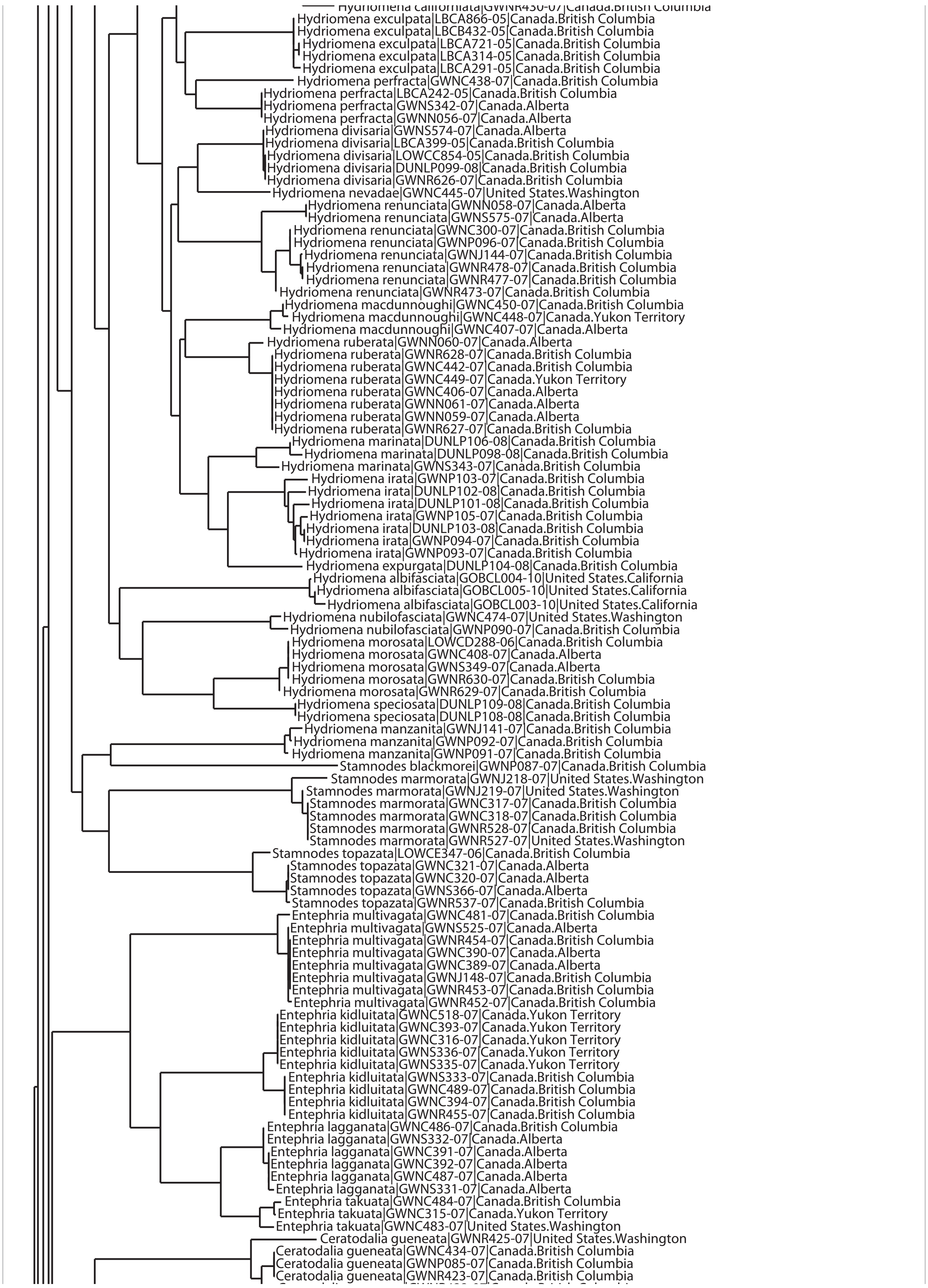
Hydriomena californiata|GWNR430-07|Canada.British Columbia
Hydriomena exculpata|LBCA866-05|Canada.British Columbia
Hydriomena exculpata|LBCB432-05|Canada.British Columbia
Hydriomena exculpata|LBCA721-05|Canada.British Columbia
Hydriomena exculpata|LBCA314-05|Canada.British Columbia
Hydriomena exculpata|LBCA291-05|Canada.British Columbia
Hydriomena perfracta|GWNC438-07|Canada.British Columbia
Hydriomena perfracta|LBCA242-05|Canada.British Columbia
Hydriomena perfracta|GWNS342-07|Canada.Alberta
Hydriomena perfracta|GWNN056-07|Canada.Alberta
Hydriomena divisaria|GWNS574-07|Canada.Alberta
Hydriomena divisaria|LBCA399-05|Canada.British Columbia
Hydriomena divisaria|LOWCC854-05|Canada.British Columbia
Hydriomena divisaria|DUNLP099-08|Canada.British Columbia
Hydriomena divisaria|GWNR626-07|Canada.British Columbia
Hydriomena nevadae|GWNC445-07|United States.Washington
Hydriomena renunciata|GWNN058-07|Canada.Alberta
Hydriomena renunciata|GWNS575-07|Canada.Alberta
Hydriomena renunciata|GWNC300-07|Canada.British Columbia
Hydriomena renunciata|GWNP096-07|Canada.British Columbia
Hydriomena renunciata|GWNJ144-07|Canada.British Columbia
Hydriomena renunciata|GWNR478-07|Canada.British Columbia
Hydriomena renunciata|GWNR477-07|Canada.British Columbia
Hydriomena renunciata|GWNR473-07|Canada.British Columbia
Hydriomena macdunnoughi|GWNC450-07|Canada.British Columbia
Hydriomena macdunnoughi|GWNC448-07|Canada.Yukon Territory
Hydriomena macdunnoughi|GWNC407-07|Canada.Alberta
Hydriomena ruberata|GWNN060-07|Canada.Alberta
Hydriomena ruberata|GWNR628-07|Canada.British Columbia
Hydriomena ruberata|GWNC442-07|Canada.British Columbia
Hydriomena ruberata|GWNC449-07|Canada.Yukon Territory
Hydriomena ruberata|GWNC406-07|Canada.Alberta
Hydriomena ruberata|GWNN061-07|Canada.Alberta
Hydriomena ruberata|GWNN059-07|Canada.Alberta
Hydriomena ruberata|GWNR627-07|Canada.British Columbia
Hydriomena marinata|DUNLP106-08|Canada.British Columbia
Hydriomena marinata|DUNLP098-08|Canada.British Columbia
Hydriomena marinata|GWNS343-07|Canada.British Columbia
Hydriomena irata|GWNP103-07|Canada.British Columbia
Hydriomena irata|DUNLP102-08|Canada.British Columbia
Hydriomena irata|DUNLP101-08|Canada.British Columbia
Hydriomena irata|GWNP105-07|Canada.British Columbia
Hydriomena irata|DUNLP103-08|Canada.British Columbia
Hydriomena irata|GWNP094-07|Canada.British Columbia
Hydriomena irata|GWNP093-07|Canada.British Columbia
Hydriomena expurgata|DUNLP104-08|Canada.British Columbia
Hydriomena albifasciata|GOBCL004-10|United States.California
Hydriomena albifasciata|GOBCL005-10|United States.California
Hydriomena albifasciata|GOBCL003-10|United States.California
Hydriomena nubilofasciata|GWNC474-07|United States.Washington
Hydriomena nubilofasciata|GWNP090-07|Canada.British Columbia
Hydriomena morosata|LOWCD288-06|Canada.British Columbia
Hydriomena morosata|GWNC408-07|Canada.Alberta
Hydriomena morosata|GWNS349-07|Canada.Alberta
Hydriomena morosata|GWNR630-07|Canada.British Columbia
Hydriomena morosata|GWNR629-07|Canada.British Columbia
Hydriomena speciosata|DUNLP109-08|Canada.British Columbia
Hydriomena speciosata|DUNLP108-08|Canada.British Columbia
Hydriomena manzanita|GWNJ141-07|Canada.British Columbia
Hydriomena manzanita|GWNP092-07|Canada.British Columbia
Hydriomena manzanita|GWNP091-07|Canada.British Columbia
Stamnodes blackmorei|GWNP087-07|Canada.British Columbia
Stamnodes marmorata|GWNJ218-07|United States.Washington
Stamnodes marmorata|GWNJ219-07|United States.Washington
Stamnodes marmorata|GWNC317-07|Canada.British Columbia
Stamnodes marmorata|GWNC318-07|Canada.British Columbia
Stamnodes marmorata|GWNR528-07|Canada.British Columbia
Stamnodes marmorata|GWNR527-07|United States.Washington
Stamnodes topazata|LOWCE347-06|Canada.British Columbia
Stamnodes topazata|GWNC321-07|Canada.Alberta
Stamnodes topazata|GWNC320-07|Canada.Alberta
Stamnodes topazata|GWNS366-07|Canada.Alberta
Stamnodes topazata|GWNR537-07|Canada.British Columbia
Entephria multivagata|GWNC481-07|Canada.British Columbia
Entephria multivagata|GWNS525-07|Canada.Alberta
Entephria multivagata|GWNR454-07|Canada.British Columbia
Entephria multivagata|GWNC390-07|Canada.Alberta
Entephria multivagata|GWNC389-07|Canada.Alberta
Entephria multivagata|GWNJ148-07|Canada.British Columbia
Entephria multivagata|GWNR453-07|Canada.British Columbia
Entephria multivagata|GWNR452-07|Canada.British Columbia
Entephria kidluitata|GWNC518-07|Canada.Yukon Territory
Entephria kidluitata|GWNC393-07|Canada.Yukon Territory
Entephria kidluitata|GWNC316-07|Canada.Yukon Territory
Entephria kidluitata|GWNS336-07|Canada.Yukon Territory
Entephria kidluitata|GWNS335-07|Canada.Yukon Territory
Entephria kidluitata|GWNS333-07|Canada.British Columbia
Entephria kidluitata|GWNC489-07|Canada.British Columbia
Entephria kidluitata|GWNC394-07|Canada.British Columbia
Entephria kidluitata|GWNR455-07|Canada.British Columbia
Entephria lagganata|GWNC486-07|Canada.British Columbia
Entephria lagganata|GWNS332-07|Canada.Alberta
Entephria lagganata|GWNC391-07|Canada.Alberta
Entephria lagganata|GWNC392-07|Canada.Alberta
Entephria lagganata|GWNC487-07|Canada.Alberta
Entephria lagganata|GWNS331-07|Canada.Alberta
Entephria takuata|GWNC484-07|Canada.British Columbia
Entephria takuata|GWNC315-07|Canada.Yukon Territory
Entephria takuata|GWNC483-07|United States.Washington
Ceratodalia gueneata|GWNR425-07|United States.Washington
Ceratodalia gueneata|GWNC434-07|Canada.British Columbia
Ceratodalia gueneata|GWNP085-07|Canada.British Columbia
Ceratodalia gueneata|GWNR423-07|Canada.British Columbia

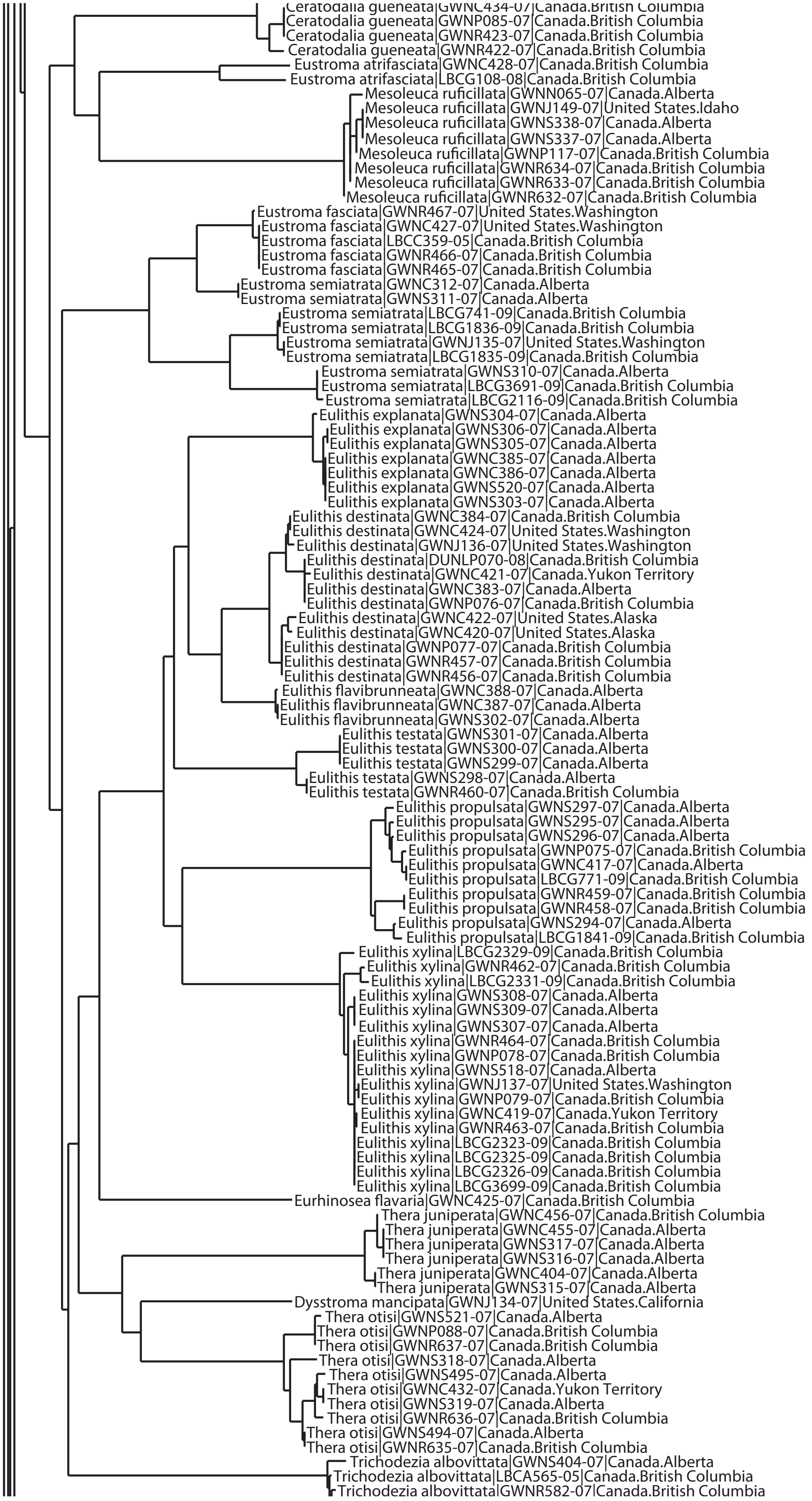

Ceratodalia gueneata|GWNC434-07|Canada.British Columbia
Ceratodalia gueneata|GWNP085-07|Canada.British Columbia
Ceratodalia gueneata|GWNR423-07|Canada.British Columbia
Ceratodalia gueneata|GWNR422-07|Canada.British Columbia
Eustroma atrifasciata|GWNC428-07|Canada.British Columbia
Eustroma atrifasciata|LBCG108-08|Canada.British Columbia
Mesoleuca ruficillata|GWNN065-07|Canada.Alberta
Mesoleuca ruficillata|GWNJ149-07|United States.Idaho
Mesoleuca ruficillata|GWNS338-07|Canada.Alberta
Mesoleuca ruficillata|GWNS337-07|Canada.Alberta
Mesoleuca ruficillata|GWNP117-07|Canada.British Columbia
Mesoleuca ruficillata|GWNR634-07|Canada.British Columbia
Mesoleuca ruficillata|GWNR633-07|Canada.British Columbia
Mesoleuca ruficillata|GWNR632-07|Canada.British Columbia
Eustroma fasciata|GWNR467-07|United States.Washington
Eustroma fasciata|GWNC427-07|United States.Washington
Eustroma fasciata|LBCC359-05|Canada.British Columbia
Eustroma fasciata|GWNR466-07|Canada.British Columbia
Eustroma fasciata|GWNR465-07|Canada.British Columbia
Eustroma semiatrata|GWNC312-07|Canada.Alberta
Eustroma semiatrata|GWNS311-07|Canada.Alberta
Eustroma semiatrata|LBCG741-09|Canada.British Columbia
Eustroma semiatrata|LBCG1836-09|Canada.British Columbia
Eustroma semiatrata|GWNJ135-07|United States.Washington
Eustroma semiatrata|LBCG1835-09|Canada.British Columbia
Eustroma semiatrata|GWNS310-07|Canada.Alberta
Eustroma semiatrata|LBCG3691-09|Canada.British Columbia
Eustroma semiatrata|LBCG2116-09|Canada.British Columbia
Eulithis explanata|GWNS304-07|Canada.Alberta
Eulithis explanata|GWNS306-07|Canada.Alberta
Eulithis explanata|GWNS305-07|Canada.Alberta
Eulithis explanata|GWNC385-07|Canada.Alberta
Eulithis explanata|GWNC386-07|Canada.Alberta
Eulithis explanata|GWNS520-07|Canada.Alberta
Eulithis explanata|GWNS303-07|Canada.Alberta
Eulithis destinata|GWNC384-07|Canada.British Columbia
Eulithis destinata|GWNC424-07|United States.Washington
Eulithis destinata|GWNJ136-07|United States.Washington
Eulithis destinata|DUNLP070-08|Canada.British Columbia
Eulithis destinata|GWNC421-07|Canada.Yukon Territory
Eulithis destinata|GWNC383-07|Canada.Alberta
Eulithis destinata|GWNP076-07|Canada.British Columbia
Eulithis destinata|GWNC422-07|United States.Alaska
Eulithis destinata|GWNC420-07|United States.Alaska
Eulithis destinata|GWNP077-07|Canada.British Columbia
Eulithis destinata|GWNR457-07|Canada.British Columbia
Eulithis destinata|GWNR456-07|Canada.British Columbia
Eulithis flavibrunneata|GWNC388-07|Canada.Alberta
Eulithis flavibrunneata|GWNC387-07|Canada.Alberta
Eulithis flavibrunneata|GWNS302-07|Canada.Alberta
Eulithis testata|GWNS301-07|Canada.Alberta
Eulithis testata|GWNS300-07|Canada.Alberta
Eulithis testata|GWNS299-07|Canada.Alberta
Eulithis testata|GWNS298-07|Canada.Alberta
Eulithis testata|GWNR460-07|Canada.British Columbia
Eulithis propulsata|GWNS297-07|Canada.Alberta
Eulithis propulsata|GWNS295-07|Canada.Alberta
Eulithis propulsata|GWNS296-07|Canada.Alberta
Eulithis propulsata|GWNP075-07|Canada.British Columbia
Eulithis propulsata|GWNC417-07|Canada.Alberta
Eulithis propulsata|LBCG771-09|Canada.British Columbia
Eulithis propulsata|GWNR459-07|Canada.British Columbia
Eulithis propulsata|GWNR458-07|Canada.British Columbia
Eulithis propulsata|GWNS294-07|Canada.Alberta
Eulithis propulsata|LBCG1841-09|Canada.British Columbia
Eulithis xylina|LBCG2329-09|Canada.British Columbia
Eulithis xylina|GWNR462-07|Canada.British Columbia
Eulithis xylina|LBCG2331-09|Canada.British Columbia
Eulithis xylina|GWNS308-07|Canada.Alberta
Eulithis xylina|GWNS309-07|Canada.Alberta
Eulithis xylina|GWNS307-07|Canada.Alberta
Eulithis xylina|GWNR464-07|Canada.British Columbia
Eulithis xylina|GWNP078-07|Canada.British Columbia
Eulithis xylina|GWNS518-07|Canada.Alberta
Eulithis xylina|GWNJ137-07|United States.Washington
Eulithis xylina|GWNP079-07|Canada.British Columbia
Eulithis xylina|GWNC419-07|Canada.Yukon Territory
Eulithis xylina|GWNR463-07|Canada.British Columbia
Eulithis xylina|LBCG2323-09|Canada.British Columbia
Eulithis xylina|LBCG2325-09|Canada.British Columbia
Eulithis xylina|LBCG2326-09|Canada.British Columbia
Eulithis xylina|LBCG3699-09|Canada.British Columbia
Eurhinosea flavaria|GWNC425-07|Canada.British Columbia
Thera juniperata|GWNC456-07|Canada.British Columbia
Thera juniperata|GWNC455-07|Canada.Alberta
Thera juniperata|GWNS317-07|Canada.Alberta
Thera juniperata|GWNS316-07|Canada.Alberta
Thera juniperata|GWNC404-07|Canada.Alberta
Thera juniperata|GWNS315-07|Canada.Alberta
Dysstroma mancipata|GWNJ134-07|United States.California
Thera otisi|GWNS521-07|Canada.Alberta
Thera otisi|GWNP088-07|Canada.British Columbia
Thera otisi|GWNR637-07|Canada.British Columbia
Thera otisi|GWNS318-07|Canada.Alberta
Thera otisi|GWNS495-07|Canada.Alberta
Thera otisi|GWNC432-07|Canada.Yukon Territory
Thera otisi|GWNS319-07|Canada.Alberta
Thera otisi|GWNR636-07|Canada.British Columbia
Thera otisi|GWNS494-07|Canada.Alberta
Thera otisi|GWNR635-07|Canada.British Columbia
Trichodezia albovittata|GWNS404-07|Canada.Alberta
Trichodezia albovittata|LBCA565-05|Canada.British Columbia
Trichodezia albovittata|GWNR582-07|Canada.British Columbia

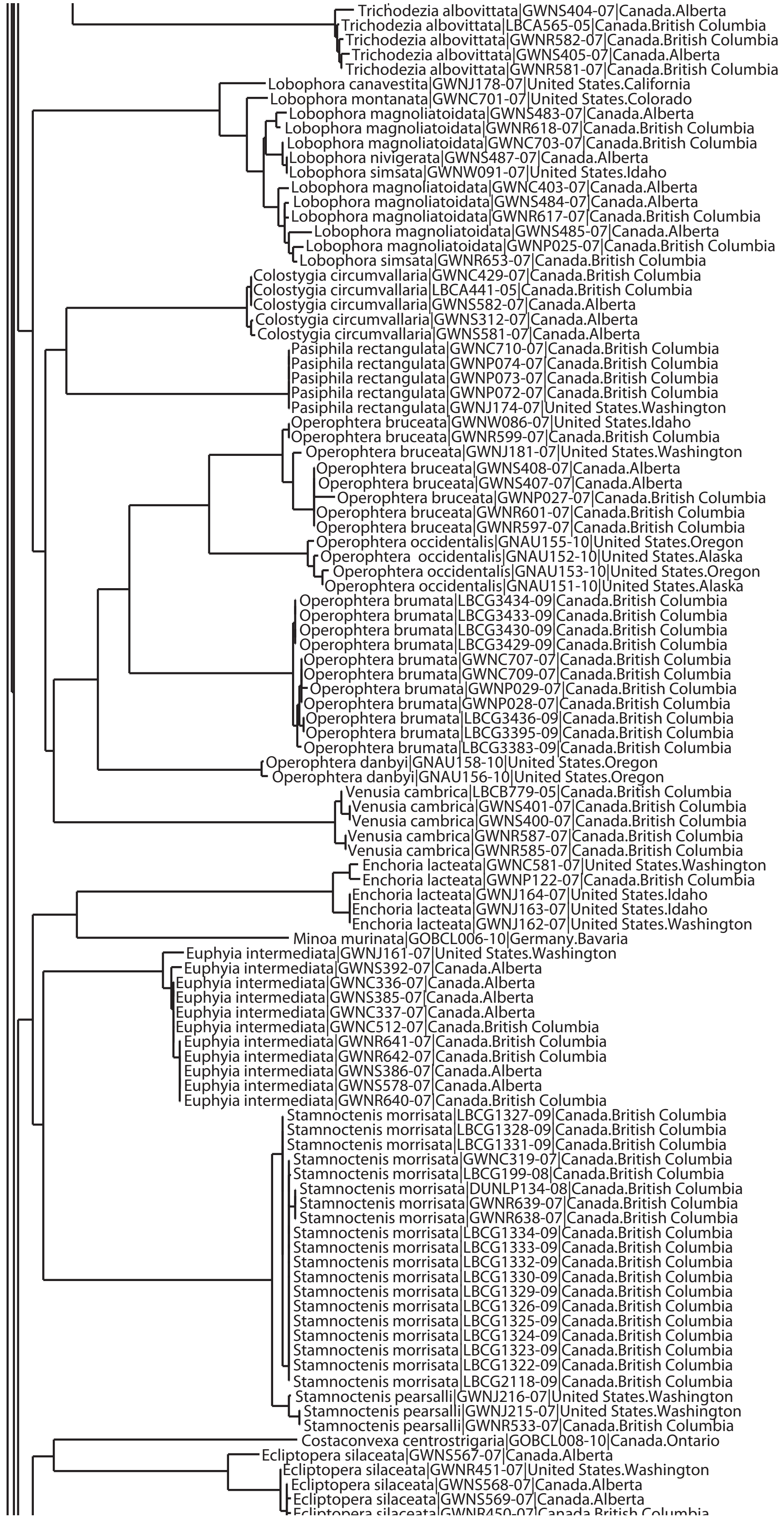

Trichodezia albovittata|GWNS404-07|Canada.Alberta
Trichodezia albovittata|LBCA565-05|Canada.British Columbia
Trichodezia albovittata|GWNR582-07|Canada.British Columbia
Trichodezia albovittata|GWNS405-07|Canada.Alberta
Trichodezia albovittata|GWNR581-07|Canada.British Columbia
Lobophora canavestita|GWNJ178-07|United States.California
Lobophora montanata|GWNC701-07|United States.Colorado
Lobophora magnoliatoidata|GWNS483-07|Canada.Alberta
Lobophora magnoliatoidata|GWNR618-07|Canada.British Columbia
Lobophora magnoliatoidata|GWNC703-07|Canada.British Columbia
Lobophora nivigerata|GWNS487-07|Canada.Alberta
Lobophora simsata|GWNW091-07|United States.Idaho
Lobophora magnoliatoidata|GWNC403-07|Canada.Alberta
Lobophora magnoliatoidata|GWNS484-07|Canada.Alberta
Lobophora magnoliatoidata|GWNR617-07|Canada.British Columbia
Lobophora magnoliatoidata|GWNS485-07|Canada.Alberta
Lobophora magnoliatoidata|GWNP025-07|Canada.British Columbia
Lobophora simsata|GWNR653-07|Canada.British Columbia
Colostygia circumvallaria|GWNC429-07|Canada.British Columbia
Colostygia circumvallaria|LBCA441-05|Canada.British Columbia
Colostygia circumvallaria|GWNS582-07|Canada.Alberta
Colostygia circumvallaria|GWNS312-07|Canada.Alberta
Colostygia circumvallaria|GWNS581-07|Canada.Alberta
Pasiphila rectangulata|GWNC710-07|Canada.British Columbia
Pasiphila rectangulata|GWNP074-07|Canada.British Columbia
Pasiphila rectangulata|GWNP073-07|Canada.British Columbia
Pasiphila rectangulata|GWNP072-07|Canada.British Columbia
Pasiphila rectangulata|GWNJ174-07|United States.Washington
Operophtera bruceata|GWNW086-07|United States.Idaho
Operophtera bruceata|GWNR599-07|Canada.British Columbia
Operophtera bruceata|GWNJ181-07|United States.Washington
Operophtera bruceata|GWNS408-07|Canada.Alberta
Operophtera bruceata|GWNS407-07|Canada.Alberta
Operophtera bruceata|GWNP027-07|Canada.British Columbia
Operophtera bruceata|GWNR601-07|Canada.British Columbia
Operophtera bruceata|GWNR597-07|Canada.British Columbia
Operophtera occidentalis|GNAU155-10|United States.Oregon
Operophtera occidentalis|GNAU152-10|United States.Alaska
Operophtera occidentalis|GNAU153-10|United States.Oregon
Operophtera occidentalis|GNAU151-10|United States.Alaska
Operophtera brumata|LBCG3434-09|Canada.British Columbia
Operophtera brumata|LBCG3433-09|Canada.British Columbia
Operophtera brumata|LBCG3430-09|Canada.British Columbia
Operophtera brumata|LBCG3429-09|Canada.British Columbia
Operophtera brumata|GWNC707-07|Canada.British Columbia
Operophtera brumata|GWNC709-07|Canada.British Columbia
Operophtera brumata|GWNP029-07|Canada.British Columbia
Operophtera brumata|GWNP028-07|Canada.British Columbia
Operophtera brumata|LBCG3436-09|Canada.British Columbia
Operophtera brumata|LBCG3395-09|Canada.British Columbia
Operophtera brumata|LBCG3383-09|Canada.British Columbia
Operophtera danbyi|GNAU158-10|United States.Oregon
Operophtera danbyi|GNAU156-10|United States.Oregon
Venusia cambrica|LBCB779-05|Canada.British Columbia
Venusia cambrica|GWNS401-07|Canada.British Columbia
Venusia cambrica|GWNS400-07|Canada.British Columbia
Venusia cambrica|GWNR587-07|Canada.British Columbia
Venusia cambrica|GWNR585-07|Canada.British Columbia
Enchoria lacteata|GWNC581-07|United States.Washington
Enchoria lacteata|GWNP122-07|Canada.British Columbia
Enchoria lacteata|GWNJ164-07|United States.Idaho
Enchoria lacteata|GWNJ163-07|United States.Idaho
Enchoria lacteata|GWNJ162-07|United States.Washington
Minoa murinata|GOBCL006-10|Germany.Bavaria
Euphyia intermediata|GWNJ161-07|United States.Washington
Euphyia intermediata|GWNS392-07|Canada.Alberta
Euphyia intermediata|GWNC336-07|Canada.Alberta
Euphyia intermediata|GWNS385-07|Canada.Alberta
Euphyia intermediata|GWNC337-07|Canada.Alberta
Euphyia intermediata|GWNC512-07|Canada.British Columbia
Euphyia intermediata|GWNR641-07|Canada.British Columbia
Euphyia intermediata|GWNR642-07|Canada.British Columbia
Euphyia intermediata|GWNS386-07|Canada.Alberta
Euphyia intermediata|GWNS578-07|Canada.Alberta
Euphyia intermediata|GWNR640-07|Canada.British Columbia
Stamnoctenis morrisata|LBCG1327-09|Canada.British Columbia
Stamnoctenis morrisata|LBCG1328-09|Canada.British Columbia
Stamnoctenis morrisata|LBCG1331-09|Canada.British Columbia
Stamnoctenis morrisata|GWNC319-07|Canada.British Columbia
Stamnoctenis morrisata|LBCG199-08|Canada.British Columbia
Stamnoctenis morrisata|DUNLP134-08|Canada.British Columbia
Stamnoctenis morrisata|GWNR639-07|Canada.British Columbia
Stamnoctenis morrisata|GWNR638-07|Canada.British Columbia
Stamnoctenis morrisata|LBCG1334-09|Canada.British Columbia
Stamnoctenis morrisata|LBCG1333-09|Canada.British Columbia
Stamnoctenis morrisata|LBCG1332-09|Canada.British Columbia
Stamnoctenis morrisata|LBCG1330-09|Canada.British Columbia
Stamnoctenis morrisata|LBCG1329-09|Canada.British Columbia
Stamnoctenis morrisata|LBCG1326-09|Canada.British Columbia
Stamnoctenis morrisata|LBCG1325-09|Canada.British Columbia
Stamnoctenis morrisata|LBCG1324-09|Canada.British Columbia
Stamnoctenis morrisata|LBCG1323-09|Canada.British Columbia
Stamnoctenis morrisata|LBCG1322-09|Canada.British Columbia
Stamnoctenis morrisata|LBCG2118-09|Canada.British Columbia
Stamnoctenis pearsalli|GWNJ216-07|United States.Washington
Stamnoctenis pearsalli|GWNJ215-07|United States.Washington
Stamnoctenis pearsalli|GWNR533-07|Canada.British Columbia
Costaconvexa centrostrigaria|GOBCL008-10|Canada.Ontario
Ecliptopera silaceata|GWNS567-07|Canada.Alberta
Ecliptopera silaceata|GWNR451-07|United States.Washington
Ecliptopera silaceata|GWNS568-07|Canada.Alberta
Ecliptopera silaceata|GWNS569-07|Canada.Alberta
Ecliptopera silaceata|GWNR450-07|Canada.British Columbia

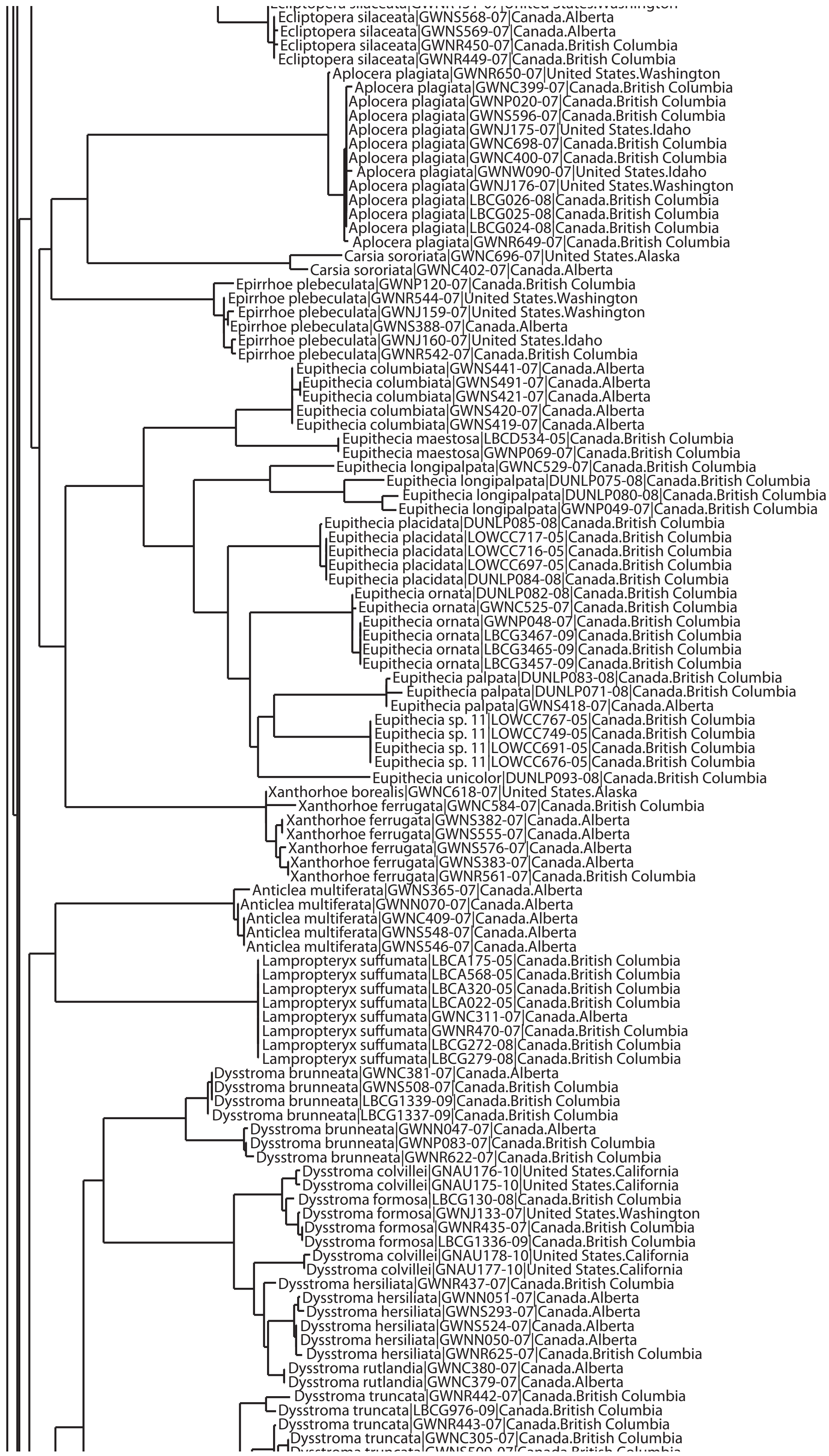

Ecliptopera silaceata|GWNS568-07|Canada.Alberta
Ecliptopera silaceata|GWNS569-07|Canada.Alberta
Ecliptopera silaceata|GWNR450-07|Canada.British Columbia
Ecliptopera silaceata|GWNR449-07|Canada.British Columbia
Aplocera plagiata|GWNR650-07|United States.Washington
Aplocera plagiata|GWNC399-07|Canada.British Columbia
Aplocera plagiata|GWNP020-07|Canada.British Columbia
Aplocera plagiata|GWNS596-07|Canada.British Columbia
Aplocera plagiata|GWNJ175-07|United States.Idaho
Aplocera plagiata|GWNC698-07|Canada.British Columbia
Aplocera plagiata|GWNC400-07|Canada.British Columbia
Aplocera plagiata|GWNW090-07|United States.Idaho
Aplocera plagiata|GWNJ176-07|United States.Washington
Aplocera plagiata|LBCG026-08|Canada.British Columbia
Aplocera plagiata|LBCG025-08|Canada.British Columbia
Aplocera plagiata|LBCG024-08|Canada.British Columbia
Aplocera plagiata|GWNR649-07|Canada.British Columbia
Carsia sororiata|GWNC696-07|United States.Alaska
Carsia sororiata|GWNC402-07|Canada.Alberta
Epirrhoe plebeculata|GWNP120-07|Canada.British Columbia
Epirrhoe plebeculata|GWNR544-07|United States.Washington
Epirrhoe plebeculata|GWNJ159-07|United States.Washington
Epirrhoe plebeculata|GWNS388-07|Canada.Alberta
Epirrhoe plebeculata|GWNJ160-07|United States.Idaho
Epirrhoe plebeculata|GWNR542-07|Canada.British Columbia
Eupithecia columbiata|GWNS441-07|Canada.Alberta
Eupithecia columbiata|GWNS491-07|Canada.Alberta
Eupithecia columbiata|GWNS421-07|Canada.Alberta
Eupithecia columbiata|GWNS420-07|Canada.Alberta
Eupithecia columbiata|GWNS419-07|Canada.Alberta
Eupithecia maestosa|LBCD534-05|Canada.British Columbia
Eupithecia maestosa|GWNP069-07|Canada.British Columbia
Eupithecia longipalpata|GWNC529-07|Canada.British Columbia
Eupithecia longipalpata|DUNLP075-08|Canada.British Columbia
Eupithecia longipalpata|DUNLP080-08|Canada.British Columbia
Eupithecia longipalpata|GWNP049-07|Canada.British Columbia
Eupithecia placidata|DUNLP085-08|Canada.British Columbia
Eupithecia placidata|LOWCC717-05|Canada.British Columbia
Eupithecia placidata|LOWCC716-05|Canada.British Columbia
Eupithecia placidata|LOWCC697-05|Canada.British Columbia
Eupithecia placidata|DUNLP084-08|Canada.British Columbia
Eupithecia ornata|DUNLP082-08|Canada.British Columbia
Eupithecia ornata|GWNC525-07|Canada.British Columbia
Eupithecia ornata|GWNP048-07|Canada.British Columbia
Eupithecia ornata|LBCG3467-09|Canada.British Columbia
Eupithecia ornata|LBCG3465-09|Canada.British Columbia
Eupithecia ornata|LBCG3457-09|Canada.British Columbia
Eupithecia palpata|DUNLP083-08|Canada.British Columbia
Eupithecia palpata|DUNLP071-08|Canada.British Columbia
Eupithecia palpata|GWNS418-07|Canada.Alberta
Eupithecia sp. 11|LOWCC767-05|Canada.British Columbia
Eupithecia sp. 11|LOWCC749-05|Canada.British Columbia
Eupithecia sp. 11|LOWCC691-05|Canada.British Columbia
Eupithecia sp. 11|LOWCC676-05|Canada.British Columbia
Eupithecia unicolor|DUNLP093-08|Canada.British Columbia
Xanthorhoe borealis|GWNC618-07|United States.Alaska
Xanthorhoe ferrugata|GWNC584-07|Canada.British Columbia
Xanthorhoe ferrugata|GWNS382-07|Canada.Alberta
Xanthorhoe ferrugata|GWNS555-07|Canada.Alberta
Xanthorhoe ferrugata|GWNS576-07|Canada.Alberta
Xanthorhoe ferrugata|GWNS383-07|Canada.Alberta
Xanthorhoe ferrugata|GWNR561-07|Canada.British Columbia
Anticlea multiferata|GWNS365-07|Canada.Alberta
Anticlea multiferata|GWNN070-07|Canada.Alberta
Anticlea multiferata|GWNC409-07|Canada.Alberta
Anticlea multiferata|GWNS548-07|Canada.Alberta
Anticlea multiferata|GWNS546-07|Canada.Alberta
Lampropteryx suffumata|LBCA175-05|Canada.British Columbia
Lampropteryx suffumata|LBCA568-05|Canada.British Columbia
Lampropteryx suffumata|LBCA320-05|Canada.British Columbia
Lampropteryx suffumata|LBCA022-05|Canada.British Columbia
Lampropteryx suffumata|GWNC311-07|Canada.Alberta
Lampropteryx suffumata|GWNR470-07|Canada.British Columbia
Lampropteryx suffumata|LBCG272-08|Canada.British Columbia
Lampropteryx suffumata|LBCG279-08|Canada.British Columbia
Dysstroma brunneata|GWNC381-07|Canada.Alberta
Dysstroma brunneata|GWNS508-07|Canada.British Columbia
Dysstroma brunneata|LBCG1339-09|Canada.British Columbia
Dysstroma brunneata|LBCG1337-09|Canada.British Columbia
Dysstroma brunneata|GWNN047-07|Canada.Alberta
Dysstroma brunneata|GWNP083-07|Canada.British Columbia
Dysstroma brunneata|GWNR622-07|Canada.British Columbia
Dysstroma colvillei|GNAU176-10|United States.California
Dysstroma colvillei|GNAU175-10|United States.California
Dysstroma formosa|LBCG130-08|Canada.British Columbia
Dysstroma formosa|GWNJ133-07|United States.Washington
Dysstroma formosa|GWNR435-07|Canada.British Columbia
Dysstroma formosa|LBCG1336-09|Canada.British Columbia
Dysstroma colvillei|GNAU178-10|United States.California
Dysstroma colvillei|GNAU177-10|United States.California
Dysstroma hersiliata|GWNR437-07|Canada.British Columbia
Dysstroma hersiliata|GWNN051-07|Canada.Alberta
Dysstroma hersiliata|GWNS293-07|Canada.Alberta
Dysstroma hersiliata|GWNS524-07|Canada.Alberta
Dysstroma hersiliata|GWNN050-07|Canada.Alberta
Dysstroma hersiliata|GWNR625-07|Canada.British Columbia
Dysstroma rutlandia|GWNC380-07|Canada.Alberta
Dysstroma rutlandia|GWNC379-07|Canada.Alberta
Dysstroma truncata|GWNR442-07|Canada.British Columbia
Dysstroma truncata|LBCG976-09|Canada.British Columbia
Dysstroma truncata|GWNR443-07|Canada.British Columbia
Dysstroma truncata|GWNC305-07|Canada.British Columbia

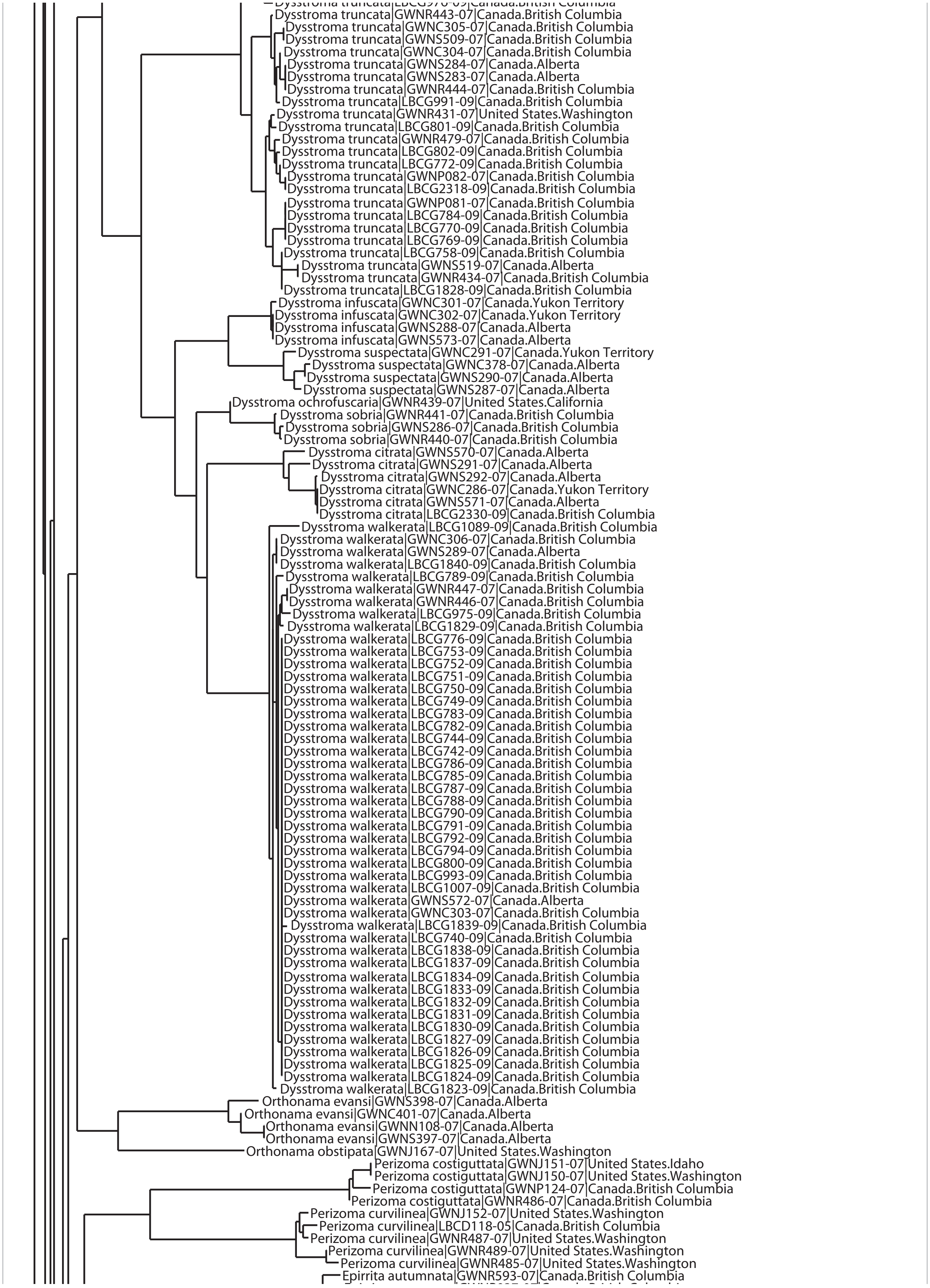
Dysstroma truncata|GWNR443-07|Canada.British Columbia
Dysstroma truncata|GWNC305-07|Canada.British Columbia
Dysstroma truncata|GWNS509-07|Canada.British Columbia
Dysstroma truncata|GWNC304-07|Canada.British Columbia
Dysstroma truncata|GWNS284-07|Canada.Alberta
Dysstroma truncata|GWNS283-07|Canada.Alberta
Dysstroma truncata|GWNR444-07|Canada.British Columbia
Dysstroma truncata|LBCG991-09|Canada.British Columbia
Dysstroma truncata|GWNR431-07|United States.Washington
Dysstroma truncata|LBCG801-09|Canada.British Columbia
Dysstroma truncata|GWNR479-07|Canada.British Columbia
Dysstroma truncata|LBCG802-09|Canada.British Columbia
Dysstroma truncata|LBCG772-09|Canada.British Columbia
Dysstroma truncata|GWNP082-07|Canada.British Columbia
Dysstroma truncata|LBCG2318-09|Canada.British Columbia
Dysstroma truncata|GWNP081-07|Canada.British Columbia
Dysstroma truncata|LBCG784-09|Canada.British Columbia
Dysstroma truncata|LBCG770-09|Canada.British Columbia
Dysstroma truncata|LBCG769-09|Canada.British Columbia
Dysstroma truncata|LBCG758-09|Canada.British Columbia
Dysstroma truncata|GWNS519-07|Canada.Alberta
Dysstroma truncata|GWNR434-07|Canada.British Columbia
Dysstroma truncata|LBCG1828-09|Canada.British Columbia
Dysstroma infuscata|GWNC301-07|Canada.Yukon Territory
Dysstroma infuscata|GWNC302-07|Canada.Yukon Territory
Dysstroma infuscata|GWNS288-07|Canada.Alberta
Dysstroma infuscata|GWNS573-07|Canada.Alberta
Dysstroma suspectata|GWNC291-07|Canada.Yukon Territory
Dysstroma suspectata|GWNC378-07|Canada.Alberta
Dysstroma suspectata|GWNS290-07|Canada.Alberta
Dysstroma suspectata|GWNS287-07|Canada.Alberta
Dysstroma ochrofuscaria|GWNR439-07|United States.California
Dysstroma sobria|GWNR441-07|Canada.British Columbia
Dysstroma sobria|GWNS286-07|Canada.British Columbia
Dysstroma sobria|GWNR440-07|Canada.British Columbia
Dysstroma citrata|GWNS570-07|Canada.Alberta
Dysstroma citrata|GWNS291-07|Canada.Alberta
Dysstroma citrata|GWNS292-07|Canada.Alberta
Dysstroma citrata|GWNC286-07|Canada.Yukon Territory
Dysstroma citrata|GWNS571-07|Canada.Alberta
Dysstroma citrata|LBCG2330-09|Canada.British Columbia
Dysstroma walkerata|LBCG1089-09|Canada.British Columbia
Dysstroma walkerata|GWNC306-07|Canada.British Columbia
Dysstroma walkerata|GWNS289-07|Canada.Alberta
Dysstroma walkerata|LBCG1840-09|Canada.British Columbia
Dysstroma walkerata|LBCG789-09|Canada.British Columbia
Dysstroma walkerata|GWNR447-07|Canada.British Columbia
Dysstroma walkerata|GWNR446-07|Canada.British Columbia
Dysstroma walkerata|LBCG975-09|Canada.British Columbia
Dysstroma walkerata|LBCG1829-09|Canada.British Columbia
Dysstroma walkerata|LBCG776-09|Canada.British Columbia
Dysstroma walkerata|LBCG753-09|Canada.British Columbia
Dysstroma walkerata|LBCG752-09|Canada.British Columbia
Dysstroma walkerata|LBCG751-09|Canada.British Columbia
Dysstroma walkerata|LBCG750-09|Canada.British Columbia
Dysstroma walkerata|LBCG749-09|Canada.British Columbia
Dysstroma walkerata|LBCG783-09|Canada.British Columbia
Dysstroma walkerata|LBCG782-09|Canada.British Columbia
Dysstroma walkerata|LBCG744-09|Canada.British Columbia
Dysstroma walkerata|LBCG742-09|Canada.British Columbia
Dysstroma walkerata|LBCG786-09|Canada.British Columbia
Dysstroma walkerata|LBCG785-09|Canada.British Columbia
Dysstroma walkerata|LBCG787-09|Canada.British Columbia
Dysstroma walkerata|LBCG788-09|Canada.British Columbia
Dysstroma walkerata|LBCG790-09|Canada.British Columbia
Dysstroma walkerata|LBCG791-09|Canada.British Columbia
Dysstroma walkerata|LBCG792-09|Canada.British Columbia
Dysstroma walkerata|LBCG794-09|Canada.British Columbia
Dysstroma walkerata|LBCG800-09|Canada.British Columbia
Dysstroma walkerata|LBCG993-09|Canada.British Columbia
Dysstroma walkerata|LBCG1007-09|Canada.British Columbia
Dysstroma walkerata|GWNS572-07|Canada.Alberta
Dysstroma walkerata|GWNC303-07|Canada.British Columbia
Dysstroma walkerata|LBCG1839-09|Canada.British Columbia
Dysstroma walkerata|LBCG740-09|Canada.British Columbia
Dysstroma walkerata|LBCG1838-09|Canada.British Columbia
Dysstroma walkerata|LBCG1837-09|Canada.British Columbia
Dysstroma walkerata|LBCG1834-09|Canada.British Columbia
Dysstroma walkerata|LBCG1833-09|Canada.British Columbia
Dysstroma walkerata|LBCG1832-09|Canada.British Columbia
Dysstroma walkerata|LBCG1831-09|Canada.British Columbia
Dysstroma walkerata|LBCG1830-09|Canada.British Columbia
Dysstroma walkerata|LBCG1827-09|Canada.British Columbia
Dysstroma walkerata|LBCG1826-09|Canada.British Columbia
Dysstroma walkerata|LBCG1825-09|Canada.British Columbia
Dysstroma walkerata|LBCG1824-09|Canada.British Columbia
Dysstroma walkerata|LBCG1823-09|Canada.British Columbia
Orthonama evansi|GWNS398-07|Canada.Alberta
Orthonama evansi|GWNC401-07|Canada.Alberta
Orthonama evansi|GWNN108-07|Canada.Alberta
Orthonama evansi|GWNS397-07|Canada.Alberta
Orthonama obstipata|GWNJ167-07|United States.Washington
Perizoma costiguttata|GWNJ151-07|United States.Idaho
Perizoma costiguttata|GWNJ150-07|United States.Washington
Perizoma costiguttata|GWNP124-07|Canada.British Columbia
Perizoma costiguttata|GWNR486-07|Canada.British Columbia
Perizoma curvilinea|GWNJ152-07|United States.Washington
Perizoma curvilinea|LBCD118-05|Canada.British Columbia
Perizoma curvilinea|GWNR487-07|United States.Washington
Perizoma curvilinea|GWNR489-07|United States.Washington
Perizoma curvilinea|GWNR485-07|United States.Washington
Epirrita autumnata|GWNR593-07|Canada.British Columbia

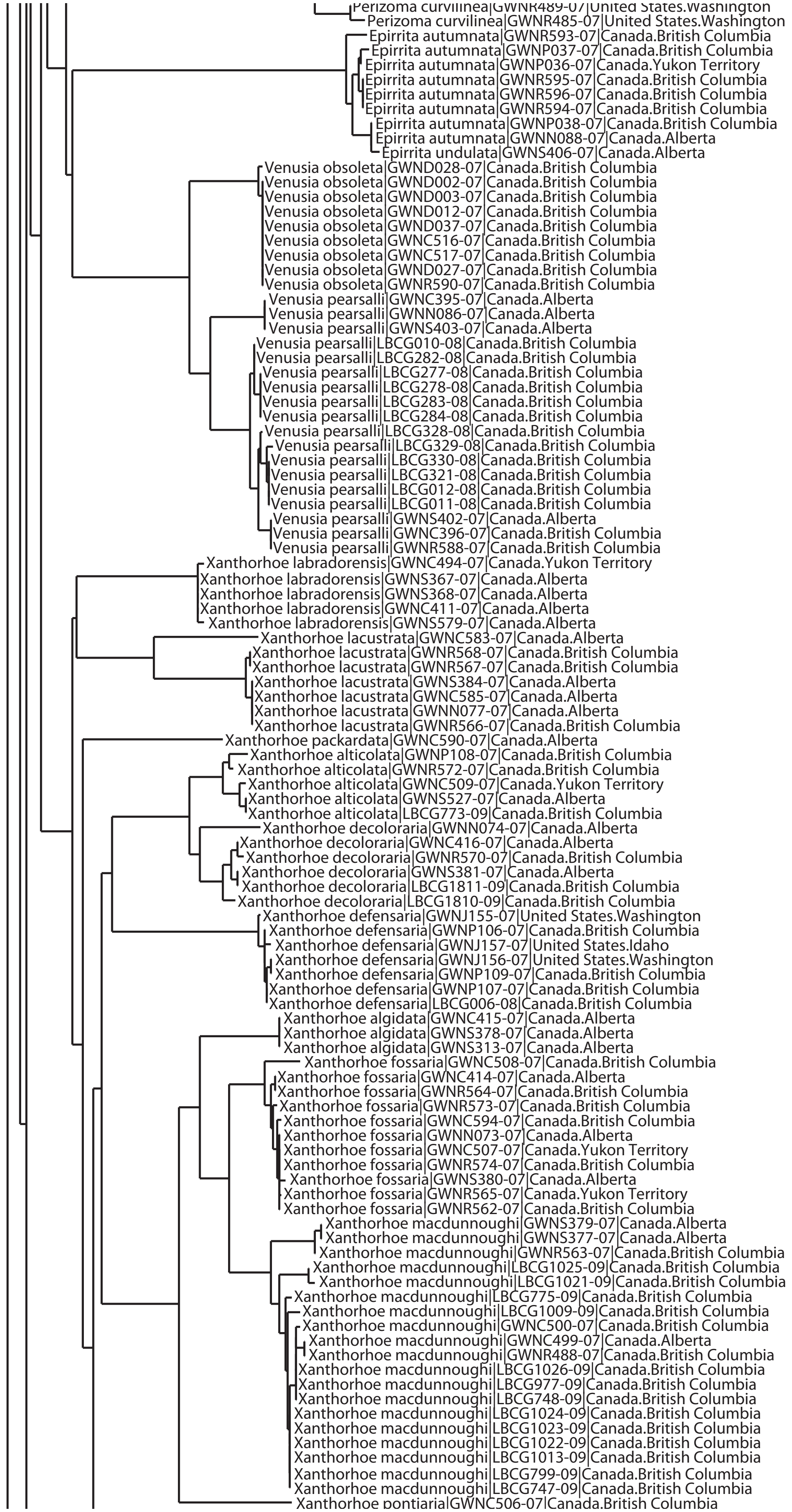

Perizoma curvilinea|GWNR489-07|United States.Washington
Perizoma curvilinea|GWNR485-07|United States.Washington
Epirrita autumnata|GWNR593-07|Canada.British Columbia
Epirrita autumnata|GWNP037-07|Canada.British Columbia
Epirrita autumnata|GWNP036-07|Canada.Yukon Territory
Epirrita autumnata|GWNR595-07|Canada.British Columbia
Epirrita autumnata|GWNR596-07|Canada.British Columbia
Epirrita autumnata|GWNR594-07|Canada.British Columbia
Epirrita autumnata|GWNP038-07|Canada.British Columbia
Epirrita autumnata|GWNN088-07|Canada.Alberta
Epirrita undulata|GWNS406-07|Canada.Alberta
Venusia obsoleta|GWND028-07|Canada.British Columbia
Venusia obsoleta|GWND002-07|Canada.British Columbia
Venusia obsoleta|GWND003-07|Canada.British Columbia
Venusia obsoleta|GWND012-07|Canada.British Columbia
Venusia obsoleta|GWND037-07|Canada.British Columbia
Venusia obsoleta|GWNC516-07|Canada.British Columbia
Venusia obsoleta|GWNC517-07|Canada.British Columbia
Venusia obsoleta|GWND027-07|Canada.British Columbia
Venusia obsoleta|GWNR590-07|Canada.British Columbia
Venusia pearsalli|GWNC395-07|Canada.Alberta
Venusia pearsalli|GWNN086-07|Canada.Alberta
Venusia pearsalli|GWNS403-07|Canada.Alberta
Venusia pearsalli|LBCG010-08|Canada.British Columbia
Venusia pearsalli|LBCG282-08|Canada.British Columbia
Venusia pearsalli|LBCG277-08|Canada.British Columbia
Venusia pearsalli|LBCG278-08|Canada.British Columbia
Venusia pearsalli|LBCG283-08|Canada.British Columbia
Venusia pearsalli|LBCG284-08|Canada.British Columbia
Venusia pearsalli|LBCG328-08|Canada.British Columbia
Venusia pearsalli|LBCG329-08|Canada.British Columbia
Venusia pearsalli|LBCG330-08|Canada.British Columbia
Venusia pearsalli|LBCG321-08|Canada.British Columbia
Venusia pearsalli|LBCG012-08|Canada.British Columbia
Venusia pearsalli|LBCG011-08|Canada.British Columbia
Venusia pearsalli|GWNS402-07|Canada.Alberta
Venusia pearsalli|GWNC396-07|Canada.British Columbia
Venusia pearsalli|GWNR588-07|Canada.British Columbia
Xanthorhoe labradorensis|GWNC494-07|Canada.Yukon Territory
Xanthorhoe labradorensis|GWNS367-07|Canada.Alberta
Xanthorhoe labradorensis|GWNS368-07|Canada.Alberta
Xanthorhoe labradorensis|GWNC411-07|Canada.Alberta
Xanthorhoe labradorensis|GWNS579-07|Canada.Alberta
Xanthorhoe lacustrata|GWNC583-07|Canada.Alberta
Xanthorhoe lacustrata|GWNR568-07|Canada.British Columbia
Xanthorhoe lacustrata|GWNR567-07|Canada.British Columbia
Xanthorhoe lacustrata|GWNS384-07|Canada.Alberta
Xanthorhoe lacustrata|GWNC585-07|Canada.Alberta
Xanthorhoe lacustrata|GWNN077-07|Canada.Alberta
Xanthorhoe lacustrata|GWNR566-07|Canada.British Columbia
Xanthorhoe packardata|GWNC590-07|Canada.Alberta
Xanthorhoe alticolata|GWNP108-07|Canada.British Columbia
Xanthorhoe alticolata|GWNR572-07|Canada.British Columbia
Xanthorhoe alticolata|GWNC509-07|Canada.Yukon Territory
Xanthorhoe alticolata|GWNS527-07|Canada.Alberta
Xanthorhoe alticolata|LBCG773-09|Canada.British Columbia
Xanthorhoe decoloraria|GWNN074-07|Canada.Alberta
Xanthorhoe decoloraria|GWNC416-07|Canada.Alberta
Xanthorhoe decoloraria|GWNR570-07|Canada.British Columbia
Xanthorhoe decoloraria|GWNS381-07|Canada.Alberta
Xanthorhoe decoloraria|LBCG1811-09|Canada.British Columbia
Xanthorhoe decoloraria|LBCG1810-09|Canada.British Columbia
Xanthorhoe defensaria|GWNJ155-07|United States.Washington
Xanthorhoe defensaria|GWNP106-07|Canada.British Columbia
Xanthorhoe defensaria|GWNJ157-07|United States.Idaho
Xanthorhoe defensaria|GWNJ156-07|United States.Washington
Xanthorhoe defensaria|GWNP109-07|Canada.British Columbia
Xanthorhoe defensaria|GWNP107-07|Canada.British Columbia
Xanthorhoe defensaria|LBCG006-08|Canada.British Columbia
Xanthorhoe algidata|GWNC415-07|Canada.Alberta
Xanthorhoe algidata|GWNS378-07|Canada.Alberta
Xanthorhoe algidata|GWNS313-07|Canada.Alberta
Xanthorhoe fossaria|GWNC508-07|Canada.British Columbia
Xanthorhoe fossaria|GWNC414-07|Canada.Alberta
Xanthorhoe fossaria|GWNR564-07|Canada.British Columbia
Xanthorhoe fossaria|GWNR573-07|Canada.British Columbia
Xanthorhoe fossaria|GWNC594-07|Canada.British Columbia
Xanthorhoe fossaria|GWNN073-07|Canada.Alberta
Xanthorhoe fossaria|GWNC507-07|Canada.Yukon Territory
Xanthorhoe fossaria|GWNR574-07|Canada.British Columbia
Xanthorhoe fossaria|GWNS380-07|Canada.Alberta
Xanthorhoe fossaria|GWNR565-07|Canada.Yukon Territory
Xanthorhoe fossaria|GWNR562-07|Canada.British Columbia
Xanthorhoe macdunnoughi|GWNS379-07|Canada.Alberta
Xanthorhoe macdunnoughi|GWNS377-07|Canada.Alberta
Xanthorhoe macdunnoughi|GWNR563-07|Canada.British Columbia
Xanthorhoe macdunnoughi|LBCG1025-09|Canada.British Columbia
Xanthorhoe macdunnoughi|LBCG1021-09|Canada.British Columbia
Xanthorhoe macdunnoughi|LBCG775-09|Canada.British Columbia
Xanthorhoe macdunnoughi|LBCG1009-09|Canada.British Columbia
Xanthorhoe macdunnoughi|GWNC500-07|Canada.British Columbia
Xanthorhoe macdunnoughi|GWNC499-07|Canada.Alberta
Xanthorhoe macdunnoughi|GWNR488-07|Canada.British Columbia
Xanthorhoe macdunnoughi|LBCG1026-09|Canada.British Columbia
Xanthorhoe macdunnoughi|LBCG977-09|Canada.British Columbia
Xanthorhoe macdunnoughi|LBCG748-09|Canada.British Columbia
Xanthorhoe macdunnoughi|LBCG1024-09|Canada.British Columbia
Xanthorhoe macdunnoughi|LBCG1023-09|Canada.British Columbia
Xanthorhoe macdunnoughi|LBCG1022-09|Canada.British Columbia
Xanthorhoe macdunnoughi|LBCG1013-09|Canada.British Columbia
Xanthorhoe macdunnoughi|LBCG799-09|Canada.British Columbia
Xanthorhoe macdunnoughi|LBCG747-09|Canada.British Columbia
Xanthorhoe pontiaria|GWNC506-07|Canada.British Columbia

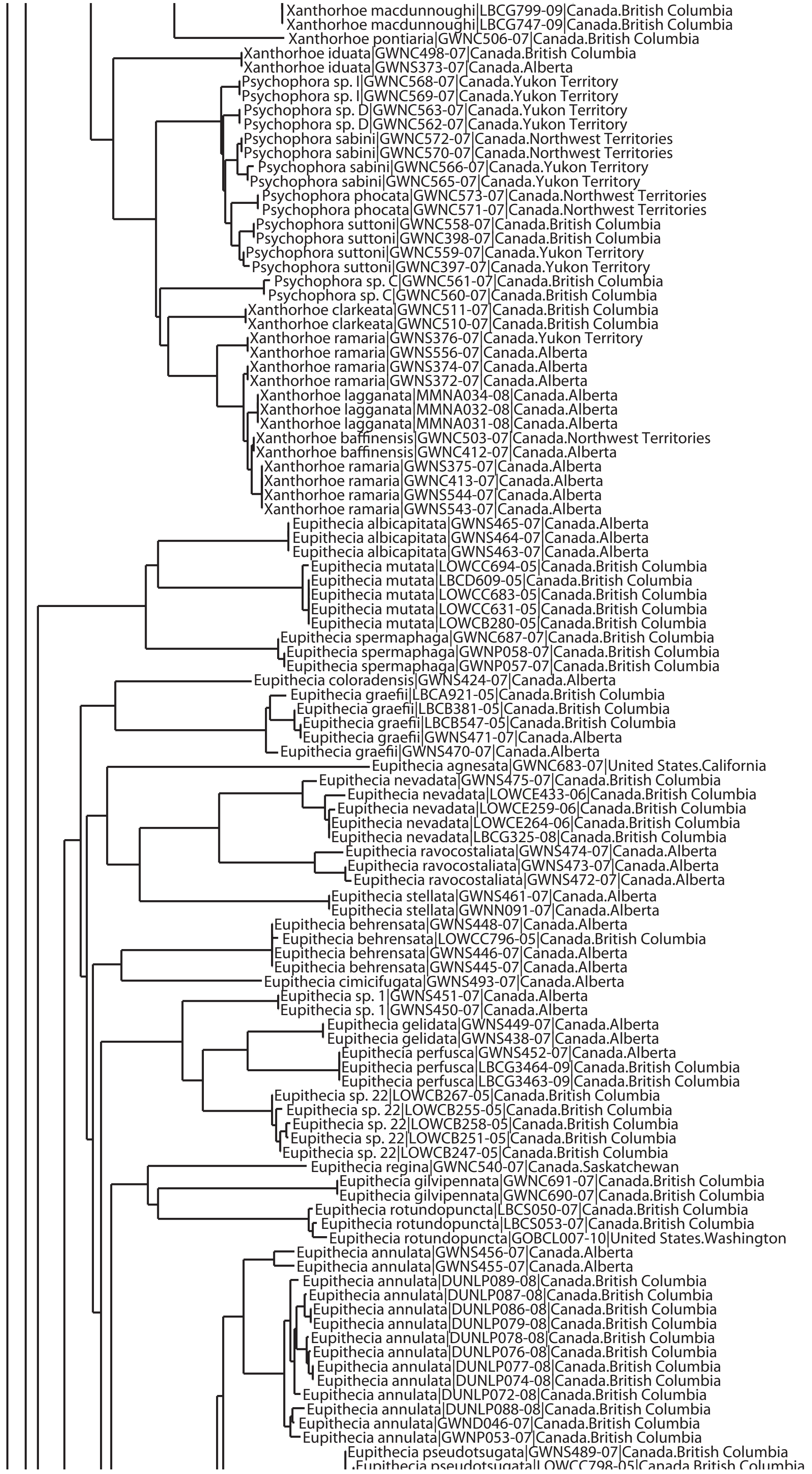

Xanthorhoe macdunnoughi|LBCG799-09|Canada.British Columbia
Xanthorhoe macdunnoughi|LBCG747-09|Canada.British Columbia
Xanthorhoe pontiaria|GWNC506-07|Canada.British Columbia
Xanthorhoe iduata|GWNC498-07|Canada.British Columbia
Xanthorhoe iduata|GWNS373-07|Canada.Alberta
Psychophora sp. I|GWNC568-07|Canada.Yukon Territory
Psychophora sp. I|GWNC569-07|Canada.Yukon Territory
Psychophora sp. D|GWNC563-07|Canada.Yukon Territory
Psychophora sp. D|GWNC562-07|Canada.Yukon Territory
Psychophora sabini|GWNC572-07|Canada.Northwest Territories
Psychophora sabini|GWNC570-07|Canada.Northwest Territories
Psychophora sabini|GWNC566-07|Canada.Yukon Territory
Psychophora sabini|GWNC565-07|Canada.Yukon Territory
Psychophora phocata|GWNC573-07|Canada.Northwest Territories
Psychophora phocata|GWNC571-07|Canada.Northwest Territories
Psychophora suttoni|GWNC558-07|Canada.British Columbia
Psychophora suttoni|GWNC398-07|Canada.British Columbia
Psychophora suttoni|GWNC559-07|Canada.Yukon Territory
Psychophora suttoni|GWNC397-07|Canada.Yukon Territory
Psychophora sp. C|GWNC561-07|Canada.British Columbia
Psychophora sp. C|GWNC560-07|Canada.British Columbia
Xanthorhoe clarkeata|GWNC511-07|Canada.British Columbia
Xanthorhoe clarkeata|GWNC510-07|Canada.British Columbia
Xanthorhoe ramaria|GWNS376-07|Canada.Yukon Territory
Xanthorhoe ramaria|GWNS556-07|Canada.Alberta
Xanthorhoe ramaria|GWNS374-07|Canada.Alberta
Xanthorhoe ramaria|GWNS372-07|Canada.Alberta
Xanthorhoe lagganata|MMNA034-08|Canada.Alberta
Xanthorhoe lagganata|MMNA032-08|Canada.Alberta
Xanthorhoe lagganata|MMNA031-08|Canada.Alberta
Xanthorhoe baffinensis|GWNC503-07|Canada.Northwest Territories
Xanthorhoe baffinensis|GWNC412-07|Canada.Alberta
Xanthorhoe ramaria|GWNS375-07|Canada.Alberta
Xanthorhoe ramaria|GWNC413-07|Canada.Alberta
Xanthorhoe ramaria|GWNS544-07|Canada.Alberta
Xanthorhoe ramaria|GWNS543-07|Canada.Alberta
Eupithecia albicapitata|GWNS465-07|Canada.Alberta
Eupithecia albicapitata|GWNS464-07|Canada.Alberta
Eupithecia albicapitata|GWNS463-07|Canada.Alberta
Eupithecia mutata|LOWCC694-05|Canada.British Columbia
Eupithecia mutata|LBCD609-05|Canada.British Columbia
Eupithecia mutata|LOWCC683-05|Canada.British Columbia
Eupithecia mutata|LOWCC631-05|Canada.British Columbia
Eupithecia mutata|LOWCB280-05|Canada.British Columbia
Eupithecia spermaphaga|GWNC687-07|Canada.British Columbia
Eupithecia spermaphaga|GWNP058-07|Canada.British Columbia
Eupithecia spermaphaga|GWNP057-07|Canada.British Columbia
Eupithecia coloradensis|GWNS424-07|Canada.Alberta
Eupithecia graefii|LBCA921-05|Canada.British Columbia
Eupithecia graefii|LBCB381-05|Canada.British Columbia
Eupithecia graefii|LBCB547-05|Canada.British Columbia
Eupithecia graefii|GWNS471-07|Canada.Alberta
Eupithecia graefii|GWNS470-07|Canada.Alberta
Eupithecia agnesata|GWNC683-07|United States.California
Eupithecia nevadata|GWNS475-07|Canada.British Columbia
Eupithecia nevadata|LOWCE433-06|Canada.British Columbia
Eupithecia nevadata|LOWCE259-06|Canada.British Columbia
Eupithecia nevadata|LOWCE264-06|Canada.British Columbia
Eupithecia nevadata|LBCG325-08|Canada.British Columbia
Eupithecia ravocostaliata|GWNS474-07|Canada.Alberta
Eupithecia ravocostaliata|GWNS473-07|Canada.Alberta
Eupithecia ravocostaliata|GWNS472-07|Canada.Alberta
Eupithecia stellata|GWNS461-07|Canada.Alberta
Eupithecia stellata|GWNN091-07|Canada.Alberta
Eupithecia behrensata|GWNS448-07|Canada.Alberta
Eupithecia behrensata|LOWCC796-05|Canada.British Columbia
Eupithecia behrensata|GWNS446-07|Canada.Alberta
Eupithecia behrensata|GWNS445-07|Canada.Alberta
Eupithecia cimicifugata|GWNS493-07|Canada.Alberta
Eupithecia sp. 1|GWNS451-07|Canada.Alberta
Eupithecia sp. 1|GWNS450-07|Canada.Alberta
Eupithecia gelidata|GWNS449-07|Canada.Alberta
Eupithecia gelidata|GWNS438-07|Canada.Alberta
Eupithecia perfusca|GWNS452-07|Canada.Alberta
Eupithecia perfusca|LBCG3464-09|Canada.British Columbia
Eupithecia perfusca|LBCG3463-09|Canada.British Columbia
Eupithecia sp. 22|LOWCB267-05|Canada.British Columbia
Eupithecia sp. 22|LOWCB255-05|Canada.British Columbia
Eupithecia sp. 22|LOWCB258-05|Canada.British Columbia
Eupithecia sp. 22|LOWCB251-05|Canada.British Columbia
Eupithecia sp. 22|LOWCB247-05|Canada.British Columbia
Eupithecia regina|GWNC540-07|Canada.Saskatchewan
Eupithecia gilvipennata|GWNC691-07|Canada.British Columbia
Eupithecia gilvipennata|GWNC690-07|Canada.British Columbia
Eupithecia rotundopuncta|LBCS050-07|Canada.British Columbia
Eupithecia rotundopuncta|LBCS053-07|Canada.British Columbia
Eupithecia rotundopuncta|GOBCL007-10|United States.Washington
Eupithecia annulata|GWNS456-07|Canada.Alberta
Eupithecia annulata|GWNS455-07|Canada.Alberta
Eupithecia annulata|DUNLP089-08|Canada.British Columbia
Eupithecia annulata|DUNLP087-08|Canada.British Columbia
Eupithecia annulata|DUNLP086-08|Canada.British Columbia
Eupithecia annulata|DUNLP079-08|Canada.British Columbia
Eupithecia annulata|DUNLP078-08|Canada.British Columbia
Eupithecia annulata|DUNLP076-08|Canada.British Columbia
Eupithecia annulata|DUNLP077-08|Canada.British Columbia
Eupithecia annulata|DUNLP074-08|Canada.British Columbia
Eupithecia annulata|DUNLP072-08|Canada.British Columbia
Eupithecia annulata|DUNLP088-08|Canada.British Columbia
Eupithecia annulata|GWND046-07|Canada.British Columbia
Eupithecia annulata|GWNP053-07|Canada.British Columbia
Eupithecia pseudotsugata|GWNS489-07|Canada.British Columbia
Eupithecia pseudotsugata|LOWCC798-05|Canada.British Columbia

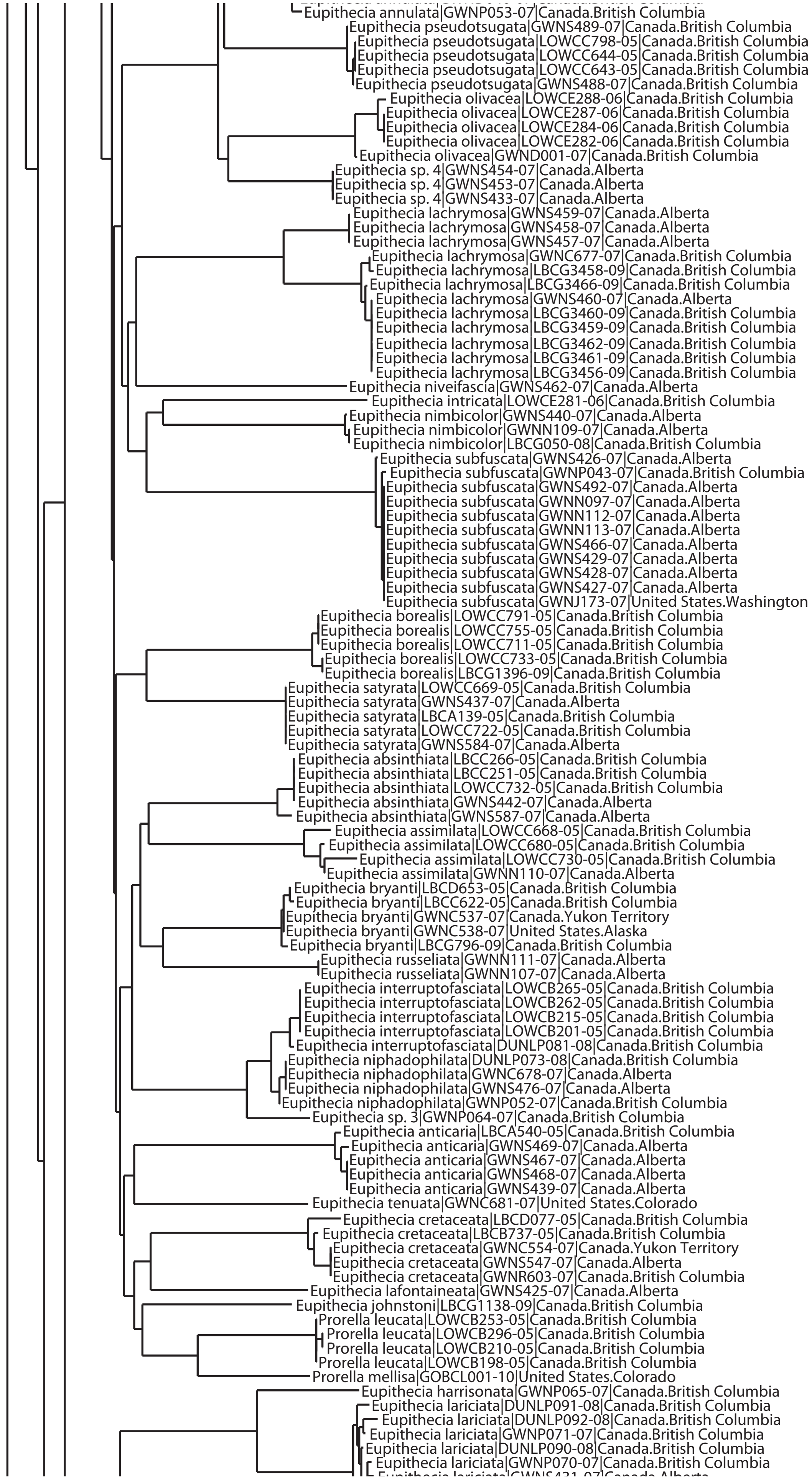

Eupithecia annulata|GWNP053-07|Canada.British Columbia
Eupithecia pseudotsugata|GWNS489-07|Canada.British Columbia
Eupithecia pseudotsugata|LOWCC798-05|Canada.British Columbia
Eupithecia pseudotsugata|LOWCC644-05|Canada.British Columbia
Eupithecia pseudotsugata|LOWCC643-05|Canada.British Columbia
Eupithecia pseudotsugata|GWNS488-07|Canada.British Columbia
Eupithecia olivacea|LOWCE288-06|Canada.British Columbia
Eupithecia olivacea|LOWCE287-06|Canada.British Columbia
Eupithecia olivacea|LOWCE284-06|Canada.British Columbia
Eupithecia olivacea|LOWCE282-06|Canada.British Columbia
Eupithecia olivacea|GWND001-07|Canada.British Columbia
Eupithecia sp. 4|GWNS454-07|Canada.Alberta
Eupithecia sp. 4|GWNS453-07|Canada.Alberta
Eupithecia sp. 4|GWNS433-07|Canada.Alberta
Eupithecia lachrymosa|GWNS459-07|Canada.Alberta
Eupithecia lachrymosa|GWNS458-07|Canada.Alberta
Eupithecia lachrymosa|GWNS457-07|Canada.Alberta
Eupithecia lachrymosa|GWNC677-07|Canada.British Columbia
Eupithecia lachrymosa|LBCG3458-09|Canada.British Columbia
Eupithecia lachrymosa|LBCG3466-09|Canada.British Columbia
Eupithecia lachrymosa|GWNS460-07|Canada.Alberta
Eupithecia lachrymosa|LBCG3460-09|Canada.British Columbia
Eupithecia lachrymosa|LBCG3459-09|Canada.British Columbia
Eupithecia lachrymosa|LBCG3462-09|Canada.British Columbia
Eupithecia lachrymosa|LBCG3461-09|Canada.British Columbia
Eupithecia lachrymosa|LBCG3456-09|Canada.British Columbia
Eupithecia niveifascia|GWNS462-07|Canada.Alberta
Eupithecia intricata|LOWCE281-06|Canada.British Columbia
Eupithecia nimbicolor|GWNS440-07|Canada.Alberta
Eupithecia nimbicolor|GWNN109-07|Canada.Alberta
Eupithecia nimbicolor|LBCG050-08|Canada.British Columbia
Eupithecia subfuscata|GWNS426-07|Canada.Alberta
Eupithecia subfuscata|GWNP043-07|Canada.British Columbia
Eupithecia subfuscata|GWNS492-07|Canada.Alberta
Eupithecia subfuscata|GWNN097-07|Canada.Alberta
Eupithecia subfuscata|GWNN112-07|Canada.Alberta
Eupithecia subfuscata|GWNN113-07|Canada.Alberta
Eupithecia subfuscata|GWNS466-07|Canada.Alberta
Eupithecia subfuscata|GWNS429-07|Canada.Alberta
Eupithecia subfuscata|GWNS428-07|Canada.Alberta
Eupithecia subfuscata|GWNS427-07|Canada.Alberta
Eupithecia subfuscata|GWNJ173-07|United States.Washington
Eupithecia borealis|LOWCC791-05|Canada.British Columbia
Eupithecia borealis|LOWCC755-05|Canada.British Columbia
Eupithecia borealis|LOWCC711-05|Canada.British Columbia
Eupithecia borealis|LOWCC733-05|Canada.British Columbia
Eupithecia borealis|LBCG1396-09|Canada.British Columbia
Eupithecia satyrata|LOWCC669-05|Canada.British Columbia
Eupithecia satyrata|GWNS437-07|Canada.Alberta
Eupithecia satyrata|LBCA139-05|Canada.British Columbia
Eupithecia satyrata|LOWCC722-05|Canada.British Columbia
Eupithecia satyrata|GWNS584-07|Canada.Alberta
Eupithecia absinthiata|LBCC266-05|Canada.British Columbia
Eupithecia absinthiata|LBCC251-05|Canada.British Columbia
Eupithecia absinthiata|LOWCC732-05|Canada.British Columbia
Eupithecia absinthiata|GWNS442-07|Canada.Alberta
Eupithecia absinthiata|GWNS587-07|Canada.Alberta
Eupithecia assimilata|LOWCC668-05|Canada.British Columbia
Eupithecia assimilata|LOWCC680-05|Canada.British Columbia
Eupithecia assimilata|LOWCC730-05|Canada.British Columbia
Eupithecia assimilata|GWNN110-07|Canada.Alberta
Eupithecia bryanti|LBCD653-05|Canada.British Columbia
Eupithecia bryanti|LBCC622-05|Canada.British Columbia
Eupithecia bryanti|GWNC537-07|Canada.Yukon Territory
Eupithecia bryanti|GWNC538-07|United States.Alaska
Eupithecia bryanti|LBCG796-09|Canada.British Columbia
Eupithecia russeliata|GWNN111-07|Canada.Alberta
Eupithecia russeliata|GWNN107-07|Canada.Alberta
Eupithecia interruptofasciata|LOWCB265-05|Canada.British Columbia
Eupithecia interruptofasciata|LOWCB262-05|Canada.British Columbia
Eupithecia interruptofasciata|LOWCB215-05|Canada.British Columbia
Eupithecia interruptofasciata|LOWCB201-05|Canada.British Columbia
Eupithecia interruptofasciata|DUNLP081-08|Canada.British Columbia
Eupithecia niphadophilata|DUNLP073-08|Canada.British Columbia
Eupithecia niphadophilata|GWNC678-07|Canada.Alberta
Eupithecia niphadophilata|GWNS476-07|Canada.Alberta
Eupithecia niphadophilata|GWNP052-07|Canada.British Columbia
Eupithecia sp. 3|GWNP064-07|Canada.British Columbia
Eupithecia anticaria|LBCA540-05|Canada.British Columbia
Eupithecia anticaria|GWNS469-07|Canada.Alberta
Eupithecia anticaria|GWNS467-07|Canada.Alberta
Eupithecia anticaria|GWNS468-07|Canada.Alberta
Eupithecia anticaria|GWNS439-07|Canada.Alberta
Eupithecia tenuata|GWNC681-07|United States.Colorado
Eupithecia cretaceata|LBCD077-05|Canada.British Columbia
Eupithecia cretaceata|LBCB737-05|Canada.British Columbia
Eupithecia cretaceata|GWNC554-07|Canada.Yukon Territory
Eupithecia cretaceata|GWNS547-07|Canada.Alberta
Eupithecia cretaceata|GWNR603-07|Canada.British Columbia
Eupithecia lafontaineata|GWNS425-07|Canada.Alberta
Eupithecia johnstoni|LBCG1138-09|Canada.British Columbia
Prorella leucata|LOWCB253-05|Canada.British Columbia
Prorella leucata|LOWCB296-05|Canada.British Columbia
Prorella leucata|LOWCB210-05|Canada.British Columbia
Prorella leucata|LOWCB198-05|Canada.British Columbia
Prorella mellisa|GOBCL001-10|United States.Colorado
Eupithecia harrisonata|GWNP065-07|Canada.British Columbia
Eupithecia lariciata|DUNLP091-08|Canada.British Columbia
Eupithecia lariciata|DUNLP092-08|Canada.British Columbia
Eupithecia lariciata|GWNP071-07|Canada.British Columbia
Eupithecia lariciata|DUNLP090-08|Canada.British Columbia
Eupithecia lariciata|GWNP070-07|Canada.British Columbia

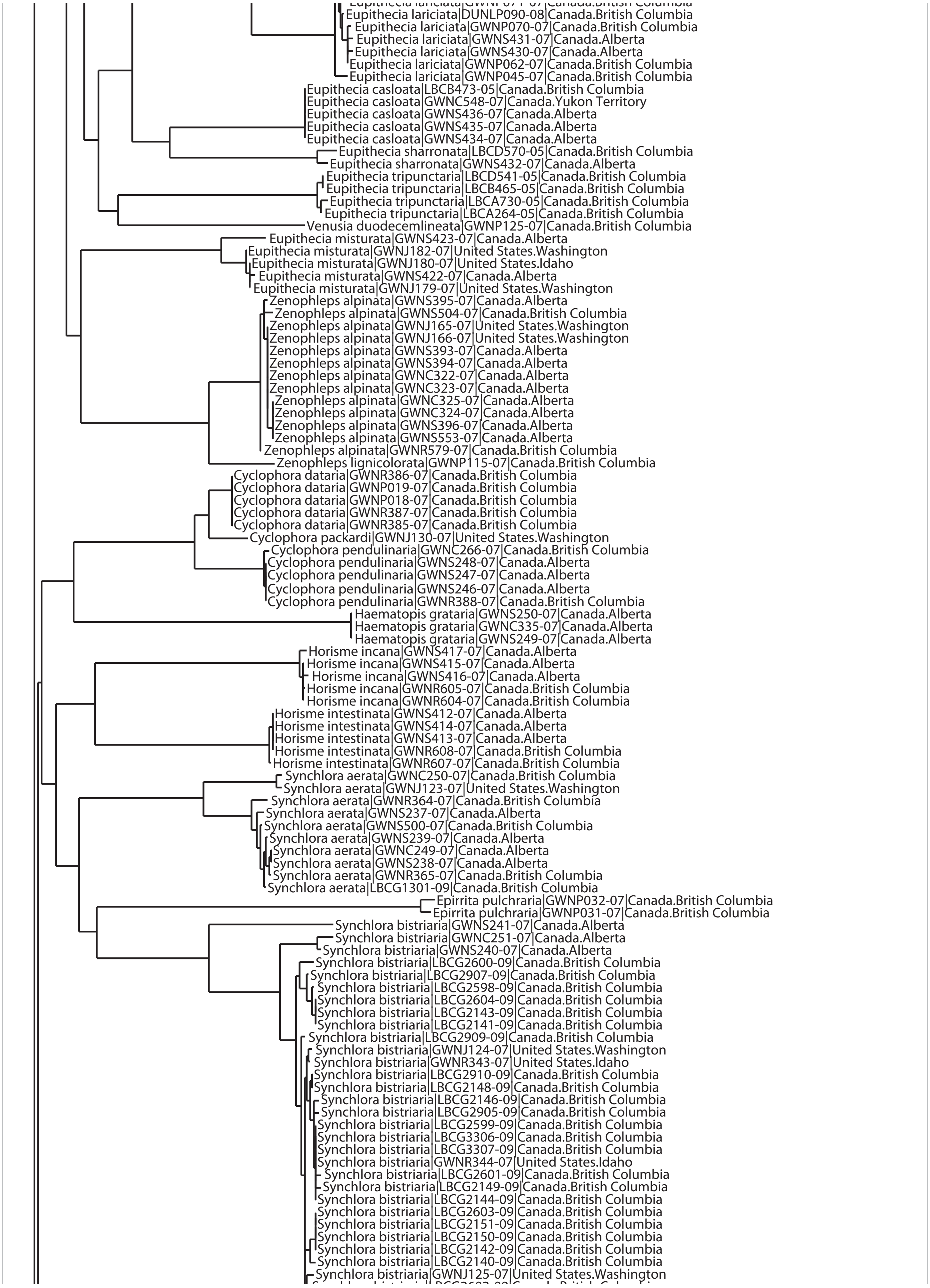
Eupithecia lariciata|GWNP071-07|Canada.British Columbia
Eupithecia lariciata|DUNLP090-08|Canada.British Columbia
Eupithecia lariciata|GWNP070-07|Canada.British Columbia
Eupithecia lariciata|GWNS431-07|Canada.Alberta
Eupithecia lariciata|GWNS430-07|Canada.Alberta
Eupithecia lariciata|GWNP062-07|Canada.British Columbia
Eupithecia lariciata|GWNP045-07|Canada.British Columbia
Eupithecia casloata|LBCB473-05|Canada.British Columbia
Eupithecia casloata|GWNC548-07|Canada.Yukon Territory
Eupithecia casloata|GWNS436-07|Canada.Alberta
Eupithecia casloata|GWNS435-07|Canada.Alberta
Eupithecia casloata|GWNS434-07|Canada.Alberta
Eupithecia sharronata|LBCD570-05|Canada.British Columbia
Eupithecia sharronata|GWNS432-07|Canada.Alberta
Eupithecia tripunctaria|LBCD541-05|Canada.British Columbia
Eupithecia tripunctaria|LBCB465-05|Canada.British Columbia
Eupithecia tripunctaria|LBCA730-05|Canada.British Columbia
Eupithecia tripunctaria|LBCA264-05|Canada.British Columbia
Venusia duodecemlineata|GWNP125-07|Canada.British Columbia
Eupithecia misturata|GWNS423-07|Canada.Alberta
Eupithecia misturata|GWNJ182-07|United States.Washington
Eupithecia misturata|GWNJ180-07|United States.Idaho
Eupithecia misturata|GWNS422-07|Canada.Alberta
Eupithecia misturata|GWNJ179-07|United States.Washington
Zenophleps alpinata|GWNS395-07|Canada.Alberta
Zenophleps alpinata|GWNS504-07|Canada.British Columbia
Zenophleps alpinata|GWNJ165-07|United States.Washington
Zenophleps alpinata|GWNJ166-07|United States.Washington
Zenophleps alpinata|GWNS393-07|Canada.Alberta
Zenophleps alpinata|GWNS394-07|Canada.Alberta
Zenophleps alpinata|GWNC322-07|Canada.Alberta
Zenophleps alpinata|GWNC323-07|Canada.Alberta
Zenophleps alpinata|GWNC325-07|Canada.Alberta
Zenophleps alpinata|GWNC324-07|Canada.Alberta
Zenophleps alpinata|GWNS396-07|Canada.Alberta
Zenophleps alpinata|GWNS553-07|Canada.Alberta
Zenophleps alpinata|GWNR579-07|Canada.British Columbia
Zenophleps lignicolorata|GWNP115-07|Canada.British Columbia
Cyclophora dataria|GWNR386-07|Canada.British Columbia
Cyclophora dataria|GWNP019-07|Canada.British Columbia
Cyclophora dataria|GWNP018-07|Canada.British Columbia
Cyclophora dataria|GWNR387-07|Canada.British Columbia
Cyclophora dataria|GWNR385-07|Canada.British Columbia
Cyclophora packardi|GWNJ130-07|United States.Washington
Cyclophora pendulinaria|GWNC266-07|Canada.British Columbia
Cyclophora pendulinaria|GWNS248-07|Canada.Alberta
Cyclophora pendulinaria|GWNS247-07|Canada.Alberta
Cyclophora pendulinaria|GWNS246-07|Canada.Alberta
Cyclophora pendulinaria|GWNR388-07|Canada.British Columbia
Haematopis grataria|GWNS250-07|Canada.Alberta
Haematopis grataria|GWNC335-07|Canada.Alberta
Haematopis grataria|GWNS249-07|Canada.Alberta
Horisme incana|GWNS417-07|Canada.Alberta
Horisme incana|GWNS415-07|Canada.Alberta
Horisme incana|GWNS416-07|Canada.Alberta
Horisme incana|GWNR605-07|Canada.British Columbia
Horisme incana|GWNR604-07|Canada.British Columbia
Horisme intestinata|GWNS412-07|Canada.Alberta
Horisme intestinata|GWNS414-07|Canada.Alberta
Horisme intestinata|GWNS413-07|Canada.Alberta
Horisme intestinata|GWNR608-07|Canada.British Columbia
Horisme intestinata|GWNR607-07|Canada.British Columbia
Synchlora aerata|GWNC250-07|Canada.British Columbia
Synchlora aerata|GWNJ123-07|United States.Washington
Synchlora aerata|GWNR364-07|Canada.British Columbia
Synchlora aerata|GWNS237-07|Canada.Alberta
Synchlora aerata|GWNS500-07|Canada.British Columbia
Synchlora aerata|GWNS239-07|Canada.Alberta
Synchlora aerata|GWNC249-07|Canada.Alberta
Synchlora aerata|GWNS238-07|Canada.Alberta
Synchlora aerata|GWNR365-07|Canada.British Columbia
Synchlora aerata|LBCG1301-09|Canada.British Columbia
Epirrita pulchraria|GWNP032-07|Canada.British Columbia
Epirrita pulchraria|GWNP031-07|Canada.British Columbia
Synchlora bistriaria|GWNS241-07|Canada.Alberta
Synchlora bistriaria|GWNC251-07|Canada.Alberta
Synchlora bistriaria|GWNS240-07|Canada.Alberta
Synchlora bistriaria|LBCG2600-09|Canada.British Columbia
Synchlora bistriaria|LBCG2907-09|Canada.British Columbia
Synchlora bistriaria|LBCG2598-09|Canada.British Columbia
Synchlora bistriaria|LBCG2604-09|Canada.British Columbia
Synchlora bistriaria|LBCG2143-09|Canada.British Columbia
Synchlora bistriaria|LBCG2141-09|Canada.British Columbia
Synchlora bistriaria|LBCG2909-09|Canada.British Columbia
Synchlora bistriaria|GWNJ124-07|United States.Washington
Synchlora bistriaria|GWNR343-07|United States.Idaho
Synchlora bistriaria|LBCG2910-09|Canada.British Columbia
Synchlora bistriaria|LBCG2148-09|Canada.British Columbia
Synchlora bistriaria|LBCG2146-09|Canada.British Columbia
Synchlora bistriaria|LBCG2905-09|Canada.British Columbia
Synchlora bistriaria|LBCG2599-09|Canada.British Columbia
Synchlora bistriaria|LBCG3306-09|Canada.British Columbia
Synchlora bistriaria|LBCG3307-09|Canada.British Columbia
Synchlora bistriaria|GWNR344-07|United States.Idaho
Synchlora bistriaria|LBCG2601-09|Canada.British Columbia
Synchlora bistriaria|LBCG2149-09|Canada.British Columbia
Synchlora bistriaria|LBCG2144-09|Canada.British Columbia
Synchlora bistriaria|LBCG2603-09|Canada.British Columbia
Synchlora bistriaria|LBCG2151-09|Canada.British Columbia
Synchlora bistriaria|LBCG2150-09|Canada.British Columbia
Synchlora bistriaria|LBCG2142-09|Canada.British Columbia
Synchlora bistriaria|LBCG2140-09|Canada.British Columbia
Synchlora bistriaria|GWNJ125-07|United States.Washington

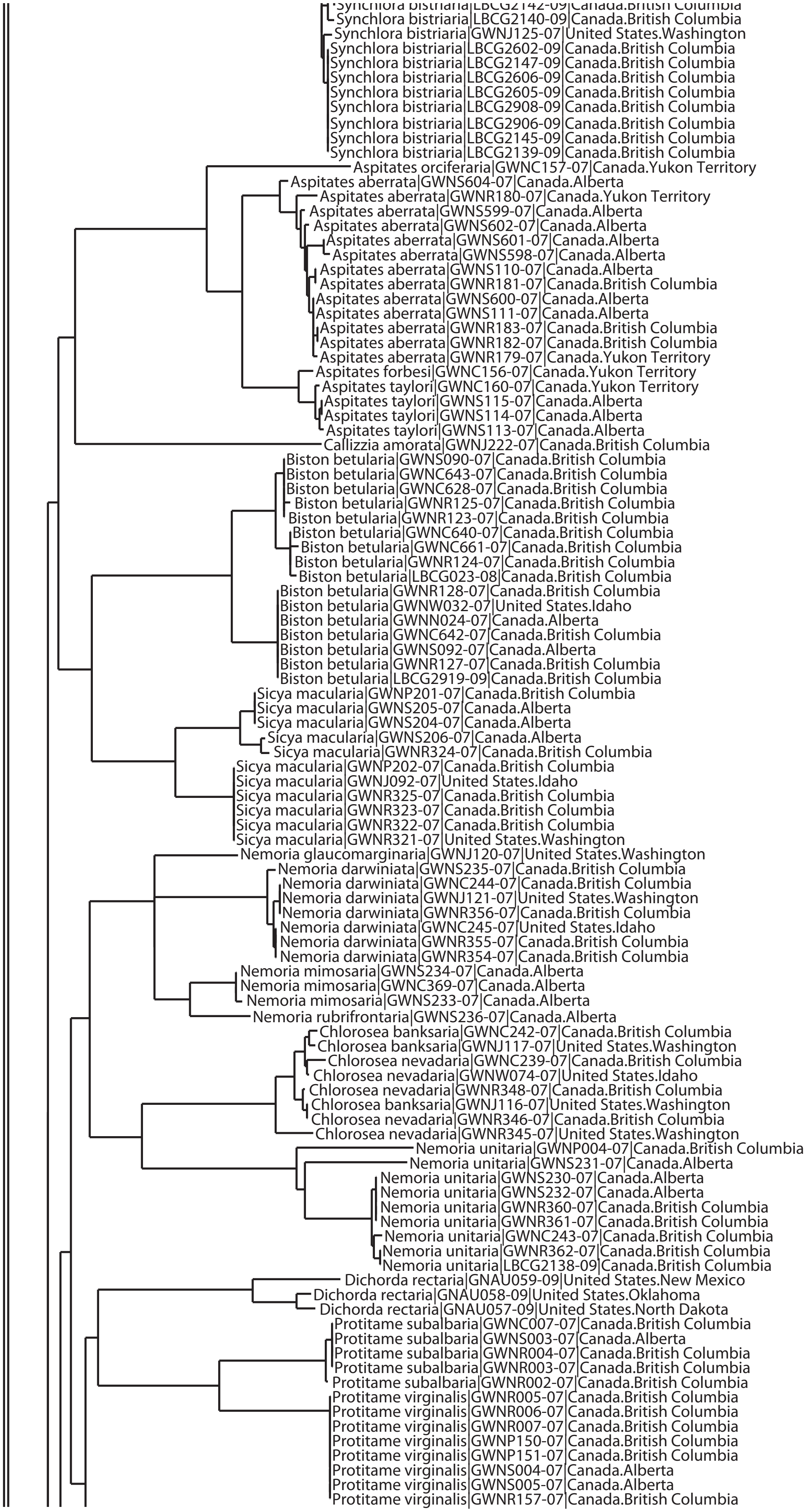
Synchlora bistriaria|LBCG2142-09|Canada.British Columbia
Synchlora bistriaria|LBCG2140-09|Canada.British Columbia
Synchlora bistriaria|GWNJ125-07|United States.Washington
Synchlora bistriaria|LBCG2602-09|Canada.British Columbia
Synchlora bistriaria|LBCG2147-09|Canada.British Columbia
Synchlora bistriaria|LBCG2606-09|Canada.British Columbia
Synchlora bistriaria|LBCG2605-09|Canada.British Columbia
Synchlora bistriaria|LBCG2908-09|Canada.British Columbia
Synchlora bistriaria|LBCG2906-09|Canada.British Columbia
Synchlora bistriaria|LBCG2145-09|Canada.British Columbia
Synchlora bistriaria|LBCG2139-09|Canada.British Columbia
Aspitates orciferaria|GWNC157-07|Canada.Yukon Territory
Aspitates aberrata|GWNS604-07|Canada.Alberta
Aspitates aberrata|GWNR180-07|Canada.Yukon Territory
Aspitates aberrata|GWNS599-07|Canada.Alberta
Aspitates aberrata|GWNS602-07|Canada.Alberta
Aspitates aberrata|GWNS601-07|Canada.Alberta
Aspitates aberrata|GWNS598-07|Canada.Alberta
Aspitates aberrata|GWNS110-07|Canada.Alberta
Aspitates aberrata|GWNR181-07|Canada.British Columbia
Aspitates aberrata|GWNS600-07|Canada.Alberta
Aspitates aberrata|GWNS111-07|Canada.Alberta
Aspitates aberrata|GWNR183-07|Canada.British Columbia
Aspitates aberrata|GWNR182-07|Canada.British Columbia
Aspitates aberrata|GWNR179-07|Canada.Yukon Territory
Aspitates forbesi|GWNC156-07|Canada.Yukon Territory
Aspitates taylori|GWNC160-07|Canada.Yukon Territory
Aspitates taylori|GWNS115-07|Canada.Alberta
Aspitates taylori|GWNS114-07|Canada.Alberta
Aspitates taylori|GWNS113-07|Canada.Alberta
Callizzia amorata|GWNJ222-07|Canada.British Columbia
Biston betularia|GWNS090-07|Canada.British Columbia
Biston betularia|GWNC643-07|Canada.British Columbia
Biston betularia|GWNC628-07|Canada.British Columbia
Biston betularia|GWNR125-07|Canada.British Columbia
Biston betularia|GWNR123-07|Canada.British Columbia
Biston betularia|GWNC640-07|Canada.British Columbia
Biston betularia|GWNC661-07|Canada.British Columbia
Biston betularia|GWNR124-07|Canada.British Columbia
Biston betularia|LBCG023-08|Canada.British Columbia
Biston betularia|GWNR128-07|Canada.British Columbia
Biston betularia|GWNW032-07|United States.Idaho
Biston betularia|GWNN024-07|Canada.Alberta
Biston betularia|GWNC642-07|Canada.British Columbia
Biston betularia|GWNS092-07|Canada.Alberta
Biston betularia|GWNR127-07|Canada.British Columbia
Biston betularia|LBCG2919-09|Canada.British Columbia
Sicya macularia|GWNP201-07|Canada.British Columbia
Sicya macularia|GWNS205-07|Canada.Alberta
Sicya macularia|GWNS204-07|Canada.Alberta
Sicya macularia|GWNS206-07|Canada.Alberta
Sicya macularia|GWNR324-07|Canada.British Columbia
Sicya macularia|GWNP202-07|Canada.British Columbia
Sicya macularia|GWNJ092-07|United States.Idaho
Sicya macularia|GWNR325-07|Canada.British Columbia
Sicya macularia|GWNR323-07|Canada.British Columbia
Sicya macularia|GWNR322-07|Canada.British Columbia
Sicya macularia|GWNR321-07|United States.Washington
Nemoria glaucomarginaria|GWNJ120-07|United States.Washington
Nemoria darwiniata|GWNS235-07|Canada.British Columbia
Nemoria darwiniata|GWNC244-07|Canada.British Columbia
Nemoria darwiniata|GWNJ121-07|United States.Washington
Nemoria darwiniata|GWNR356-07|Canada.British Columbia
Nemoria darwiniata|GWNC245-07|United States.Idaho
Nemoria darwiniata|GWNR355-07|Canada.British Columbia
Nemoria darwiniata|GWNR354-07|Canada.British Columbia
Nemoria mimosaria|GWNS234-07|Canada.Alberta
Nemoria mimosaria|GWNC369-07|Canada.Alberta
Nemoria mimosaria|GWNS233-07|Canada.Alberta
Nemoria rubrifrontaria|GWNS236-07|Canada.Alberta
Chlorosea banksaria|GWNC242-07|Canada.British Columbia
Chlorosea banksaria|GWNJ117-07|United States.Washington
Chlorosea nevadaria|GWNC239-07|Canada.British Columbia
Chlorosea nevadaria|GWNW074-07|United States.Idaho
Chlorosea nevadaria|GWNR348-07|Canada.British Columbia
Chlorosea banksaria|GWNJ116-07|United States.Washington
Chlorosea nevadaria|GWNR346-07|Canada.British Columbia
Chlorosea nevadaria|GWNR345-07|United States.Washington
Nemoria unitaria|GWNP004-07|Canada.British Columbia
Nemoria unitaria|GWNS231-07|Canada.Alberta
Nemoria unitaria|GWNS230-07|Canada.Alberta
Nemoria unitaria|GWNS232-07|Canada.Alberta
Nemoria unitaria|GWNR360-07|Canada.British Columbia
Nemoria unitaria|GWNR361-07|Canada.British Columbia
Nemoria unitaria|GWNC243-07|Canada.British Columbia
Nemoria unitaria|GWNR362-07|Canada.British Columbia
Nemoria unitaria|LBCG2138-09|Canada.British Columbia
Dichorda rectaria|GNAU059-09|United States.New Mexico
Dichorda rectaria|GNAU058-09|United States.Oklahoma
Dichorda rectaria|GNAU057-09|United States.North Dakota
Protitame subalbaria|GWNC007-07|Canada.British Columbia
Protitame subalbaria|GWNS003-07|Canada.Alberta
Protitame subalbaria|GWNR004-07|Canada.British Columbia
Protitame subalbaria|GWNR003-07|Canada.British Columbia
Protitame subalbaria|GWNR002-07|Canada.British Columbia
Protitame virginalis|GWNR005-07|Canada.British Columbia
Protitame virginalis|GWNR006-07|Canada.British Columbia
Protitame virginalis|GWNR007-07|Canada.British Columbia
Protitame virginalis|GWNP150-07|Canada.British Columbia
Protitame virginalis|GWNP151-07|Canada.British Columbia
Protitame virginalis|GWNS004-07|Canada.Alberta
Protitame virginalis|GWNS005-07|Canada.Alberta
Protitame virginalis|GWNR157-07|Canada.British Columbia

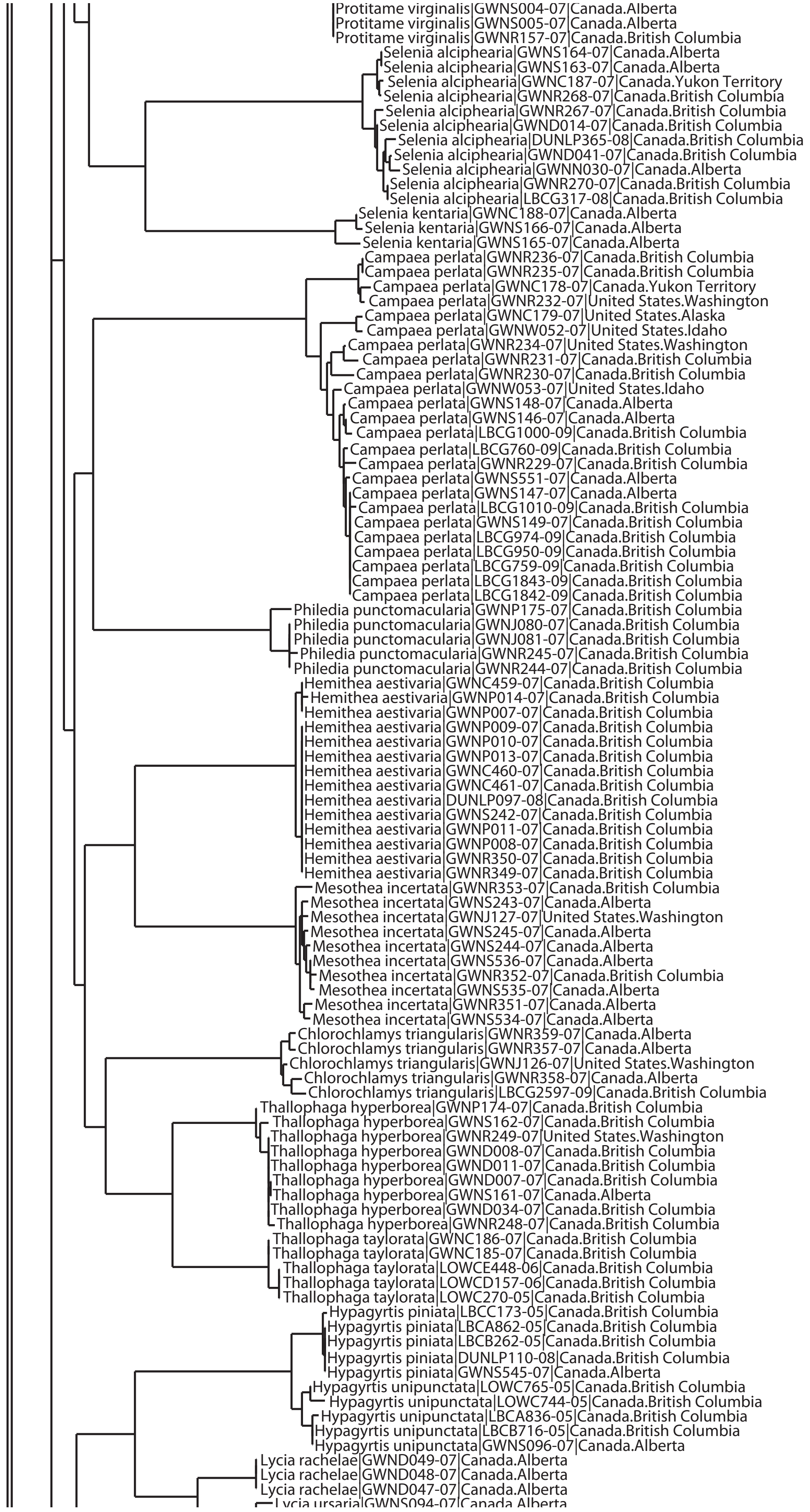
Protitame virginalis|GWNS004-07|Canada.Alberta
Protitame virginalis|GWNS005-07|Canada.Alberta
Protitame virginalis|GWNR157-07|Canada.British Columbia
Selenia alciphearia|GWNS164-07|Canada.Alberta
Selenia alciphearia|GWNS163-07|Canada.Alberta
Selenia alciphearia|GWNC187-07|Canada.Yukon Territory
Selenia alciphearia|GWNR268-07|Canada.British Columbia
Selenia alciphearia|GWNR267-07|Canada.British Columbia
Selenia alciphearia|GWND014-07|Canada.British Columbia
Selenia alciphearia|DUNLP365-08|Canada.British Columbia
Selenia alciphearia|GWND041-07|Canada.British Columbia
Selenia alciphearia|GWNN030-07|Canada.Alberta
Selenia alciphearia|GWNR270-07|Canada.British Columbia
Selenia alciphearia|LBCG317-08|Canada.British Columbia
Selenia kentaria|GWNC188-07|Canada.Alberta
Selenia kentaria|GWNS166-07|Canada.Alberta
Selenia kentaria|GWNS165-07|Canada.Alberta
Campaea perlata|GWNR236-07|Canada.British Columbia
Campaea perlata|GWNR235-07|Canada.British Columbia
Campaea perlata|GWNC178-07|Canada.Yukon Territory
Campaea perlata|GWNR232-07|United States.Washington
Campaea perlata|GWNC179-07|United States.Alaska
Campaea perlata|GWNW052-07|United States.Idaho
Campaea perlata|GWNR234-07|United States.Washington
Campaea perlata|GWNR231-07|Canada.British Columbia
Campaea perlata|GWNR230-07|Canada.British Columbia
Campaea perlata|GWNW053-07|United States.Idaho
Campaea perlata|GWNS148-07|Canada.Alberta
Campaea perlata|GWNS146-07|Canada.Alberta
Campaea perlata|LBCG1000-09|Canada.British Columbia
Campaea perlata|LBCG760-09|Canada.British Columbia
Campaea perlata|GWNR229-07|Canada.British Columbia
Campaea perlata|GWNS551-07|Canada.Alberta
Campaea perlata|GWNS147-07|Canada.Alberta
Campaea perlata|LBCG1010-09|Canada.British Columbia
Campaea perlata|GWNS149-07|Canada.British Columbia
Campaea perlata|LBCG974-09|Canada.British Columbia
Campaea perlata|LBCG950-09|Canada.British Columbia
Campaea perlata|LBCG759-09|Canada.British Columbia
Campaea perlata|LBCG1843-09|Canada.British Columbia
Campaea perlata|LBCG1842-09|Canada.British Columbia
Philedia punctomacularia|GWNP175-07|Canada.British Columbia
Philedia punctomacularia|GWNJ080-07|Canada.British Columbia
Philedia punctomacularia|GWNJ081-07|Canada.British Columbia
Philedia punctomacularia|GWNR245-07|Canada.British Columbia
Philedia punctomacularia|GWNR244-07|Canada.British Columbia
Hemithea aestivaria|GWNC459-07|Canada.British Columbia
Hemithea aestivaria|GWNP014-07|Canada.British Columbia
Hemithea aestivaria|GWNP007-07|Canada.British Columbia
Hemithea aestivaria|GWNP009-07|Canada.British Columbia
Hemithea aestivaria|GWNP010-07|Canada.British Columbia
Hemithea aestivaria|GWNP013-07|Canada.British Columbia
Hemithea aestivaria|GWNC460-07|Canada.British Columbia
Hemithea aestivaria|GWNC461-07|Canada.British Columbia
Hemithea aestivaria|DUNLP097-08|Canada.British Columbia
Hemithea aestivaria|GWNS242-07|Canada.British Columbia
Hemithea aestivaria|GWNP011-07|Canada.British Columbia
Hemithea aestivaria|GWNP008-07|Canada.British Columbia
Hemithea aestivaria|GWNR350-07|Canada.British Columbia
Hemithea aestivaria|GWNR349-07|Canada.British Columbia
Mesothea incertata|GWNR353-07|Canada.British Columbia
Mesothea incertata|GWNS243-07|Canada.Alberta
Mesothea incertata|GWNJ127-07|United States.Washington
Mesothea incertata|GWNS245-07|Canada.Alberta
Mesothea incertata|GWNS244-07|Canada.Alberta
Mesothea incertata|GWNS536-07|Canada.Alberta
Mesothea incertata|GWNR352-07|Canada.British Columbia
Mesothea incertata|GWNS535-07|Canada.Alberta
Mesothea incertata|GWNR351-07|Canada.Alberta
Mesothea incertata|GWNS534-07|Canada.Alberta
Chlorochlamys triangularis|GWNR359-07|Canada.Alberta
Chlorochlamys triangularis|GWNR357-07|Canada.Alberta
Chlorochlamys triangularis|GWNJ126-07|United States.Washington
Chlorochlamys triangularis|GWNR358-07|Canada.Alberta
Chlorochlamys triangularis|LBCG2597-09|Canada.British Columbia
Thallophaga hyperborea|GWNP174-07|Canada.British Columbia
Thallophaga hyperborea|GWNS162-07|Canada.British Columbia
Thallophaga hyperborea|GWNR249-07|United States.Washington
Thallophaga hyperborea|GWND008-07|Canada.British Columbia
Thallophaga hyperborea|GWND011-07|Canada.British Columbia
Thallophaga hyperborea|GWND007-07|Canada.British Columbia
Thallophaga hyperborea|GWNS161-07|Canada.Alberta
Thallophaga hyperborea|GWND034-07|Canada.British Columbia
Thallophaga hyperborea|GWNR248-07|Canada.British Columbia
Thallophaga taylorata|GWNC186-07|Canada.British Columbia
Thallophaga taylorata|GWNC185-07|Canada.British Columbia
Thallophaga taylorata|LOWCE448-06|Canada.British Columbia
Thallophaga taylorata|LOWCD157-06|Canada.British Columbia
Thallophaga taylorata|LOWC270-05|Canada.British Columbia
Hypagyrtis piniata|LBCC173-05|Canada.British Columbia
Hypagyrtis piniata|LBCA862-05|Canada.British Columbia
Hypagyrtis piniata|LBCB262-05|Canada.British Columbia
Hypagyrtis piniata|DUNLP110-08|Canada.British Columbia
Hypagyrtis piniata|GWNS545-07|Canada.Alberta
Hypagyrtis unipunctata|LOWC765-05|Canada.British Columbia
Hypagyrtis unipunctata|LOWC744-05|Canada.British Columbia
Hypagyrtis unipunctata|LBCA836-05|Canada.British Columbia
Hypagyrtis unipunctata|LBCB716-05|Canada.British Columbia
Hypagyrtis unipunctata|GWNS096-07|Canada.Alberta
Lycia rachelae|GWND049-07|Canada.Alberta
Lycia rachelae|GWND048-07|Canada.Alberta
Lycia rachelae|GWND047-07|Canada.Alberta
Lycia ursaria|GWNS094-07|Canada.Alberta

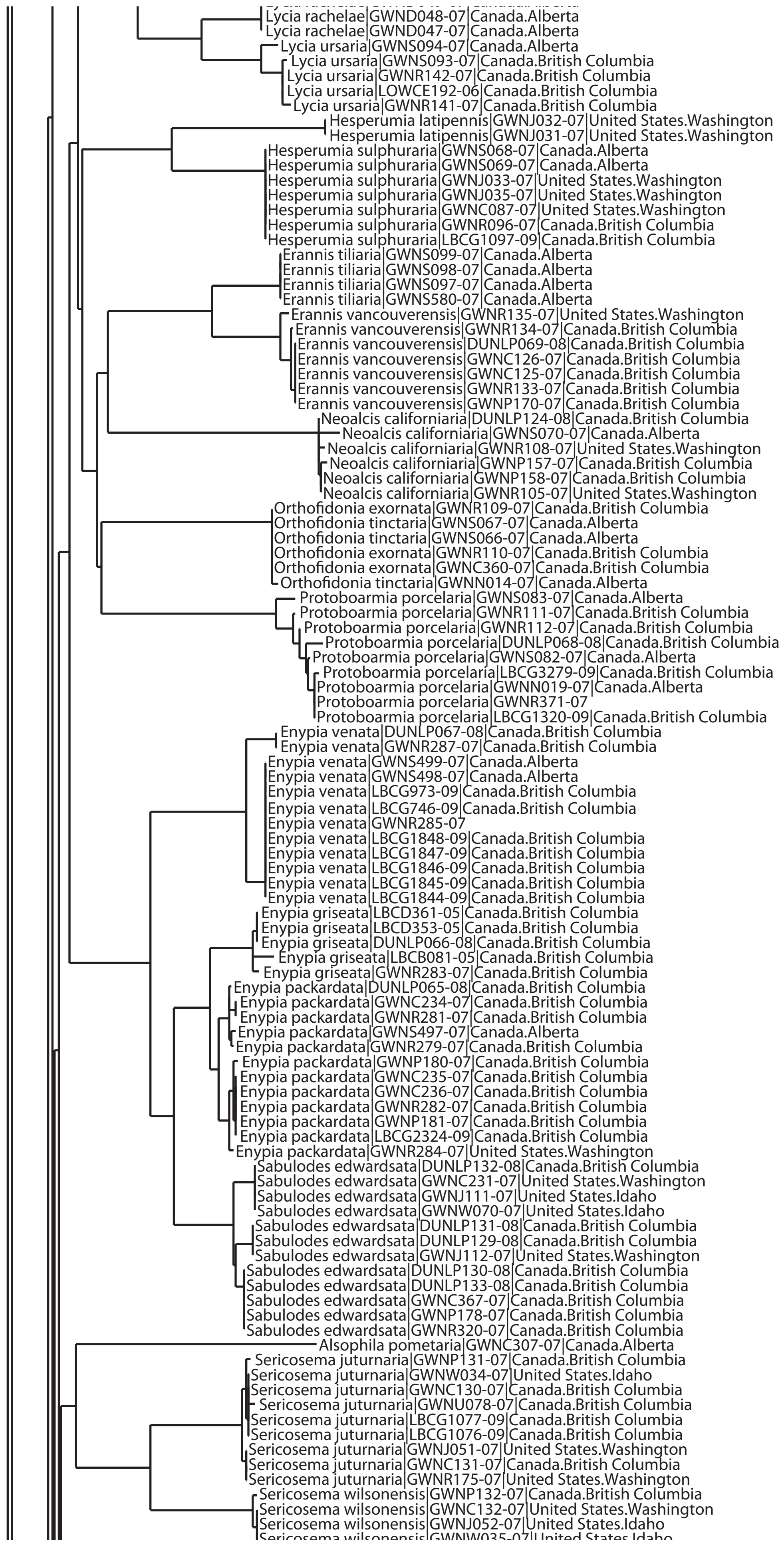
Lycia rachelae|GWND048-07|Canada.Alberta
Lycia rachelae|GWND047-07|Canada.Alberta
Lycia ursaria|GWNS094-07|Canada.Alberta
Lycia ursaria|GWNS093-07|Canada.British Columbia
Lycia ursaria|GWNR142-07|Canada.British Columbia
Lycia ursaria|LOWCE192-06|Canada.British Columbia
Lycia ursaria|GWNR141-07|Canada.British Columbia
Hesperumia latipennis|GWNJ032-07|United States.Washington
Hesperumia latipennis|GWNJ031-07|United States.Washington
Hesperumia sulphuraria|GWNS068-07|Canada.Alberta
Hesperumia sulphuraria|GWNS069-07|Canada.Alberta
Hesperumia sulphuraria|GWNJ033-07|United States.Washington
Hesperumia sulphuraria|GWNJ035-07|United States.Washington
Hesperumia sulphuraria|GWNC087-07|United States.Washington
Hesperumia sulphuraria|GWNR096-07|Canada.British Columbia
Hesperumia sulphuraria|LBCG1097-09|Canada.British Columbia
Erannis tiliaria|GWNS099-07|Canada.Alberta
Erannis tiliaria|GWNS098-07|Canada.Alberta
Erannis tiliaria|GWNS097-07|Canada.Alberta
Erannis tiliaria|GWNS580-07|Canada.Alberta
Erannis vancouverensis|GWNR135-07|United States.Washington
Erannis vancouverensis|GWNR134-07|Canada.British Columbia
Erannis vancouverensis|DUNLP069-08|Canada.British Columbia
Erannis vancouverensis|GWNC126-07|Canada.British Columbia
Erannis vancouverensis|GWNC125-07|Canada.British Columbia
Erannis vancouverensis|GWNR133-07|Canada.British Columbia
Erannis vancouverensis|GWNP170-07|Canada.British Columbia
Neoalcis californiaria|DUNLP124-08|Canada.British Columbia
Neoalcis californiaria|GWNS070-07|Canada.Alberta
Neoalcis californiaria|GWNR108-07|United States.Washington
Neoalcis californiaria|GWNP157-07|Canada.British Columbia
Neoalcis californiaria|GWNP158-07|Canada.British Columbia
Neoalcis californiaria|GWNR105-07|United States.Washington
Orthofidonia exornata|GWNR109-07|Canada.British Columbia
Orthofidonia tinctaria|GWNS067-07|Canada.Alberta
Orthofidonia tinctaria|GWNS066-07|Canada.Alberta
Orthofidonia exornata|GWNR110-07|Canada.British Columbia
Orthofidonia exornata|GWNC360-07|Canada.British Columbia
Orthofidonia tinctaria|GWNN014-07|Canada.Alberta
Protoboarmia porcelaria|GWNS083-07|Canada.Alberta
Protoboarmia porcelaria|GWNR111-07|Canada.British Columbia
Protoboarmia porcelaria|GWNR112-07|Canada.British Columbia
Protoboarmia porcelaria|DUNLP068-08|Canada.British Columbia
Protoboarmia porcelaria|GWNS082-07|Canada.Alberta
Protoboarmia porcelaria|LBCG3279-09|Canada.British Columbia
Protoboarmia porcelaria|GWNN019-07|Canada.Alberta
Protoboarmia porcelaria|GWNR371-07
Protoboarmia porcelaria|LBCG1320-09|Canada.British Columbia
Enypia venata|DUNLP067-08|Canada.British Columbia
Enypia venata|GWNR287-07|Canada.British Columbia
Enypia venata|GWNS499-07|Canada.Alberta
Enypia venata|GWNS498-07|Canada.Alberta
Enypia venata|LBCG973-09|Canada.British Columbia
Enypia venata|LBCG746-09|Canada.British Columbia
Enypia venata|GWNR285-07
Enypia venata|LBCG1848-09|Canada.British Columbia
Enypia venata|LBCG1847-09|Canada.British Columbia
Enypia venata|LBCG1846-09|Canada.British Columbia
Enypia venata|LBCG1845-09|Canada.British Columbia
Enypia venata|LBCG1844-09|Canada.British Columbia
Enypia griseata|LBCD361-05|Canada.British Columbia
Enypia griseata|LBCD353-05|Canada.British Columbia
Enypia griseata|DUNLP066-08|Canada.British Columbia
Enypia griseata|LBCB081-05|Canada.British Columbia
Enypia griseata|GWNR283-07|Canada.British Columbia
Enypia packardata|DUNLP065-08|Canada.British Columbia
Enypia packardata|GWNC234-07|Canada.British Columbia
Enypia packardata|GWNR281-07|Canada.British Columbia
Enypia packardata|GWNS497-07|Canada.Alberta
Enypia packardata|GWNR279-07|Canada.British Columbia
Enypia packardata|GWNP180-07|Canada.British Columbia
Enypia packardata|GWNC235-07|Canada.British Columbia
Enypia packardata|GWNC236-07|Canada.British Columbia
Enypia packardata|GWNR282-07|Canada.British Columbia
Enypia packardata|GWNP181-07|Canada.British Columbia
Enypia packardata|LBCG2324-09|Canada.British Columbia
Enypia packardata|GWNR284-07|United States.Washington
Sabulodes edwardsata|DUNLP132-08|Canada.British Columbia
Sabulodes edwardsata|GWNC231-07|United States.Washington
Sabulodes edwardsata|GWNJ111-07|United States.Idaho
Sabulodes edwardsata|GWNW070-07|United States.Idaho
Sabulodes edwardsata|DUNLP131-08|Canada.British Columbia
Sabulodes edwardsata|DUNLP129-08|Canada.British Columbia
Sabulodes edwardsata|GWNJ112-07|United States.Washington
Sabulodes edwardsata|DUNLP130-08|Canada.British Columbia
Sabulodes edwardsata|DUNLP133-08|Canada.British Columbia
Sabulodes edwardsata|GWNC367-07|Canada.British Columbia
Sabulodes edwardsata|GWNP178-07|Canada.British Columbia
Sabulodes edwardsata|GWNR320-07|Canada.British Columbia
Alsophila pometaria|GWNC307-07|Canada.Alberta
Sericosema juturnaria|GWNP131-07|Canada.British Columbia
Sericosema juturnaria|GWNW034-07|United States.Idaho
Sericosema juturnaria|GWNC130-07|Canada.British Columbia
Sericosema juturnaria|GWNU078-07|Canada.British Columbia
Sericosema juturnaria|LBCG1077-09|Canada.British Columbia
Sericosema juturnaria|LBCG1076-09|Canada.British Columbia
Sericosema juturnaria|GWNJ051-07|United States.Washington
Sericosema juturnaria|GWNC131-07|Canada.British Columbia
Sericosema juturnaria|GWNR175-07|United States.Washington
Sericosema wilsonensis|GWNP132-07|Canada.British Columbia
Sericosema wilsonensis|GWNC132-07|United States.Washington
Sericosema wilsonensis|GWNJ052-07|United States.Idaho

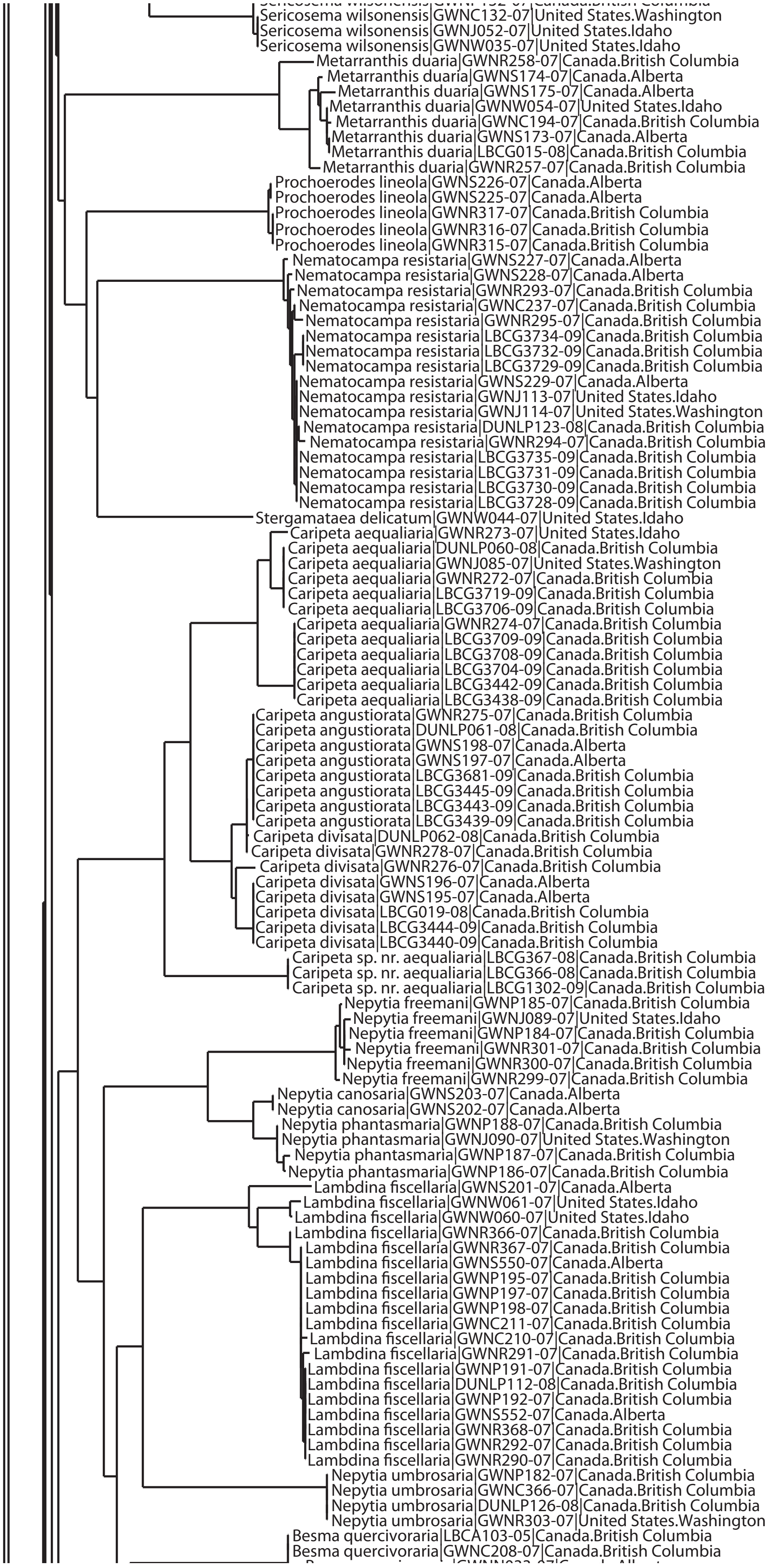

Sericosema wilsonensis|GWNC132-07|United States.Washington
Sericosema wilsonensis|GWNJ052-07|United States.Idaho
Sericosema wilsonensis|GWNW035-07|United States.Idaho
Metarranthis duaria|GWNR258-07|Canada.British Columbia
Metarranthis duaria|GWNS174-07|Canada.Alberta
Metarranthis duaria|GWNS175-07|Canada.Alberta
Metarranthis duaria|GWNW054-07|United States.Idaho
Metarranthis duaria|GWNC194-07|Canada.British Columbia
Metarranthis duaria|GWNS173-07|Canada.Alberta
Metarranthis duaria|LBCG015-08|Canada.British Columbia
Metarranthis duaria|GWNR257-07|Canada.British Columbia
Prochoerodes lineola|GWNS226-07|Canada.Alberta
Prochoerodes lineola|GWNS225-07|Canada.Alberta
Prochoerodes lineola|GWNR317-07|Canada.British Columbia
Prochoerodes lineola|GWNR316-07|Canada.British Columbia
Prochoerodes lineola|GWNR315-07|Canada.British Columbia
Nematocampa resistaria|GWNS227-07|Canada.Alberta
Nematocampa resistaria|GWNS228-07|Canada.Alberta
Nematocampa resistaria|GWNR293-07|Canada.British Columbia
Nematocampa resistaria|GWNC237-07|Canada.British Columbia
Nematocampa resistaria|GWNR295-07|Canada.British Columbia
Nematocampa resistaria|LBCG3734-09|Canada.British Columbia
Nematocampa resistaria|LBCG3732-09|Canada.British Columbia
Nematocampa resistaria|LBCG3729-09|Canada.British Columbia
Nematocampa resistaria|GWNS229-07|Canada.Alberta
Nematocampa resistaria|GWNJ113-07|United States.Idaho
Nematocampa resistaria|GWNJ114-07|United States.Washington
Nematocampa resistaria|DUNLP123-08|Canada.British Columbia
Nematocampa resistaria|GWNR294-07|Canada.British Columbia
Nematocampa resistaria|LBCG3735-09|Canada.British Columbia
Nematocampa resistaria|LBCG3731-09|Canada.British Columbia
Nematocampa resistaria|LBCG3730-09|Canada.British Columbia
Nematocampa resistaria|LBCG3728-09|Canada.British Columbia
Stergamataea delicatum|GWNW044-07|United States.Idaho
Caripeta aequaliaria|GWNR273-07|United States.Idaho
Caripeta aequaliaria|DUNLP060-08|Canada.British Columbia
Caripeta aequaliaria|GWNJ085-07|United States.Washington
Caripeta aequaliaria|GWNR272-07|Canada.British Columbia
Caripeta aequaliaria|LBCG3719-09|Canada.British Columbia
Caripeta aequaliaria|LBCG3706-09|Canada.British Columbia
Caripeta aequaliaria|GWNR274-07|Canada.British Columbia
Caripeta aequaliaria|LBCG3709-09|Canada.British Columbia
Caripeta aequaliaria|LBCG3708-09|Canada.British Columbia
Caripeta aequaliaria|LBCG3704-09|Canada.British Columbia
Caripeta aequaliaria|LBCG3442-09|Canada.British Columbia
Caripeta aequaliaria|LBCG3438-09|Canada.British Columbia
Caripeta angustiorata|GWNR275-07|Canada.British Columbia
Caripeta angustiorata|DUNLP061-08|Canada.British Columbia
Caripeta angustiorata|GWNS198-07|Canada.Alberta
Caripeta angustiorata|GWNS197-07|Canada.Alberta
Caripeta angustiorata|LBCG3681-09|Canada.British Columbia
Caripeta angustiorata|LBCG3445-09|Canada.British Columbia
Caripeta angustiorata|LBCG3443-09|Canada.British Columbia
Caripeta angustiorata|LBCG3439-09|Canada.British Columbia
Caripeta divisata|DUNLP062-08|Canada.British Columbia
Caripeta divisata|GWNR278-07|Canada.British Columbia
Caripeta divisata|GWNR276-07|Canada.British Columbia
Caripeta divisata|GWNS196-07|Canada.Alberta
Caripeta divisata|GWNS195-07|Canada.Alberta
Caripeta divisata|LBCG019-08|Canada.British Columbia
Caripeta divisata|LBCG3444-09|Canada.British Columbia
Caripeta divisata|LBCG3440-09|Canada.British Columbia
Caripeta sp. nr. aequaliaria|LBCG367-08|Canada.British Columbia
Caripeta sp. nr. aequaliaria|LBCG366-08|Canada.British Columbia
Caripeta sp. nr. aequaliaria|LBCG1302-09|Canada.British Columbia
Nepytia freemani|GWNP185-07|Canada.British Columbia
Nepytia freemani|GWNJ089-07|United States.Idaho
Nepytia freemani|GWNP184-07|Canada.British Columbia
Nepytia freemani|GWNR301-07|Canada.British Columbia
Nepytia freemani|GWNR300-07|Canada.British Columbia
Nepytia freemani|GWNR299-07|Canada.British Columbia
Nepytia canosaria|GWNS203-07|Canada.Alberta
Nepytia canosaria|GWNS202-07|Canada.Alberta
Nepytia phantasmaria|GWNP188-07|Canada.British Columbia
Nepytia phantasmaria|GWNJ090-07|United States.Washington
Nepytia phantasmaria|GWNP187-07|Canada.British Columbia
Nepytia phantasmaria|GWNP186-07|Canada.British Columbia
Lambdina fiscellaria|GWNS201-07|Canada.Alberta
Lambdina fiscellaria|GWNW061-07|United States.Idaho
Lambdina fiscellaria|GWNW060-07|United States.Idaho
Lambdina fiscellaria|GWNR366-07|Canada.British Columbia
Lambdina fiscellaria|GWNR367-07|Canada.British Columbia
Lambdina fiscellaria|GWNS550-07|Canada.Alberta
Lambdina fiscellaria|GWNP195-07|Canada.British Columbia
Lambdina fiscellaria|GWNP197-07|Canada.British Columbia
Lambdina fiscellaria|GWNP198-07|Canada.British Columbia
Lambdina fiscellaria|GWNC211-07|Canada.British Columbia
Lambdina fiscellaria|GWNC210-07|Canada.British Columbia
Lambdina fiscellaria|GWNR291-07|Canada.British Columbia
Lambdina fiscellaria|GWNP191-07|Canada.British Columbia
Lambdina fiscellaria|DUNLP112-08|Canada.British Columbia
Lambdina fiscellaria|GWNP192-07|Canada.British Columbia
Lambdina fiscellaria|GWNS552-07|Canada.Alberta
Lambdina fiscellaria|GWNR368-07|Canada.British Columbia
Lambdina fiscellaria|GWNR292-07|Canada.British Columbia
Lambdina fiscellaria|GWNR290-07|Canada.British Columbia
Nepytia umbrosaria|GWNP182-07|Canada.British Columbia
Nepytia umbrosaria|GWNC366-07|Canada.British Columbia
Nepytia umbrosaria|DUNLP126-08|Canada.British Columbia
Nepytia umbrosaria|GWNR303-07|United States.Washington
Besma quercivoraria|LBCA103-05|Canada.British Columbia
Besma quercivoraria|GWNC208-07|Canada.British Columbia

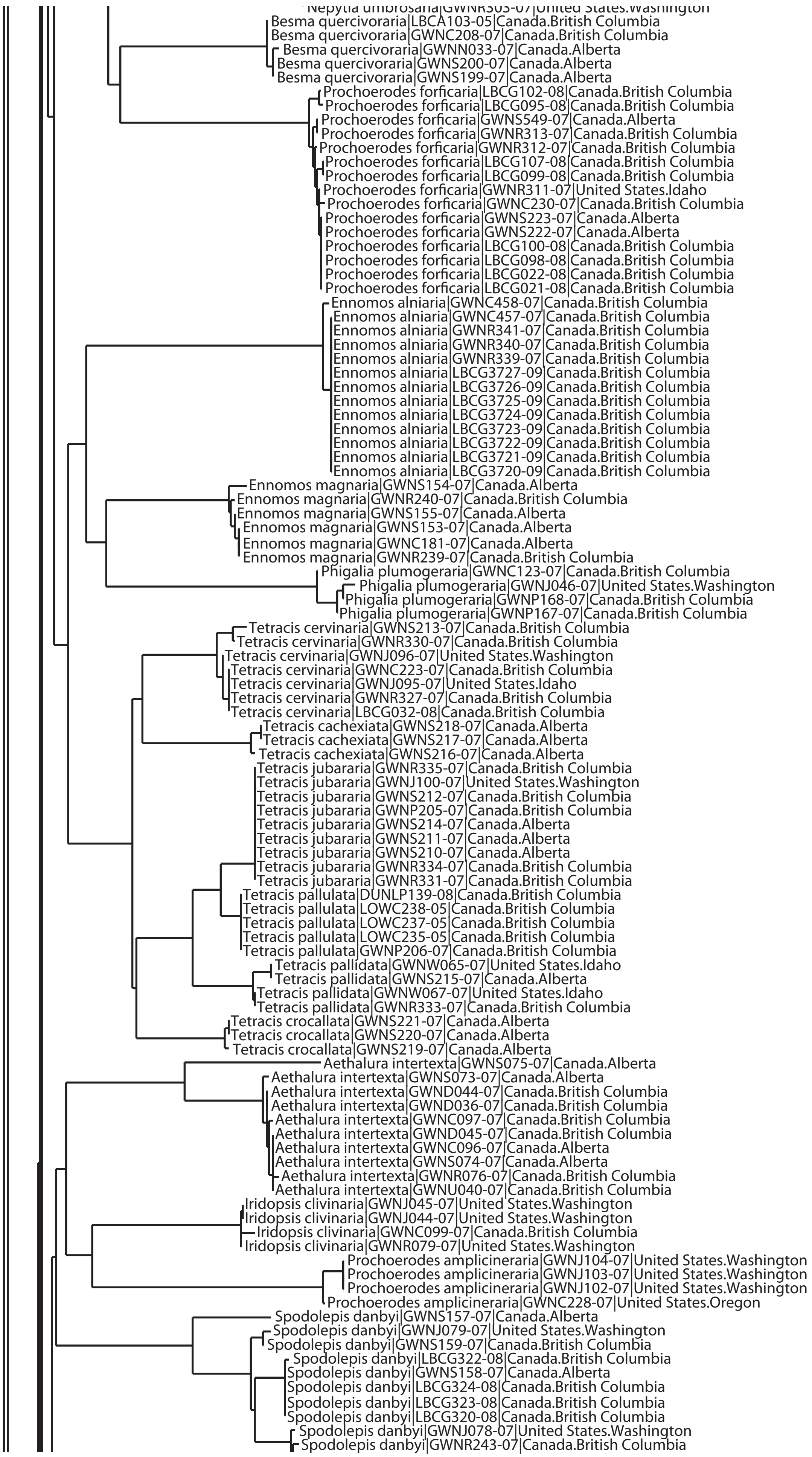
Nepytia umbrosaria|GWNR303-07|United States.Washington
Besma quercivoraria|LBCA103-05|Canada.British Columbia
Besma quercivoraria|GWNC208-07|Canada.British Columbia
Besma quercivoraria|GWNN033-07|Canada.Alberta
Besma quercivoraria|GWNS200-07|Canada.Alberta
Besma quercivoraria|GWNS199-07|Canada.Alberta
Prochoerodes forficaria|LBCG102-08|Canada.British Columbia
Prochoerodes forficaria|LBCG095-08|Canada.British Columbia
Prochoerodes forficaria|GWNS549-07|Canada.Alberta
Prochoerodes forficaria|GWNR313-07|Canada.British Columbia
Prochoerodes forficaria|GWNR312-07|Canada.British Columbia
Prochoerodes forficaria|LBCG107-08|Canada.British Columbia
Prochoerodes forficaria|LBCG099-08|Canada.British Columbia
Prochoerodes forficaria|GWNR311-07|United States.Idaho
Prochoerodes forficaria|GWNC230-07|Canada.British Columbia
Prochoerodes forficaria|GWNS223-07|Canada.Alberta
Prochoerodes forficaria|GWNS222-07|Canada.Alberta
Prochoerodes forficaria|LBCG100-08|Canada.British Columbia
Prochoerodes forficaria|LBCG098-08|Canada.British Columbia
Prochoerodes forficaria|LBCG022-08|Canada.British Columbia
Prochoerodes forficaria|LBCG021-08|Canada.British Columbia
Ennomos alniaria|GWNC458-07|Canada.British Columbia
Ennomos alniaria|GWNC457-07|Canada.British Columbia
Ennomos alniaria|GWNR341-07|Canada.British Columbia
Ennomos alniaria|GWNR340-07|Canada.British Columbia
Ennomos alniaria|GWNR339-07|Canada.British Columbia
Ennomos alniaria|LBCG3727-09|Canada.British Columbia
Ennomos alniaria|LBCG3726-09|Canada.British Columbia
Ennomos alniaria|LBCG3725-09|Canada.British Columbia
Ennomos alniaria|LBCG3724-09|Canada.British Columbia
Ennomos alniaria|LBCG3723-09|Canada.British Columbia
Ennomos alniaria|LBCG3722-09|Canada.British Columbia
Ennomos alniaria|LBCG3721-09|Canada.British Columbia
Ennomos alniaria|LBCG3720-09|Canada.British Columbia
Ennomos magnaria|GWNS154-07|Canada.Alberta
Ennomos magnaria|GWNR240-07|Canada.British Columbia
Ennomos magnaria|GWNS155-07|Canada.Alberta
Ennomos magnaria|GWNS153-07|Canada.Alberta
Ennomos magnaria|GWNC181-07|Canada.Alberta
Ennomos magnaria|GWNR239-07|Canada.British Columbia
Phigalia plumogeraria|GWNC123-07|Canada.British Columbia
Phigalia plumogeraria|GWNJ046-07|United States.Washington
Phigalia plumogeraria|GWNP168-07|Canada.British Columbia
Phigalia plumogeraria|GWNP167-07|Canada.British Columbia
Tetracis cervinaria|GWNS213-07|Canada.British Columbia
Tetracis cervinaria|GWNR330-07|Canada.British Columbia
Tetracis cervinaria|GWNJ096-07|United States.Washington
Tetracis cervinaria|GWNC223-07|Canada.British Columbia
Tetracis cervinaria|GWNJ095-07|United States.Idaho
Tetracis cervinaria|GWNR327-07|Canada.British Columbia
Tetracis cervinaria|LBCG032-08|Canada.British Columbia
Tetracis cachexiata|GWNS218-07|Canada.Alberta
Tetracis cachexiata|GWNS217-07|Canada.Alberta
Tetracis cachexiata|GWNS216-07|Canada.Alberta
Tetracis jubararia|GWNR335-07|Canada.British Columbia
Tetracis jubararia|GWNJ100-07|United States.Washington
Tetracis jubararia|GWNS212-07|Canada.British Columbia
Tetracis jubararia|GWNP205-07|Canada.British Columbia
Tetracis jubararia|GWNS214-07|Canada.Alberta
Tetracis jubararia|GWNS211-07|Canada.Alberta
Tetracis jubararia|GWNS210-07|Canada.Alberta
Tetracis jubararia|GWNR334-07|Canada.British Columbia
Tetracis jubararia|GWNR331-07|Canada.British Columbia
Tetracis pallulata|DUNLP139-08|Canada.British Columbia
Tetracis pallulata|LOWC238-05|Canada.British Columbia
Tetracis pallulata|LOWC237-05|Canada.British Columbia
Tetracis pallulata|LOWC235-05|Canada.British Columbia
Tetracis pallulata|GWNP206-07|Canada.British Columbia
Tetracis pallidata|GWNW065-07|United States.Idaho
Tetracis pallidata|GWNS215-07|Canada.Alberta
Tetracis pallidata|GWNW067-07|United States.Idaho
Tetracis pallidata|GWNR333-07|Canada.British Columbia
Tetracis crocallata|GWNS221-07|Canada.Alberta
Tetracis crocallata|GWNS220-07|Canada.Alberta
Tetracis crocallata|GWNS219-07|Canada.Alberta
Aethalura intertexta|GWNS075-07|Canada.Alberta
Aethalura intertexta|GWNS073-07|Canada.Alberta
Aethalura intertexta|GWND044-07|Canada.British Columbia
Aethalura intertexta|GWND036-07|Canada.British Columbia
Aethalura intertexta|GWNC097-07|Canada.British Columbia
Aethalura intertexta|GWND045-07|Canada.British Columbia
Aethalura intertexta|GWNC096-07|Canada.Alberta
Aethalura intertexta|GWNS074-07|Canada.Alberta
Aethalura intertexta|GWNR076-07|Canada.British Columbia
Aethalura intertexta|GWNU040-07|Canada.British Columbia
Iridopsis clivinaria|GWNJ045-07|United States.Washington
Iridopsis clivinaria|GWNJ044-07|United States.Washington
Iridopsis clivinaria|GWNC099-07|Canada.British Columbia
Iridopsis clivinaria|GWNR079-07|United States.Washington
Prochoerodes amplicineraria|GWNJ104-07|United States.Washington
Prochoerodes amplicineraria|GWNJ103-07|United States.Washington
Prochoerodes amplicineraria|GWNJ102-07|United States.Washington
Prochoerodes amplicineraria|GWNC228-07|United States.Oregon
Spodolepis danbyi|GWNS157-07|Canada.Alberta
Spodolepis danbyi|GWNJ079-07|United States.Washington
Spodolepis danbyi|GWNS159-07|Canada.British Columbia
Spodolepis danbyi|LBCG322-08|Canada.British Columbia
Spodolepis danbyi|GWNS158-07|Canada.Alberta
Spodolepis danbyi|LBCG324-08|Canada.British Columbia
Spodolepis danbyi|LBCG323-08|Canada.British Columbia
Spodolepis danbyi|LBCG320-08|Canada.British Columbia
Spodolepis danbyi|GWNJ078-07|United States.Washington
Spodolepis danbyi|GWNR243-07|Canada.British Columbia

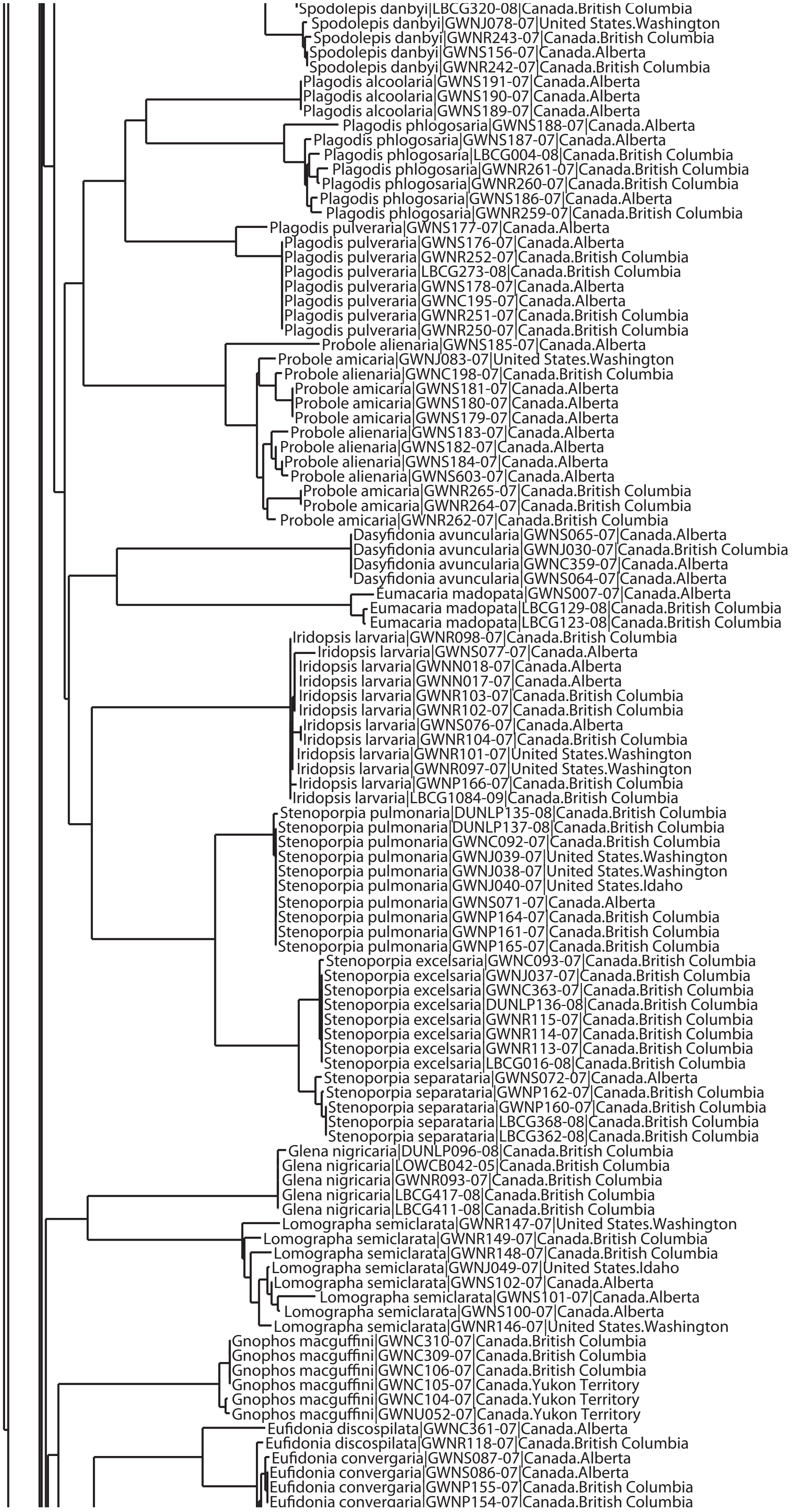
Spodolepis danbyi|LBCG320-08|Canada.British Columbia
Spodolepis danbyi|GWNJ078-07|United States.Washington
Spodolepis danbyi|GWNR243-07|Canada.British Columbia
Spodolepis danbyi|GWNS156-07|Canada.Alberta
Spodolepis danbyi|GWNR242-07|Canada.British Columbia
Plagodis alcoolaria|GWNS191-07|Canada.Alberta
Plagodis alcoolaria|GWNS190-07|Canada.Alberta
Plagodis alcoolaria|GWNS189-07|Canada.Alberta
Plagodis phlogosaria|GWNS188-07|Canada.Alberta
Plagodis phlogosaria|GWNS187-07|Canada.Alberta
Plagodis phlogosaria|LBCG004-08|Canada.British Columbia
Plagodis phlogosaria|GWNR261-07|Canada.British Columbia
Plagodis phlogosaria|GWNR260-07|Canada.British Columbia
Plagodis phlogosaria|GWNS186-07|Canada.Alberta
Plagodis phlogosaria|GWNR259-07|Canada.British Columbia
Plagodis pulveraria|GWNS177-07|Canada.Alberta
Plagodis pulveraria|GWNS176-07|Canada.Alberta
Plagodis pulveraria|GWNR252-07|Canada.British Columbia
Plagodis pulveraria|LBCG273-08|Canada.British Columbia
Plagodis pulveraria|GWNS178-07|Canada.Alberta
Plagodis pulveraria|GWNC195-07|Canada.Alberta
Plagodis pulveraria|GWNR251-07|Canada.British Columbia
Plagodis pulveraria|GWNR250-07|Canada.British Columbia
Probole alienaria|GWNS185-07|Canada.Alberta
Probole amicaria|GWNJ083-07|United States.Washington
Probole alienaria|GWNC198-07|Canada.British Columbia
Probole amicaria|GWNS181-07|Canada.Alberta
Probole amicaria|GWNS180-07|Canada.Alberta
Probole amicaria|GWNS179-07|Canada.Alberta
Probole alienaria|GWNS183-07|Canada.Alberta
Probole alienaria|GWNS182-07|Canada.Alberta
Probole alienaria|GWNS184-07|Canada.Alberta
Probole alienaria|GWNS603-07|Canada.Alberta
Probole amicaria|GWNR265-07|Canada.British Columbia
Probole amicaria|GWNR264-07|Canada.British Columbia
Probole amicaria|GWNR262-07|Canada.British Columbia
Dasyfidonia avuncularia|GWNS065-07|Canada.Alberta
Dasyfidonia avuncularia|GWNJ030-07|Canada.British Columbia
Dasyfidonia avuncularia|GWNC359-07|Canada.Alberta
Dasyfidonia avuncularia|GWNS064-07|Canada.Alberta
Eumacaria madopata|GWNS007-07|Canada.Alberta
Eumacaria madopata|LBCG129-08|Canada.British Columbia
Eumacaria madopata|LBCG123-08|Canada.British Columbia
Iridopsis larvaria|GWNR098-07|Canada.British Columbia
Iridopsis larvaria|GWNS077-07|Canada.Alberta
Iridopsis larvaria|GWNN018-07|Canada.Alberta
Iridopsis larvaria|GWNN017-07|Canada.Alberta
Iridopsis larvaria|GWNR103-07|Canada.British Columbia
Iridopsis larvaria|GWNR102-07|Canada.British Columbia
Iridopsis larvaria|GWNS076-07|Canada.Alberta
Iridopsis larvaria|GWNR104-07|Canada.British Columbia
Iridopsis larvaria|GWNR101-07|United States.Washington
Iridopsis larvaria|GWNR097-07|United States.Washington
Iridopsis larvaria|GWNP166-07|Canada.British Columbia
Iridopsis larvaria|LBCG1084-09|Canada.British Columbia
Stenoporpia pulmonaria|DUNLP135-08|Canada.British Columbia
Stenoporpia pulmonaria|DUNLP137-08|Canada.British Columbia
Stenoporpia pulmonaria|GWNC092-07|Canada.British Columbia
Stenoporpia pulmonaria|GWNJ039-07|United States.Washington
Stenoporpia pulmonaria|GWNJ038-07|United States.Washington
Stenoporpia pulmonaria|GWNJ040-07|United States.Idaho
Stenoporpia pulmonaria|GWNS071-07|Canada.Alberta
Stenoporpia pulmonaria|GWNP164-07|Canada.British Columbia
Stenoporpia pulmonaria|GWNP161-07|Canada.British Columbia
Stenoporpia pulmonaria|GWNP165-07|Canada.British Columbia
Stenoporpia excelsaria|GWNC093-07|Canada.British Columbia
Stenoporpia excelsaria|GWNJ037-07|Canada.British Columbia
Stenoporpia excelsaria|GWNC363-07|Canada.British Columbia
Stenoporpia excelsaria|DUNLP136-08|Canada.British Columbia
Stenoporpia excelsaria|GWNR115-07|Canada.British Columbia
Stenoporpia excelsaria|GWNR114-07|Canada.British Columbia
Stenoporpia excelsaria|GWNR113-07|Canada.British Columbia
Stenoporpia excelsaria|LBCG016-08|Canada.British Columbia
Stenoporpia separataria|GWNS072-07|Canada.Alberta
Stenoporpia separataria|GWNP162-07|Canada.British Columbia
Stenoporpia separataria|GWNP160-07|Canada.British Columbia
Stenoporpia separataria|LBCG368-08|Canada.British Columbia
Stenoporpia separataria|LBCG362-08|Canada.British Columbia
Glena nigricaria|DUNLP096-08|Canada.British Columbia
Glena nigricaria|LOWCB042-05|Canada.British Columbia
Glena nigricaria|GWNR093-07|Canada.British Columbia
Glena nigricaria|LBCG417-08|Canada.British Columbia
Glena nigricaria|LBCG411-08|Canada.British Columbia
Lomographa semiclarata|GWNR147-07|United States.Washington
Lomographa semiclarata|GWNR149-07|Canada.British Columbia
Lomographa semiclarata|GWNR148-07|Canada.British Columbia
Lomographa semiclarata|GWNJ049-07|United States.Idaho
Lomographa semiclarata|GWNS102-07|Canada.Alberta
Lomographa semiclarata|GWNS101-07|Canada.Alberta
Lomographa semiclarata|GWNS100-07|Canada.Alberta
Lomographa semiclarata|GWNR146-07|United States.Washington
Gnophos macguffini|GWNC310-07|Canada.British Columbia
Gnophos macguffini|GWNC309-07|Canada.British Columbia
Gnophos macguffini|GWNC106-07|Canada.British Columbia
Gnophos macguffini|GWNC105-07|Canada.Yukon Territory
Gnophos macguffini|GWNC104-07|Canada.Yukon Territory
Gnophos macguffini|GWNU052-07|Canada.Yukon Territory
Eufidonia discospilata|GWNC361-07|Canada.Alberta
Eufidonia discospilata|GWNR118-07|Canada.British Columbia
Eufidonia convergaria|GWNS087-07|Canada.Alberta
Eufidonia convergaria|GWNS086-07|Canada.Alberta
Eufidonia convergaria|GWNP155-07|Canada.British Columbia
Eufidonia convergaria|GWNP154-07|Canada.British Columbia

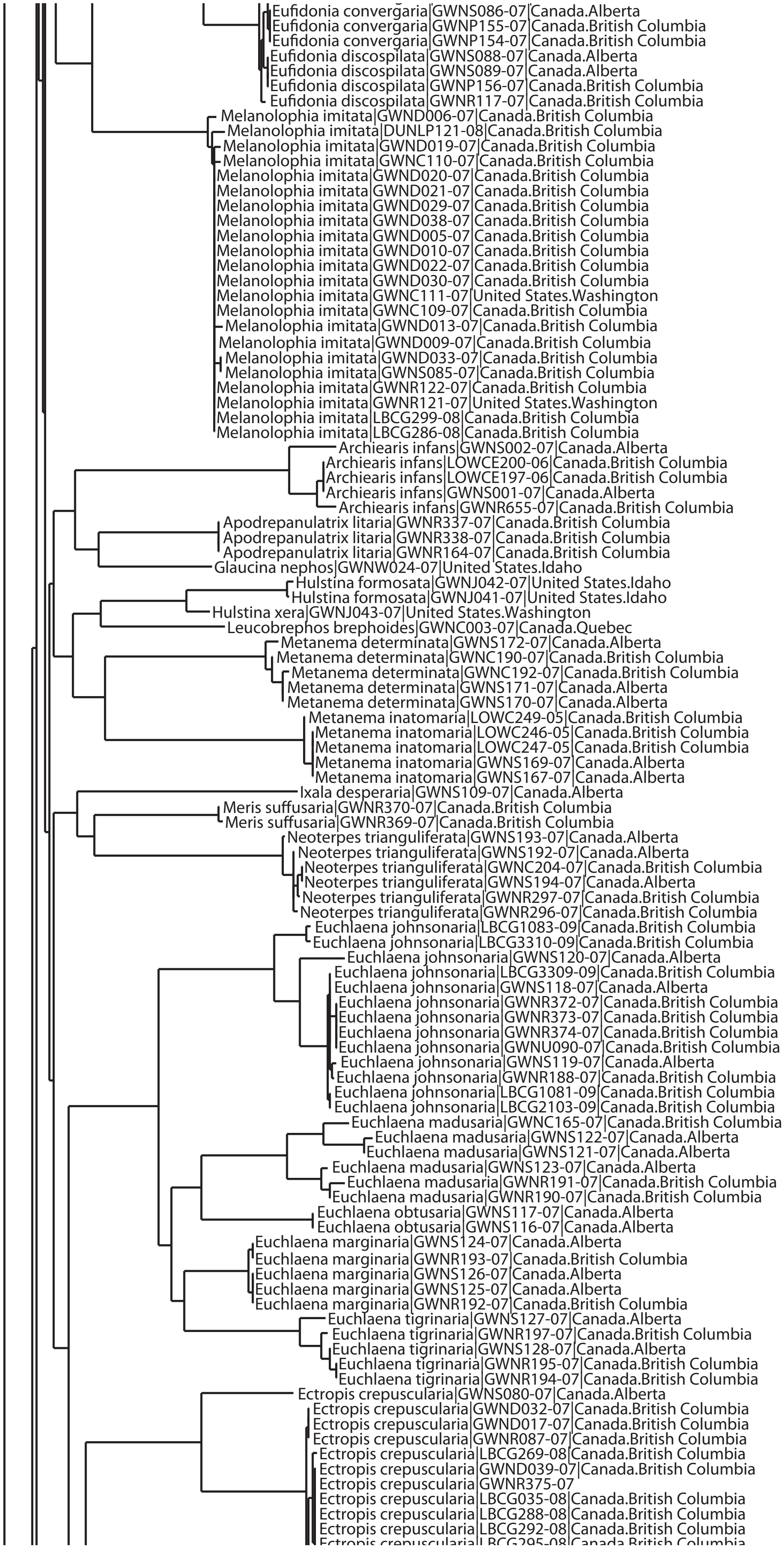
Eufidonia convergaria|GWNS086-07|Canada.Alberta
Eufidonia convergaria|GWNP155-07|Canada.British Columbia
Eufidonia convergaria|GWNP154-07|Canada.British Columbia
Eufidonia discospilata|GWNS088-07|Canada.Alberta
Eufidonia discospilata|GWNS089-07|Canada.Alberta
Eufidonia discospilata|GWNP156-07|Canada.British Columbia
Eufidonia discospilata|GWNR117-07|Canada.British Columbia
Melanolophia imitata|GWND006-07|Canada.British Columbia
Melanolophia imitata|DUNLP121-08|Canada.British Columbia
Melanolophia imitata|GWND019-07|Canada.British Columbia
Melanolophia imitata|GWNC110-07|Canada.British Columbia
Melanolophia imitata|GWND020-07|Canada.British Columbia
Melanolophia imitata|GWND021-07|Canada.British Columbia
Melanolophia imitata|GWND029-07|Canada.British Columbia
Melanolophia imitata|GWND038-07|Canada.British Columbia
Melanolophia imitata|GWND005-07|Canada.British Columbia
Melanolophia imitata|GWND010-07|Canada.British Columbia
Melanolophia imitata|GWND022-07|Canada.British Columbia
Melanolophia imitata|GWND030-07|Canada.British Columbia
Melanolophia imitata|GWNC111-07|United States.Washington
Melanolophia imitata|GWNC109-07|Canada.British Columbia
Melanolophia imitata|GWND013-07|Canada.British Columbia
Melanolophia imitata|GWND009-07|Canada.British Columbia
Melanolophia imitata|GWND033-07|Canada.British Columbia
Melanolophia imitata|GWNS085-07|Canada.British Columbia
Melanolophia imitata|GWNR122-07|Canada.British Columbia
Melanolophia imitata|GWNR121-07|United States.Washington
Melanolophia imitata|LBCG299-08|Canada.British Columbia
Melanolophia imitata|LBCG286-08|Canada.British Columbia
Archiearis infans|GWNS002-07|Canada.Alberta
Archiearis infans|LOWCE200-06|Canada.British Columbia
Archiearis infans|LOWCE197-06|Canada.British Columbia
Archiearis infans|GWNS001-07|Canada.Alberta
Archiearis infans|GWNR655-07|Canada.British Columbia
Apodrepanulatrix litaria|GWNR337-07|Canada.British Columbia
Apodrepanulatrix litaria|GWNR338-07|Canada.British Columbia
Apodrepanulatrix litaria|GWNR164-07|Canada.British Columbia
Glaucina nephos|GWNW024-07|United States.Idaho
Hulstina formosata|GWNJ042-07|United States.Idaho
Hulstina formosata|GWNJ041-07|United States.Idaho
Hulstina xera|GWNJ043-07|United States.Washington
Leucobrephos brephoides|GWNC003-07|Canada.Quebec
Metanema determinata|GWNS172-07|Canada.Alberta
Metanema determinata|GWNC190-07|Canada.British Columbia
Metanema determinata|GWNC192-07|Canada.British Columbia
Metanema determinata|GWNS171-07|Canada.Alberta
Metanema determinata|GWNS170-07|Canada.Alberta
Metanema inatomaria|LOWC249-05|Canada.British Columbia
Metanema inatomaria|LOWC246-05|Canada.British Columbia
Metanema inatomaria|LOWC247-05|Canada.British Columbia
Metanema inatomaria|GWNS169-07|Canada.Alberta
Metanema inatomaria|GWNS167-07|Canada.Alberta
Ixala desperaria|GWNS109-07|Canada.Alberta
Meris suffusaria|GWNR370-07|Canada.British Columbia
Meris suffusaria|GWNR369-07|Canada.British Columbia
Neoterpes trianguliferata|GWNS193-07|Canada.Alberta
Neoterpes trianguliferata|GWNS192-07|Canada.Alberta
Neoterpes trianguliferata|GWNC204-07|Canada.British Columbia
Neoterpes trianguliferata|GWNS194-07|Canada.Alberta
Neoterpes trianguliferata|GWNR297-07|Canada.British Columbia
Neoterpes trianguliferata|GWNR296-07|Canada.British Columbia
Euchlaena johnsonaria|LBCG1083-09|Canada.British Columbia
Euchlaena johnsonaria|LBCG3310-09|Canada.British Columbia
Euchlaena johnsonaria|GWNS120-07|Canada.Alberta
Euchlaena johnsonaria|LBCG3309-09|Canada.British Columbia
Euchlaena johnsonaria|GWNS118-07|Canada.Alberta
Euchlaena johnsonaria|GWNR372-07|Canada.British Columbia
Euchlaena johnsonaria|GWNR373-07|Canada.British Columbia
Euchlaena johnsonaria|GWNR374-07|Canada.British Columbia
Euchlaena johnsonaria|GWNU090-07|Canada.British Columbia
Euchlaena johnsonaria|GWNS119-07|Canada.Alberta
Euchlaena johnsonaria|GWNR188-07|Canada.British Columbia
Euchlaena johnsonaria|LBCG1081-09|Canada.British Columbia
Euchlaena johnsonaria|LBCG2103-09|Canada.British Columbia
Euchlaena madusaria|GWNC165-07|Canada.British Columbia
Euchlaena madusaria|GWNS122-07|Canada.Alberta
Euchlaena madusaria|GWNS121-07|Canada.Alberta
Euchlaena madusaria|GWNS123-07|Canada.Alberta
Euchlaena madusaria|GWNR191-07|Canada.British Columbia
Euchlaena madusaria|GWNR190-07|Canada.British Columbia
Euchlaena obtusaria|GWNS117-07|Canada.Alberta
Euchlaena obtusaria|GWNS116-07|Canada.Alberta
Euchlaena marginaria|GWNS124-07|Canada.Alberta
Euchlaena marginaria|GWNR193-07|Canada.British Columbia
Euchlaena marginaria|GWNS126-07|Canada.Alberta
Euchlaena marginaria|GWNS125-07|Canada.Alberta
Euchlaena marginaria|GWNR192-07|Canada.British Columbia
Euchlaena tigrinaria|GWNS127-07|Canada.Alberta
Euchlaena tigrinaria|GWNR197-07|Canada.British Columbia
Euchlaena tigrinaria|GWNS128-07|Canada.Alberta
Euchlaena tigrinaria|GWNR195-07|Canada.British Columbia
Euchlaena tigrinaria|GWNR194-07|Canada.British Columbia
Ectropis crepuscularia|GWNS080-07|Canada.Alberta
Ectropis crepuscularia|GWND032-07|Canada.British Columbia
Ectropis crepuscularia|GWND017-07|Canada.British Columbia
Ectropis crepuscularia|GWNR087-07|Canada.British Columbia
Ectropis crepuscularia|LBCG269-08|Canada.British Columbia
Ectropis crepuscularia|GWND039-07|Canada.British Columbia
Ectropis crepuscularia|GWNR375-07
Ectropis crepuscularia|LBCG035-08|Canada.British Columbia
Ectropis crepuscularia|LBCG288-08|Canada.British Columbia
Ectropis crepuscularia|LBCG292-08|Canada.British Columbia

Ectropis crepuscularia|[illegible]|Canada.British Columbia
Ectropis crepuscularia|LBCG288-08|Canada.British Columbia
Ectropis crepuscularia|LBCG292-08|Canada.British Columbia
Ectropis crepuscularia|LBCG295-08|Canada.British Columbia
Ectropis crepuscularia|LBCG296-08|Canada.British Columbia
Ectropis crepuscularia|GWND016-07|Canada.British Columbia
Ectropis crepuscularia|GWNC107-07|Canada.British Columbia
Ectropis crepuscularia|LBCG281-08|Canada.British Columbia
Ectropis crepuscularia|LBCG297-08|Canada.British Columbia
Ectropis crepuscularia|GWNS081-07|Canada.Alberta
Ectropis crepuscularia|GWND042-07|Canada.British Columbia
Ectropis crepuscularia|LBCG304-08|Canada.British Columbia
Ectropis crepuscularia|LBCG318-08|Canada.British Columbia
Ectropis crepuscularia|LBCG319-08|Canada.British Columbia
Xanthotype urticaria|GWNS129-07|Canada.Alberta
Xanthotype sospeta|GWNC601-07|Canada.Alberta
Xanthotype sospeta|GWNS132-07|Canada.Alberta
Xanthotype sospeta|GWNS131-07|Canada.Alberta
Xanthotype urticaria|GWNR199-07|Canada.British Columbia
Xanthotype sospeta|GWNS133-07|Canada.Alberta
Xanthotype urticaria|GWNC365-07|Canada.Alberta
Xanthotype urticaria|GWNR198-07|Canada.British Columbia
Drepanulatrix bifilata|GWNW037-07|United States.Idaho
Drepanulatrix quadraria|GWNJ061-07|United States.Washington
Drepanulatrix unicalcararia|GWNC142-07|Canada.British Columbia
Drepanulatrix unicalcararia|LBCG1080-09|Canada.British Columbia
Drepanulatrix unicalcararia|LBCG1082-09|Canada.British Columbia
Drepanulatrix unicalcararia|GWNW042-07|United States.Idaho
Drepanulatrix unicalcararia|GWNJ064-07|United States.Idaho
Drepanulatrix unicalcararia|GWNR171-07|Canada.British Columbia
Cabera borealis|GWNS533-07|Canada.Alberta
Cabera borealis|GWNS532-07|Canada.Alberta
Cabera borealis|GWNS531-07|Canada.Alberta
Cabera borealis|GWNS530-07|Canada.Alberta
Cabera borealis|GWNS529-07|Canada.Alberta
Cabera borealis|GWNS528-07|Canada.Alberta
Cabera erythemaria|GWNS105-07|Canada.Alberta
Cabera erythemaria|GWNR151-07|Canada.British Columbia
Cabera exanthemata|GWNR155-07|Canada.British Columbia
Cabera erythemaria|GWNS104-07|Canada.Alberta
Cabera erythemaria|GWNR152-07|Canada.British Columbia
Cabera erythemaria|GWNR150-07|United States.Washington
Cabera erythemaria|GWNS103-07|Canada.Alberta
Cabera erythemaria|GWNW036-07|United States.Idaho
Cabera exanthemata|GWNR153-07|Canada.British Columbia
Cabera variolaria|LBCA045-05|Canada.British Columbia
Cabera variolaria|GWNS107-07|Canada.Alberta
Cabera variolaria|GWNS106-07|Canada.Alberta
Cabera variolaria|GWNS595-07|Canada.Alberta
Cabera variolaria|GWNR158-07|Canada.British Columbia
Drepanulatrix falcataria|GWNJ057-07|United States.Washington
Drepanulatrix falcataria|LBCG1061-09|Canada.British Columbia
Drepanulatrix falcataria|LBCG1063-09|Canada.British Columbia
Drepanulatrix falcataria|LBCG1060-09|Canada.British Columbia
Drepanulatrix falcataria|LBCG1088-09|Canada.British Columbia
Drepanulatrix falcataria|LBCG1079-09|Canada.British Columbia
Drepanulatrix carnearia|GWNW038-07|United States.Idaho
Drepanulatrix falcataria|GWNC148-07|Canada.British Columbia
Drepanulatrix falcataria|GWNR160-07|Canada.British Columbia
Drepanulatrix carnearia|GWNS108-07|Canada.Alberta
Drepanulatrix falcataria|GWNR159-07|Canada.British Columbia
Drepanulatrix foeminaria|GWNW039-07|United States.Washington
Drepanulatrix foeminaria|GWNC146-07|Canada.British Columbia
Drepanulatrix foeminaria|GWNJ059-07|United States.Washington
Drepanulatrix foeminaria|GWNW041-07|United States.Idaho
Drepanulatrix secundaria|GWNU087-07|Canada.British Columbia
Drepanulatrix secundaria|GWNC149-07|Canada.British Columbia
Drepanulatrix secundaria|GWNJ058-07|United States.Idaho
Drepanulatrix secundaria|GWNR165-07|Canada.British Columbia
Eudrepanulatrix rectifascia|GWNC140-07|Canada.British Columbia
Eudrepanulatrix rectifascia|LBCG1062-09|Canada.British Columbia
Eudrepanulatrix rectifascia|LBCG1086-09|Canada.British Columbia
Eudrepanulatrix rectifascia|LBCG1094-09|Canada.British Columbia
Eudrepanulatrix rectifascia|GWNR172-07|United States.Washington
Gabriola dyari|GWNR222-07|Canada.British Columbia
Gabriola dyari|LBCG2316-09|Canada.British Columbia
Gabriola dyari|GWNW050-07|United States.Idaho
Gabriola dyari|GWNR220-07|Canada.British Columbia
Gabriola dyari|GWNR221-07|Canada.British Columbia
Gabriola dyari|GWNJ074-07|United States.Washington
Gabriola dyari|DUNLP095-08|Canada.British Columbia
Gabriola dyari|GWNW051-07|United States.Idaho
Gabriola dyari|GWNP173-07|Canada.British Columbia
Gabriola dyari|LBCG201-08|Canada.British Columbia
Gabriola dyari|LBCG2317-09|Canada.British Columbia
Gabriola dyari|GWNS152-07|Canada.British Columbia
Gabriola dyari|GWNR219-07|Canada.British Columbia
Gabriola dyari|GWNR218-07|Canada.British Columbia
Anavitrinella addendaria|GNAU170-10|United States.Nevada
Anavitrinella addendaria|GNAU168-10|United States.Nevada
Anavitrinella addendaria|GNAU173-10|United States.Texas
Anavitrinella addendaria|GNAU171-10|United States.Texas
Anavitrinella addendaria|GWNW025-07|United States.Idaho
Anavitrinella pampinaria|DUNLP059-08|Canada.British Columbia
Anavitrinella pampinaria|GWNS078-07|Canada.Alberta
Anavitrinella pampinaria|GWNS079-07|Canada.Alberta
Anavitrinella pampinaria|GWNR082-07|Canada.British Columbia
Anavitrinella pampinaria|GWNR078-07|Canada.British Columbia
Ematurga amitaria|GWNN016-07|Canada.Alberta
Macaria atrimacularia|GNAU182-10|United States.Texas
Macaria colata|GWNJ004-07|United States.Washington
Macaria colata|GWNC041-07|Canada.British Columbia
Macaria colata|GWNJ001-07|United States.Washington
Macaria colata|GWNR018-07|Canada.British Columbia

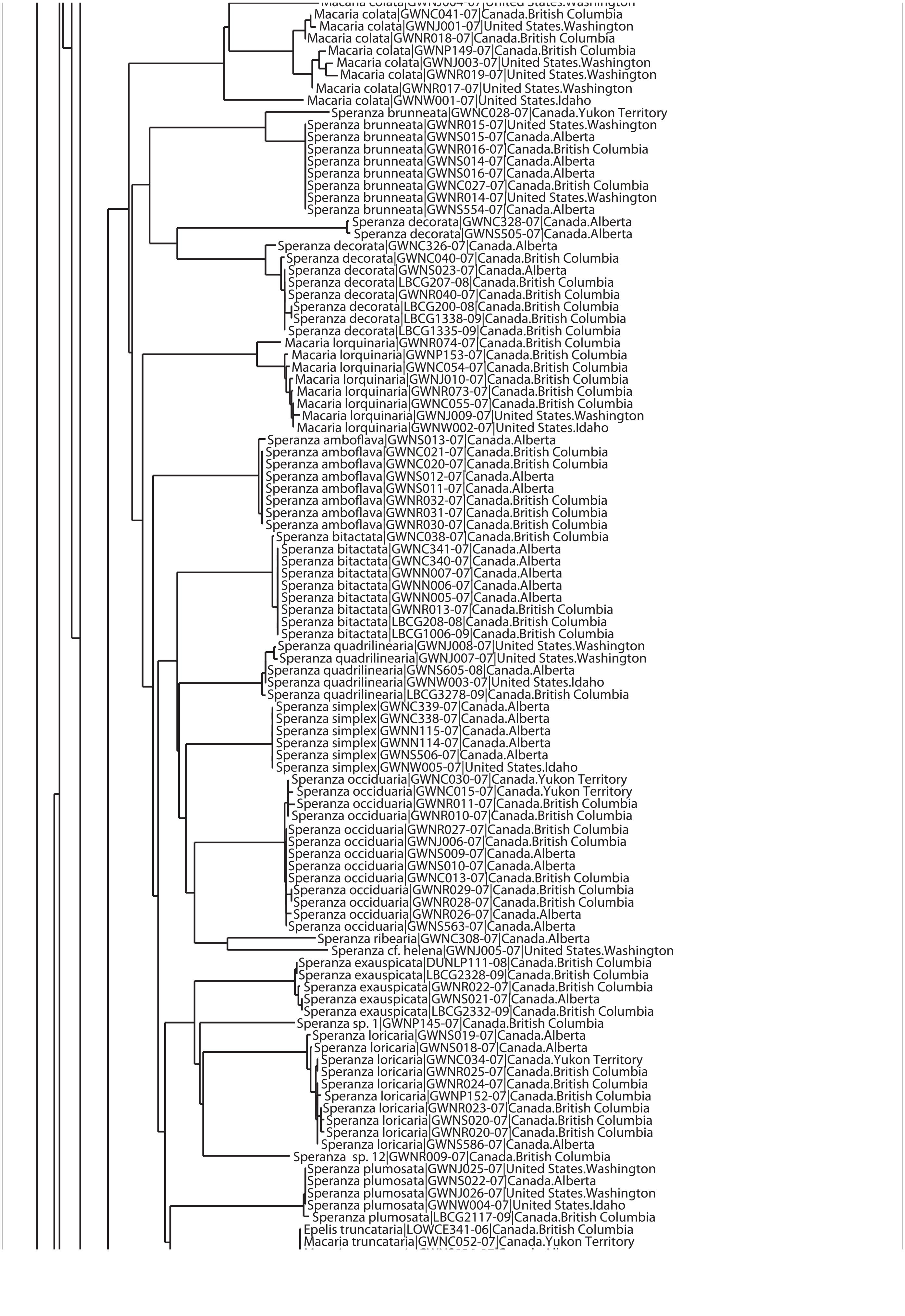

Macaria colata|GWNC041-07|Canada.British Columbia
Macaria colata|GWNJ001-07|United States.Washington
Macaria colata|GWNR018-07|Canada.British Columbia
Macaria colata|GWNP149-07|Canada.British Columbia
Macaria colata|GWNJ003-07|United States.Washington
Macaria colata|GWNR019-07|United States.Washington
Macaria colata|GWNR017-07|United States.Washington
Macaria colata|GWNW001-07|United States.Idaho
Speranza brunneata|GWNC028-07|Canada.Yukon Territory
Speranza brunneata|GWNR015-07|United States.Washington
Speranza brunneata|GWNS015-07|Canada.Alberta
Speranza brunneata|GWNR016-07|Canada.British Columbia
Speranza brunneata|GWNS014-07|Canada.Alberta
Speranza brunneata|GWNS016-07|Canada.Alberta
Speranza brunneata|GWNC027-07|Canada.British Columbia
Speranza brunneata|GWNR014-07|United States.Washington
Speranza brunneata|GWNS554-07|Canada.Alberta
Speranza decorata|GWNC328-07|Canada.Alberta
Speranza decorata|GWNS505-07|Canada.Alberta
Speranza decorata|GWNC326-07|Canada.Alberta
Speranza decorata|GWNC040-07|Canada.British Columbia
Speranza decorata|GWNS023-07|Canada.Alberta
Speranza decorata|LBCG207-08|Canada.British Columbia
Speranza decorata|GWNR040-07|Canada.British Columbia
Speranza decorata|LBCG200-08|Canada.British Columbia
Speranza decorata|LBCG1338-09|Canada.British Columbia
Speranza decorata|LBCG1335-09|Canada.British Columbia
Macaria lorquinaria|GWNR074-07|Canada.British Columbia
Macaria lorquinaria|GWNP153-07|Canada.British Columbia
Macaria lorquinaria|GWNC054-07|Canada.British Columbia
Macaria lorquinaria|GWNJ010-07|Canada.British Columbia
Macaria lorquinaria|GWNR073-07|Canada.British Columbia
Macaria lorquinaria|GWNC055-07|Canada.British Columbia
Macaria lorquinaria|GWNJ009-07|United States.Washington
Macaria lorquinaria|GWNW002-07|United States.Idaho
Speranza amboflava|GWNS013-07|Canada.Alberta
Speranza amboflava|GWNC021-07|Canada.British Columbia
Speranza amboflava|GWNC020-07|Canada.British Columbia
Speranza amboflava|GWNS012-07|Canada.Alberta
Speranza amboflava|GWNS011-07|Canada.Alberta
Speranza amboflava|GWNR032-07|Canada.British Columbia
Speranza amboflava|GWNR031-07|Canada.British Columbia
Speranza amboflava|GWNR030-07|Canada.British Columbia
Speranza bitactata|GWNC038-07|Canada.British Columbia
Speranza bitactata|GWNC341-07|Canada.Alberta
Speranza bitactata|GWNC340-07|Canada.Alberta
Speranza bitactata|GWNN007-07|Canada.Alberta
Speranza bitactata|GWNN006-07|Canada.Alberta
Speranza bitactata|GWNN005-07|Canada.Alberta
Speranza bitactata|GWNR013-07|Canada.British Columbia
Speranza bitactata|LBCG208-08|Canada.British Columbia
Speranza bitactata|LBCG1006-09|Canada.British Columbia
Speranza quadrilinearia|GWNJ008-07|United States.Washington
Speranza quadrilinearia|GWNJ007-07|United States.Washington
Speranza quadrilinearia|GWNS605-08|Canada.Alberta
Speranza quadrilinearia|GWNW003-07|United States.Idaho
Speranza quadrilinearia|LBCG3278-09|Canada.British Columbia
Speranza simplex|GWNC339-07|Canada.Alberta
Speranza simplex|GWNC338-07|Canada.Alberta
Speranza simplex|GWNN115-07|Canada.Alberta
Speranza simplex|GWNN114-07|Canada.Alberta
Speranza simplex|GWNS506-07|Canada.Alberta
Speranza simplex|GWNW005-07|United States.Idaho
Speranza occiduaria|GWNC030-07|Canada.Yukon Territory
Speranza occiduaria|GWNC015-07|Canada.Yukon Territory
Speranza occiduaria|GWNR011-07|Canada.British Columbia
Speranza occiduaria|GWNR010-07|Canada.British Columbia
Speranza occiduaria|GWNR027-07|Canada.British Columbia
Speranza occiduaria|GWNJ006-07|Canada.British Columbia
Speranza occiduaria|GWNS009-07|Canada.Alberta
Speranza occiduaria|GWNS010-07|Canada.Alberta
Speranza occiduaria|GWNC013-07|Canada.British Columbia
Speranza occiduaria|GWNR029-07|Canada.British Columbia
Speranza occiduaria|GWNR028-07|Canada.British Columbia
Speranza occiduaria|GWNR026-07|Canada.Alberta
Speranza occiduaria|GWNS563-07|Canada.Alberta
Speranza ribearia|GWNC308-07|Canada.Alberta
Speranza cf. helena|GWNJ005-07|United States.Washington
Speranza exauspicata|DUNLP111-08|Canada.British Columbia
Speranza exauspicata|LBCG2328-09|Canada.British Columbia
Speranza exauspicata|GWNR022-07|Canada.British Columbia
Speranza exauspicata|GWNS021-07|Canada.Alberta
Speranza exauspicata|LBCG2332-09|Canada.British Columbia
Speranza sp. 1|GWNP145-07|Canada.British Columbia
Speranza loricaria|GWNS019-07|Canada.Alberta
Speranza loricaria|GWNS018-07|Canada.Alberta
Speranza loricaria|GWNC034-07|Canada.Yukon Territory
Speranza loricaria|GWNR025-07|Canada.British Columbia
Speranza loricaria|GWNR024-07|Canada.British Columbia
Speranza loricaria|GWNP152-07|Canada.British Columbia
Speranza loricaria|GWNR023-07|Canada.British Columbia
Speranza loricaria|GWNS020-07|Canada.British Columbia
Speranza loricaria|GWNR020-07|Canada.British Columbia
Speranza loricaria|GWNS586-07|Canada.Alberta
Speranza sp. 12|GWNR009-07|Canada.British Columbia
Speranza plumosata|GWNJ025-07|United States.Washington
Speranza plumosata|GWNS022-07|Canada.Alberta
Speranza plumosata|GWNJ026-07|United States.Washington
Speranza plumosata|GWNW004-07|United States.Idaho
Speranza plumosata|LBCG2117-09|Canada.British Columbia
Epelis truncataria|LOWCE341-06|Canada.British Columbia
Macaria truncataria|GWNC052-07|Canada.Yukon Territory

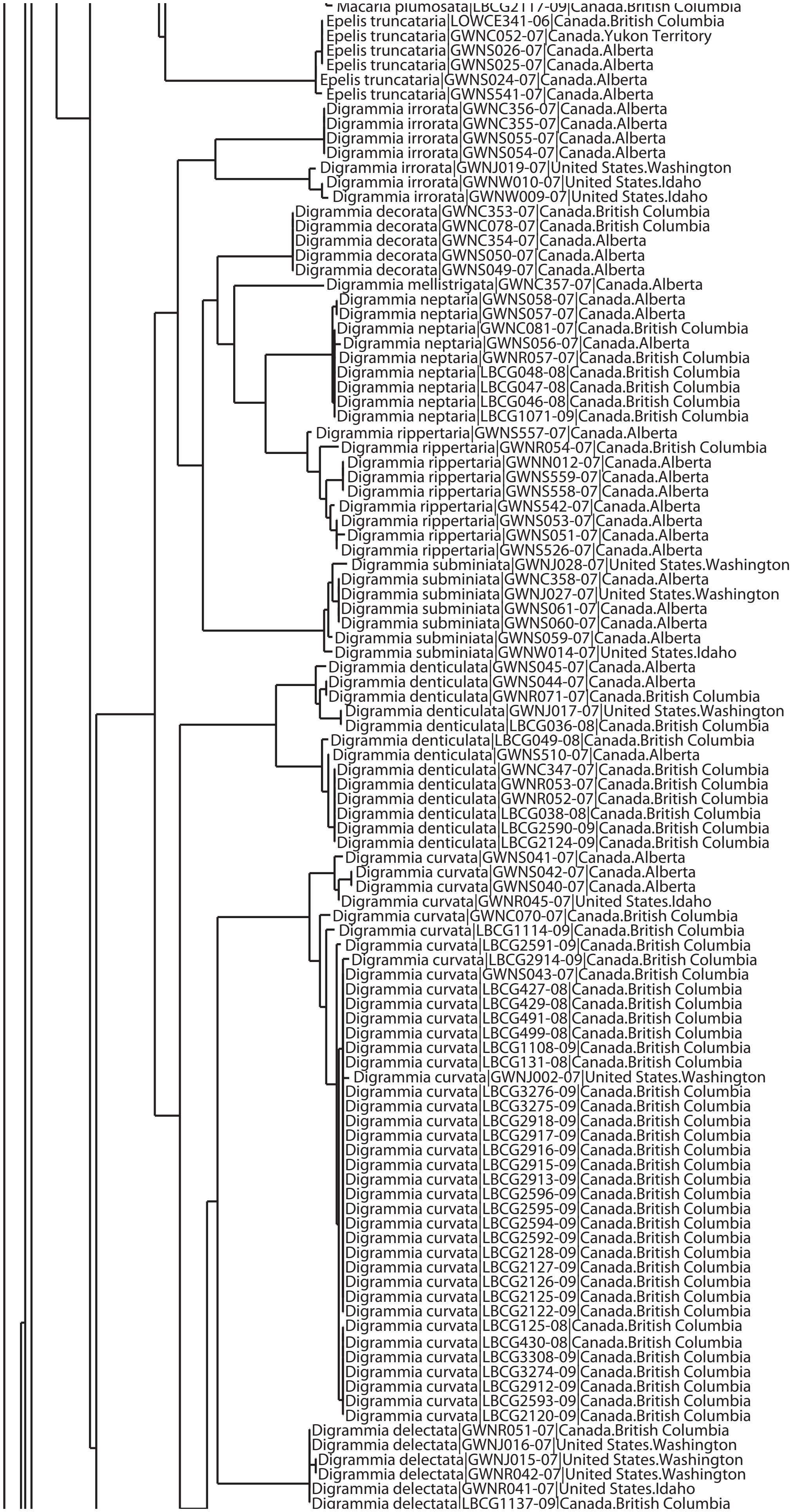
Macaria plumosata|LBCG2117-09|Canada.British Columbia
Epelis truncataria|LOWCE341-06|Canada.British Columbia
Epelis truncataria|GWNC052-07|Canada.Yukon Territory
Epelis truncataria|GWNS026-07|Canada.Alberta
Epelis truncataria|GWNS025-07|Canada.Alberta
Epelis truncataria|GWNS024-07|Canada.Alberta
Epelis truncataria|GWNS541-07|Canada.Alberta
Digrammia irrorata|GWNC356-07|Canada.Alberta
Digrammia irrorata|GWNC355-07|Canada.Alberta
Digrammia irrorata|GWNS055-07|Canada.Alberta
Digrammia irrorata|GWNS054-07|Canada.Alberta
Digrammia irrorata|GWNJ019-07|United States.Washington
Digrammia irrorata|GWNW010-07|United States.Idaho
Digrammia irrorata|GWNW009-07|United States.Idaho
Digrammia decorata|GWNC353-07|Canada.British Columbia
Digrammia decorata|GWNC078-07|Canada.British Columbia
Digrammia decorata|GWNC354-07|Canada.Alberta
Digrammia decorata|GWNS050-07|Canada.Alberta
Digrammia decorata|GWNS049-07|Canada.Alberta
Digrammia mellistrigata|GWNC357-07|Canada.Alberta
Digrammia neptaria|GWNS058-07|Canada.Alberta
Digrammia neptaria|GWNS057-07|Canada.Alberta
Digrammia neptaria|GWNC081-07|Canada.British Columbia
Digrammia neptaria|GWNS056-07|Canada.Alberta
Digrammia neptaria|GWNR057-07|Canada.British Columbia
Digrammia neptaria|LBCG048-08|Canada.British Columbia
Digrammia neptaria|LBCG047-08|Canada.British Columbia
Digrammia neptaria|LBCG046-08|Canada.British Columbia
Digrammia neptaria|LBCG1071-09|Canada.British Columbia
Digrammia rippertaria|GWNS557-07|Canada.Alberta
Digrammia rippertaria|GWNR054-07|Canada.British Columbia
Digrammia rippertaria|GWNN012-07|Canada.Alberta
Digrammia rippertaria|GWNS559-07|Canada.Alberta
Digrammia rippertaria|GWNS558-07|Canada.Alberta
Digrammia rippertaria|GWNS542-07|Canada.Alberta
Digrammia rippertaria|GWNS053-07|Canada.Alberta
Digrammia rippertaria|GWNS051-07|Canada.Alberta
Digrammia rippertaria|GWNS526-07|Canada.Alberta
Digrammia subminiata|GWNJ028-07|United States.Washington
Digrammia subminiata|GWNC358-07|Canada.Alberta
Digrammia subminiata|GWNJ027-07|United States.Washington
Digrammia subminiata|GWNS061-07|Canada.Alberta
Digrammia subminiata|GWNS060-07|Canada.Alberta
Digrammia subminiata|GWNS059-07|Canada.Alberta
Digrammia subminiata|GWNW014-07|United States.Idaho
Digrammia denticulata|GWNS045-07|Canada.Alberta
Digrammia denticulata|GWNS044-07|Canada.Alberta
Digrammia denticulata|GWNR071-07|Canada.British Columbia
Digrammia denticulata|GWNJ017-07|United States.Washington
Digrammia denticulata|LBCG036-08|Canada.British Columbia
Digrammia denticulata|LBCG049-08|Canada.British Columbia
Digrammia denticulata|GWNS510-07|Canada.Alberta
Digrammia denticulata|GWNC347-07|Canada.British Columbia
Digrammia denticulata|GWNR053-07|Canada.British Columbia
Digrammia denticulata|GWNR052-07|Canada.British Columbia
Digrammia denticulata|LBCG038-08|Canada.British Columbia
Digrammia denticulata|LBCG2590-09|Canada.British Columbia
Digrammia denticulata|LBCG2124-09|Canada.British Columbia
Digrammia curvata|GWNS041-07|Canada.Alberta
Digrammia curvata|GWNS042-07|Canada.Alberta
Digrammia curvata|GWNS040-07|Canada.Alberta
Digrammia curvata|GWNR045-07|United States.Idaho
Digrammia curvata|GWNC070-07|Canada.British Columbia
Digrammia curvata|LBCG1114-09|Canada.British Columbia
Digrammia curvata|LBCG2591-09|Canada.British Columbia
Digrammia curvata|LBCG2914-09|Canada.British Columbia
Digrammia curvata|GWNS043-07|Canada.British Columbia
Digrammia curvata|LBCG427-08|Canada.British Columbia
Digrammia curvata|LBCG429-08|Canada.British Columbia
Digrammia curvata|LBCG491-08|Canada.British Columbia
Digrammia curvata|LBCG499-08|Canada.British Columbia
Digrammia curvata|LBCG1108-09|Canada.British Columbia
Digrammia curvata|LBCG131-08|Canada.British Columbia
Digrammia curvata|GWNJ002-07|United States.Washington
Digrammia curvata|LBCG3276-09|Canada.British Columbia
Digrammia curvata|LBCG3275-09|Canada.British Columbia
Digrammia curvata|LBCG2918-09|Canada.British Columbia
Digrammia curvata|LBCG2917-09|Canada.British Columbia
Digrammia curvata|LBCG2916-09|Canada.British Columbia
Digrammia curvata|LBCG2915-09|Canada.British Columbia
Digrammia curvata|LBCG2913-09|Canada.British Columbia
Digrammia curvata|LBCG2596-09|Canada.British Columbia
Digrammia curvata|LBCG2595-09|Canada.British Columbia
Digrammia curvata|LBCG2594-09|Canada.British Columbia
Digrammia curvata|LBCG2592-09|Canada.British Columbia
Digrammia curvata|LBCG2128-09|Canada.British Columbia
Digrammia curvata|LBCG2127-09|Canada.British Columbia
Digrammia curvata|LBCG2126-09|Canada.British Columbia
Digrammia curvata|LBCG2125-09|Canada.British Columbia
Digrammia curvata|LBCG2122-09|Canada.British Columbia
Digrammia curvata|LBCG125-08|Canada.British Columbia
Digrammia curvata|LBCG430-08|Canada.British Columbia
Digrammia curvata|LBCG3308-09|Canada.British Columbia
Digrammia curvata|LBCG3274-09|Canada.British Columbia
Digrammia curvata|LBCG2912-09|Canada.British Columbia
Digrammia curvata|LBCG2593-09|Canada.British Columbia
Digrammia curvata|LBCG2120-09|Canada.British Columbia
Digrammia delectata|GWNR051-07|Canada.British Columbia
Digrammia delectata|GWNJ016-07|United States.Washington
Digrammia delectata|GWNJ015-07|United States.Washington
Digrammia delectata|GWNR042-07|United States.Washington
Digrammia delectata|GWNR041-07|United States.Idaho
Digrammia delectata|LBCG1137-09|Canada.British Columbia

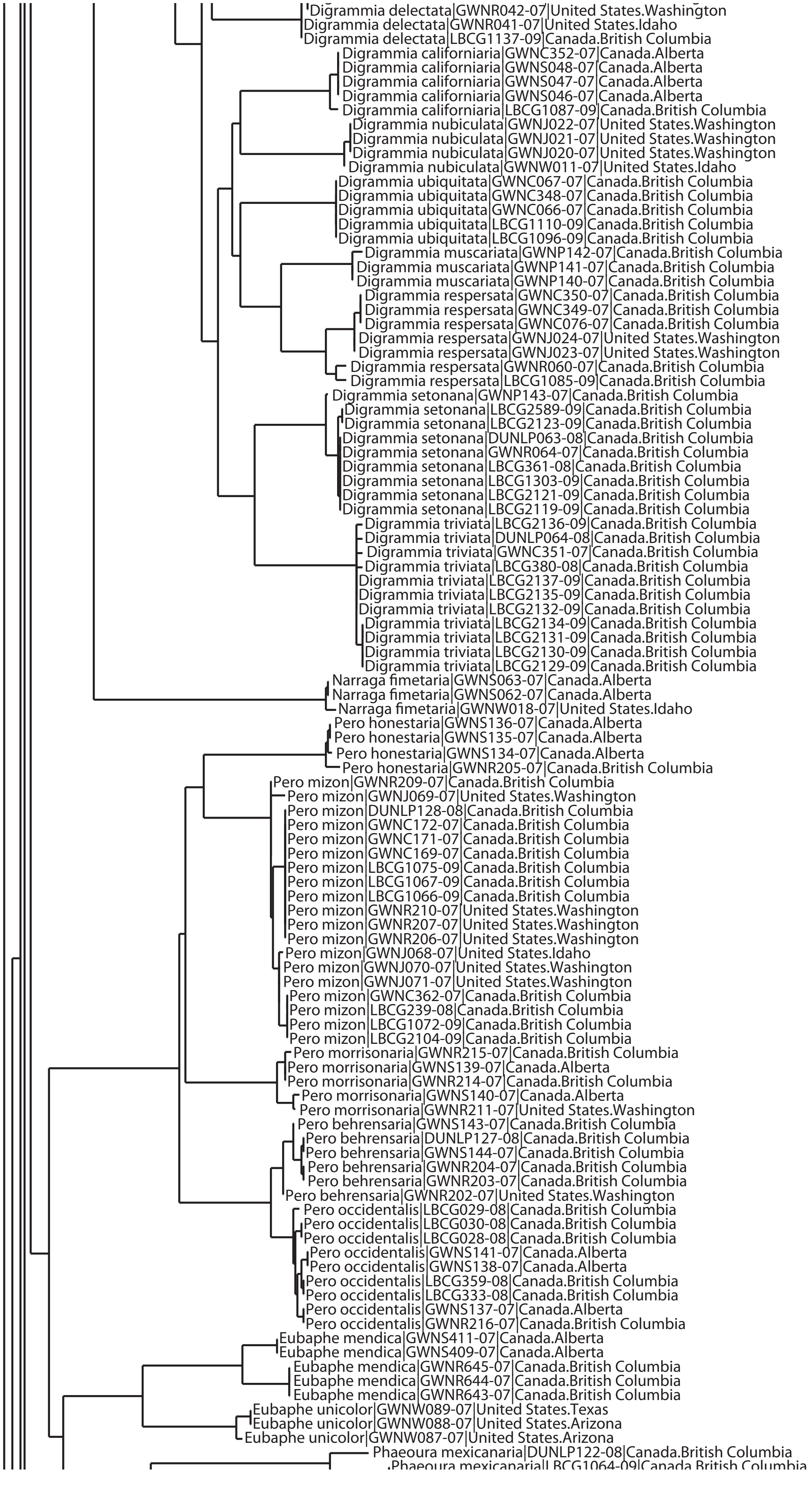
Digrammia delectata|GWNR042-07|United States.Washington
Digrammia delectata|GWNR041-07|United States.Idaho
Digrammia delectata|LBCG1137-09|Canada.British Columbia
Digrammia californiaria|GWNC352-07|Canada.Alberta
Digrammia californiaria|GWNS048-07|Canada.Alberta
Digrammia californiaria|GWNS047-07|Canada.Alberta
Digrammia californiaria|GWNS046-07|Canada.Alberta
Digrammia californiaria|LBCG1087-09|Canada.British Columbia
Digrammia nubiculata|GWNJ022-07|United States.Washington
Digrammia nubiculata|GWNJ021-07|United States.Washington
Digrammia nubiculata|GWNJ020-07|United States.Washington
Digrammia nubiculata|GWNW011-07|United States.Idaho
Digrammia ubiquitata|GWNC067-07|Canada.British Columbia
Digrammia ubiquitata|GWNC348-07|Canada.British Columbia
Digrammia ubiquitata|GWNC066-07|Canada.British Columbia
Digrammia ubiquitata|LBCG1110-09|Canada.British Columbia
Digrammia ubiquitata|LBCG1096-09|Canada.British Columbia
Digrammia muscariata|GWNP142-07|Canada.British Columbia
Digrammia muscariata|GWNP141-07|Canada.British Columbia
Digrammia muscariata|GWNP140-07|Canada.British Columbia
Digrammia respersata|GWNC350-07|Canada.British Columbia
Digrammia respersata|GWNC349-07|Canada.British Columbia
Digrammia respersata|GWNC076-07|Canada.British Columbia
Digrammia respersata|GWNJ024-07|United States.Washington
Digrammia respersata|GWNJ023-07|United States.Washington
Digrammia respersata|GWNR060-07|Canada.British Columbia
Digrammia respersata|LBCG1085-09|Canada.British Columbia
Digrammia setonana|GWNP143-07|Canada.British Columbia
Digrammia setonana|LBCG2589-09|Canada.British Columbia
Digrammia setonana|LBCG2123-09|Canada.British Columbia
Digrammia setonana|DUNLP063-08|Canada.British Columbia
Digrammia setonana|GWNR064-07|Canada.British Columbia
Digrammia setonana|LBCG361-08|Canada.British Columbia
Digrammia setonana|LBCG1303-09|Canada.British Columbia
Digrammia setonana|LBCG2121-09|Canada.British Columbia
Digrammia setonana|LBCG2119-09|Canada.British Columbia
Digrammia triviata|LBCG2136-09|Canada.British Columbia
Digrammia triviata|DUNLP064-08|Canada.British Columbia
Digrammia triviata|GWNC351-07|Canada.British Columbia
Digrammia triviata|LBCG380-08|Canada.British Columbia
Digrammia triviata|LBCG2137-09|Canada.British Columbia
Digrammia triviata|LBCG2135-09|Canada.British Columbia
Digrammia triviata|LBCG2132-09|Canada.British Columbia
Digrammia triviata|LBCG2134-09|Canada.British Columbia
Digrammia triviata|LBCG2131-09|Canada.British Columbia
Digrammia triviata|LBCG2130-09|Canada.British Columbia
Digrammia triviata|LBCG2129-09|Canada.British Columbia
Narraga fimetaria|GWNS063-07|Canada.Alberta
Narraga fimetaria|GWNS062-07|Canada.Alberta
Narraga fimetaria|GWNW018-07|United States.Idaho
Pero honestaria|GWNS136-07|Canada.Alberta
Pero honestaria|GWNS135-07|Canada.Alberta
Pero honestaria|GWNS134-07|Canada.Alberta
Pero honestaria|GWNR205-07|Canada.British Columbia
Pero mizon|GWNR209-07|Canada.British Columbia
Pero mizon|GWNJ069-07|United States.Washington
Pero mizon|DUNLP128-08|Canada.British Columbia
Pero mizon|GWNC172-07|Canada.British Columbia
Pero mizon|GWNC171-07|Canada.British Columbia
Pero mizon|GWNC169-07|Canada.British Columbia
Pero mizon|LBCG1075-09|Canada.British Columbia
Pero mizon|LBCG1067-09|Canada.British Columbia
Pero mizon|LBCG1066-09|Canada.British Columbia
Pero mizon|GWNR210-07|United States.Washington
Pero mizon|GWNR207-07|United States.Washington
Pero mizon|GWNR206-07|United States.Washington
Pero mizon|GWNJ068-07|United States.Idaho
Pero mizon|GWNJ070-07|United States.Washington
Pero mizon|GWNJ071-07|United States.Washington
Pero mizon|GWNC362-07|Canada.British Columbia
Pero mizon|LBCG239-08|Canada.British Columbia
Pero mizon|LBCG1072-09|Canada.British Columbia
Pero mizon|LBCG2104-09|Canada.British Columbia
Pero morrisonaria|GWNR215-07|Canada.British Columbia
Pero morrisonaria|GWNS139-07|Canada.Alberta
Pero morrisonaria|GWNR214-07|Canada.British Columbia
Pero morrisonaria|GWNS140-07|Canada.Alberta
Pero morrisonaria|GWNR211-07|United States.Washington
Pero behrensaria|GWNS143-07|Canada.British Columbia
Pero behrensaria|DUNLP127-08|Canada.British Columbia
Pero behrensaria|GWNS144-07|Canada.British Columbia
Pero behrensaria|GWNR204-07|Canada.British Columbia
Pero behrensaria|GWNR203-07|Canada.British Columbia
Pero behrensaria|GWNR202-07|United States.Washington
Pero occidentalis|LBCG029-08|Canada.British Columbia
Pero occidentalis|LBCG030-08|Canada.British Columbia
Pero occidentalis|LBCG028-08|Canada.British Columbia
Pero occidentalis|GWNS141-07|Canada.Alberta
Pero occidentalis|GWNS138-07|Canada.Alberta
Pero occidentalis|LBCG359-08|Canada.British Columbia
Pero occidentalis|LBCG333-08|Canada.British Columbia
Pero occidentalis|GWNS137-07|Canada.Alberta
Pero occidentalis|GWNR216-07|Canada.British Columbia
Eubaphe mendica|GWNS411-07|Canada.Alberta
Eubaphe mendica|GWNS409-07|Canada.Alberta
Eubaphe mendica|GWNR645-07|Canada.British Columbia
Eubaphe mendica|GWNR644-07|Canada.British Columbia
Eubaphe mendica|GWNR643-07|Canada.British Columbia
Eubaphe unicolor|GWNW089-07|United States.Texas
Eubaphe unicolor|GWNW088-07|United States.Arizona
Eubaphe unicolor|GWNW087-07|United States.Arizona
Phaeoura mexicanaria|DUNLP122-08|Canada.British Columbia
Phaeoura mexicanaria|LBCG1064-09|Canada.British Columbia

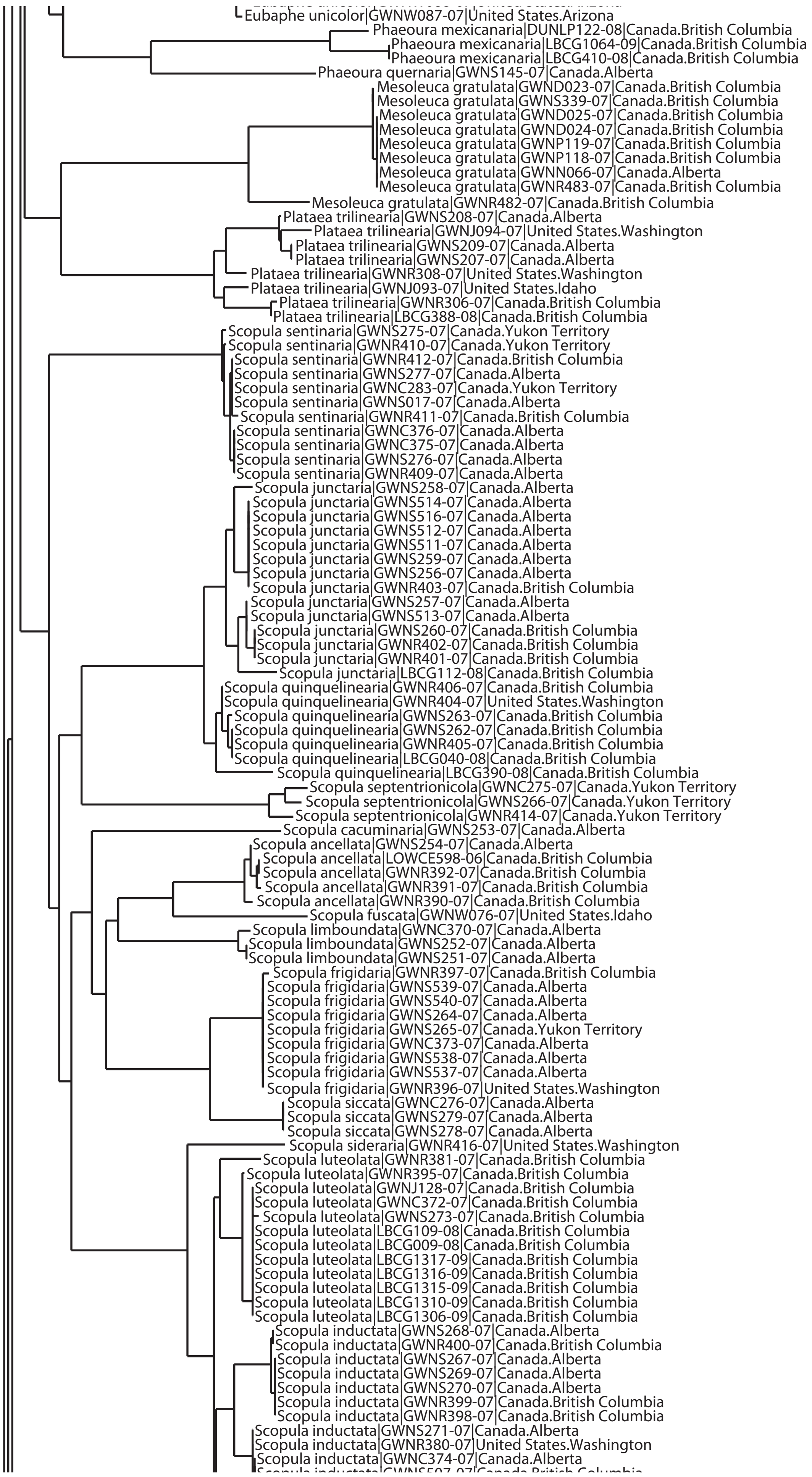
Eubaphe unicolor|GWNW087-07|United States.Arizona
Phaeoura mexicanaria|DUNLP122-08|Canada.British Columbia
Phaeoura mexicanaria|LBCG1064-09|Canada.British Columbia
Phaeoura mexicanaria|LBCG410-08|Canada.British Columbia
Phaeoura quernaria|GWNS145-07|Canada.Alberta
Mesoleuca gratulata|GWND023-07|Canada.British Columbia
Mesoleuca gratulata|GWNS339-07|Canada.British Columbia
Mesoleuca gratulata|GWND025-07|Canada.British Columbia
Mesoleuca gratulata|GWND024-07|Canada.British Columbia
Mesoleuca gratulata|GWNP119-07|Canada.British Columbia
Mesoleuca gratulata|GWNP118-07|Canada.British Columbia
Mesoleuca gratulata|GWNN066-07|Canada.Alberta
Mesoleuca gratulata|GWNR483-07|Canada.British Columbia
Mesoleuca gratulata|GWNR482-07|Canada.British Columbia
Plataea trilinearia|GWNS208-07|Canada.Alberta
Plataea trilinearia|GWNJ094-07|United States.Washington
Plataea trilinearia|GWNS209-07|Canada.Alberta
Plataea trilinearia|GWNS207-07|Canada.Alberta
Plataea trilinearia|GWNR308-07|United States.Washington
Plataea trilinearia|GWNJ093-07|United States.Idaho
Plataea trilinearia|GWNR306-07|Canada.British Columbia
Plataea trilinearia|LBCG388-08|Canada.British Columbia
Scopula sentinaria|GWNS275-07|Canada.Yukon Territory
Scopula sentinaria|GWNR410-07|Canada.Yukon Territory
Scopula sentinaria|GWNR412-07|Canada.British Columbia
Scopula sentinaria|GWNS277-07|Canada.Alberta
Scopula sentinaria|GWNC283-07|Canada.Yukon Territory
Scopula sentinaria|GWNS017-07|Canada.Alberta
Scopula sentinaria|GWNR411-07|Canada.British Columbia
Scopula sentinaria|GWNC376-07|Canada.Alberta
Scopula sentinaria|GWNC375-07|Canada.Alberta
Scopula sentinaria|GWNS276-07|Canada.Alberta
Scopula sentinaria|GWNR409-07|Canada.Alberta
Scopula junctaria|GWNS258-07|Canada.Alberta
Scopula junctaria|GWNS514-07|Canada.Alberta
Scopula junctaria|GWNS516-07|Canada.Alberta
Scopula junctaria|GWNS512-07|Canada.Alberta
Scopula junctaria|GWNS511-07|Canada.Alberta
Scopula junctaria|GWNS259-07|Canada.Alberta
Scopula junctaria|GWNS256-07|Canada.Alberta
Scopula junctaria|GWNR403-07|Canada.British Columbia
Scopula junctaria|GWNS257-07|Canada.Alberta
Scopula junctaria|GWNS513-07|Canada.Alberta
Scopula junctaria|GWNS260-07|Canada.British Columbia
Scopula junctaria|GWNR402-07|Canada.British Columbia
Scopula junctaria|GWNR401-07|Canada.British Columbia
Scopula junctaria|LBCG112-08|Canada.British Columbia
Scopula quinquelinearia|GWNR406-07|Canada.British Columbia
Scopula quinquelinearia|GWNR404-07|United States.Washington
Scopula quinquelinearia|GWNS263-07|Canada.British Columbia
Scopula quinquelinearia|GWNS262-07|Canada.British Columbia
Scopula quinquelinearia|GWNR405-07|Canada.British Columbia
Scopula quinquelinearia|LBCG040-08|Canada.British Columbia
Scopula quinquelinearia|LBCG390-08|Canada.British Columbia
Scopula septentrionicola|GWNC275-07|Canada.Yukon Territory
Scopula septentrionicola|GWNS266-07|Canada.Yukon Territory
Scopula septentrionicola|GWNR414-07|Canada.Yukon Territory
Scopula cacuminaria|GWNS253-07|Canada.Alberta
Scopula ancellata|GWNS254-07|Canada.Alberta
Scopula ancellata|LOWCE598-06|Canada.British Columbia
Scopula ancellata|GWNR392-07|Canada.British Columbia
Scopula ancellata|GWNR391-07|Canada.British Columbia
Scopula ancellata|GWNR390-07|Canada.British Columbia
Scopula fuscata|GWNW076-07|United States.Idaho
Scopula limboundata|GWNC370-07|Canada.Alberta
Scopula limboundata|GWNS252-07|Canada.Alberta
Scopula limboundata|GWNS251-07|Canada.Alberta
Scopula frigidaria|GWNR397-07|Canada.British Columbia
Scopula frigidaria|GWNS539-07|Canada.Alberta
Scopula frigidaria|GWNS540-07|Canada.Alberta
Scopula frigidaria|GWNS264-07|Canada.Alberta
Scopula frigidaria|GWNS265-07|Canada.Yukon Territory
Scopula frigidaria|GWNC373-07|Canada.Alberta
Scopula frigidaria|GWNS538-07|Canada.Alberta
Scopula frigidaria|GWNS537-07|Canada.Alberta
Scopula frigidaria|GWNR396-07|United States.Washington
Scopula siccata|GWNC276-07|Canada.Alberta
Scopula siccata|GWNS279-07|Canada.Alberta
Scopula siccata|GWNS278-07|Canada.Alberta
Scopula sideraria|GWNR416-07|United States.Washington
Scopula luteolata|GWNR381-07|Canada.British Columbia
Scopula luteolata|GWNR395-07|Canada.British Columbia
Scopula luteolata|GWNJ128-07|Canada.British Columbia
Scopula luteolata|GWNC372-07|Canada.British Columbia
Scopula luteolata|GWNS273-07|Canada.British Columbia
Scopula luteolata|LBCG109-08|Canada.British Columbia
Scopula luteolata|LBCG009-08|Canada.British Columbia
Scopula luteolata|LBCG1317-09|Canada.British Columbia
Scopula luteolata|LBCG1316-09|Canada.British Columbia
Scopula luteolata|LBCG1315-09|Canada.British Columbia
Scopula luteolata|LBCG1310-09|Canada.British Columbia
Scopula luteolata|LBCG1306-09|Canada.British Columbia
Scopula inductata|GWNS268-07|Canada.Alberta
Scopula inductata|GWNR400-07|Canada.British Columbia
Scopula inductata|GWNS267-07|Canada.Alberta
Scopula inductata|GWNS269-07|Canada.Alberta
Scopula inductata|GWNS270-07|Canada.Alberta
Scopula inductata|GWNR399-07|Canada.British Columbia
Scopula inductata|GWNR398-07|Canada.British Columbia
Scopula inductata|GWNS271-07|Canada.Alberta
Scopula inductata|GWNR380-07|United States.Washington
Scopula inductata|GWNC374-07|Canada.Alberta

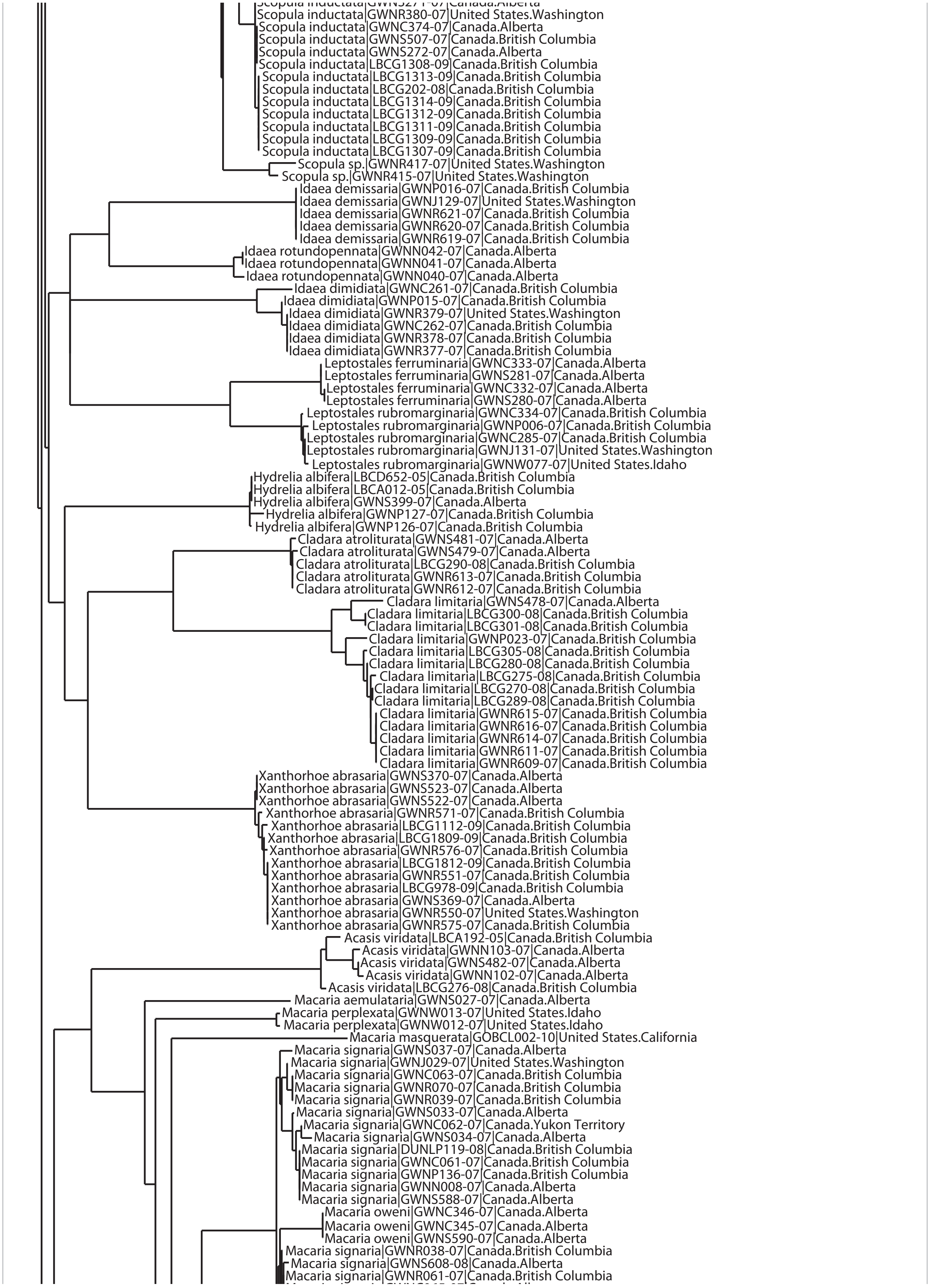

Scopula inductata|GWNS271-07|Canada.Alberta
Scopula inductata|GWNR380-07|United States.Washington
Scopula inductata|GWNC374-07|Canada.Alberta
Scopula inductata|GWNS507-07|Canada.British Columbia
Scopula inductata|GWNS272-07|Canada.Alberta
Scopula inductata|LBCG1308-09|Canada.British Columbia
Scopula inductata|LBCG1313-09|Canada.British Columbia
Scopula inductata|LBCG202-08|Canada.British Columbia
Scopula inductata|LBCG1314-09|Canada.British Columbia
Scopula inductata|LBCG1312-09|Canada.British Columbia
Scopula inductata|LBCG1311-09|Canada.British Columbia
Scopula inductata|LBCG1309-09|Canada.British Columbia
Scopula inductata|LBCG1307-09|Canada.British Columbia
Scopula sp.|GWNR417-07|United States.Washington
Scopula sp.|GWNR415-07|United States.Washington
Idaea demissaria|GWNP016-07|Canada.British Columbia
Idaea demissaria|GWNJ129-07|United States.Washington
Idaea demissaria|GWNR621-07|Canada.British Columbia
Idaea demissaria|GWNR620-07|Canada.British Columbia
Idaea demissaria|GWNR619-07|Canada.British Columbia
Idaea rotundopennata|GWNN042-07|Canada.Alberta
Idaea rotundopennata|GWNN041-07|Canada.Alberta
Idaea rotundopennata|GWNN040-07|Canada.Alberta
Idaea dimidiata|GWNC261-07|Canada.British Columbia
Idaea dimidiata|GWNP015-07|Canada.British Columbia
Idaea dimidiata|GWNR379-07|United States.Washington
Idaea dimidiata|GWNC262-07|Canada.British Columbia
Idaea dimidiata|GWNR378-07|Canada.British Columbia
Idaea dimidiata|GWNR377-07|Canada.British Columbia
Leptostales ferruminaria|GWNC333-07|Canada.Alberta
Leptostales ferruminaria|GWNS281-07|Canada.Alberta
Leptostales ferruminaria|GWNC332-07|Canada.Alberta
Leptostales ferruminaria|GWNS280-07|Canada.Alberta
Leptostales rubromarginaria|GWNC334-07|Canada.British Columbia
Leptostales rubromarginaria|GWNP006-07|Canada.British Columbia
Leptostales rubromarginaria|GWNC285-07|Canada.British Columbia
Leptostales rubromarginaria|GWNJ131-07|United States.Washington
Leptostales rubromarginaria|GWNW077-07|United States.Idaho
Hydrelia albifera|LBCD652-05|Canada.British Columbia
Hydrelia albifera|LBCA012-05|Canada.British Columbia
Hydrelia albifera|GWNS399-07|Canada.Alberta
Hydrelia albifera|GWNP127-07|Canada.British Columbia
Hydrelia albifera|GWNP126-07|Canada.British Columbia
Cladara atroliturata|GWNS481-07|Canada.Alberta
Cladara atroliturata|GWNS479-07|Canada.Alberta
Cladara atroliturata|LBCG290-08|Canada.British Columbia
Cladara atroliturata|GWNR613-07|Canada.British Columbia
Cladara atroliturata|GWNR612-07|Canada.British Columbia
Cladara limitaria|GWNS478-07|Canada.Alberta
Cladara limitaria|LBCG300-08|Canada.British Columbia
Cladara limitaria|LBCG301-08|Canada.British Columbia
Cladara limitaria|GWNP023-07|Canada.British Columbia
Cladara limitaria|LBCG305-08|Canada.British Columbia
Cladara limitaria|LBCG280-08|Canada.British Columbia
Cladara limitaria|LBCG275-08|Canada.British Columbia
Cladara limitaria|LBCG270-08|Canada.British Columbia
Cladara limitaria|LBCG289-08|Canada.British Columbia
Cladara limitaria|GWNR615-07|Canada.British Columbia
Cladara limitaria|GWNR616-07|Canada.British Columbia
Cladara limitaria|GWNR614-07|Canada.British Columbia
Cladara limitaria|GWNR611-07|Canada.British Columbia
Cladara limitaria|GWNR609-07|Canada.British Columbia
Xanthorhoe abrasaria|GWNS370-07|Canada.Alberta
Xanthorhoe abrasaria|GWNS523-07|Canada.Alberta
Xanthorhoe abrasaria|GWNS522-07|Canada.Alberta
Xanthorhoe abrasaria|GWNR571-07|Canada.British Columbia
Xanthorhoe abrasaria|LBCG1112-09|Canada.British Columbia
Xanthorhoe abrasaria|LBCG1809-09|Canada.British Columbia
Xanthorhoe abrasaria|GWNR576-07|Canada.British Columbia
Xanthorhoe abrasaria|LBCG1812-09|Canada.British Columbia
Xanthorhoe abrasaria|GWNR551-07|Canada.British Columbia
Xanthorhoe abrasaria|LBCG978-09|Canada.British Columbia
Xanthorhoe abrasaria|GWNS369-07|Canada.Alberta
Xanthorhoe abrasaria|GWNR550-07|United States.Washington
Xanthorhoe abrasaria|GWNR575-07|Canada.British Columbia
Acasis viridata|LBCA192-05|Canada.British Columbia
Acasis viridata|GWNN103-07|Canada.Alberta
Acasis viridata|GWNS482-07|Canada.Alberta
Acasis viridata|GWNN102-07|Canada.Alberta
Acasis viridata|LBCG276-08|Canada.British Columbia
Macaria aemulataria|GWNS027-07|Canada.Alberta
Macaria perplexata|GWNW013-07|United States.Idaho
Macaria perplexata|GWNW012-07|United States.Idaho
Macaria masquerata|GOBCL002-10|United States.California
Macaria signaria|GWNS037-07|Canada.Alberta
Macaria signaria|GWNJ029-07|United States.Washington
Macaria signaria|GWNC063-07|Canada.British Columbia
Macaria signaria|GWNR070-07|Canada.British Columbia
Macaria signaria|GWNR039-07|Canada.British Columbia
Macaria signaria|GWNS033-07|Canada.Alberta
Macaria signaria|GWNC062-07|Canada.Yukon Territory
Macaria signaria|GWNS034-07|Canada.Alberta
Macaria signaria|DUNLP119-08|Canada.British Columbia
Macaria signaria|GWNC061-07|Canada.British Columbia
Macaria signaria|GWNP136-07|Canada.British Columbia
Macaria signaria|GWNN008-07|Canada.Alberta
Macaria signaria|GWNS588-07|Canada.Alberta
Macaria oweni|GWNC346-07|Canada.Alberta
Macaria oweni|GWNC345-07|Canada.Alberta
Macaria oweni|GWNS590-07|Canada.Alberta
Macaria signaria|GWNR038-07|Canada.British Columbia
Macaria signaria|GWNS608-08|Canada.Alberta
Macaria signaria|GWNR061-07|Canada.British Columbia

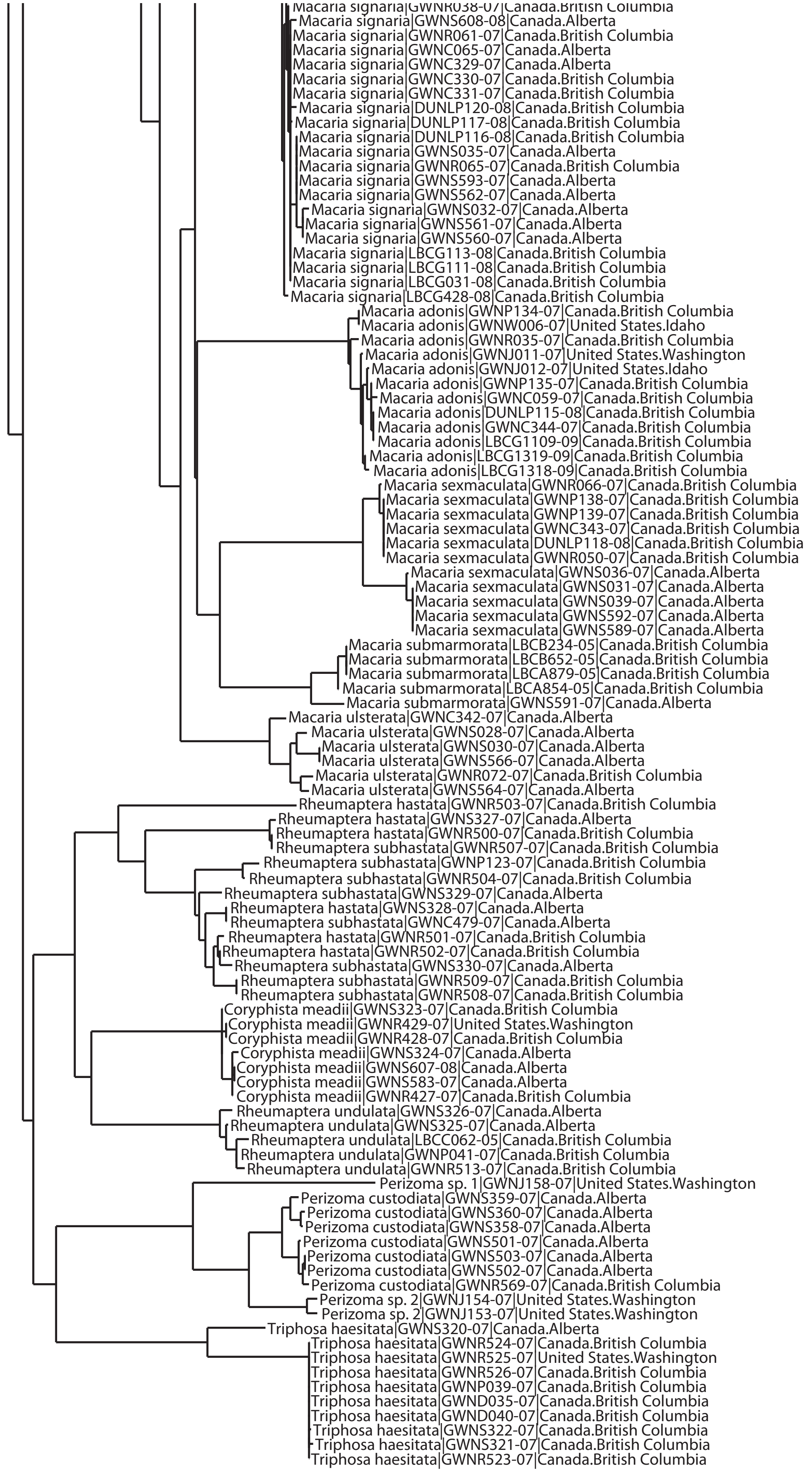
Macaria signaria|GWNR038-07|Canada.British Columbia
Macaria signaria|GWNS608-08|Canada.Alberta
Macaria signaria|GWNR061-07|Canada.British Columbia
Macaria signaria|GWNC065-07|Canada.Alberta
Macaria signaria|GWNC329-07|Canada.Alberta
Macaria signaria|GWNC330-07|Canada.British Columbia
Macaria signaria|GWNC331-07|Canada.British Columbia
Macaria signaria|DUNLP120-08|Canada.British Columbia
Macaria signaria|DUNLP117-08|Canada.British Columbia
Macaria signaria|DUNLP116-08|Canada.British Columbia
Macaria signaria|GWNS035-07|Canada.Alberta
Macaria signaria|GWNR065-07|Canada.British Columbia
Macaria signaria|GWNS593-07|Canada.Alberta
Macaria signaria|GWNS562-07|Canada.Alberta
Macaria signaria|GWNS032-07|Canada.Alberta
Macaria signaria|GWNS561-07|Canada.Alberta
Macaria signaria|GWNS560-07|Canada.Alberta
Macaria signaria|LBCG113-08|Canada.British Columbia
Macaria signaria|LBCG111-08|Canada.British Columbia
Macaria signaria|LBCG031-08|Canada.British Columbia
Macaria signaria|LBCG428-08|Canada.British Columbia
Macaria adonis|GWNP134-07|Canada.British Columbia
Macaria adonis|GWNW006-07|United States.Idaho
Macaria adonis|GWNR035-07|Canada.British Columbia
Macaria adonis|GWNJ011-07|United States.Washington
Macaria adonis|GWNJ012-07|United States.Idaho
Macaria adonis|GWNP135-07|Canada.British Columbia
Macaria adonis|GWNC059-07|Canada.British Columbia
Macaria adonis|DUNLP115-08|Canada.British Columbia
Macaria adonis|GWNC344-07|Canada.British Columbia
Macaria adonis|LBCG1109-09|Canada.British Columbia
Macaria adonis|LBCG1319-09|Canada.British Columbia
Macaria adonis|LBCG1318-09|Canada.British Columbia
Macaria sexmaculata|GWNR066-07|Canada.British Columbia
Macaria sexmaculata|GWNP138-07|Canada.British Columbia
Macaria sexmaculata|GWNP139-07|Canada.British Columbia
Macaria sexmaculata|GWNC343-07|Canada.British Columbia
Macaria sexmaculata|DUNLP118-08|Canada.British Columbia
Macaria sexmaculata|GWNR050-07|Canada.British Columbia
Macaria sexmaculata|GWNS036-07|Canada.Alberta
Macaria sexmaculata|GWNS031-07|Canada.Alberta
Macaria sexmaculata|GWNS039-07|Canada.Alberta
Macaria sexmaculata|GWNS592-07|Canada.Alberta
Macaria sexmaculata|GWNS589-07|Canada.Alberta
Macaria submarmorata|LBCB234-05|Canada.British Columbia
Macaria submarmorata|LBCB652-05|Canada.British Columbia
Macaria submarmorata|LBCA879-05|Canada.British Columbia
Macaria submarmorata|LBCA854-05|Canada.British Columbia
Macaria submarmorata|GWNS591-07|Canada.Alberta
Macaria ulsterata|GWNC342-07|Canada.Alberta
Macaria ulsterata|GWNS028-07|Canada.Alberta
Macaria ulsterata|GWNS030-07|Canada.Alberta
Macaria ulsterata|GWNS566-07|Canada.Alberta
Macaria ulsterata|GWNR072-07|Canada.British Columbia
Macaria ulsterata|GWNS564-07|Canada.Alberta
Rheumaptera hastata|GWNR503-07|Canada.British Columbia
Rheumaptera hastata|GWNS327-07|Canada.Alberta
Rheumaptera hastata|GWNR500-07|Canada.British Columbia
Rheumaptera subhastata|GWNR507-07|Canada.British Columbia
Rheumaptera subhastata|GWNP123-07|Canada.British Columbia
Rheumaptera subhastata|GWNR504-07|Canada.British Columbia
Rheumaptera subhastata|GWNS329-07|Canada.Alberta
Rheumaptera hastata|GWNS328-07|Canada.Alberta
Rheumaptera subhastata|GWNC479-07|Canada.Alberta
Rheumaptera hastata|GWNR501-07|Canada.British Columbia
Rheumaptera hastata|GWNR502-07|Canada.British Columbia
Rheumaptera subhastata|GWNS330-07|Canada.Alberta
Rheumaptera subhastata|GWNR509-07|Canada.British Columbia
Rheumaptera subhastata|GWNR508-07|Canada.British Columbia
Coryphista meadii|GWNS323-07|Canada.British Columbia
Coryphista meadii|GWNR429-07|United States.Washington
Coryphista meadii|GWNR428-07|Canada.British Columbia
Coryphista meadii|GWNS324-07|Canada.Alberta
Coryphista meadii|GWNS607-08|Canada.Alberta
Coryphista meadii|GWNS583-07|Canada.Alberta
Coryphista meadii|GWNR427-07|Canada.British Columbia
Rheumaptera undulata|GWNS326-07|Canada.Alberta
Rheumaptera undulata|GWNS325-07|Canada.Alberta
Rheumaptera undulata|LBCC062-05|Canada.British Columbia
Rheumaptera undulata|GWNP041-07|Canada.British Columbia
Rheumaptera undulata|GWNR513-07|Canada.British Columbia
Perizoma sp. 1|GWNJ158-07|United States.Washington
Perizoma custodiata|GWNS359-07|Canada.Alberta
Perizoma custodiata|GWNS360-07|Canada.Alberta
Perizoma custodiata|GWNS358-07|Canada.Alberta
Perizoma custodiata|GWNS501-07|Canada.Alberta
Perizoma custodiata|GWNS503-07|Canada.Alberta
Perizoma custodiata|GWNS502-07|Canada.Alberta
Perizoma custodiata|GWNR569-07|Canada.British Columbia
Perizoma sp. 2|GWNJ154-07|United States.Washington
Perizoma sp. 2|GWNJ153-07|United States.Washington
Triphosa haesitata|GWNS320-07|Canada.Alberta
Triphosa haesitata|GWNR524-07|Canada.British Columbia
Triphosa haesitata|GWNR525-07|United States.Washington
Triphosa haesitata|GWNR526-07|Canada.British Columbia
Triphosa haesitata|GWNP039-07|Canada.British Columbia
Triphosa haesitata|GWND035-07|Canada.British Columbia
Triphosa haesitata|GWND040-07|Canada.British Columbia
Triphosa haesitata|GWNS322-07|Canada.British Columbia
Triphosa haesitata|GWNS321-07|Canada.British Columbia
Triphosa haesitata|GWNR523-07|Canada.British Columbia
