# Supplementary material for: A Comprehensive DNA Barcode Library for the Looper Moths (Lepidoptera: Geometridae) of British Columbia, Canada
Source: PLoS One. 2011 Mar 28;6(3):e18290. doi: 10.1371/journal.pone.0018290 (PMC3065486; doi:10.1371/journal.pone.0018290)
Supplement: Table S1 — List of specimens analyzed in the present study. Specimen accessions, BOLD process IDs, GenBank accessions, collection localities, and the storing institution are provided for each specimen. (PDF) [file pone.0018290.s002.pdf]

Species	Specimen ID	BOLD ID	GenBank Acc.	Country	State/Province	Institution Storing
Acasis viridata	08-JDWBC-0276	LBCG276-08	HQ647388	Canada	British Columbia	Spencer Entomological Museum, UBC
Acasis viridata	HLC-20192	LBCA192-05	HQ647384	Canada	British Columbia	Biodiversity Institute of Ontario
Acasis viridata	NFRC-P-2007-100093	GWNN102-07	HQ647387	Canada	Alberta	Northern Forestry Centre, Canadian Forest Service
Acasis viridata	NFRC-P-2007-100094	GWNN103-07	HQ647386	Canada	Alberta	Northern Forestry Centre, Canadian Forest Service
Acasis viridata	UASM57989	GWNS482-07	HQ647385	Canada	Alberta	Strickland Museum of Entomology, University of Alberta
Aethalura intertexta	07-JDWBC-0044	GWND036-07	HQ647393	Canada	British Columbia	Royal British Columbia Museum
Aethalura intertexta	07-JDWBC-0052	GWND044-07	HQ647392	Canada	British Columbia	Royal British Columbia Museum
Aethalura intertexta	07-JDWBC-0053	GWND045-07	HQ647391	Canada	British Columbia	Royal British Columbia Museum
Aethalura intertexta	CNCLEP00033095	GWNC096-07	HQ647390	Canada	Alberta	Canadian National Collection of Insects, Arachnids and Nematodes
Aethalura intertexta	CNCLEP00033096	GWNC097-07	HQ647389	Canada	British Columbia	Canadian National Collection of Insects, Arachnids and Nematodes
Aethalura intertexta	ENT996-003503	GWNR076-07	HQ647397	Canada	British Columbia	Royal British Columbia Museum
Aethalura intertexta	SEM-UBC-GEO-0082	GWNU040-07	HQ647398	Canada	British Columbia	Spencer Entomological Museum, UBC
Aethalura intertexta	UASM41863	GWNS074-07	HQ647395	Canada	Alberta	Strickland Museum of Entomology, University of Alberta
Aethalura intertexta	UASM58063	GWNS073-07	HQ647396	Canada	Alberta	Strickland Museum of Entomology, University of Alberta
Aethalura intertexta	UASM58363	GWNS075-07	HQ647394	Canada	Alberta	Strickland Museum of Entomology, University of Alberta
Alsophila pometaria	CNCLEP00033306	GWNC307-07	HQ647399	Canada	Alberta	Canadian National Collection of Insects, Arachnids and Nematodes
Anavitrinella addendaria	09-JDWGEO-468	GNAU168-10	HM392443	United States	Nevada	Smithsonian Institution
Anavitrinella addendaria	09-JDWGEO-470	GNAU170-10	HM392444	United States	Nevada	Smithsonian Institution
Anavitrinella addendaria	09-JDWGEO-471	GNAU171-10	HM392445	United States	Texas	Smithsonian Institution
Anavitrinella addendaria	09-JDWGEO-473	GNAU173-10	HM392446	United States	Texas	Smithsonian Institution
Anavitrinella addendaria	WFBM-07-0025	GWNW025-07	HQ647400	United States	Idaho	University of Idaho, W. F. Barr Entomological Collection
Anavitrinella pampinaria	Dun-08-059	DUNLP059-08	HQ647401	Canada	British Columbia	Pacific Forestry Centre, Canadian Forest Service
Anavitrinella pampinaria	ENT991-009990	GWNR078-07	HQ647405	Canada	British Columbia	Royal British Columbia Museum
Anavitrinella pampinaria	ENT996-003594	GWNR082-07	HQ647404	Canada	British Columbia	Royal British Columbia Museum
Anavitrinella pampinaria	UASM57120	GWNS078-07	HQ647403	Canada	Alberta	Strickland Museum of Entomology, University of Alberta
Anavitrinella pampinaria	UASM57250	GWNS079-07	HQ647402	Canada	Alberta	Strickland Museum of Entomology, University of Alberta
Anticlea multiferata	CNCLEP00033408	GWNC409-07	HQ647406	Canada	Alberta	Canadian National Collection of Insects, Arachnids and Nematodes
Anticlea multiferata	NFRC-P-2007-100070	GWNN070-07	HQ647408	Canada	Alberta	Northern Forestry Centre, Canadian Forest Service
Anticlea multiferata	UASM7017	GWNS365-07	HQ647407	Canada	Alberta	Strickland Museum of Entomology, University of Alberta
Anticlea multiferata	UASM95875	GWNS546-07	HQ647410	Canada	Alberta	Strickland Museum of Entomology, University of Alberta
Anticlea multiferata	UASM95877	GWNS548-07	HQ647409	Canada	Alberta	Strickland Museum of Entomology, University of Alberta
Anticlea vasiliata	07-JDWBC-0004	GWND004-07	HQ647414	Canada	British Columbia	Royal British Columbia Museum
Anticlea vasiliata	07-JDWBC-0025	GWND018-07	HQ647413	Canada	British Columbia	Royal British Columbia Museum
Anticlea vasiliata	07-JDWBC-0039	GWND031-07	HQ647412	Canada	British Columbia	Royal British Columbia Museum
Anticlea vasiliata	07-JDWBC-0051	GWND043-07	HQ647411	Canada	British Columbia	Royal British Columbia Museum
Anticlea vasiliata	08-JDWBC-0268	LBCG268-08	HQ647425	Canada	British Columbia	Spencer Entomological Museum, UBC
Anticlea vasiliata	08-JDWBC-0274	LBCG274-08	HQ647426	Canada	British Columbia	Spencer Entomological Museum, UBC
Anticlea vasiliata	08-JDWBC-0285	LBCG285-08	HQ647427	Canada	British Columbia	Spencer Entomological Museum, UBC
Anticlea vasiliata	08-JDWBC-0287	LBCG287-08	HQ647428	Canada	British Columbia	Spencer Entomological Museum, UBC
Anticlea vasiliata	08-JDWBC-0291	LBCG291-08	HQ647429	Canada	British Columbia	Spencer Entomological Museum, UBC
Anticlea vasiliata	08-JDWBC-0294	LBCG294-08	HQ647430	Canada	British Columbia	Spencer Entomological Museum, UBC
Anticlea vasiliata	08-JDWBC-0298	LBCG298-08	HQ647423	Canada	British Columbia	Spencer Entomological Museum, UBC
Anticlea vasiliata	08-JDWBC-0302	LBCG302-08	HQ647424	Canada	British Columbia	Spencer Entomological Museum, UBC
Anticlea vasiliata	08-JDWBC-0303	LBCG303-08	HQ647431	Canada	British Columbia	Spencer Entomological Museum, UBC
Anticlea vasiliata	08-JDWBC-0326	LBCG326-08	HQ647422	Canada	British Columbia	Spencer Entomological Museum, UBC
Anticlea vasiliata	08-JDWBC-0327	LBCG327-08	HQ647421	Canada	British Columbia	Spencer Entomological Museum, UBC
Anticlea vasiliata	ENT002-001713	GWNR420-07	HQ647419	Canada	British Columbia	Royal British Columbia Museum
Anticlea vasiliata	ENT996-004604	GWNR419-07	HQ647420	United States	Washington	Royal British Columbia Museum
Anticlea vasiliata	UASM28798	GWNS364-07	HQ647417	Canada	Alberta	Strickland Museum of Entomology, University of Alberta
Anticlea vasiliata	UASM59734	GWNS362-07	HQ647415	Canada	British Columbia	Strickland Museum of Entomology, University of Alberta
Anticlea vasiliata	UASM59735	GWNS361-07	HQ647416	Canada	British Columbia	Strickland Museum of Entomology, University of Alberta
Anticlea vasiliata	UASM7016	GWNS363-07	HQ647418	Canada	Alberta	Strickland Museum of Entomology, University of Alberta
Aplocera plagiata	08-JDWBC-0024	LBCG024-08	HQ647442	Canada	British Columbia	Spencer Entomological Museum, UBC
Aplocera plagiata	08-JDWBC-0025	LBCG025-08	HQ647441	Canada	British Columbia	Spencer Entomological Museum, UBC
Aplocera plagiata	08-JDWBC-0026	LBCG026-08	HQ647440	Canada	British Columbia	Spencer Entomological Museum, UBC
Aplocera plagiata	CNCLEP00033398	GWNC399-07	HQ647433	Canada	British Columbia	Canadian National Collection of Insects, Arachnids and Nematodes
Aplocera plagiata	CNCLEP00033399	GWNC400-07	HQ647432	Canada	British Columbia	Canadian National Collection of Insects, Arachnids and Nematodes
Aplocera plagiata	CNCLEP00035464	GWNC698-07	HQ647434	Canada	British Columbia	Canadian National Collection of Insects, Arachnids and Nematodes
Aplocera plagiata	ENT007-000946	GWNR649-07	HQ647444	Canada	British Columbia	Royal British Columbia Museum
Aplocera plagiata	ENT007-000947	GWNR650-07	HQ647443	United States	Washington	Royal British Columbia Museum
Aplocera plagiata	JD1779_UASM	GWNS596-07	HQ647436	Canada	British Columbia	Strickland Museum of Entomology, University of Alberta
Aplocera plagiata	JECW-07-0175	GWNJ175-07	HQ647435	United States	Idaho	James Entomological Collection, Washington State University
Aplocera plagiata	JECW-07-0176	GWNJ176-07	HQ647439	United States	Washington	James Entomological Collection, Washington State University
Aplocera plagiata	PFC-2007-0227	GWNP020-07	HQ647437	Canada	British Columbia	Pacific Forestry Centre, Canadian Forest Service
Aplocera plagiata	WFBM-07-0090	GWNW090-07	HQ647438	United States	Idaho	University of Idaho, W. F. Barr Entomological Collection
Apodrepanulatrix litaria	ENT996-003737	GWNR164-07	HQ647447	Canada	British Columbia	Royal British Columbia Museum
Apodrepanulatrix litaria	ENT996-007425	GWNR337-07	HQ647446	Canada	British Columbia	Royal British Columbia Museum
Apodrepanulatrix litaria	ENT996-007426	GWNR338-07	HQ647445	Canada	British Columbia	Royal British Columbia Museum
Archiearis infans	CGWC-3957	LOWCE197-06	HQ647449	Canada	British Columbia	Biodiversity Institute of Ontario
Archiearis infans	CGWC-3960	LOWCE200-06	HQ647448	Canada	British Columbia	Biodiversity Institute of Ontario
Archiearis infans	ENT006-000367	GWNR655-07	HQ647452	Canada	British Columbia	Royal British Columbia Museum
Archiearis infans	UASM24822	GWNS002-07	HQ647450	Canada	Alberta	Strickland Museum of Entomology, University of Alberta
Archiearis infans	UASM59113	GWNS001-07	HQ647451	Canada	Alberta	Strickland Museum of Entomology, University of Alberta
Aspitates aberrata	CBCC1026	GWNS598-07	HQ647460	Canada	Alberta	Strickland Museum of Entomology, University of Alberta
Aspitates aberrata	ENT002-001591	GWNR183-07	HQ647461	Canada	British Columbia	Royal British Columbia Museum
Aspitates aberrata	ENT002-001592	GWNR182-07	HQ647462	Canada	British Columbia	Royal British Columbia Museum
Aspitates aberrata	ENT002-001598	GWNR181-07	HQ647463	Canada	British Columbia	Royal British Columbia Museum
Aspitates aberrata	ENT996-003806	GWNR180-07	HQ647464	Canada	Yukon Territory	Royal British Columbia Museum
Aspitates aberrata	ENT996-003814	GWNR179-07	HQ647465	Canada	Yukon Territory	Royal British Columbia Museum
Aspitates aberrata	UASM41548	GWNS601-07	HQ647457	Canada	Alberta	Strickland Museum of Entomology, University of Alberta
Aspitates aberrata	UASM41549	GWNS600-07	HQ647458	Canada	Alberta	Strickland Museum of Entomology, University of Alberta
Aspitates aberrata	UASM41550	GWNS111-07	HQ647453	Canada	Alberta	Strickland Museum of Entomology, University of Alberta
Aspitates aberrata	UASM41850	GWNS602-07	HQ647456	Canada	Alberta	Strickland Museum of Entomology, University of Alberta
Aspitates aberrata	UASM58225	GWNS599-07	HQ647459	Canada	Alberta	Strickland Museum of Entomology, University of Alberta
Aspitates aberrata	UASM58226	GWNS604-07	HQ647455	Canada	Alberta	Strickland Museum of Entomology, University of Alberta
Aspitates aberrata	UASM58227	GWNS110-07	HQ647454	Canada	Alberta	Strickland Museum of Entomology, University of Alberta
Aspitates forbesi	CNCLEP00033155	GWNC156-07	HQ647466	Canada	Yukon Territory	Canadian National Collection of Insects, Arachnids and Nematodes
Aspitates orciferaria	CNCLEP00033156	GWNC157-07	HQ647467	Canada	Yukon Territory	Canadian National Collection of Insects, Arachnids and Nematodes
Aspitates taylori	CNCLEP00033159	GWNC160-07	HQ647468	Canada	Yukon Territory	Canadian National Collection of Insects, Arachnids and Nematodes
Aspitates taylori	UASM53010	GWNS113-07	HQ647471	Canada	Alberta	Strickland Museum of Entomology, University of Alberta
Aspitates taylori	UASM78370	GWNS114-07	HQ647470	Canada	Alberta	Strickland Museum of Entomology, University of Alberta
Aspitates taylori	UASM78373	GWNS115-07	HQ647469	Canada	Alberta	Strickland Museum of Entomology, University of Alberta
Besma quercivoraria	CNCLEP00033207	GWNC208-07	HQ647473	Canada	British Columbia	Canadian National Collection of Insects, Arachnids and Nematodes
Besma quercivoraria	HLC-20103	LBCA103-05	HQ647472	Canada	British Columbia	Biodiversity Institute of Ontario
Besma quercivoraria	NFRC-P-2007-100033	GWNN033-07	HQ647474	Canada	Alberta	Northern Forestry Centre, Canadian Forest Service
Besma quercivoraria	UASM7115	GWNS200-07	HQ647475	Canada	Alberta	Strickland Museum of Entomology, University of Alberta
Besma quercivoraria	UASM78416	GWNS199-07	HQ647476	Canada	Alberta	Strickland Museum of Entomology, University of Alberta
Biston betularia	08-JDWBC-0023	LBCG023-08	HQ647491	Canada	British Columbia	Spencer Entomological Museum, UBC
Biston betularia	08-JDWBC-2919	LBCG2919-09	HQ647492	Canada	British Columbia	Spencer Entomological Museum, UBC
Biston betularia	CNCLEP00034190	GWNC628-07	HQ647479	Canada	British Columbia	Canadian National Collection of Insects, Arachnids and Nematodes
Biston betularia	CNCLEP00035404	GWNC640-07	HQ647478	Canada	British Columbia	Canadian National Collection of Insects, Arachnids and Nematodes
Biston betularia	CNCLEP00035406	GWNC642-07	HQ647477	Canada	British Columbia	Canadian National Collection of Insects, Arachnids and Nematodes
Biston betularia	CNCLEP00035407	GWNC643-07	HQ647481	Canada	British Columbia	Canadian National Collection of Insects, Arachnids and Nematodes
Biston betularia	CNCLEP00035427	GWNC661-07	HQ647480	Canada	British Columbia	Canadian National Collection of Insects, Arachnids and Nematodes
Biston betularia	ENT002-001549	GWNR128-07	HQ647486	Canada	British Columbia	Royal British Columbia Museum
Biston betularia	ENT991-009890	GWNR125-07	HQ647488	Canada	British Columbia	Royal British Columbia Museum

Biston betularia	ENT996-003620	GWNR123-07	HQ647490	Canada	British Columbia	Royal British Columbia Museum
Biston betularia	ENT996-003625	GWNR127-07	HQ647487	Canada	British Columbia	Royal British Columbia Museum
Biston betularia	ENT996-003671	GWNR124-07	HQ647489	Canada	British Columbia	Royal British Columbia Museum
Biston betularia	NFRC-P-2007-100024	GWNN024-07	HQ647484	Canada	Alberta	Northern Forestry Centre, Canadian Forest Service
Biston betularia	UASM43300	GWNS092-07	HQ647483	Canada	Alberta	Strickland Museum of Entomology, University of Alberta
Biston betularia	UASM95800	GWNS090-07	HQ647482	Canada	British Columbia	Strickland Museum of Entomology, University of Alberta
Biston betularia	WFBM-07-0032	GWNW032-07	HQ647485	United States	Idaho	University of Idaho, W. F. Barr Entomological Collection
Cabera borealis	UASM53018	GWNS531-07	HQ647495	Canada	Alberta	Strickland Museum of Entomology, University of Alberta
Cabera borealis	UASM53019	GWNS528-07	HQ647498	Canada	Alberta	Strickland Museum of Entomology, University of Alberta
Cabera borealis	UASM53020	GWNS529-07	HQ647497	Canada	Alberta	Strickland Museum of Entomology, University of Alberta
Cabera borealis	UASM53021	GWNS530-07	HQ647496	Canada	Alberta	Strickland Museum of Entomology, University of Alberta
Cabera borealis	UASM53022	GWNS532-07	HQ647494	Canada	Alberta	Strickland Museum of Entomology, University of Alberta
Cabera borealis	UASM53023	GWNS533-07	HQ647493	Canada	Alberta	Strickland Museum of Entomology, University of Alberta
Cabera erythemaria	ENT002-001580	GWNR151-07	HQ647504	Canada	British Columbia	Royal British Columbia Museum
Cabera erythemaria	ENT002-001581	GWNR152-07	HQ647503	Canada	British Columbia	Royal British Columbia Museum
Cabera erythemaria	ENT996-003751	GWNR150-07	HQ647505	United States	Washington	Royal British Columbia Museum
Cabera erythemaria	UASM58283	GWNS104-07	HQ647500	Canada	Alberta	Strickland Museum of Entomology, University of Alberta
Cabera erythemaria	UASM58784	GWNS105-07	HQ647499	Canada	Alberta	Strickland Museum of Entomology, University of Alberta
Cabera erythemaria	UASM78475	GWNS103-07	HQ647501	Canada	Alberta	Strickland Museum of Entomology, University of Alberta
Cabera erythemaria	WFBM-07-0036	GWNW036-07	HQ647502	United States	Idaho	University of Idaho, W. F. Barr Entomological Collection
Cabera exanthemata	ENT002-001577	GWNR155-07	HQ647506	Canada	British Columbia	Royal British Columbia Museum
Cabera exanthemata	ENT996-003649	GWNR153-07	HQ647507	Canada	British Columbia	Royal British Columbia Museum
Cabera variolaria	ENT002-001588	GWNR158-07	HQ647512	Canada	British Columbia	Royal British Columbia Museum
Cabera variolaria	HLC-20045	LBCA045-05	HQ647508	Canada	British Columbia	Biodiversity Institute of Ontario
Cabera variolaria	UASM57238	GWNS106-07	HQ647510	Canada	Alberta	Strickland Museum of Entomology, University of Alberta
Cabera variolaria	UASM58128	GWNS107-07	HQ647509	Canada	Alberta	Strickland Museum of Entomology, University of Alberta
Cabera variolaria	UASM77909_duplicate	GWNS595-07	HQ647511	Canada	Alberta	Strickland Museum of Entomology, University of Alberta
Callizzia amorata	JECW-07-0222	GWNJ222-07	HQ647513	Canada	British Columbia	James Entomological Collection, Washington State University
Campaea perlata	08-JDWBC-0759	LBCG759-09	HQ647528	Canada	British Columbia	Spencer Entomological Museum, UBC
Campaea perlata	08-JDWBC-0760	LBCG760-09	HQ647527	Canada	British Columbia	Spencer Entomological Museum, UBC
Campaea perlata	08-JDWBC-0950	LBCG950-09	HQ647526	Canada	British Columbia	Spencer Entomological Museum, UBC
Campaea perlata	08-JDWBC-0974	LBCG974-09	HQ647525	Canada	British Columbia	Spencer Entomological Museum, UBC
Campaea perlata	08-JDWBC-1000	LBCG1000-09	HQ647524	Canada	British Columbia	Spencer Entomological Museum, UBC
Campaea perlata	08-JDWBC-1010	LBCG1010-09	HQ647523	Canada	British Columbia	Spencer Entomological Museum, UBC
Campaea perlata	08-JDWBC-1842	LBCG1842-09	HQ647537	Canada	British Columbia	Spencer Entomological Museum, UBC
Campaea perlata	08-JDWBC-1843	LBCG1843-09	HQ647536	Canada	British Columbia	Spencer Entomological Museum, UBC
Campaea perlata	CBCC1005	GWNS146-07	HQ647519	Canada	Alberta	Strickland Museum of Entomology, University of Alberta
Campaea perlata	CBCC1215	GWNS551-07	HQ647522	Canada	Alberta	Strickland Museum of Entomology, University of Alberta
Campaea perlata	CNCLEP00033177	GWNC178-07	HQ647514	Canada	Yukon Territory	Canadian National Collection of Insects, Arachnids and Nematodes
Campaea perlata	CNCLEP00033178	GWNC179-07	HQ647515	United States	Alaska	Canadian National Collection of Insects, Arachnids and Nematodes
Campaea perlata	ENT002-001606	GWNR235-07	HQ647530	Canada	British Columbia	Royal British Columbia Museum
Campaea perlata	ENT002-001608	GWNR236-07	HQ647529	Canada	British Columbia	Royal British Columbia Museum
Campaea perlata	ENT987-000522	GWNR229-07	HQ647535	Canada	British Columbia	Royal British Columbia Museum
Campaea perlata	ENT991-010357	GWNR230-07	HQ647534	Canada	British Columbia	Royal British Columbia Museum
Campaea perlata	ENT996-003908	GWNR231-07	HQ647533	Canada	British Columbia	Royal British Columbia Museum
Campaea perlata	ENT996-003929	GWNR234-07	HQ647531	United States	Washington	Royal British Columbia Museum
Campaea perlata	ENT996-003930	GWNR232-07	HQ647532	United States	Washington	Royal British Columbia Museum
Campaea perlata	UASM2060	GWNS147-07	HQ647518	Canada	Alberta	Strickland Museum of Entomology, University of Alberta
Campaea perlata	UASM59737	GWNS149-07	HQ647516	Canada	British Columbia	Strickland Museum of Entomology, University of Alberta
Campaea perlata	UASM78393	GWNS148-07	HQ647517	Canada	Alberta	Strickland Museum of Entomology, University of Alberta
Campaea perlata	WFBM-07-0052	GWNW052-07	HQ647521	United States	Idaho	University of Idaho, W. F. Barr Entomological Collection
Campaea perlata	WFBM-07-0053	GWNW053-07	HQ647520	United States	Idaho	University of Idaho, W. F. Barr Entomological Collection
Caripeta aequaliaria	08-JDWBC-3438	LBCG3438-09	HQ647549	Canada	British Columbia	Spencer Entomological Museum, UBC
Caripeta aequaliaria	08-JDWBC-3442	LBCG3442-09	HQ647548	Canada	British Columbia	Spencer Entomological Museum, UBC
Caripeta aequaliaria	08-JDWBC-3704	LBCG3704-09	HQ647547	Canada	British Columbia	Royal British Columbia Museum
Caripeta aequaliaria	08-JDWBC-3706	LBCG3706-09	HQ647546	Canada	British Columbia	Royal British Columbia Museum
Caripeta aequaliaria	08-JDWBC-3708	LBCG3708-09	HQ647545	Canada	British Columbia	Royal British Columbia Museum
Caripeta aequaliaria	08-JDWBC-3709	LBCG3709-09	HQ647544	Canada	British Columbia	Royal British Columbia Museum
Caripeta aequaliaria	08-JDWBC-3719	LBCG3719-09	HQ647543	Canada	British Columbia	Royal British Columbia Museum
Caripeta aequaliaria	Dun-08-060	DUNLP060-08	HQ647538	Canada	British Columbia	Pacific Forestry Centre, Canadian Forest Service
Caripeta aequaliaria	ENT996-004053	GWNR274-07	HQ647540	Canada	British Columbia	Royal British Columbia Museum
Caripeta aequaliaria	ENT996-004056	GWNR273-07	HQ647541	United States	Idaho	Royal British Columbia Museum
Caripeta aequaliaria	ENT996-004069	GWNR272-07	HQ647542	Canada	British Columbia	Royal British Columbia Museum
Caripeta aequaliaria	JECW-07-0085	GWNJ085-07	HQ647539	United States	Washington	James Entomological Collection, Washington State University
Caripeta angustiorata	08-JDWBC-3439	LBCG3439-09	HQ647557	Canada	British Columbia	Spencer Entomological Museum, UBC
Caripeta angustiorata	08-JDWBC-3443	LBCG3443-09	HQ647556	Canada	British Columbia	Spencer Entomological Museum, UBC
Caripeta angustiorata	08-JDWBC-3445	LBCG3445-09	HQ647555	Canada	British Columbia	Spencer Entomological Museum, UBC
Caripeta angustiorata	08-JDWBC-3681	LBCG3681-09	HQ647554	Canada	Alberta	Strickland Museum of Entomology, University of Alberta
Caripeta angustiorata	Dun-08-061	DUNLP061-08	HQ647550	Canada	British Columbia	Pacific Forestry Centre, Canadian Forest Service
Caripeta angustiorata	ENT996-004070	GWNR275-07	HQ647551	Canada	British Columbia	Royal British Columbia Museum
Caripeta angustiorata	UASM24795	GWNS198-07	HQ647552	Canada	Alberta	Strickland Museum of Entomology, University of Alberta
Caripeta angustiorata	UASM43308	GWNS197-07	HQ647553	Canada	Alberta	Strickland Museum of Entomology, University of Alberta
Caripeta divisata	08-JDWBC-0019	LBCG019-08	HQ647563	Canada	British Columbia	Spencer Entomological Museum, UBC
Caripeta divisata	08-JDWBC-3440	LBCG3440-09	HQ647565	Canada	British Columbia	Spencer Entomological Museum, UBC
Caripeta divisata	08-JDWBC-3444	LBCG3444-09	HQ647564	Canada	British Columbia	Spencer Entomological Museum, UBC
Caripeta divisata	Dun-08-062	DUNLP062-08	HQ647558	Canada	British Columbia	Pacific Forestry Centre, Canadian Forest Service
Caripeta divisata	ENT996-003994	GWNR276-07	HQ647562	Canada	British Columbia	Royal British Columbia Museum
Caripeta divisata	ENT996-003995	GWNR278-07	HQ647561	Canada	British Columbia	Royal British Columbia Museum
Caripeta divisata	UASM43303	GWNS196-07	HQ647559	Canada	Alberta	Strickland Museum of Entomology, University of Alberta
Caripeta divisata	UASM78434	GWNS195-07	HQ647560	Canada	Alberta	Strickland Museum of Entomology, University of Alberta
Caripeta sp.	08-JDWBC-0366	LBCG366-08	HQ647567	Canada	British Columbia	Spencer Entomological Museum, UBC
Caripeta sp.	08-JDWBC-0367	LBCG367-08	HQ647566	Canada	British Columbia	Spencer Entomological Museum, UBC
Caripeta sp.	08-JDWBC-1302	LBCG1302-09	HQ647568	Canada	British Columbia	Spencer Entomological Museum, UBC
Carsia sororiata	CNCLEP00033401	GWNC402-07	HQ647570	Canada	Alberta	Canadian National Collection of Insects, Arachnids and Nematodes
Carsia sororiata	CNCLEP00035462	GWNC696-07	HQ647569	United States	Alaska	Canadian National Collection of Insects, Arachnids and Nematodes
Ceratodalia gueneata	CNCLEP00033433	GWNC434-07	HQ647571	Canada	British Columbia	Canadian National Collection of Insects, Arachnids and Nematodes
Ceratodalia gueneata	ENT987-000018	GWNR423-07	HQ647574	Canada	British Columbia	Royal British Columbia Museum
Ceratodalia gueneata	ENT996-004465	GWNR425-07	HQ647573	United States	Washington	Royal British Columbia Museum
Ceratodalia gueneata	ENT996-004479	GWNR422-07	HQ647575	Canada	British Columbia	Royal British Columbia Museum
Ceratodalia gueneata	PFC-2007-0292	GWNP085-07	HQ647572	Canada	British Columbia	Pacific Forestry Centre, Canadian Forest Service
Chlorochlamys triangularis	08-JDWBC-2597	LBCG2597-09	HQ647580	Canada	British Columbia	Spencer Entomological Museum, UBC
Chlorochlamys triangularis	ENT996-007618	GWNR358-07	HQ647578	Canada	British Columbia	Royal British Columbia Museum
Chlorochlamys triangularis	ENT996-007620	GWNR359-07	HQ647577	Canada	British Columbia	Royal British Columbia Museum
Chlorochlamys triangularis	ENT996-007623	GWNR357-07	HQ647579	Canada	British Columbia	Royal British Columbia Museum
Chlorochlamys triangularis	JECW-07-0126	GWNJ126-07	HQ647576	United States	Washington	James Entomological Collection, Washington State University
Chlorosea banksaria	CNCLEP00033241	GWNC242-07	HQ647581	Canada	British Columbia	Canadian National Collection of Insects, Arachnids and Nematodes
Chlorosea banksaria	JECW-07-0116	GWNJ116-07	HQ647583	United States	Washington	James Entomological Collection, Washington State University
Chlorosea banksaria	JECW-07-0117	GWNJ117-07	HQ647582	United States	Washington	James Entomological Collection, Washington State University
Chlorosea nevadaria	CNCLEP00033238	GWNC239-07	HQ647584	Canada	British Columbia	Canadian National Collection of Insects, Arachnids and Nematodes
Chlorosea nevadaria	ENT996-004163	GWNR348-07	HQ647586	Canada	British Columbia	Royal British Columbia Museum
Chlorosea nevadaria	ENT996-004164	GWNR346-07	HQ647587	Canada	British Columbia	Royal British Columbia Museum
Chlorosea nevadaria	ENT996-004166	GWNR345-07	HQ647588	United States	Washington	Royal British Columbia Museum
Chlorosea nevadaria	WFBM-07-0074	GWNW074-07	HQ647585	United States	Idaho	University of Idaho, W. F. Barr Entomological Collection
Cladara atroliturata	08-JDWBC-0290	LBCG290-08	HQ647591	Canada	British Columbia	Spencer Entomological Museum, UBC
Cladara atroliturata	ENT991-010173	GWNR612-07	HQ647593	Canada	British Columbia	Royal British Columbia Museum
Cladara atroliturata	ENT996-005672	GWNR613-07	HQ647592	Canada	British Columbia	Royal British Columbia Museum
Cladara atroliturata	UASM24900	GWNS479-07	HQ647590	Canada	Alberta	Strickland Museum of Entomology, University of Alberta

Cladara atroliturata	UASM59112	GWNS481-07	HQ647589	Canada	Alberta	Strickland Museum of Entomology, University of Alberta
Cladara limitaria	08-JDWBC-0270	LBCG270-08	HQ647598	Canada	British Columbia	Spencer Entomological Museum, UBC
Cladara limitaria	08-JDWBC-0275	LBCG275-08	HQ647599	Canada	British Columbia	Spencer Entomological Museum, UBC
Cladara limitaria	08-JDWBC-0280	LBCG280-08	HQ647600	Canada	British Columbia	Spencer Entomological Museum, UBC
Cladara limitaria	08-JDWBC-0289	LBCG289-08	HQ647601	Canada	British Columbia	Spencer Entomological Museum, UBC
Cladara limitaria	08-JDWBC-0300	LBCG300-08	HQ647596	Canada	British Columbia	Spencer Entomological Museum, UBC
Cladara limitaria	08-JDWBC-0301	LBCG301-08	HQ647597	Canada	British Columbia	Spencer Entomological Museum, UBC
Cladara limitaria	08-JDWBC-0305	LBCG305-08	HQ647602	Canada	British Columbia	Spencer Entomological Museum, UBC
Cladara limitaria	ENT991-010622	GWNR615-07	HQ647604	Canada	British Columbia	Royal British Columbia Museum
Cladara limitaria	ENT991-010627	GWNR616-07	HQ647603	Canada	British Columbia	Royal British Columbia Museum
Cladara limitaria	ENT991-066564	GWNR614-07	HQ647605	Canada	British Columbia	Royal British Columbia Museum
Cladara limitaria	ENT996-005662	GWNR611-07	HQ647606	Canada	British Columbia	Royal British Columbia Museum
Cladara limitaria	ENT996-005668	GWNR609-07	HQ647607	Canada	British Columbia	Royal British Columbia Museum
Cladara limitaria	PFC-2007-0230	GWNP023-07	HQ647595	Canada	British Columbia	Pacific Forestry Centre, Canadian Forest Service
Cladara limitaria	UASM41858	GWNS478-07	HQ647594	Canada	Alberta	Strickland Museum of Entomology, University of Alberta
Colostygia turbata	CNCLEP00033428	GWNC429-07	HQ647609	Canada	British Columbia	Canadian National Collection of Insects, Arachnids and Nematodes
Colostygia turbata	HLC-20441	LBCA441-05	HQ647608	Canada	British Columbia	Biodiversity Institute of Ontario
Colostygia turbata	UASM41706	GWNS312-07	HQ647610	Canada	Alberta	Strickland Museum of Entomology, University of Alberta
Colostygia turbata	UASM95880	GWNS582-07	HQ647611	Canada	Alberta	Strickland Museum of Entomology, University of Alberta
Colostygia turbata	UASM95881	GWNS581-07	HQ647612	Canada	Alberta	Strickland Museum of Entomology, University of Alberta
Coryphista meadii	CBCC1025	GWNS324-07	HQ647615	Canada	Alberta	Strickland Museum of Entomology, University of Alberta
Coryphista meadii	ENT996-004431	GWNR427-07	HQ647619	Canada	British Columbia	Royal British Columbia Museum
Coryphista meadii	ENT996-004438	GWNR429-07	HQ647617	United States	Washington	Royal British Columbia Museum
Coryphista meadii	ENT996-004443	GWNR428-07	HQ647618	Canada	British Columbia	Royal British Columbia Museum
Coryphista meadii	UASM59128	GWNS583-07	HQ647616	Canada	Alberta	Strickland Museum of Entomology, University of Alberta
Coryphista meadii	UASM59730	GWNS323-07	HQ647614	Canada	British Columbia	Strickland Museum of Entomology, University of Alberta
Coryphista meadii	UASM99593	GWNS607-08	HQ647613	Canada	Alberta	Strickland Museum of Entomology, University of Alberta
Costaconvexa centrostrigaria	10-GOBCL-08	GOBCL008-10	HQ647620	Canada	Ontario	Barcode of Life Data System
Cyclophora dataria	ENT996-004281	GWNR386-07	HQ647624	Canada	British Columbia	Royal British Columbia Museum
Cyclophora dataria	ENT996-004283	GWNR387-07	HQ647623	Canada	British Columbia	Royal British Columbia Museum
Cyclophora dataria	ENT996-004300	GWNR385-07	HQ647625	Canada	British Columbia	Royal British Columbia Museum
Cyclophora dataria	PFC-2007-0225	GWNP018-07	HQ647622	Canada	British Columbia	Pacific Forestry Centre, Canadian Forest Service
Cyclophora dataria	PFC-2007-0226	GWNP019-07	HQ647621	Canada	British Columbia	Pacific Forestry Centre, Canadian Forest Service
Cyclophora packardi	JECW-07-0130	GWNJ130-07	HQ647626	United States	Washington	James Entomological Collection, Washington State University
Cyclophora pendulinaria	CNCLEP00033265	GWNC266-07	HQ647627	Canada	British Columbia	Canadian National Collection of Insects, Arachnids and Nematodes
Cyclophora pendulinaria	ENT002-001650	GWNR388-07	HQ647631	Canada	British Columbia	Royal British Columbia Museum
Cyclophora pendulinaria	UASM24652	GWNS248-07	HQ647628	Canada	Alberta	Strickland Museum of Entomology, University of Alberta
Cyclophora pendulinaria	UASM7157	GWNS246-07	HQ647630	Canada	Alberta	Strickland Museum of Entomology, University of Alberta
Cyclophora pendulinaria	UASM78395	GWNS247-07	HQ647629	Canada	Alberta	Strickland Museum of Entomology, University of Alberta
Dasyfidonia avuncularia	CNCLEP00033358	GWNC359-07	HQ647632	Canada	Alberta	Canadian National Collection of Insects, Arachnids and Nematodes
Dasyfidonia avuncularia	JECW-07-0030	GWNJ030-07	HQ647633	Canada	British Columbia	James Entomological Collection, Washington State University
Dasyfidonia avuncularia	UASM24866	GWNS064-07	HQ647635	Canada	Alberta	Strickland Museum of Entomology, University of Alberta
Dasyfidonia avuncularia	UASM24867	GWNS065-07	HQ647634	Canada	Alberta	Strickland Museum of Entomology, University of Alberta
Dichorda rectaria	09-JDWGEO-357	GNAU057-09	HM392360	United States	North Dakota	Smithsonian Institution
Dichorda rectaria	09-JDWGEO-358	GNAU058-09	HM392361	United States	Oklahoma	Smithsonian Institution
Dichorda rectaria	09-JDWGEO-359	GNAU059-09	HM392362	United States	New Mexico	Smithsonian Institution
Digrammia californiaria	08-JDWBC-1087	LBCG1087-09	HQ647640	Canada	British Columbia	Spencer Entomological Museum, UBC
Digrammia californiaria	CNCLEP00033351	GWNC352-07	HQ647636	Canada	Alberta	Canadian National Collection of Insects, Arachnids and Nematodes
Digrammia californiaria	UASM57094	GWNS048-07	HQ647637	Canada	Alberta	Strickland Museum of Entomology, University of Alberta
Digrammia californiaria	UASM57185	GWNS047-07	HQ647638	Canada	Alberta	Strickland Museum of Entomology, University of Alberta
Digrammia californiaria	UASM57355	GWNS046-07	HQ647639	Canada	Alberta	Strickland Museum of Entomology, University of Alberta
Digrammia curvata	08-JDWBC-0125	LBCG125-08	HQ647649	Canada	British Columbia	Spencer Entomological Museum, UBC
Digrammia curvata	08-JDWBC-0131	LBCG131-08	HQ647648	Canada	British Columbia	Spencer Entomological Museum, UBC
Digrammia curvata	08-JDWBC-0427	LBCG427-08	HQ647655	Canada	British Columbia	Spencer Entomological Museum, UBC
Digrammia curvata	08-JDWBC-0429	LBCG429-08	HQ647654	Canada	British Columbia	Spencer Entomological Museum, UBC
Digrammia curvata	08-JDWBC-0430	LBCG430-08	HQ647653	Canada	British Columbia	Spencer Entomological Museum, UBC
Digrammia curvata	08-JDWBC-0491	LBCG491-08	HQ647652	Canada	British Columbia	Spencer Entomological Museum, UBC
Digrammia curvata	08-JDWBC-0499	LBCG499-08	HQ647651	Canada	British Columbia	Spencer Entomological Museum, UBC
Digrammia curvata	08-JDWBC-1108	LBCG1108-09	HQ647650	Canada	British Columbia	Spencer Entomological Museum, UBC
Digrammia curvata	08-JDWBC-1114	LBCG1114-09	HQ647673	Canada	British Columbia	Spencer Entomological Museum, UBC
Digrammia curvata	08-JDWBC-2120	LBCG2120-09	HQ647679	Canada	British Columbia	Spencer Entomological Museum, UBC
Digrammia curvata	08-JDWBC-2122	LBCG2122-09	HQ647678	Canada	British Columbia	Spencer Entomological Museum, UBC
Digrammia curvata	08-JDWBC-2125	LBCG2125-09	HQ647677	Canada	British Columbia	Spencer Entomological Museum, UBC
Digrammia curvata	08-JDWBC-2126	LBCG2126-09	HQ647676	Canada	British Columbia	Spencer Entomological Museum, UBC
Digrammia curvata	08-JDWBC-2127	LBCG2127-09	HQ647675	Canada	British Columbia	Spencer Entomological Museum, UBC
Digrammia curvata	08-JDWBC-2128	LBCG2128-09	HQ647674	Canada	British Columbia	Spencer Entomological Museum, UBC
Digrammia curvata	08-JDWBC-2591	LBCG2591-09	HQ647672	Canada	British Columbia	Spencer Entomological Museum, UBC
Digrammia curvata	08-JDWBC-2592	LBCG2592-09	HQ647671	Canada	British Columbia	Spencer Entomological Museum, UBC
Digrammia curvata	08-JDWBC-2593	LBCG2593-09	HQ647670	Canada	British Columbia	Spencer Entomological Museum, UBC
Digrammia curvata	08-JDWBC-2594	LBCG2594-09	HQ647669	Canada	British Columbia	Spencer Entomological Museum, UBC
Digrammia curvata	08-JDWBC-2595	LBCG2595-09	HQ647668	Canada	British Columbia	Spencer Entomological Museum, UBC
Digrammia curvata	08-JDWBC-2596	LBCG2596-09	HQ647667	Canada	British Columbia	Spencer Entomological Museum, UBC
Digrammia curvata	08-JDWBC-2912	LBCG2912-09	HQ647666	Canada	British Columbia	Spencer Entomological Museum, UBC
Digrammia curvata	08-JDWBC-2913	LBCG2913-09	HQ647665	Canada	British Columbia	Spencer Entomological Museum, UBC
Digrammia curvata	08-JDWBC-2914	LBCG2914-09	HQ647664	Canada	British Columbia	Spencer Entomological Museum, UBC
Digrammia curvata	08-JDWBC-2915	LBCG2915-09	HQ647663	Canada	British Columbia	Spencer Entomological Museum, UBC
Digrammia curvata	08-JDWBC-2916	LBCG2916-09	HQ647662	Canada	British Columbia	Spencer Entomological Museum, UBC
Digrammia curvata	08-JDWBC-2917	LBCG2917-09	HQ647661	Canada	British Columbia	Spencer Entomological Museum, UBC
Digrammia curvata	08-JDWBC-2918	LBCG2918-09	HQ647660	Canada	British Columbia	Spencer Entomological Museum, UBC
Digrammia curvata	08-JDWBC-3274	LBCG3274-09	HQ647659	Canada	British Columbia	Spencer Entomological Museum, UBC
Digrammia curvata	08-JDWBC-3275	LBCG3275-09	HQ647658	Canada	British Columbia	Spencer Entomological Museum, UBC
Digrammia curvata	08-JDWBC-3276	LBCG3276-09	HQ647657	Canada	British Columbia	Spencer Entomological Museum, UBC
Digrammia curvata	08-JDWBC-3308	LBCG3308-09	HQ647656	Canada	British Columbia	Spencer Entomological Museum, UBC
Digrammia curvata	CNCLEP00033069	GWNC070-07	HQ647641	Canada	British Columbia	Canadian National Collection of Insects, Arachnids and Nematodes
Digrammia curvata	ENT996-003358	GWNR045-07	HQ647647	United States	Idaho	Royal British Columbia Museum
Digrammia curvata	JD1944	GWNS043-07	HQ647643	Canada	British Columbia	Strickland Museum of Entomology, University of Alberta
Digrammia curvata	JECW-07-0002	GWNJ002-07	HQ647642	United States	Washington	James Entomological Collection, Washington State University
Digrammia curvata	UASM57118	GWNS042-07	HQ647644	Canada	Alberta	Strickland Museum of Entomology, University of Alberta
Digrammia curvata	UASM57480	GWNS040-07	HQ647646	Canada	Alberta	Strickland Museum of Entomology, University of Alberta
Digrammia curvata	UASM58935	GWNS041-07	HQ647645	Canada	Alberta	Strickland Museum of Entomology, University of Alberta
Digrammia decorata	CNCLEP00033077	GWNC078-07	HQ647681	Canada	British Columbia	Canadian National Collection of Insects, Arachnids and Nematodes
Digrammia decorata	CNCLEP00033352	GWNC353-07	HQ647680	Canada	British Columbia	Canadian National Collection of Insects, Arachnids and Nematodes
Digrammia decorata	CNCLEP00033353	GWNC354-07	HQ647682	Canada	Alberta	Canadian National Collection of Insects, Arachnids and Nematodes
Digrammia decorata	UASM41643	GWNS050-07	HQ647683	Canada	Alberta	Strickland Museum of Entomology, University of Alberta
Digrammia decorata	UASM57200	GWNS049-07	HQ647684	Canada	Alberta	Strickland Museum of Entomology, University of Alberta
Digrammia delectata	08-JDWBC-1137	LBCG1137-09	HQ647690	Canada	British Columbia	Spencer Entomological Museum, UBC
Digrammia delectata	ENT992-004607	GWNR051-07	HQ647687	Canada	British Columbia	Royal British Columbia Museum
Digrammia delectata	ENT996-003280	GWNR041-07	HQ647689	United States	Idaho	Royal British Columbia Museum
Digrammia delectata	ENT996-003284	GWNR042-07	HQ647688	United States	Washington	Royal British Columbia Museum
Digrammia delectata	JECW-07-0015	GWNJ015-07	HQ647686	United States	Washington	James Entomological Collection, Washington State University
Digrammia delectata	JECW-07-0016	GWNJ016-07	HQ647685	United States	Washington	James Entomological Collection, Washington State University
Digrammia denticulata	08-JDWBC-0036	LBCG036-08	HQ647701	Canada	British Columbia	Spencer Entomological Museum, UBC
Digrammia denticulata	08-JDWBC-0038	LBCG038-08	HQ647700	Canada	British Columbia	Spencer Entomological Museum, UBC
Digrammia denticulata	08-JDWBC-0049	LBCG049-08	HQ647699	Canada	British Columbia	Spencer Entomological Museum, UBC
Digrammia denticulata	08-JDWBC-2124	LBCG2124-09	HQ647703	Canada	British Columbia	Spencer Entomological Museum, UBC
Digrammia denticulata	08-JDWBC-2590	LBCG2590-09	HQ647702	Canada	British Columbia	Spencer Entomological Museum, UBC
Digrammia denticulata	CNCLEP00033346	GWNC347-07	HQ647691	Canada	British Columbia	Canadian National Collection of Insects, Arachnids and Nematodes

Digrammia denticulata	ENT002-001541	GWNR053-07	HQ647697	Canada	British Columbia	Royal British Columbia Museum
Digrammia denticulata	ENT996-003288	GWNR071-07	HQ647696	Canada	British Columbia	Royal British Columbia Museum
Digrammia denticulata	ENT996-003386	GWNR052-07	HQ647698	Canada	British Columbia	Royal British Columbia Museum
Digrammia denticulata	JD0205	GWNS510-07	HQ647695	Canada	Alberta	Strickland Museum of Entomology, University of Alberta
Digrammia denticulata	JECW-07-0017	GWNJ017-07	HQ647692	United States	Washington	James Entomological Collection, Washington State University
Digrammia denticulata	UASM57479	GWNS045-07	HQ647693	Canada	Alberta	Strickland Museum of Entomology, University of Alberta
Digrammia denticulata	UASM58855	GWNS044-07	HQ647694	Canada	Alberta	Strickland Museum of Entomology, University of Alberta
Digrammia irrorata	CNCLEP00033354	GWNC355-07	HQ647705	Canada	Alberta	Canadian National Collection of Insects, Arachnids and Nematodes
Digrammia irrorata	CNCLEP00033355	GWNC356-07	HQ647704	Canada	Alberta	Canadian National Collection of Insects, Arachnids and Nematodes
Digrammia irrorata	JECW-07-0019	GWNJ019-07	HQ647706	United States	Washington	James Entomological Collection, Washington State University
Digrammia irrorata	UASM57117	GWNS055-07	HQ647707	Canada	Alberta	Strickland Museum of Entomology, University of Alberta
Digrammia irrorata	UASM57267	GWNS054-07	HQ647708	Canada	Alberta	Strickland Museum of Entomology, University of Alberta
Digrammia irrorata	WFBM-07-0009	GWNW009-07	HQ647710	United States	Idaho	University of Idaho, W. F. Barr Entomological Collection
Digrammia irrorata	WFBM-07-0010	GWNW010-07	HQ647709	United States	Idaho	University of Idaho, W. F. Barr Entomological Collection
Digrammia mellistrigata	CNCLEP00033356	GWNC357-07	HQ647711	Canada	Alberta	Canadian National Collection of Insects, Arachnids and Nematodes
Digrammia muscariata	PFC-2007-0350	GWNP140-07	HQ647714	Canada	British Columbia	Pacific Forestry Centre, Canadian Forest Service
Digrammia muscariata	PFC-2007-0351	GWNP141-07	HQ647713	Canada	British Columbia	Pacific Forestry Centre, Canadian Forest Service
Digrammia muscariata	PFC-2007-0352	GWNP142-07	HQ647712	Canada	British Columbia	Pacific Forestry Centre, Canadian Forest Service
Digrammia neptaria	08-JDWBC-0046	LBCG046-08	HQ647722	Canada	British Columbia	Spencer Entomological Museum, UBC
Digrammia neptaria	08-JDWBC-0047	LBCG047-08	HQ647721	Canada	British Columbia	Spencer Entomological Museum, UBC
Digrammia neptaria	08-JDWBC-0048	LBCG048-08	HQ647720	Canada	British Columbia	Spencer Entomological Museum, UBC
Digrammia neptaria	08-JDWBC-1071	LBCG1071-09	HQ647723	Canada	British Columbia	Spencer Entomological Museum, UBC
Digrammia neptaria	CNCLEP00033080	GWNC081-07	HQ647715	Canada	British Columbia	Canadian National Collection of Insects, Arachnids and Nematodes
Digrammia neptaria	ENT991-126956	GWNR057-07	HQ647719	Canada	British Columbia	Royal British Columbia Museum
Digrammia neptaria	UASM58268	GWNS057-07	HQ647717	Canada	Alberta	Strickland Museum of Entomology, University of Alberta
Digrammia neptaria	UASM77934	GWNS058-07	HQ647716	Canada	Alberta	Strickland Museum of Entomology, University of Alberta
Digrammia neptaria	UASM95799	GWNS056-07	HQ647718	Canada	Alberta	Strickland Museum of Entomology, University of Alberta
Digrammia nubiculata	JECW-07-0020	GWNJ020-07	HQ647726	United States	Washington	James Entomological Collection, Washington State University
Digrammia nubiculata	JECW-07-0021	GWNJ021-07	HQ647725	United States	Washington	James Entomological Collection, Washington State University
Digrammia nubiculata	JECW-07-0022	GWNJ022-07	HQ647724	United States	Washington	James Entomological Collection, Washington State University
Digrammia nubiculata	WFBM-07-0011	GWNW011-07	HQ647727	United States	Idaho	University of Idaho, W. F. Barr Entomological Collection
Digrammia ordinata	08-JDWBC-1096	LBCG1096-09	HQ647732	Canada	British Columbia	Spencer Entomological Museum, UBC
Digrammia ordinata	08-JDWBC-1110	LBCG1110-09	HQ647731	Canada	British Columbia	Spencer Entomological Museum, UBC
Digrammia ordinata	CNCLEP00033065	GWNC066-07	HQ647730	Canada	British Columbia	Canadian National Collection of Insects, Arachnids and Nematodes
Digrammia ordinata	CNCLEP00033066	GWNC067-07	HQ647729	Canada	British Columbia	Canadian National Collection of Insects, Arachnids and Nematodes
Digrammia ordinata	CNCLEP00033347	GWNC348-07	HQ647728	Canada	British Columbia	Canadian National Collection of Insects, Arachnids and Nematodes
Digrammia respersata	08-JDWBC-1085	LBCG1085-09	HQ647739	Canada	British Columbia	Spencer Entomological Museum, UBC
Digrammia respersata	CNCLEP00033075	GWNC076-07	HQ647735	Canada	British Columbia	Canadian National Collection of Insects, Arachnids and Nematodes
Digrammia respersata	CNCLEP00033348	GWNC349-07	HQ647734	Canada	British Columbia	Canadian National Collection of Insects, Arachnids and Nematodes
Digrammia respersata	CNCLEP00033349	GWNC350-07	HQ647733	Canada	British Columbia	Canadian National Collection of Insects, Arachnids and Nematodes
Digrammia respersata	ENT996-003390	GWNR060-07	HQ647738	Canada	British Columbia	Royal British Columbia Museum
Digrammia respersata	JECW-07-0023	GWNJ023-07	HQ647737	United States	Washington	James Entomological Collection, Washington State University
Digrammia respersata	JECW-07-0024	GWNJ024-07	HQ647736	United States	Washington	James Entomological Collection, Washington State University
Digrammia rippertaria	ENT002-001542	GWNR054-07	HQ647743	Canada	British Columbia	Royal British Columbia Museum
Digrammia rippertaria	NFRC-P-2007-100012	GWNN012-07	HQ647742	Canada	Alberta	Northern Forestry Centre, Canadian Forest Service
Digrammia rippertaria	UASM53012	GWNS051-07	HQ647741	Canada	Alberta	Strickland Museum of Entomology, University of Alberta
Digrammia rippertaria	UASM58890	GWNS053-07	HQ647740	Canada	Alberta	Strickland Museum of Entomology, University of Alberta
Digrammia rippertaria	UASM78402	GWNS558-07	HQ647745	Canada	Alberta	Strickland Museum of Entomology, University of Alberta
Digrammia rippertaria	UASM78403	GWNS559-07	HQ647744	Canada	Alberta	Strickland Museum of Entomology, University of Alberta
Digrammia rippertaria	UASM78404	GWNS557-07	HQ647746	Canada	Alberta	Strickland Museum of Entomology, University of Alberta
Digrammia rippertaria	UASM95861	GWNS526-07	HQ647748	Canada	Alberta	Strickland Museum of Entomology, University of Alberta
Digrammia rippertaria	UASM95871	GWNS542-07	HQ647747	Canada	Alberta	Strickland Museum of Entomology, University of Alberta
Digrammia setonana	08-JDWBC-0361	LBCG361-08	HQ647752	Canada	British Columbia	Spencer Entomological Museum, UBC
Digrammia setonana	08-JDWBC-1303	LBCG1303-09	HQ647753	Canada	British Columbia	Spencer Entomological Museum, UBC
Digrammia setonana	08-JDWBC-2119	LBCG2119-09	HQ647757	Canada	British Columbia	Spencer Entomological Museum, UBC
Digrammia setonana	08-JDWBC-2121	LBCG2121-09	HQ647756	Canada	British Columbia	Spencer Entomological Museum, UBC
Digrammia setonana	08-JDWBC-2123	LBCG2123-09	HQ647755	Canada	British Columbia	Spencer Entomological Museum, UBC
Digrammia setonana	08-JDWBC-2589	LBCG2589-09	HQ647754	Canada	British Columbia	Spencer Entomological Museum, UBC
Digrammia setonana	Dun-08-063	DUNLP063-08	HQ647749	Canada	British Columbia	Pacific Forestry Centre, Canadian Forest Service
Digrammia setonana	ENT996-003346	GWNR064-07	HQ647751	Canada	British Columbia	Royal British Columbia Museum
Digrammia setonana	PFC-2007-0353	GWNP143-07	HQ647750	Canada	British Columbia	Pacific Forestry Centre, Canadian Forest Service
Digrammia subminiata	CNCLEP00033357	GWNC358-07	HQ647758	Canada	Alberta	Canadian National Collection of Insects, Arachnids and Nematodes
Digrammia subminiata	JECW-07-0027	GWNJ027-07	HQ647760	United States	Washington	James Entomological Collection, Washington State University
Digrammia subminiata	JECW-07-0028	GWNJ028-07	HQ647759	United States	Washington	James Entomological Collection, Washington State University
Digrammia subminiata	UASM57247	GWNS061-07	HQ647761	Canada	Alberta	Strickland Museum of Entomology, University of Alberta
Digrammia subminiata	UASM58490	GWNS060-07	HQ647762	Canada	Alberta	Strickland Museum of Entomology, University of Alberta
Digrammia subminiata	UASM77972	GWNS059-07	HQ647763	Canada	Alberta	Strickland Museum of Entomology, University of Alberta
Digrammia subminiata	WFBM-07-0014	GWNW014-07	HQ647764	United States	Idaho	University of Idaho, W. F. Barr Entomological Collection
Digrammia triviata	08-JDWBC-0380	LBCG380-08	HQ647767	Canada	British Columbia	Spencer Entomological Museum, UBC
Digrammia triviata	08-JDWBC-2129	LBCG2129-09	HQ647775	Canada	British Columbia	Spencer Entomological Museum, UBC
Digrammia triviata	08-JDWBC-2130	LBCG2130-09	HQ647774	Canada	British Columbia	Spencer Entomological Museum, UBC
Digrammia triviata	08-JDWBC-2131	LBCG2131-09	HQ647773	Canada	British Columbia	Spencer Entomological Museum, UBC
Digrammia triviata	08-JDWBC-2132	LBCG2132-09	HQ647772	Canada	British Columbia	Spencer Entomological Museum, UBC
Digrammia triviata	08-JDWBC-2134	LBCG2134-09	HQ647771	Canada	British Columbia	Spencer Entomological Museum, UBC
Digrammia triviata	08-JDWBC-2135	LBCG2135-09	HQ647770	Canada	British Columbia	Spencer Entomological Museum, UBC
Digrammia triviata	08-JDWBC-2136	LBCG2136-09	HQ647769	Canada	British Columbia	Spencer Entomological Museum, UBC
Digrammia triviata	08-JDWBC-2137	LBCG2137-09	HQ647768	Canada	British Columbia	Spencer Entomological Museum, UBC
Digrammia triviata	CNCLEP00033350	GWNC351-07	HQ647765	Canada	British Columbia	Canadian National Collection of Insects, Arachnids and Nematodes
Digrammia triviata	Dun-08-064	DUNLP064-08	HQ647766	Canada	British Columbia	Pacific Forestry Centre, Canadian Forest Service
Drepanulatrix bifilata	WFBM-07-0037	GWNW037-07	HQ647776	United States	Idaho	University of Idaho, W. F. Barr Entomological Collection
Drepanulatrix carnearia	UASM59634	GWNS108-07	HQ647777	Canada	Alberta	Strickland Museum of Entomology, University of Alberta
Drepanulatrix carnearia	WFBM-07-0038	GWNW038-07	HQ647778	United States	Idaho	University of Idaho, W. F. Barr Entomological Collection
Drepanulatrix falcataria	08-JDWBC-1060	LBCG1060-09	HQ647785	Canada	British Columbia	Spencer Entomological Museum, UBC
Drepanulatrix falcataria	08-JDWBC-1061	LBCG1061-09	HQ647784	Canada	British Columbia	Spencer Entomological Museum, UBC
Drepanulatrix falcataria	08-JDWBC-1063	LBCG1063-09	HQ647783	Canada	British Columbia	Spencer Entomological Museum, UBC
Drepanulatrix falcataria	08-JDWBC-1079	LBCG1079-09	HQ647782	Canada	British Columbia	Spencer Entomological Museum, UBC
Drepanulatrix falcataria	08-JDWBC-1088	LBCG1088-09	HQ647781	Canada	British Columbia	Spencer Entomological Museum, UBC
Drepanulatrix falcataria	CNCLEP00033147	GWNC148-07	HQ647779	Canada	British Columbia	Canadian National Collection of Insects, Arachnids and Nematodes
Drepanulatrix falcataria	ENT996-003748	GWNR160-07	HQ647786	Canada	British Columbia	Royal British Columbia Museum
Drepanulatrix falcataria	ENT996-003808	GWNR159-07	HQ647787	Canada	British Columbia	Royal British Columbia Museum
Drepanulatrix falcataria	JECW-07-0057	GWNJ057-07	HQ647780	United States	Washington	James Entomological Collection, Washington State University
Drepanulatrix foeminaria	CNCLEP00033145	GWNC146-07	HQ647788	Canada	British Columbia	Canadian National Collection of Insects, Arachnids and Nematodes
Drepanulatrix foeminaria	JECW-07-0059	GWNJ059-07	HQ647789	United States	Washington	James Entomological Collection, Washington State University
Drepanulatrix foeminaria	WFBM-07-0039	GWNW039-07	HQ647790	United States	Washington	University of Idaho, W. F. Barr Entomological Collection
Drepanulatrix foeminaria	WFBM-07-0041	GWNW041-07	HQ647791	United States	Idaho	University of Idaho, W. F. Barr Entomological Collection
Drepanulatrix quadraria	JECW-07-0061	GWNJ061-07	HQ647792	United States	Washington	James Entomological Collection, Washington State University
Drepanulatrix secundaria	CNCLEP00033148	GWNC149-07	HQ647793	Canada	British Columbia	Canadian National Collection of Insects, Arachnids and Nematodes
Drepanulatrix secundaria	ENT996-003723	GWNR165-07	HQ647796	Canada	British Columbia	Royal British Columbia Museum
Drepanulatrix secundaria	JECW-07-0058	GWNJ058-07	HQ647794	United States	Idaho	James Entomological Collection, Washington State University
Drepanulatrix secundaria	SEM-UBC-GEO-0146	GWNU087-07	HQ647795	Canada	British Columbia	Spencer Entomological Museum, UBC
Drepanulatrix unicalcararia	08-JDWBC-1080	LBCG1080-09	HQ647801	Canada	British Columbia	Spencer Entomological Museum, UBC
Drepanulatrix unicalcararia	08-JDWBC-1082	LBCG1082-09	HQ647800	Canada	British Columbia	Spencer Entomological Museum, UBC
Drepanulatrix unicalcararia	CNCLEP00033141	GWNC142-07	HQ647797	Canada	British Columbia	Canadian National Collection of Insects, Arachnids and Nematodes
Drepanulatrix unicalcararia	ENT996-003771	GWNR171-07	HQ647802	Canada	British Columbia	Royal British Columbia Museum
Drepanulatrix unicalcararia	JECW-07-0064	GWNJ064-07	HQ647798	United States	Idaho	James Entomological Collection, Washington State University
Drepanulatrix unicalcararia	WFBM-07-0042	GWNW042-07	HQ647799	United States	Idaho	University of Idaho, W. F. Barr Entomological Collection
Dysstroma brunneata	08-JDWBC-1337	LBCG1337-09	HQ647808	Canada	British Columbia	Spencer Entomological Museum, UBC

Dysstroma brunneata	08-JDWBC-1339	LBCG1339-09	HQ647807	Canada	British Columbia	Spencer Entomological Museum, UBC
Dysstroma brunneata	CNCLEP00033380	GWNC381-07	HQ647803	Canada	Alberta	Canadian National Collection of Insects, Arachnids and Nematodes
Dysstroma brunneata	ENT996-007670	GWNR622-07	HQ647809	Canada	British Columbia	Royal British Columbia Museum
Dysstroma brunneata	JD1796	GWNS508-07	HQ647804	Canada	British Columbia	Strickland Museum of Entomology, University of Alberta
Dysstroma brunneata	NFRC-P-2007-100047	GWNN047-07	HQ647806	Canada	Alberta	Northern Forestry Centre, Canadian Forest Service
Dysstroma brunneata	PFC-2007-0290	GWNP083-07	HQ647805	Canada	British Columbia	Pacific Forestry Centre, Canadian Forest Service
Dysstroma citrata	08-JDWBC-2330	LBCG2330-09	HQ647815	Canada	British Columbia	Spencer Entomological Museum, UBC
Dysstroma citrata	CNCLEP00033285	GWNC286-07	HQ647810	Canada	Yukon Territory	Canadian National Collection of Insects, Arachnids and Nematodes
Dysstroma citrata	UASM7160	GWNS291-07	HQ647812	Canada	Alberta	Strickland Museum of Entomology, University of Alberta
Dysstroma citrata	UASM7291	GWNS292-07	HQ647811	Canada	Alberta	Strickland Museum of Entomology, University of Alberta
Dysstroma citrata	UASM78399	GWNS571-07	HQ647813	Canada	Alberta	Strickland Museum of Entomology, University of Alberta
Dysstroma citrata	UASM78400	GWNS570-07	HQ647814	Canada	Alberta	Strickland Museum of Entomology, University of Alberta
Dysstroma colvillei	09-JDWGEO-475	GNAU175-10	HM392447	United States	California	Smithsonian Institution
Dysstroma colvillei	09-JDWGEO-476	GNAU176-10	HM392448	United States	California	Smithsonian Institution
Dysstroma colvillei	09-JDWGEO-477	GNAU177-10	HM392449	United States	California	Smithsonian Institution
Dysstroma colvillei	09-JDWGEO-478	GNAU178-10	HM392450	United States	California	Smithsonian Institution
Dysstroma formosa	08-JDWBC-0130	LBCG130-08	HQ647818	Canada	British Columbia	Spencer Entomological Museum, UBC
Dysstroma formosa	08-JDWBC-1336	LBCG1336-09	HQ647819	Canada	British Columbia	Spencer Entomological Museum, UBC
Dysstroma formosa	ENT992-004604	GWNR435-07	HQ647817	Canada	British Columbia	Royal British Columbia Museum
Dysstroma formosa	JECW-07-0133	GWNJ133-07	HQ647816	United States	Washington	James Entomological Collection, Washington State University
Dysstroma hersiliata	ENT996-004641	GWNR437-07	HQ647824	Canada	British Columbia	Royal British Columbia Museum
Dysstroma hersiliata	ENT996-007662	GWNR625-07	HQ647825	Canada	British Columbia	Royal British Columbia Museum
Dysstroma hersiliata	NFRC-P-2007-100050	GWNN050-07	HQ647821	Canada	Alberta	Northern Forestry Centre, Canadian Forest Service
Dysstroma hersiliata	NFRC-P-2007-100051	GWNN051-07	HQ647820	Canada	Alberta	Northern Forestry Centre, Canadian Forest Service
Dysstroma hersiliata	UASM34966	GWNS293-07	HQ647822	Canada	Alberta	Strickland Museum of Entomology, University of Alberta
Dysstroma hersiliata	UASM95863	GWNS524-07	HQ647823	Canada	Alberta	Strickland Museum of Entomology, University of Alberta
Dysstroma infuscata	CNCLEP00033300	GWNC301-07	HQ647827	Canada	Yukon Territory	Canadian National Collection of Insects, Arachnids and Nematodes
Dysstroma infuscata	CNCLEP00033301	GWNC302-07	HQ647826	Canada	Yukon Territory	Canadian National Collection of Insects, Arachnids and Nematodes
Dysstroma infuscata	UASM24173	GWNS288-07	HQ647828	Canada	Alberta	Strickland Museum of Entomology, University of Alberta
Dysstroma infuscata	UASM78397	GWNS573-07	HQ647829	Canada	Alberta	Strickland Museum of Entomology, University of Alberta
Dysstroma mancipata	JECW-07-0134	GWNJ134-07	HQ647830	United States	California	James Entomological Collection, Washington State University
Dysstroma ochrofuscaria	ENT996-004696	GWNR439-07	HQ647831	United States	California	Royal British Columbia Museum
Dysstroma rutlandia	CNCLEP00033378	GWNC379-07	HQ647833	Canada	Alberta	Canadian National Collection of Insects, Arachnids and Nematodes
Dysstroma rutlandia	CNCLEP00033379	GWNC380-07	HQ647832	Canada	Alberta	Canadian National Collection of Insects, Arachnids and Nematodes
Dysstroma sobria	ENT987-001238	GWNR441-07	HQ647835	Canada	British Columbia	Royal British Columbia Museum
Dysstroma sobria	ENT991-131360	GWNR440-07	HQ647836	Canada	British Columbia	Royal British Columbia Museum
Dysstroma sobria	UASM59742	GWNS286-07	HQ647834	Canada	British Columbia	Strickland Museum of Entomology, University of Alberta
Dysstroma suspectata	CNCLEP00033290	GWNC291-07	HQ647837	Canada	Yukon Territory	Canadian National Collection of Insects, Arachnids and Nematodes
Dysstroma suspectata	CNCLEP00033377	GWNC378-07	HQ647838	Canada	Alberta	Canadian National Collection of Insects, Arachnids and Nematodes
Dysstroma suspectata	UASM24171	GWNS290-07	HQ647839	Canada	Alberta	Strickland Museum of Entomology, University of Alberta
Dysstroma suspectata	UASM24172	GWNS287-07	HQ647840	Canada	Alberta	Strickland Museum of Entomology, University of Alberta
Dysstroma truncata	08-JDWBC-0758	LBCG758-09	HQ647863	Canada	British Columbia	Spencer Entomological Museum, UBC
Dysstroma truncata	08-JDWBC-0769	LBCG769-09	HQ647862	Canada	British Columbia	Spencer Entomological Museum, UBC
Dysstroma truncata	08-JDWBC-0770	LBCG770-09	HQ647861	Canada	British Columbia	Spencer Entomological Museum, UBC
Dysstroma truncata	08-JDWBC-0772	LBCG772-09	HQ647860	Canada	British Columbia	Spencer Entomological Museum, UBC
Dysstroma truncata	08-JDWBC-0784	LBCG784-09	HQ647859	Canada	British Columbia	Spencer Entomological Museum, UBC
Dysstroma truncata	08-JDWBC-0801	LBCG801-09	HQ647858	Canada	British Columbia	Spencer Entomological Museum, UBC
Dysstroma truncata	08-JDWBC-0802	LBCG802-09	HQ647857	Canada	British Columbia	Spencer Entomological Museum, UBC
Dysstroma truncata	08-JDWBC-0976	LBCG976-09	HQ647856	Canada	British Columbia	Spencer Entomological Museum, UBC
Dysstroma truncata	08-JDWBC-0991	LBCG991-09	HQ647855	Canada	British Columbia	Spencer Entomological Museum, UBC
Dysstroma truncata	08-JDWBC-1828	LBCG1828-09	HQ647865	Canada	British Columbia	Spencer Entomological Museum, UBC
Dysstroma truncata	08-JDWBC-2318	LBCG2318-09	HQ647864	Canada	British Columbia	Spencer Entomological Museum, UBC
Dysstroma truncata	CNCLEP00033303	GWNC304-07	HQ647842	Canada	British Columbia	Canadian National Collection of Insects, Arachnids and Nematodes
Dysstroma truncata	CNCLEP00033304	GWNC305-07	HQ647841	Canada	British Columbia	Canadian National Collection of Insects, Arachnids and Nematodes
Dysstroma truncata	ENT002-001671	GWNR434-07	HQ647853	Canada	British Columbia	Royal British Columbia Museum
Dysstroma truncata	ENT991-104651	GWNR443-07	HQ647851	Canada	British Columbia	Royal British Columbia Museum
Dysstroma truncata	ENT992-021164	GWNR479-07	HQ647849	Canada	British Columbia	Royal British Columbia Museum
Dysstroma truncata	ENT996-004692	GWNR431-07	HQ647854	United States	Washington	Royal British Columbia Museum
Dysstroma truncata	ENT996-004774	GWNR444-07	HQ647850	Canada	British Columbia	Royal British Columbia Museum
Dysstroma truncata	ENT996-004779	GWNR442-07	HQ647852	Canada	British Columbia	Royal British Columbia Museum
Dysstroma truncata	JD1901	GWNS509-07	HQ647843	Canada	British Columbia	Strickland Museum of Entomology, University of Alberta
Dysstroma truncata	PFC-2007-0288	GWNP081-07	HQ647846	Canada	British Columbia	Pacific Forestry Centre, Canadian Forest Service
Dysstroma truncata	PFC-2007-0289	GWNP082-07	HQ647845	Canada	British Columbia	Pacific Forestry Centre, Canadian Forest Service
Dysstroma truncata	UASM7292	GWNS283-07	HQ647848	Canada	Alberta	Strickland Museum of Entomology, University of Alberta
Dysstroma truncata	UASM95811	GWNS284-07	HQ647847	Canada	Alberta	Strickland Museum of Entomology, University of Alberta
Dysstroma truncata	UASM95858	GWNS519-07	HQ647844	Canada	Alberta	Strickland Museum of Entomology, University of Alberta
Dysstroma walkerata	08-JDWBC-0740	LBCG740-09	HQ647896	Canada	British Columbia	Spencer Entomological Museum, UBC
Dysstroma walkerata	08-JDWBC-0742	LBCG742-09	HQ647895	Canada	British Columbia	Spencer Entomological Museum, UBC
Dysstroma walkerata	08-JDWBC-0744	LBCG744-09	HQ647894	Canada	British Columbia	Spencer Entomological Museum, UBC
Dysstroma walkerata	08-JDWBC-0749	LBCG749-09	HQ647893	Canada	British Columbia	Spencer Entomological Museum, UBC
Dysstroma walkerata	08-JDWBC-0750	LBCG750-09	HQ647892	Canada	British Columbia	Spencer Entomological Museum, UBC
Dysstroma walkerata	08-JDWBC-0751	LBCG751-09	HQ647891	Canada	British Columbia	Spencer Entomological Museum, UBC
Dysstroma walkerata	08-JDWBC-0752	LBCG752-09	HQ647890	Canada	British Columbia	Spencer Entomological Museum, UBC
Dysstroma walkerata	08-JDWBC-0753	LBCG753-09	HQ647889	Canada	British Columbia	Spencer Entomological Museum, UBC
Dysstroma walkerata	08-JDWBC-0776	LBCG776-09	HQ647888	Canada	British Columbia	Spencer Entomological Museum, UBC
Dysstroma walkerata	08-JDWBC-0782	LBCG782-09	HQ647887	Canada	British Columbia	Spencer Entomological Museum, UBC
Dysstroma walkerata	08-JDWBC-0783	LBCG783-09	HQ647886	Canada	British Columbia	Spencer Entomological Museum, UBC
Dysstroma walkerata	08-JDWBC-0785	LBCG785-09	HQ647885	Canada	British Columbia	Spencer Entomological Museum, UBC
Dysstroma walkerata	08-JDWBC-0786	LBCG786-09	HQ647884	Canada	British Columbia	Spencer Entomological Museum, UBC
Dysstroma walkerata	08-JDWBC-0787	LBCG787-09	HQ647883	Canada	British Columbia	Spencer Entomological Museum, UBC
Dysstroma walkerata	08-JDWBC-0788	LBCG788-09	HQ647882	Canada	British Columbia	Spencer Entomological Museum, UBC
Dysstroma walkerata	08-JDWBC-0789	LBCG789-09	HQ647881	Canada	British Columbia	Spencer Entomological Museum, UBC
Dysstroma walkerata	08-JDWBC-0790	LBCG790-09	HQ647880	Canada	British Columbia	Spencer Entomological Museum, UBC
Dysstroma walkerata	08-JDWBC-0791	LBCG791-09	HQ647879	Canada	British Columbia	Spencer Entomological Museum, UBC
Dysstroma walkerata	08-JDWBC-0792	LBCG792-09	HQ647878	Canada	British Columbia	Spencer Entomological Museum, UBC
Dysstroma walkerata	08-JDWBC-0794	LBCG794-09	HQ647877	Canada	British Columbia	Spencer Entomological Museum, UBC
Dysstroma walkerata	08-JDWBC-0800	LBCG800-09	HQ647876	Canada	British Columbia	Spencer Entomological Museum, UBC
Dysstroma walkerata	08-JDWBC-0975	LBCG975-09	HQ647875	Canada	British Columbia	Spencer Entomological Museum, UBC
Dysstroma walkerata	08-JDWBC-0993	LBCG993-09	HQ647874	Canada	British Columbia	Spencer Entomological Museum, UBC
Dysstroma walkerata	08-JDWBC-1007	LBCG1007-09	HQ647873	Canada	British Columbia	Spencer Entomological Museum, UBC
Dysstroma walkerata	08-JDWBC-1089	LBCG1089-09	HQ647872	Canada	British Columbia	Spencer Entomological Museum, UBC
Dysstroma walkerata	08-JDWBC-1823	LBCG1823-09	HQ647911	Canada	British Columbia	Spencer Entomological Museum, UBC
Dysstroma walkerata	08-JDWBC-1824	LBCG1824-09	HQ647910	Canada	British Columbia	Spencer Entomological Museum, UBC
Dysstroma walkerata	08-JDWBC-1825	LBCG1825-09	HQ647909	Canada	British Columbia	Spencer Entomological Museum, UBC
Dysstroma walkerata	08-JDWBC-1826	LBCG1826-09	HQ647908	Canada	British Columbia	Spencer Entomological Museum, UBC
Dysstroma walkerata	08-JDWBC-1827	LBCG1827-09	HQ647907	Canada	British Columbia	Spencer Entomological Museum, UBC
Dysstroma walkerata	08-JDWBC-1829	LBCG1829-09	HQ647906	Canada	British Columbia	Spencer Entomological Museum, UBC
Dysstroma walkerata	08-JDWBC-1830	LBCG1830-09	HQ647905	Canada	British Columbia	Spencer Entomological Museum, UBC
Dysstroma walkerata	08-JDWBC-1831	LBCG1831-09	HQ647904	Canada	British Columbia	Spencer Entomological Museum, UBC
Dysstroma walkerata	08-JDWBC-1832	LBCG1832-09	HQ647903	Canada	British Columbia	Spencer Entomological Museum, UBC
Dysstroma walkerata	08-JDWBC-1833	LBCG1833-09	HQ647902	Canada	British Columbia	Spencer Entomological Museum, UBC
Dysstroma walkerata	08-JDWBC-1834	LBCG1834-09	HQ647901	Canada	British Columbia	Spencer Entomological Museum, UBC
Dysstroma walkerata	08-JDWBC-1837	LBCG1837-09	HQ647900	Canada	British Columbia	Spencer Entomological Museum, UBC
Dysstroma walkerata	08-JDWBC-1838	LBCG1838-09	HQ647899	Canada	British Columbia	Spencer Entomological Museum, UBC
Dysstroma walkerata	08-JDWBC-1839	LBCG1839-09	HQ647898	Canada	British Columbia	Spencer Entomological Museum, UBC
Dysstroma walkerata	08-JDWBC-1840	LBCG1840-09	HQ647897	Canada	British Columbia	Spencer Entomological Museum, UBC
Dysstroma walkerata	CNCLEP00033302	GWNC303-07	HQ647867	Canada	British Columbia	Canadian National Collection of Insects, Arachnids and Nematodes

Dysstroma walkerata	CNCLEP00033305	GWNC306-07	HQ647866	Canada	British Columbia	Canadian National Collection of Insects, Arachnids and Nematodes
Dysstroma walkerata	ENT991-010337	GWNR446-07	HQ647871	Canada	British Columbia	Royal British Columbia Museum
Dysstroma walkerata	ENT996-004633	GWNR447-07	HQ647870	Canada	British Columbia	Royal British Columbia Museum
Dysstroma walkerata	UASM58659	GWNS289-07	HQ647868	Canada	Alberta	Strickland Museum of Entomology, University of Alberta
Dysstroma walkerata	UASM78398	GWNS572-07	HQ647869	Canada	Alberta	Strickland Museum of Entomology, University of Alberta
Ecliptopera silaceata	ENT002-002434	GWNR449-07	HQ647917	Canada	British Columbia	Royal British Columbia Museum
Ecliptopera silaceata	ENT992-020764	GWNR450-07	HQ647916	Canada	British Columbia	Royal British Columbia Museum
Ecliptopera silaceata	ENT996-004425	GWNR451-07	HQ647915	United States	Washington	Royal British Columbia Museum
Ecliptopera silaceata	UASM78421	GWNS568-07	HQ647913	Canada	Alberta	Strickland Museum of Entomology, University of Alberta
Ecliptopera silaceata	UASM78422	GWNS567-07	HQ647914	Canada	Alberta	Strickland Museum of Entomology, University of Alberta
Ecliptopera silaceata	UASM78426	GWNS569-07	HQ647912	Canada	Alberta	Strickland Museum of Entomology, University of Alberta
Ectropis crepuscularia	07-JDWBC-0023	GWND016-07	HQ647923	Canada	British Columbia	Royal British Columbia Museum
Ectropis crepuscularia	07-JDWBC-0024	GWND017-07	HQ647922	Canada	British Columbia	Royal British Columbia Museum
Ectropis crepuscularia	07-JDWBC-0040	GWND032-07	HQ647921	Canada	British Columbia	Royal British Columbia Museum
Ectropis crepuscularia	07-JDWBC-0047	GWND039-07	HQ647920	Canada	British Columbia	Royal British Columbia Museum
Ectropis crepuscularia	07-JDWBC-0050	GWND042-07	HQ647919	Canada	British Columbia	Royal British Columbia Museum
Ectropis crepuscularia	08-JDWBC-0035	LBCG035-08	HQ647929	Canada	British Columbia	Spencer Entomological Museum, UBC
Ectropis crepuscularia	08-JDWBC-0269	LBCG269-08	HQ647930	Canada	British Columbia	Spencer Entomological Museum, UBC
Ectropis crepuscularia	08-JDWBC-0281	LBCG281-08	HQ647931	Canada	British Columbia	Spencer Entomological Museum, UBC
Ectropis crepuscularia	08-JDWBC-0288	LBCG288-08	HQ647932	Canada	British Columbia	Spencer Entomological Museum, UBC
Ectropis crepuscularia	08-JDWBC-0292	LBCG292-08	HQ647933	Canada	British Columbia	Spencer Entomological Museum, UBC
Ectropis crepuscularia	08-JDWBC-0295	LBCG295-08	HQ647934	Canada	British Columbia	Spencer Entomological Museum, UBC
Ectropis crepuscularia	08-JDWBC-0296	LBCG296-08	HQ647935	Canada	British Columbia	Spencer Entomological Museum, UBC
Ectropis crepuscularia	08-JDWBC-0297	LBCG297-08	HQ647928	Canada	British Columbia	Spencer Entomological Museum, UBC
Ectropis crepuscularia	08-JDWBC-0304	LBCG304-08	HQ647936	Canada	British Columbia	Spencer Entomological Museum, UBC
Ectropis crepuscularia	08-JDWBC-0318	LBCG318-08	HQ647937	Canada	British Columbia	Spencer Entomological Museum, UBC
Ectropis crepuscularia	08-JDWBC-0319	LBCG319-08	HQ647938	Canada	British Columbia	Spencer Entomological Museum, UBC
Ectropis crepuscularia	CNCLEP00033106	GWNC107-07	HQ647918	Canada	British Columbia	Canadian National Collection of Insects, Arachnids and Nematodes
Ectropis crepuscularia	ENT002-001544	GWNR087-07	HQ647926	Canada	British Columbia	Royal British Columbia Museum
Ectropis crepuscularia	ENT991-10575temp	GWNR375-07	HQ647927	Canada	British Columbia	Royal British Columbia Museum
Ectropis crepuscularia	UASM57997	GWNS081-07	HQ647924	Canada	Alberta	Strickland Museum of Entomology, University of Alberta
Ectropis crepuscularia	UASM58079	GWNS080-07	HQ647925	Canada	Alberta	Strickland Museum of Entomology, University of Alberta
Ematurga amitaria	NFRC-P-2007-100016	GWNN016-07	HQ647939	Canada	Alberta	Northern Forestry Centre, Canadian Forest Service
Enchoria lacteata	CNCLEP00034143	GWNC581-07	HQ647940	United States	Washington	Canadian National Collection of Insects, Arachnids and Nematodes
Enchoria lacteata	JECW-07-0162	GWNJ162-07	HQ647944	United States	Washington	James Entomological Collection, Washington State University
Enchoria lacteata	JECW-07-0163	GWNJ163-07	HQ647942	United States	Idaho	James Entomological Collection, Washington State University
Enchoria lacteata	JECW-07-0164	GWNJ164-07	HQ647941	United States	Idaho	James Entomological Collection, Washington State University
Enchoria lacteata	PFC-2007-0332	GWNP122-07	HQ647943	Canada	British Columbia	Pacific Forestry Centre, Canadian Forest Service
Ennomos alniaria	08-JDWBC-3720	LBCG3720-09	HQ647957	Canada	British Columbia	Spencer Entomological Museum, UBC
Ennomos alniaria	08-JDWBC-3721	LBCG3721-09	HQ647956	Canada	British Columbia	Spencer Entomological Museum, UBC
Ennomos alniaria	08-JDWBC-3722	LBCG3722-09	HQ647955	Canada	British Columbia	Spencer Entomological Museum, UBC
Ennomos alniaria	08-JDWBC-3723	LBCG3723-09	HQ647954	Canada	British Columbia	Spencer Entomological Museum, UBC
Ennomos alniaria	08-JDWBC-3724	LBCG3724-09	HQ647953	Canada	British Columbia	Spencer Entomological Museum, UBC
Ennomos alniaria	08-JDWBC-3725	LBCG3725-09	HQ647952	Canada	British Columbia	Spencer Entomological Museum, UBC
Ennomos alniaria	08-JDWBC-3726	LBCG3726-09	HQ647951	Canada	British Columbia	Spencer Entomological Museum, UBC
Ennomos alniaria	08-JDWBC-3727	LBCG3727-09	HQ647950	Canada	British Columbia	Spencer Entomological Museum, UBC
Ennomos alniaria	CNCLEP00021543	GWNC457-07	HQ647946	Canada	British Columbia	Canadian National Collection of Insects, Arachnids and Nematodes
Ennomos alniaria	CNCLEP00021544	GWNC458-07	HQ647945	Canada	British Columbia	Canadian National Collection of Insects, Arachnids and Nematodes
Ennomos alniaria	ENT006-000119	GWNR339-07	HQ647949	Canada	British Columbia	Royal British Columbia Museum
Ennomos alniaria	ENT006-000120	GWNR340-07	HQ647948	Canada	British Columbia	Royal British Columbia Museum
Ennomos alniaria	ENT006-000121	GWNR341-07	HQ647947	Canada	British Columbia	Royal British Columbia Museum
Ennomos magnaria	CNCLEP00033180	GWNC181-07	HQ647958	Canada	Alberta	Canadian National Collection of Insects, Arachnids and Nematodes
Ennomos magnaria	ENT002-001611	GWNR240-07	HQ647962	Canada	British Columbia	Royal British Columbia Museum
Ennomos magnaria	ENT991-011444	GWNR239-07	HQ647963	Canada	British Columbia	Royal British Columbia Museum
Ennomos magnaria	UASM57940	GWNS155-07	HQ647959	Canada	Alberta	Strickland Museum of Entomology, University of Alberta
Ennomos magnaria	UASM58413	GWNS153-07	HQ647961	Canada	Alberta	Strickland Museum of Entomology, University of Alberta
Ennomos magnaria	UASM58860	GWNS154-07	HQ647960	Canada	Alberta	Strickland Museum of Entomology, University of Alberta
Entephria kidluitata	CNCLEP00033315	GWNC316-07	HQ647968	Canada	Yukon Territory	Canadian National Collection of Insects, Arachnids and Nematodes
Entephria kidluitata	CNCLEP00033392	GWNC393-07	HQ647967	Canada	Yukon Territory	Canadian National Collection of Insects, Arachnids and Nematodes
Entephria kidluitata	CNCLEP00033393	GWNC394-07	HQ647965	Canada	British Columbia	Canadian National Collection of Insects, Arachnids and Nematodes
Entephria kidluitata	CNCLEP00034051	GWNC489-07	HQ647964	Canada	British Columbia	Canadian National Collection of Insects, Arachnids and Nematodes
Entephria kidluitata	CNCLEP00034080	GWNC518-07	HQ647966	Canada	Yukon Territory	Canadian National Collection of Insects, Arachnids and Nematodes
Entephria kidluitata	ENT002-002425	GWNR455-07	HQ647972	Canada	British Columbia	Royal British Columbia Museum
Entephria kidluitata	UASM59231	GWNS335-07	HQ647971	Canada	Yukon Territory	Strickland Museum of Entomology, University of Alberta
Entephria kidluitata	UASM59232	GWNS336-07	HQ647970	Canada	Yukon Territory	Strickland Museum of Entomology, University of Alberta
Entephria kidluitata	UASM95814	GWNS333-07	HQ647969	Canada	British Columbia	Strickland Museum of Entomology, University of Alberta
Entephria lagganata	CNCLEP00033390	GWNC391-07	HQ647976	Canada	Alberta	Canadian National Collection of Insects, Arachnids and Nematodes
Entephria lagganata	CNCLEP00033391	GWNC392-07	HQ647975	Canada	Alberta	Canadian National Collection of Insects, Arachnids and Nematodes
Entephria lagganata	CNCLEP00034048	GWNC486-07	HQ647973	Canada	British Columbia	Canadian National Collection of Insects, Arachnids and Nematodes
Entephria lagganata	CNCLEP00034049	GWNC487-07	HQ647974	Canada	Alberta	Canadian National Collection of Insects, Arachnids and Nematodes
Entephria lagganata	UASM59277	GWNS332-07	HQ647977	Canada	Alberta	Strickland Museum of Entomology, University of Alberta
Entephria lagganata	UASM59412	GWNS331-07	HQ647978	Canada	Alberta	Strickland Museum of Entomology, University of Alberta
Entephria multivagata	CNCLEP00033388	GWNC389-07	HQ647981	Canada	Alberta	Canadian National Collection of Insects, Arachnids and Nematodes
Entephria multivagata	CNCLEP00033389	GWNC390-07	HQ647980	Canada	Alberta	Canadian National Collection of Insects, Arachnids and Nematodes
Entephria multivagata	CNCLEP00034043	GWNC481-07	HQ647979	Canada	British Columbia	Canadian National Collection of Insects, Arachnids and Nematodes
Entephria multivagata	ENT991-009943	GWNR452-07	HQ647986	Canada	British Columbia	Royal British Columbia Museum
Entephria multivagata	ENT996-004525	GWNR454-07	HQ647984	Canada	British Columbia	Royal British Columbia Museum
Entephria multivagata	ENT996-004528	GWNR453-07	HQ647985	Canada	British Columbia	Royal British Columbia Museum
Entephria multivagata	JECW-07-0148	GWNJ148-07	HQ647982	Canada	British Columbia	James Entomological Collection, Washington State University
Entephria multivagata	UASM95864	GWNS525-07	HQ647983	Canada	Alberta	Strickland Museum of Entomology, University of Alberta
Entephria takuata	CNCLEP00033314	GWNC315-07	HQ647988	Canada	Yukon Territory	Canadian National Collection of Insects, Arachnids and Nematodes
Entephria takuata	CNCLEP00034045	GWNC483-07	HQ647989	United States	Washington	Canadian National Collection of Insects, Arachnids and Nematodes
Entephria takuata	CNCLEP00034046	GWNC484-07	HQ647987	Canada	British Columbia	Canadian National Collection of Insects, Arachnids and Nematodes
Enypia griseata	Dun-08-066	DUNLP066-08	HQ647993	Canada	British Columbia	Pacific Forestry Centre, Canadian Forest Service
Enypia griseata	ENT996-004201	GWNR283-07	HQ647994	Canada	British Columbia	Royal British Columbia Museum
Enypia griseata	HLC-21021	LBCB081-05	HQ647992	Canada	British Columbia	Biodiversity Institute of Ontario
Enypia griseata	HLC-23173	LBCD353-05	HQ647991	Canada	British Columbia	Biodiversity Institute of Ontario
Enypia griseata	HLC-23181	LBCD361-05	HQ647990	Canada	British Columbia	Biodiversity Institute of Ontario
Enypia packardata	08-JDWBC-2324	LBCG2324-09	HQ648005	Canada	British Columbia	Spencer Entomological Museum, UBC
Enypia packardata	CNCLEP00033233	GWNC234-07	HQ647998	Canada	British Columbia	Canadian National Collection of Insects, Arachnids and Nematodes
Enypia packardata	CNCLEP00033234	GWNC235-07	HQ647997	Canada	British Columbia	Canadian National Collection of Insects, Arachnids and Nematodes
Enypia packardata	CNCLEP00033235	GWNC236-07	HQ647996	Canada	British Columbia	Canadian National Collection of Insects, Arachnids and Nematodes
Enypia packardata	Dun-08-065	DUNLP065-08	HQ647995	Canada	British Columbia	Pacific Forestry Centre, Canadian Forest Service
Enypia packardata	ENT996-004204	GWNR281-07	HQ648001	Canada	British Columbia	Royal British Columbia Museum
Enypia packardata	ENT996-004207	GWNR279-07	HQ648002	Canada	British Columbia	Royal British Columbia Museum
Enypia packardata	ENT996-004214	GWNR282-07	HQ648000	Canada	British Columbia	Royal British Columbia Museum
Enypia packardata	ENT996-004215	GWNR284-07	HQ648006	United States	Washington	Royal British Columbia Museum
Enypia packardata	PFC-2007-0390	GWNP180-07	HQ648004	Canada	British Columbia	Pacific Forestry Centre, Canadian Forest Service
Enypia packardata	PFC-2007-0391	GWNP181-07	HQ648003	Canada	British Columbia	Pacific Forestry Centre, Canadian Forest Service
Enypia packardata	UASM95847	GWNS497-07	HQ647999	Canada	Alberta	Strickland Museum of Entomology, University of Alberta
Enypia venata	08-JDWBC-0746	LBCG746-09	HQ648011	Canada	British Columbia	Spencer Entomological Museum, UBC
Enypia venata	08-JDWBC-0973	LBCG973-09	HQ648010	Canada	British Columbia	Spencer Entomological Museum, UBC
Enypia venata	08-JDWBC-1844	LBCG1844-09	HQ648018	Canada	British Columbia	Spencer Entomological Museum, UBC
Enypia venata	08-JDWBC-1845	LBCG1845-09	HQ648017	Canada	British Columbia	Spencer Entomological Museum, UBC
Enypia venata	08-JDWBC-1846	LBCG1846-09	HQ648016	Canada	British Columbia	Spencer Entomological Museum, UBC
Enypia venata	08-JDWBC-1847	LBCG1847-09	HQ648015	Canada	British Columbia	Spencer Entomological Museum, UBC
Enypia venata	08-JDWBC-1848	LBCG1848-09	HQ648014	Canada	British Columbia	Spencer Entomological Museum, UBC

Enypia venata	Dun-08-067	DUNLP067-08	HQ648007	Canada	British Columbia	Pacific Forestry Centre, Canadian Forest Service
Enypia venata	ENT996-004088	GWNR287-07	HQ648012	Canada	British Columbia	Royal British Columbia Museum
Enypia venata	ENT996-007583	GWNR285-07	HQ648013	Canada	British Columbia	Royal British Columbia Museum
Enypia venata	UASM95855	GWNS498-07	HQ648009	Canada	Alberta	Strickland Museum of Entomology, University of Alberta
Enypia venata	UASM95856	GWNS499-07	HQ648008	Canada	Alberta	Strickland Museum of Entomology, University of Alberta
Epirrhoe alternata	ENT002-002433	GWNR541-07	HQ648022	Canada	British Columbia	Royal British Columbia Museum
Epirrhoe alternata	ENT992-020003	GWNR540-07	HQ648023	Canada	British Columbia	Royal British Columbia Museum
Epirrhoe alternata	HLC-20554	LBCA554-05	HQ648019	Canada	British Columbia	Biodiversity Institute of Ontario
Epirrhoe alternata	UASM7021	GWNS387-07	HQ648020	Canada	Alberta	Strickland Museum of Entomology, University of Alberta
Epirrhoe alternata	UASM95838	GWNS496-07	HQ648021	Canada	Yukon Territory	Strickland Museum of Entomology, University of Alberta
Epirrhoe plebeculata	ENT996-004721	GWNR544-07	HQ648028	United States	Washington	Royal British Columbia Museum
Epirrhoe plebeculata	ENT996-004728	GWNR542-07	HQ648029	Canada	British Columbia	Royal British Columbia Museum
Epirrhoe plebeculata	JECW-07-0159	GWNJ159-07	HQ648025	United States	Washington	James Entomological Collection, Washington State University
Epirrhoe plebeculata	JECW-07-0160	GWNJ160-07	HQ648024	United States	Idaho	James Entomological Collection, Washington State University
Epirrhoe plebeculata	PFC-2007-0330	GWNP120-07	HQ648027	Canada	British Columbia	Pacific Forestry Centre, Canadian Forest Service
Epirrhoe plebeculata	UASM41837	GWNS388-07	HQ648026	Canada	Alberta	Strickland Museum of Entomology, University of Alberta
Epirrhoe sperryi	ENT002-001729	GWNR547-07	HQ648035	Canada	British Columbia	Royal British Columbia Museum
Epirrhoe sperryi	ENT002-001733	GWNR548-07	HQ648034	Canada	British Columbia	Royal British Columbia Museum
Epirrhoe sperryi	ENT991-006425	GWNR546-07	HQ648036	Canada	Alberta	Royal British Columbia Museum
Epirrhoe sperryi	ENT991-012521	GWNR545-07	HQ648037	Canada	British Columbia	Royal British Columbia Museum
Epirrhoe sperryi	JD0121	GWNS390-07	HQ648031	Canada	Alberta	Strickland Museum of Entomology, University of Alberta
Epirrhoe sperryi	UASM95829	GWNS389-07	HQ648032	Canada	Alberta	Strickland Museum of Entomology, University of Alberta
Epirrhoe sperryi	UASM95831	GWNS391-07	HQ648030	Canada	Alberta	Strickland Museum of Entomology, University of Alberta
Epirrhoe sperryi	UASM95882	GWNS594-07	HQ648033	Canada	Alberta	Strickland Museum of Entomology, University of Alberta
Epirrita autumnata	ENT002-001746	GWNR596-07	HQ648042	Canada	British Columbia	Royal British Columbia Museum
Epirrita autumnata	ENT002-001747	GWNR594-07	HQ648044	Canada	British Columbia	Royal British Columbia Museum
Epirrita autumnata	ENT002-001748	GWNR595-07	HQ648043	Canada	British Columbia	Royal British Columbia Museum
Epirrita autumnata	ENT996-004886	GWNR593-07	HQ648045	Canada	British Columbia	Royal British Columbia Museum
Epirrita autumnata	NFRC-P-2007-100088	GWNN088-07	HQ648038	Canada	Alberta	Northern Forestry Centre, Canadian Forest Service
Epirrita autumnata	PFC-2007-0243	GWNP036-07	HQ648041	Canada	Yukon Territory	Pacific Forestry Centre, Canadian Forest Service
Epirrita autumnata	PFC-2007-0244	GWNP037-07	HQ648040	Canada	British Columbia	Pacific Forestry Centre, Canadian Forest Service
Epirrita autumnata	PFC-2007-0245	GWNP038-07	HQ648039	Canada	British Columbia	Pacific Forestry Centre, Canadian Forest Service
Epirrita pulchraria	PFC-2007-0238	GWNP031-07	HQ648047	Canada	British Columbia	Pacific Forestry Centre, Canadian Forest Service
Epirrita pulchraria	PFC-2007-0239	GWNP032-07	HQ648046	Canada	British Columbia	Pacific Forestry Centre, Canadian Forest Service
Epirrita undulata	UASM41725	GWNS406-07	HQ648048	Canada	Alberta	Strickland Museum of Entomology, University of Alberta
Erannis tiliaria	UASM41366	GWNS097-07	HQ648051	Canada	Alberta	Strickland Museum of Entomology, University of Alberta
Erannis tiliaria	UASM56902	GWNS098-07	HQ648050	Canada	Alberta	Strickland Museum of Entomology, University of Alberta
Erannis tiliaria	UASM7056	GWNS099-07	HQ648049	Canada	Alberta	Strickland Museum of Entomology, University of Alberta
Erannis tiliaria	UASM95879	GWNS580-07	HQ648052	Canada	Alberta	Strickland Museum of Entomology, University of Alberta
Erannis tiliaria vancouverensis	CNCLEP00033124	GWNC125-07	HQ648055	Canada	British Columbia	Canadian National Collection of Insects, Arachnids and Nematodes
Erannis tiliaria vancouverensis	CNCLEP00033125	GWNC126-07	HQ648054	Canada	British Columbia	Canadian National Collection of Insects, Arachnids and Nematodes
Erannis tiliaria vancouverensis	Dun-08-069	DUNLP069-08	HQ648053	Canada	British Columbia	Pacific Forestry Centre, Canadian Forest Service
Erannis tiliaria vancouverensis	ENT991-010166	GWNR133-07	HQ648058	Canada	British Columbia	Royal British Columbia Museum
Erannis tiliaria vancouverensis	ENT996-003643	GWNR134-07	HQ648057	Canada	British Columbia	Royal British Columbia Museum
Erannis tiliaria vancouverensis	ENT996-003646	GWNR135-07	HQ648056	United States	Washington	Royal British Columbia Museum
Erannis tiliaria vancouverensis	PFC-2007-0380	GWNP170-07	HQ648059	Canada	British Columbia	Pacific Forestry Centre, Canadian Forest Service
Eubaphe mendica	ENT996-007836	GWNR643-07	HQ648064	Canada	British Columbia	Royal British Columbia Museum
Eubaphe mendica	ENT996-007837	GWNR644-07	HQ648063	Canada	British Columbia	Royal British Columbia Museum
Eubaphe mendica	ENT996-007841	GWNR645-07	HQ648062	Canada	British Columbia	Royal British Columbia Museum
Eubaphe mendica	UASM43221	GWNS411-07	HQ648060	Canada	Alberta	Strickland Museum of Entomology, University of Alberta
Eubaphe mendica	UASM7024	GWNS409-07	HQ648061	Canada	Alberta	Strickland Museum of Entomology, University of Alberta
Eubaphe unicolor	WFBM-07-0087	GWNW087-07	HQ648067	United States	Arizona	University of Idaho, W. F. Barr Entomological Collection
Eubaphe unicolor	WFBM-07-0088	GWNW088-07	HQ648066	United States	Arizona	University of Idaho, W. F. Barr Entomological Collection
Eubaphe unicolor	WFBM-07-0089	GWNW089-07	HQ648065	United States	Texas	University of Idaho, W. F. Barr Entomological Collection
Euchlaena johnsonaria	08-JDWBC-1081	LBCG1081-09	HQ648076	Canada	British Columbia	Spencer Entomological Museum, UBC
Euchlaena johnsonaria	08-JDWBC-1083	LBCG1083-09	HQ648075	Canada	British Columbia	Spencer Entomological Museum, UBC
Euchlaena johnsonaria	08-JDWBC-2103	LBCG2103-09	HQ648080	Canada	British Columbia	Spencer Entomological Museum, UBC
Euchlaena johnsonaria	08-JDWBC-3309	LBCG3309-09	HQ648078	Canada	British Columbia	Spencer Entomological Museum, UBC
Euchlaena johnsonaria	08-JDWBC-3310	LBCG3310-09	HQ648077	Canada	British Columbia	Spencer Entomological Museum, UBC
Euchlaena johnsonaria	ENT996-003779	GWNR188-07	HQ648079	Canada	British Columbia	Royal British Columbia Museum
Euchlaena johnsonaria	ENT996-007436	GWNR372-07	HQ648073	Canada	British Columbia	Royal British Columbia Museum
Euchlaena johnsonaria	ENT996-007437	GWNR373-07	HQ648072	Canada	British Columbia	Royal British Columbia Museum
Euchlaena johnsonaria	ENT996-007438	GWNR374-07	HQ648071	Canada	British Columbia	Royal British Columbia Museum
Euchlaena johnsonaria	SEM-UBC-GEO-0154	GWNU090-07	HQ648074	Canada	British Columbia	Spencer Entomological Museum, UBC
Euchlaena johnsonaria	UASM57957	GWNS119-07	HQ648069	Canada	Alberta	Strickland Museum of Entomology, University of Alberta
Euchlaena johnsonaria	UASM58447	GWNS118-07	HQ648070	Canada	Alberta	Strickland Museum of Entomology, University of Alberta
Euchlaena johnsonaria	UASM58497	GWNS120-07	HQ648068	Canada	Alberta	Strickland Museum of Entomology, University of Alberta
Euchlaena madusaria	CNCLEP00033164	GWNC165-07	HQ648081	Canada	British Columbia	Canadian National Collection of Insects, Arachnids and Nematodes
Euchlaena madusaria	ENT996-003813	GWNR190-07	HQ648086	Canada	British Columbia	Royal British Columbia Museum
Euchlaena madusaria	ENT996-003815	GWNR191-07	HQ648085	Canada	British Columbia	Royal British Columbia Museum
Euchlaena madusaria	UASM58366	GWNS122-07	HQ648083	Canada	Alberta	Strickland Museum of Entomology, University of Alberta
Euchlaena madusaria	UASM58673	GWNS121-07	HQ648084	Canada	Alberta	Strickland Museum of Entomology, University of Alberta
Euchlaena madusaria	UASM78420	GWNS123-07	HQ648082	Canada	Alberta	Strickland Museum of Entomology, University of Alberta
Euchlaena marginaria	ENT002-001600	GWNR193-07	HQ648090	Canada	British Columbia	Royal British Columbia Museum
Euchlaena marginaria	ENT996-003812	GWNR192-07	HQ648091	Canada	British Columbia	Royal British Columbia Museum
Euchlaena marginaria	UASM57158	GWNS124-07	HQ648089	Canada	Alberta	Strickland Museum of Entomology, University of Alberta
Euchlaena marginaria	UASM58233	GWNS125-07	HQ648088	Canada	Alberta	Strickland Museum of Entomology, University of Alberta
Euchlaena marginaria	UASM7061	GWNS126-07	HQ648087	Canada	Alberta	Strickland Museum of Entomology, University of Alberta
Euchlaena obtusaria	UASM41294	GWNS116-07	HQ648093	Canada	Alberta	Strickland Museum of Entomology, University of Alberta
Euchlaena obtusaria	UASM41966	GWNS117-07	HQ648092	Canada	Alberta	Strickland Museum of Entomology, University of Alberta
Euchlaena tigrinaria	ENT002-001601	GWNR197-07	HQ648096	Canada	British Columbia	Royal British Columbia Museum
Euchlaena tigrinaria	ENT996-003830	GWNR195-07	HQ648097	Canada	British Columbia	Royal British Columbia Museum
Euchlaena tigrinaria	ENT996-007448	GWNR194-07	HQ648098	Canada	British Columbia	Royal British Columbia Museum
Euchlaena tigrinaria	UASM57958	GWNS127-07	HQ648095	Canada	Alberta	Strickland Museum of Entomology, University of Alberta
Euchlaena tigrinaria	UASM7062	GWNS128-07	HQ648094	Canada	Alberta	Strickland Museum of Entomology, University of Alberta
Eudrepanulatrix rectifascia	08-JDWBC-1062	LBCG1062-09	HQ648102	Canada	British Columbia	Spencer Entomological Museum, UBC
Eudrepanulatrix rectifascia	08-JDWBC-1086	LBCG1086-09	HQ648101	Canada	British Columbia	Spencer Entomological Museum, UBC
Eudrepanulatrix rectifascia	08-JDWBC-1094	LBCG1094-09	HQ648100	Canada	British Columbia	Spencer Entomological Museum, UBC
Eudrepanulatrix rectifascia	CNCLEP00033139	GWNC140-07	HQ648099	Canada	British Columbia	Canadian National Collection of Insects, Arachnids and Nematodes
Eudrepanulatrix rectifascia	ENT996-003756	GWNR172-07	HQ648103	United States	Washington	Royal British Columbia Museum
Eufidonia convergaria	PFC-2007-0364	GWNP154-07	HQ648107	Canada	British Columbia	Pacific Forestry Centre, Canadian Forest Service
Eufidonia convergaria	PFC-2007-0365	GWNP155-07	HQ648106	Canada	British Columbia	Pacific Forestry Centre, Canadian Forest Service
Eufidonia convergaria	UASM24862	GWNS086-07	HQ648105	Canada	Alberta	Strickland Museum of Entomology, University of Alberta
Eufidonia convergaria	UASM41707	GWNS087-07	HQ648104	Canada	Alberta	Strickland Museum of Entomology, University of Alberta
Eufidonia discospilata	CNCLEP00033360	GWNC361-07	HQ648108	Canada	Alberta	Canadian National Collection of Insects, Arachnids and Nematodes
Eufidonia discospilata	ENT002-001547	GWNR118-07	HQ648112	Canada	British Columbia	Royal British Columbia Museum
Eufidonia discospilata	ENT002-001548	GWNR117-07	HQ648113	Canada	British Columbia	Royal British Columbia Museum
Eufidonia discospilata	PFC-2007-0366	GWNP156-07	HQ648111	Canada	British Columbia	Pacific Forestry Centre, Canadian Forest Service
Eufidonia discospilata	UASM53042	GWNS089-07	HQ648109	Canada	Alberta	Strickland Museum of Entomology, University of Alberta
Eufidonia discospilata	UASM53045	GWNS088-07	HQ648110	Canada	Alberta	Strickland Museum of Entomology, University of Alberta
Eulithis destinata	CNCLEP00033382	GWNC383-07	HQ648120	Canada	Alberta	Canadian National Collection of Insects, Arachnids and Nematodes
Eulithis destinata	CNCLEP00033383	GWNC384-07	HQ648115	Canada	British Columbia	Canadian National Collection of Insects, Arachnids and Nematodes
Eulithis destinata	CNCLEP00033419	GWNC420-07	HQ648119	United States	Alaska	Canadian National Collection of Insects, Arachnids and Nematodes
Eulithis destinata	CNCLEP00033420	GWNC421-07	HQ648116	Canada	Yukon Territory	Canadian National Collection of Insects, Arachnids and Nematodes
Eulithis destinata	CNCLEP00033421	GWNC422-07	HQ648118	United States	Alaska	Canadian National Collection of Insects, Arachnids and Nematodes
Eulithis destinata	CNCLEP00033423	GWNC424-07	HQ648117	United States	Washington	Canadian National Collection of Insects, Arachnids and Nematodes
Eulithis destinata	Dun-08-070	DUNLP070-08	HQ648114	Canada	British Columbia	Pacific Forestry Centre, Canadian Forest Service

Eulithis destinata	ENT987-001242	GWNR457-07	HQ648124	Canada	British Columbia	Royal British Columbia Museum
Eulithis destinata	ENT991-131361	GWNR456-07	HQ648125	Canada	British Columbia	Royal British Columbia Museum
Eulithis destinata	JECW-07-0136	GWNJ136-07	HQ648121	United States	Washington	James Entomological Collection, Washington State University
Eulithis destinata	PFC-2007-0283	GWNP076-07	HQ648123	Canada	British Columbia	Pacific Forestry Centre, Canadian Forest Service
Eulithis destinata	PFC-2007-0284	GWNP077-07	HQ648122	Canada	British Columbia	Pacific Forestry Centre, Canadian Forest Service
Eulithis explanata	CNCLEP00033384	GWNC385-07	HQ648127	Canada	Alberta	Canadian National Collection of Insects, Arachnids and Nematodes
Eulithis explanata	CNCLEP00033385	GWNC386-07	HQ648126	Canada	Alberta	Canadian National Collection of Insects, Arachnids and Nematodes
Eulithis explanata	UASM41842	GWNS304-07	HQ648131	Canada	Alberta	Strickland Museum of Entomology, University of Alberta
Eulithis explanata	UASM41843	GWNS303-07	HQ648132	Canada	Alberta	Strickland Museum of Entomology, University of Alberta
Eulithis explanata	UASM7350	GWNS306-07	HQ648129	Canada	Alberta	Strickland Museum of Entomology, University of Alberta
Eulithis explanata	UASM7376	GWNS305-07	HQ648130	Canada	Alberta	Strickland Museum of Entomology, University of Alberta
Eulithis explanata	UASM95854	GWNS520-07	HQ648128	Canada	Alberta	Strickland Museum of Entomology, University of Alberta
Eulithis flavibrunneata	CNCLEP00033386	GWNC387-07	HQ648134	Canada	Alberta	Canadian National Collection of Insects, Arachnids and Nematodes
Eulithis flavibrunneata	CNCLEP00033387	GWNC388-07	HQ648133	Canada	Alberta	Canadian National Collection of Insects, Arachnids and Nematodes
Eulithis flavibrunneata	UASM7328	GWNS302-07	HQ648135	Canada	Alberta	Strickland Museum of Entomology, University of Alberta
Eulithis propulsata	08-JDWBC-0771	LBCG771-09	HQ648144	Canada	British Columbia	Spencer Entomological Museum, UBC
Eulithis propulsata	08-JDWBC-1841	LBCG1841-09	HQ648145	Canada	British Columbia	Spencer Entomological Museum, UBC
Eulithis propulsata	CNCLEP00033416	GWNC417-07	HQ648136	Canada	Alberta	Canadian National Collection of Insects, Arachnids and Nematodes
Eulithis propulsata	ENT002-001697	GWNR459-07	HQ648142	Canada	British Columbia	Royal British Columbia Museum
Eulithis propulsata	ENT996-004698	GWNR458-07	HQ648143	Canada	British Columbia	Royal British Columbia Museum
Eulithis propulsata	PFC-2007-0282	GWNP075-07	HQ648137	Canada	British Columbia	Pacific Forestry Centre, Canadian Forest Service
Eulithis propulsata	UASM43259	GWNS296-07	HQ648139	Canada	Alberta	Strickland Museum of Entomology, University of Alberta
Eulithis propulsata	UASM57960	GWNS294-07	HQ648141	Canada	Alberta	Strickland Museum of Entomology, University of Alberta
Eulithis propulsata	UASM58361	GWNS295-07	HQ648140	Canada	Alberta	Strickland Museum of Entomology, University of Alberta
Eulithis propulsata	UASM7336	GWNS297-07	HQ648138	Canada	Alberta	Strickland Museum of Entomology, University of Alberta
Eulithis testata	ENT002-001699	GWNR460-07	HQ648150	Canada	British Columbia	Royal British Columbia Museum
Eulithis testata	UASM28796	GWNS301-07	HQ648146	Canada	Alberta	Strickland Museum of Entomology, University of Alberta
Eulithis testata	UASM58365	GWNS300-07	HQ648147	Canada	Alberta	Strickland Museum of Entomology, University of Alberta
Eulithis testata	UASM58861	GWNS298-07	HQ648149	Canada	Alberta	Strickland Museum of Entomology, University of Alberta
Eulithis testata	UASM7320	GWNS299-07	HQ648148	Canada	Alberta	Strickland Museum of Entomology, University of Alberta
Eulithis xylina	08-JDWBC-2323	LBCG2323-09	HQ648162	Canada	British Columbia	Spencer Entomological Museum, UBC
Eulithis xylina	08-JDWBC-2325	LBCG2325-09	HQ648163	Canada	British Columbia	Spencer Entomological Museum, UBC
Eulithis xylina	08-JDWBC-2326	LBCG2326-09	HQ648164	Canada	British Columbia	Spencer Entomological Museum, UBC
Eulithis xylina	08-JDWBC-2329	LBCG2329-09	HQ648165	Canada	British Columbia	Spencer Entomological Museum, UBC
Eulithis xylina	08-JDWBC-2331	LBCG2331-09	HQ648166	Canada	British Columbia	Spencer Entomological Museum, UBC
Eulithis xylina	08-JDWBC-3699	LBCG3699-09	HQ648167	Canada	British Columbia	Royal British Columbia Museum
Eulithis xylina	CNCLEP00033418	GWNC419-07	HQ648151	Canada	Yukon Territory	Canadian National Collection of Insects, Arachnids and Nematodes
Eulithis xylina	ENT996-004649	GWNR463-07	HQ648160	Canada	British Columbia	Royal British Columbia Museum
Eulithis xylina	ENT996-004701	GWNR464-07	HQ648159	Canada	British Columbia	Royal British Columbia Museum
Eulithis xylina	ENT996-004713	GWNR462-07	HQ648161	Canada	British Columbia	Royal British Columbia Museum
Eulithis xylina	JECW-07-0137	GWNJ137-07	HQ648152	United States	Washington	James Entomological Collection, Washington State University
Eulithis xylina	PFC-2007-0285	GWNP078-07	HQ648155	Canada	British Columbia	Pacific Forestry Centre, Canadian Forest Service
Eulithis xylina	PFC-2007-0286	GWNP079-07	HQ648154	Canada	British Columbia	Pacific Forestry Centre, Canadian Forest Service
Eulithis xylina	UASM24918	GWNS309-07	HQ648156	Canada	Alberta	Strickland Museum of Entomology, University of Alberta
Eulithis xylina	UASM7335	GWNS307-07	HQ648158	Canada	Alberta	Strickland Museum of Entomology, University of Alberta
Eulithis xylina	UASM7401	GWNS308-07	HQ648157	Canada	Alberta	Strickland Museum of Entomology, University of Alberta
Eulithis xylina	UASM95857	GWNS518-07	HQ648153	Canada	Alberta	Strickland Museum of Entomology, University of Alberta
Eumacaria latiferrugata	08-JDWBC-0123	LBCG123-08	HQ648170	Canada	British Columbia	Spencer Entomological Museum, UBC
Eumacaria latiferrugata	08-JDWBC-0129	LBCG129-08	HQ648169	Canada	British Columbia	Spencer Entomological Museum, UBC
Eumacaria latiferrugata	UASM24114	GWNS007-07	HQ648168	Canada	Alberta	Strickland Museum of Entomology, University of Alberta
Euphyia intermediata	CNCLEP00033335	GWNC336-07	HQ648173	Canada	Alberta	Canadian National Collection of Insects, Arachnids and Nematodes
Euphyia intermediata	CNCLEP00033336	GWNC337-07	HQ648172	Canada	Alberta	Canadian National Collection of Insects, Arachnids and Nematodes
Euphyia intermediata	CNCLEP00034074	GWNC512-07	HQ648171	Canada	British Columbia	Canadian National Collection of Insects, Arachnids and Nematodes
Euphyia intermediata	ENT996-007806	GWNR640-07	HQ648181	Canada	British Columbia	Royal British Columbia Museum
Euphyia intermediata	ENT996-007807	GWNR641-07	HQ648180	Canada	British Columbia	Royal British Columbia Museum
Euphyia intermediata	ENT996-007808	GWNR642-07	HQ648179	Canada	British Columbia	Royal British Columbia Museum
Euphyia intermediata	JECW-07-0161	GWNJ161-07	HQ648177	United States	Washington	James Entomological Collection, Washington State University
Euphyia intermediata	UASM7022	GWNS392-07	HQ648176	Canada	Alberta	Strickland Museum of Entomology, University of Alberta
Euphyia intermediata	UASM7353	GWNS386-07	HQ648174	Canada	Alberta	Strickland Museum of Entomology, University of Alberta
Euphyia intermediata	UASM7360	GWNS385-07	HQ648175	Canada	Alberta	Strickland Museum of Entomology, University of Alberta
Euphyia intermediata	UASM78454	GWNS578-07	HQ648178	Canada	Alberta	Strickland Museum of Entomology, University of Alberta
Eupithecia absinthiata	CGWC-2612	LOWCC732-05	HQ648184	Canada	British Columbia	Biodiversity Institute of Ontario
Eupithecia absinthiata	HLC-22131	LBCC251-05	HQ648183	Canada	British Columbia	Biodiversity Institute of Ontario
Eupithecia absinthiata	HLC-22146	LBCC266-05	HQ648182	Canada	British Columbia	Biodiversity Institute of Ontario
Eupithecia absinthiata	UASM58790	GWNS587-07	HQ648186	Canada	Alberta	Strickland Museum of Entomology, University of Alberta
Eupithecia absinthiata	UASM59018	GWNS442-07	HQ648185	Canada	Alberta	Strickland Museum of Entomology, University of Alberta
Eupithecia agnesata	CNCLEP00035449	GWNC683-07	HQ648187	United States	California	Canadian National Collection of Insects, Arachnids and Nematodes
Eupithecia albicapitata	UASM41222	GWNS465-07	HQ648188	Canada	Alberta	Strickland Museum of Entomology, University of Alberta
Eupithecia albicapitata	UASM41223	GWNS464-07	HQ648189	Canada	Alberta	Strickland Museum of Entomology, University of Alberta
Eupithecia albicapitata	UASM58007	GWNS463-07	HQ648190	Canada	Alberta	Strickland Museum of Entomology, University of Alberta
Eupithecia annulata	07-JDWBC-0054	GWND046-07	HQ648201	Canada	British Columbia	Royal British Columbia Museum
Eupithecia annulata	Dun-08-072	DUNLP072-08	HQ648200	Canada	British Columbia	Pacific Forestry Centre, Canadian Forest Service
Eupithecia annulata	Dun-08-074	DUNLP074-08	HQ648199	Canada	British Columbia	Pacific Forestry Centre, Canadian Forest Service
Eupithecia annulata	Dun-08-076	DUNLP076-08	HQ648198	Canada	British Columbia	Pacific Forestry Centre, Canadian Forest Service
Eupithecia annulata	Dun-08-077	DUNLP077-08	HQ648197	Canada	British Columbia	Pacific Forestry Centre, Canadian Forest Service
Eupithecia annulata	Dun-08-078	DUNLP078-08	HQ648196	Canada	British Columbia	Pacific Forestry Centre, Canadian Forest Service
Eupithecia annulata	Dun-08-079	DUNLP079-08	HQ648195	Canada	British Columbia	Pacific Forestry Centre, Canadian Forest Service
Eupithecia annulata	Dun-08-086	DUNLP086-08	HQ648194	Canada	British Columbia	Pacific Forestry Centre, Canadian Forest Service
Eupithecia annulata	Dun-08-087	DUNLP087-08	HQ648193	Canada	British Columbia	Pacific Forestry Centre, Canadian Forest Service
Eupithecia annulata	Dun-08-088	DUNLP088-08	HQ648192	Canada	British Columbia	Pacific Forestry Centre, Canadian Forest Service
Eupithecia annulata	Dun-08-089	DUNLP089-08	HQ648191	Canada	British Columbia	Pacific Forestry Centre, Canadian Forest Service
Eupithecia annulata	PFC-2007-0260	GWNP053-07	HQ648204	Canada	British Columbia	Pacific Forestry Centre, Canadian Forest Service
Eupithecia annulata	UASM59011	GWNS455-07	HQ648203	Canada	Alberta	Strickland Museum of Entomology, University of Alberta
Eupithecia annulata	UASM59157	GWNS456-07	HQ648202	Canada	Alberta	Strickland Museum of Entomology, University of Alberta
Eupithecia anticaria	HLC-20540	LBCA540-05	HQ648205	Canada	British Columbia	Biodiversity Institute of Ontario
Eupithecia anticaria	UASM59014	GWNS469-07	HQ648206	Canada	Alberta	Strickland Museum of Entomology, University of Alberta
Eupithecia anticaria	UASM59015	GWNS467-07	HQ648208	Canada	Alberta	Strickland Museum of Entomology, University of Alberta
Eupithecia anticaria	UASM59016	GWNS468-07	HQ648207	Canada	Alberta	Strickland Museum of Entomology, University of Alberta
Eupithecia anticaria	UASM59051	GWNS439-07	HQ648209	Canada	Alberta	Strickland Museum of Entomology, University of Alberta
Eupithecia assimilata	CGWC-2548	LOWCC668-05	HQ648212	Canada	British Columbia	Biodiversity Institute of Ontario
Eupithecia assimilata	CGWC-2560	LOWCC680-05	HQ648211	Canada	British Columbia	Biodiversity Institute of Ontario
Eupithecia assimilata	CGWC-2610	LOWCC730-05	HQ648210	Canada	British Columbia	Biodiversity Institute of Ontario
Eupithecia assimilata	NFRC-P-2004-005568	GWNN110-07	HQ648213	Canada	Alberta	Northern Forestry Centre, Canadian Forest Service
Eupithecia behrensata	CGWC-2676	LOWCC796-05	HQ648214	Canada	British Columbia	Biodiversity Institute of Ontario
Eupithecia behrensata	UASM57502	GWNS446-07	HQ648216	Canada	Alberta	Strickland Museum of Entomology, University of Alberta
Eupithecia behrensata	UASM57503	GWNS445-07	HQ648217	Canada	Alberta	Strickland Museum of Entomology, University of Alberta
Eupithecia behrensata	UASM58370	GWNS448-07	HQ648215	Canada	Alberta	Strickland Museum of Entomology, University of Alberta
Eupithecia borealis	08-JDWBC-1396	LBCG1396-09	HQ648222	Canada	British Columbia	Spencer Entomological Museum, UBC
Eupithecia borealis	CGWC-2591	LOWCC711-05	HQ648221	Canada	British Columbia	Biodiversity Institute of Ontario
Eupithecia borealis	CGWC-2613	LOWCC733-05	HQ648220	Canada	British Columbia	Biodiversity Institute of Ontario
Eupithecia borealis	CGWC-2635	LOWCC755-05	HQ648219	Canada	British Columbia	Biodiversity Institute of Ontario
Eupithecia borealis	CGWC-2671	LOWCC791-05	HQ648218	Canada	British Columbia	Biodiversity Institute of Ontario
Eupithecia bryanti	08-JDWBC-0796	LBCG796-09	HQ648227	Canada	British Columbia	Spencer Entomological Museum, UBC
Eupithecia bryanti	CNCLEP00034099	GWNC537-07	HQ648225	Canada	Yukon Territory	Canadian National Collection of Insects, Arachnids and Nematodes
Eupithecia bryanti	CNCLEP00034100	GWNC538-07	HQ648226	United States	Alaska	Canadian National Collection of Insects, Arachnids and Nematodes
Eupithecia bryanti	HLC-22502	LBCC622-05	HQ648224	Canada	British Columbia	Biodiversity Institute of Ontario
Eupithecia bryanti	HLC-23473	LBCD653-05	HQ648223	Canada	British Columbia	Biodiversity Institute of Ontario

Eupithecia casloata	CNCLEP00034110	GWNC548-07	HQ648229	Canada	Yukon Territory	Canadian National Collection of Insects, Arachnids and Nematodes
Eupithecia casloata	HLC-21413	LBCB473-05	HQ648228	Canada	British Columbia	Biodiversity Institute of Ontario
Eupithecia casloata	UASM41220	GWNS435-07	HQ648231	Canada	Alberta	Strickland Museum of Entomology, University of Alberta
Eupithecia casloata	UASM78504	GWNS436-07	HQ648230	Canada	Alberta	Strickland Museum of Entomology, University of Alberta
Eupithecia casloata	UASM78505	GWNS434-07	HQ648232	Canada	Alberta	Strickland Museum of Entomology, University of Alberta
Eupithecia cimicifugata	UASM95846	GWNS493-07	HQ648233	Canada	Alberta	Strickland Museum of Entomology, University of Alberta
Eupithecia coloradensis	UASM59026	GWNS424-07	HQ648234	Canada	Alberta	Strickland Museum of Entomology, University of Alberta
Eupithecia columbiata	UASM57330	GWNS491-07	HQ648235	Canada	Alberta	Strickland Museum of Entomology, University of Alberta
Eupithecia columbiata	UASM57332	GWNS441-07	HQ648236	Canada	Alberta	Strickland Museum of Entomology, University of Alberta
Eupithecia columbiata	UASM57978	GWNS419-07	HQ648239	Canada	Alberta	Strickland Museum of Entomology, University of Alberta
Eupithecia columbiata	UASM57980	GWNS420-07	HQ648238	Canada	Alberta	Strickland Museum of Entomology, University of Alberta
Eupithecia columbiata	UASM58002	GWNS421-07	HQ648237	Canada	Alberta	Strickland Museum of Entomology, University of Alberta
Eupithecia cretaceata	CNCLEP00034116	GWNC554-07	HQ648242	Canada	Yukon Territory	Canadian National Collection of Insects, Arachnids and Nematodes
Eupithecia cretaceata	ENT002-002426	GWNR603-07	HQ648244	Canada	British Columbia	Royal British Columbia Museum
Eupithecia cretaceata	HLC-21677	LBCB737-05	HQ648241	Canada	British Columbia	Biodiversity Institute of Ontario
Eupithecia cretaceata	HLC-22897	LBCD077-05	HQ648240	Canada	British Columbia	Biodiversity Institute of Ontario
Eupithecia cretaceata	UASM95876	GWNS547-07	HQ648243	Canada	Alberta	Strickland Museum of Entomology, University of Alberta
Eupithecia gelidata	UASM41224	GWNS438-07	HQ648246	Canada	Alberta	Strickland Museum of Entomology, University of Alberta
Eupithecia gelidata	UASM41225	GWNS449-07	HQ648245	Canada	Alberta	Strickland Museum of Entomology, University of Alberta
Eupithecia gilvipennata	CNCLEP00035456	GWNC690-07	HQ648248	Canada	British Columbia	Canadian National Collection of Insects, Arachnids and Nematodes
Eupithecia gilvipennata	CNCLEP00035457	GWNC691-07	HQ648247	Canada	British Columbia	Canadian National Collection of Insects, Arachnids and Nematodes
Eupithecia graefii	CBCC1094	GWNS471-07	HQ648252	Canada	Alberta	Strickland Museum of Entomology, University of Alberta
Eupithecia graefii	HLC-20921	LBCA921-05	HQ648251	Canada	British Columbia	Biodiversity Institute of Ontario
Eupithecia graefii	HLC-21321	LBCB381-05	HQ648250	Canada	British Columbia	Biodiversity Institute of Ontario
Eupithecia graefii	HLC-21487	LBCB547-05	HQ648249	Canada	British Columbia	Biodiversity Institute of Ontario
Eupithecia graefii	UASM59177	GWNS470-07	HQ648253	Canada	Alberta	Strickland Museum of Entomology, University of Alberta
Eupithecia harrisonata	PFC-2007-0272	GWNP065-07	HQ648254	Canada	British Columbia	Pacific Forestry Centre, Canadian Forest Service
Eupithecia interruptofasciata	CGWC-1141	LOWCB201-05	HQ648258	Canada	British Columbia	Biodiversity Institute of Ontario
Eupithecia interruptofasciata	CGWC-1155	LOWCB215-05	HQ648257	Canada	British Columbia	Biodiversity Institute of Ontario
Eupithecia interruptofasciata	CGWC-1202	LOWCB262-05	HQ648256	Canada	British Columbia	Biodiversity Institute of Ontario
Eupithecia interruptofasciata	CGWC-1205	LOWCB265-05	HQ648255	Canada	British Columbia	Biodiversity Institute of Ontario
Eupithecia interruptofasciata	Dun-08-081	DUNLP081-08	HQ648259	Canada	British Columbia	Pacific Forestry Centre, Canadian Forest Service
Eupithecia intricata	CGWC-4041	LOWCE281-06	HQ648260	Canada	British Columbia	Biodiversity Institute of Ontario
Eupithecia johnstoni	08-JDWBC-1138	LBCG1138-09	HQ648261	Canada	British Columbia	Spencer Entomological Museum, UBC
Eupithecia lachrymosa	08-JDWBC-3456	LBCG3456-09	HQ648273	Canada	British Columbia	Spencer Entomological Museum, UBC
Eupithecia lachrymosa	08-JDWBC-3458	LBCG3458-09	HQ648272	Canada	British Columbia	Spencer Entomological Museum, UBC
Eupithecia lachrymosa	08-JDWBC-3459	LBCG3459-09	HQ648271	Canada	British Columbia	Spencer Entomological Museum, UBC
Eupithecia lachrymosa	08-JDWBC-3460	LBCG3460-09	HQ648270	Canada	British Columbia	Spencer Entomological Museum, UBC
Eupithecia lachrymosa	08-JDWBC-3461	LBCG3461-09	HQ648269	Canada	British Columbia	Spencer Entomological Museum, UBC
Eupithecia lachrymosa	08-JDWBC-3462	LBCG3462-09	HQ648268	Canada	British Columbia	Spencer Entomological Museum, UBC
Eupithecia lachrymosa	08-JDWBC-3466	LBCG3466-09	HQ648267	Canada	British Columbia	Spencer Entomological Museum, UBC
Eupithecia lachrymosa	CNCLEP00035443	GWNC677-07	HQ648262	Canada	British Columbia	Canadian National Collection of Insects, Arachnids and Nematodes
Eupithecia lachrymosa	UASM58004	GWNS459-07	HQ648264	Canada	Alberta	Strickland Museum of Entomology, University of Alberta
Eupithecia lachrymosa	UASM58269	GWNS458-07	HQ648265	Canada	Alberta	Strickland Museum of Entomology, University of Alberta
Eupithecia lachrymosa	UASM59013	GWNS460-07	HQ648263	Canada	Alberta	Strickland Museum of Entomology, University of Alberta
Eupithecia lachrymosa	UASM59036	GWNS457-07	HQ648266	Canada	Alberta	Strickland Museum of Entomology, University of Alberta
Eupithecia lafontaineata	UASM59048	GWNS425-07	HQ648274	Canada	Alberta	Strickland Museum of Entomology, University of Alberta
Eupithecia lariciata	Dun-08-090	DUNLP090-08	HQ648275	Canada	British Columbia	Pacific Forestry Centre, Canadian Forest Service
Eupithecia lariciata	Dun-08-091	DUNLP091-08	HQ648276	Canada	British Columbia	Pacific Forestry Centre, Canadian Forest Service
Eupithecia lariciata	Dun-08-092	DUNLP092-08	HQ648277	Canada	British Columbia	Pacific Forestry Centre, Canadian Forest Service
Eupithecia lariciata	PFC-2007-0252	GWNP045-07	HQ648283	Canada	British Columbia	Pacific Forestry Centre, Canadian Forest Service
Eupithecia lariciata	PFC-2007-0269	GWNP062-07	HQ648282	Canada	British Columbia	Pacific Forestry Centre, Canadian Forest Service
Eupithecia lariciata	PFC-2007-0277	GWNP070-07	HQ648281	Canada	British Columbia	Pacific Forestry Centre, Canadian Forest Service
Eupithecia lariciata	PFC-2007-0278	GWNP071-07	HQ648280	Canada	British Columbia	Pacific Forestry Centre, Canadian Forest Service
Eupithecia lariciata	UASM58235	GWNS430-07	HQ648279	Canada	Alberta	Strickland Museum of Entomology, University of Alberta
Eupithecia lariciata	UASM58236	GWNS431-07	HQ648278	Canada	Alberta	Strickland Museum of Entomology, University of Alberta
Eupithecia longipalpata	CNCLEP00034091	GWNC529-07	HQ648286	Canada	British Columbia	Canadian National Collection of Insects, Arachnids and Nematodes
Eupithecia longipalpata	Dun-08-075	DUNLP075-08	HQ648285	Canada	British Columbia	Pacific Forestry Centre, Canadian Forest Service
Eupithecia longipalpata	Dun-08-080	DUNLP080-08	HQ648284	Canada	British Columbia	Pacific Forestry Centre, Canadian Forest Service
Eupithecia longipalpata	PFC-2007-0256	GWNP049-07	HQ648287	Canada	British Columbia	Pacific Forestry Centre, Canadian Forest Service
Eupithecia maestosa	HLC-23354	LBCD534-05	HQ648288	Canada	British Columbia	Biodiversity Institute of Ontario
Eupithecia maestosa	PFC-2007-0276	GWNP069-07	HQ648289	Canada	British Columbia	Pacific Forestry Centre, Canadian Forest Service
Eupithecia misturata	JECW-07-0179	GWNJ179-07	HQ648294	United States	Washington	James Entomological Collection, Washington State University
Eupithecia misturata	JECW-07-0180	GWNJ180-07	HQ648290	United States	Idaho	James Entomological Collection, Washington State University
Eupithecia misturata	JECW-07-0182	GWNJ182-07	HQ648293	United States	Washington	James Entomological Collection, Washington State University
Eupithecia misturata	UASM59023	GWNS422-07	HQ648292	Canada	Alberta	Strickland Museum of Entomology, University of Alberta
Eupithecia misturata	UASM59050	GWNS423-07	HQ648291	Canada	Alberta	Strickland Museum of Entomology, University of Alberta
Eupithecia mutata	CGWC-1220	LOWCB280-05	HQ648299	Canada	British Columbia	Biodiversity Institute of Ontario
Eupithecia mutata	CGWC-2511	LOWCC631-05	HQ648298	Canada	British Columbia	Biodiversity Institute of Ontario
Eupithecia mutata	CGWC-2563	LOWCC683-05	HQ648297	Canada	British Columbia	Biodiversity Institute of Ontario
Eupithecia mutata	CGWC-2574	LOWCC694-05	HQ648296	Canada	British Columbia	Biodiversity Institute of Ontario
Eupithecia mutata	HLC-23429	LBCD609-05	HQ648295	Canada	British Columbia	Biodiversity Institute of Ontario
Eupithecia nevadata	08-JDWBC-0325	LBCG325-08	HQ648304	Canada	British Columbia	Spencer Entomological Museum, UBC
Eupithecia nevadata	CGWC-4019	LOWCE259-06	HQ648302	Canada	British Columbia	Biodiversity Institute of Ontario
Eupithecia nevadata	CGWC-4024	LOWCE264-06	HQ648301	Canada	British Columbia	Biodiversity Institute of Ontario
Eupithecia nevadata	CGWC-4193	LOWCE433-06	HQ648300	Canada	British Columbia	Biodiversity Institute of Ontario
Eupithecia nevadata	UASM95835	GWNS475-07	HQ648303	Canada	British Columbia	Strickland Museum of Entomology, University of Alberta
Eupithecia nimbicolor	08-JDWBC-0050	LBCG050-08	HQ648307	Canada	British Columbia	Spencer Entomological Museum, UBC
Eupithecia nimbicolor	NFRC-P-2004-005102	GWNN109-07	HQ648305	Canada	Alberta	Northern Forestry Centre, Canadian Forest Service
Eupithecia nimbicolor	UASM58237	GWNS440-07	HQ648306	Canada	Alberta	Strickland Museum of Entomology, University of Alberta
Eupithecia niphadophilata	CNCLEP00035444	GWNC678-07	HQ648309	Canada	Alberta	Canadian National Collection of Insects, Arachnids and Nematodes
Eupithecia niphadophilata	Dun-08-073	DUNLP073-08	HQ648308	Canada	British Columbia	Pacific Forestry Centre, Canadian Forest Service
Eupithecia niphadophilata	PFC-2007-0259	GWNP052-07	HQ648311	Canada	British Columbia	Pacific Forestry Centre, Canadian Forest Service
Eupithecia niphadophilata	UASM59396	GWNS476-07	HQ648310	Canada	Alberta	Strickland Museum of Entomology, University of Alberta
Eupithecia niveifascia	UASM59049	GWNS462-07	HQ648312	Canada	Alberta	Strickland Museum of Entomology, University of Alberta
Eupithecia olivacea	07-JDWBC-0001	GWND001-07	HQ648317	Canada	British Columbia	Royal British Columbia Museum
Eupithecia olivacea	CGWC-4042	LOWCE282-06	HQ648316	Canada	British Columbia	Biodiversity Institute of Ontario
Eupithecia olivacea	CGWC-4044	LOWCE284-06	HQ648315	Canada	British Columbia	Biodiversity Institute of Ontario
Eupithecia olivacea	CGWC-4047	LOWCE287-06	HQ648314	Canada	British Columbia	Biodiversity Institute of Ontario
Eupithecia olivacea	CGWC-4048	LOWCE288-06	HQ648313	Canada	British Columbia	Biodiversity Institute of Ontario
Eupithecia ornata	08-JDWBC-3457	LBCG3457-09	HQ648323	Canada	British Columbia	Spencer Entomological Museum, UBC
Eupithecia ornata	08-JDWBC-3465	LBCG3465-09	HQ648322	Canada	British Columbia	Spencer Entomological Museum, UBC
Eupithecia ornata	08-JDWBC-3467	LBCG3467-09	HQ648321	Canada	British Columbia	Spencer Entomological Museum, UBC
Eupithecia ornata	CNCLEP00034087	GWNC525-07	HQ648319	Canada	British Columbia	Canadian National Collection of Insects, Arachnids and Nematodes
Eupithecia ornata	Dun-08-082	DUNLP082-08	HQ648318	Canada	British Columbia	Pacific Forestry Centre, Canadian Forest Service
Eupithecia ornata	PFC-2007-0255	GWNP048-07	HQ648320	Canada	British Columbia	Pacific Forestry Centre, Canadian Forest Service
Eupithecia palpata	Dun-08-071	DUNLP071-08	HQ648325	Canada	British Columbia	Pacific Forestry Centre, Canadian Forest Service
Eupithecia palpata	Dun-08-083	DUNLP083-08	HQ648324	Canada	British Columbia	Pacific Forestry Centre, Canadian Forest Service
Eupithecia palpata	UASM57999	GWNS418-07	HQ648326	Canada	Alberta	Strickland Museum of Entomology, University of Alberta
Eupithecia perfusca	08-JDWBC-3463	LBCG3463-09	HQ648329	Canada	British Columbia	Spencer Entomological Museum, UBC
Eupithecia perfusca	08-JDWBC-3464	LBCG3464-09	HQ648328	Canada	British Columbia	Spencer Entomological Museum, UBC
Eupithecia perfusca	UASM59037	GWNS452-07	HQ648327	Canada	Alberta	Strickland Museum of Entomology, University of Alberta
Eupithecia placidata	CGWC-2577	LOWCC697-05	HQ648332	Canada	British Columbia	Biodiversity Institute of Ontario
Eupithecia placidata	CGWC-2596	LOWCC716-05	HQ648331	Canada	British Columbia	Biodiversity Institute of Ontario
Eupithecia placidata	CGWC-2597	LOWCC717-05	HQ648330	Canada	British Columbia	Biodiversity Institute of Ontario
Eupithecia placidata	Dun-08-084	DUNLP084-08	HQ648334	Canada	British Columbia	Pacific Forestry Centre, Canadian Forest Service
Eupithecia placidata	Dun-08-085	DUNLP085-08	HQ648333	Canada	British Columbia	Pacific Forestry Centre, Canadian Forest Service

Eupithecia pseudotsugata	CGWC-2523	LOWCC643-05	HQ648337	Canada	British Columbia	Biodiversity Institute of Ontario
Eupithecia pseudotsugata	CGWC-2524	LOWCC644-05	HQ648336	Canada	British Columbia	Biodiversity Institute of Ontario
Eupithecia pseudotsugata	CGWC-2678	LOWCC798-05	HQ648335	Canada	British Columbia	Biodiversity Institute of Ontario
Eupithecia pseudotsugata	UASM95841	GWNS488-07	HQ648339	Canada	British Columbia	Strickland Museum of Entomology, University of Alberta
Eupithecia pseudotsugata	UASM95842	GWNS489-07	HQ648338	Canada	British Columbia	Strickland Museum of Entomology, University of Alberta
Eupithecia pusillata	PFC-2007-0271	GWNP064-07	HQ648340	Canada	British Columbia	Pacific Forestry Centre, Canadian Forest Service
Eupithecia ravocostaliata	UASM57953	GWNS473-07	HQ648342	Canada	Alberta	Strickland Museum of Entomology, University of Alberta
Eupithecia ravocostaliata	UASM58049	GWNS472-07	HQ648343	Canada	Alberta	Strickland Museum of Entomology, University of Alberta
Eupithecia ravocostaliata	UASM7026	GWNS474-07	HQ648341	Canada	Alberta	Strickland Museum of Entomology, University of Alberta
Eupithecia regina	CNCLEP00034102	GWNC540-07	HQ648344	Canada	Saskatchewan	Canadian National Collection of Insects, Arachnids and Nematodes
Eupithecia rotundopuncta	10-GOBCL-07	GOBCL007-10	HQ648345	United States	Washington	Barcode of Life Data System
Eupithecia rotundopuncta	UBC-2007-0034	LBCS050-07	FJ412550	Canada	British Columbia	Canadian National Collection of Insects, Arachnids and Nematodes
Eupithecia rotundopuncta	UBC-2007-0037	LBCS053-07	FJ412549	Canada	British Columbia	Canadian National Collection of Insects, Arachnids and Nematodes
Eupithecia russeliata	CBCC1254	GWNN111-07	HQ648346	Canada	Alberta	Northern Forestry Centre, Canadian Forest Service
Eupithecia russeliata	NFRC-P-2004-005105	GWNN107-07	HQ648347	Canada	Alberta	Northern Forestry Centre, Canadian Forest Service
Eupithecia satyrata	CGWC-2549	LOWCC669-05	HQ648350	Canada	British Columbia	Biodiversity Institute of Ontario
Eupithecia satyrata	CGWC-2602	LOWCC722-05	HQ648349	Canada	British Columbia	Biodiversity Institute of Ontario
Eupithecia satyrata	HLC-20139	LBCA139-05	HQ648348	Canada	British Columbia	Biodiversity Institute of Ontario
Eupithecia satyrata	UASM53003	GWNS437-07	HQ648351	Canada	Alberta	Strickland Museum of Entomology, University of Alberta
Eupithecia satyrata	UASM53004	GWNS584-07	HQ648352	Canada	Alberta	Strickland Museum of Entomology, University of Alberta
Eupithecia sharronata	HLC-23390	LBCD570-05	HQ648353	Canada	British Columbia	Biodiversity Institute of Ontario
Eupithecia sharronata	UASM53001	GWNS432-07	HQ648354	Canada	Alberta	Strickland Museum of Entomology, University of Alberta
Eupithecia sp. 1	UASM57384	GWNS451-07	HQ648355	Canada	Alberta	Strickland Museum of Entomology, University of Alberta
Eupithecia sp. 1	UASM59012	GWNS450-07	HQ648356	Canada	Alberta	Strickland Museum of Entomology, University of Alberta
Eupithecia sp. 11	CGWC-2556	LOWCC676-05	HQ648360	Canada	British Columbia	Biodiversity Institute of Ontario
Eupithecia sp. 11	CGWC-2571	LOWCC691-05	HQ648359	Canada	British Columbia	Biodiversity Institute of Ontario
Eupithecia sp. 11	CGWC-2629	LOWCC749-05	HQ648358	Canada	British Columbia	Biodiversity Institute of Ontario
Eupithecia sp. 11	CGWC-2647	LOWCC767-05	HQ648357	Canada	British Columbia	Biodiversity Institute of Ontario
Eupithecia sp. 22	CGWC-1187	LOWCB247-05	HQ648365	Canada	British Columbia	Biodiversity Institute of Ontario
Eupithecia sp. 22	CGWC-1191	LOWCB251-05	HQ648364	Canada	British Columbia	Biodiversity Institute of Ontario
Eupithecia sp. 22	CGWC-1195	LOWCB255-05	HQ648363	Canada	British Columbia	Biodiversity Institute of Ontario
Eupithecia sp. 22	CGWC-1198	LOWCB258-05	HQ648362	Canada	British Columbia	Biodiversity Institute of Ontario
Eupithecia sp. 22	CGWC-1207	LOWCB267-05	HQ648361	Canada	British Columbia	Biodiversity Institute of Ontario
Eupithecia sp. 4	UASM57329	GWNS433-07	HQ648368	Canada	Alberta	Strickland Museum of Entomology, University of Alberta
Eupithecia sp. 4	UASM59141	GWNS454-07	HQ648366	Canada	Alberta	Strickland Museum of Entomology, University of Alberta
Eupithecia sp. 4	UASM59156	GWNS453-07	HQ648367	Canada	Alberta	Strickland Museum of Entomology, University of Alberta
Eupithecia spermaphaga	CNCLEP00035453	GWNC687-07	HQ648369	Canada	British Columbia	Canadian National Collection of Insects, Arachnids and Nematodes
Eupithecia spermaphaga	PFC-2007-0264	GWNP057-07	HQ648371	Canada	British Columbia	Pacific Forestry Centre, Canadian Forest Service
Eupithecia spermaphaga	PFC-2007-0265	GWNP058-07	HQ648370	Canada	British Columbia	Pacific Forestry Centre, Canadian Forest Service
Eupithecia stellata	NFRC-P-2003-000227	GWNN091-07	HQ648373	Canada	Alberta	Northern Forestry Centre, Canadian Forest Service
Eupithecia stellata	UASM58008	GWNS461-07	HQ648372	Canada	Alberta	Strickland Museum of Entomology, University of Alberta
Eupithecia subfuscata	CBCC1253	GWNN112-07	HQ648376	Canada	Alberta	Northern Forestry Centre, Canadian Forest Service
Eupithecia subfuscata	CBCC1258	GWNN113-07	HQ648375	Canada	Alberta	Northern Forestry Centre, Canadian Forest Service
Eupithecia subfuscata	JECW-07-0173	GWNJ173-07	HQ648384	United States	Washington	James Entomological Collection, Washington State University
Eupithecia subfuscata	NFRC-P-2003-000745	GWNN097-07	HQ648377	Canada	Alberta	Northern Forestry Centre, Canadian Forest Service
Eupithecia subfuscata	PFC-2007-0250	GWNP043-07	HQ648383	Canada	British Columbia	Pacific Forestry Centre, Canadian Forest Service
Eupithecia subfuscata	UASM59017	GWNS466-07	HQ648374	Canada	Alberta	Strickland Museum of Entomology, University of Alberta
Eupithecia subfuscata	UASM59019	GWNS428-07	HQ648380	Canada	Alberta	Strickland Museum of Entomology, University of Alberta
Eupithecia subfuscata	UASM59020	GWNS427-07	HQ648381	Canada	Alberta	Strickland Museum of Entomology, University of Alberta
Eupithecia subfuscata	UASM59021	GWNS429-07	HQ648379	Canada	Alberta	Strickland Museum of Entomology, University of Alberta
Eupithecia subfuscata	UASM78503	GWNS426-07	HQ648382	Canada	Alberta	Strickland Museum of Entomology, University of Alberta
Eupithecia subfuscata	UASM95840	GWNS492-07	HQ648378	Canada	Alberta	Strickland Museum of Entomology, University of Alberta
Eupithecia tenuata	CNCLEP00035447	GWNC681-07	HQ648385	United States	Colorado	Canadian National Collection of Insects, Arachnids and Nematodes
Eupithecia tripunctaria	HLC-20264	LBCA264-05	HQ648389	Canada	British Columbia	Biodiversity Institute of Ontario
Eupithecia tripunctaria	HLC-20730	LBCA730-05	HQ648388	Canada	British Columbia	Biodiversity Institute of Ontario
Eupithecia tripunctaria	HLC-21405	LBCB465-05	HQ648387	Canada	British Columbia	Biodiversity Institute of Ontario
Eupithecia tripunctaria	HLC-23361	LBCD541-05	HQ648386	Canada	British Columbia	Biodiversity Institute of Ontario
Eupithecia unicolor	Dun-08-093	DUNLP093-08	HQ648390	Canada	British Columbia	Pacific Forestry Centre, Canadian Forest Service
Eurhinosea flavaria	CNCLEP00033424	GWNC425-07	HQ648391	Canada	British Columbia	Canadian National Collection of Insects, Arachnids and Nematodes
Eustroma atrifasciata	08-JDWBC-0108	LBCG108-08	HQ648392	Canada	British Columbia	Spencer Entomological Museum, UBC
Eustroma atrifasciata	CNCLEP00033427	GWNC428-07	FJ376631	Canada	British Columbia	Canadian National Collection of Insects, Arachnids and Nematodes
Eustroma fasciata	CNCLEP00033426	GWNC427-07	FJ376632	United States	Washington	Canadian National Collection of Insects, Arachnids and Nematodes
Eustroma fasciata	ENT991-006370	GWNR465-07	HQ648396	Canada	British Columbia	Royal British Columbia Museum
Eustroma fasciata	ENT996-004411	GWNR466-07	HQ648395	Canada	British Columbia	Royal British Columbia Museum
Eustroma fasciata	ENT996-004415	GWNR467-07	HQ648394	United States	Washington	Royal British Columbia Museum
Eustroma fasciata	HLC-22239	LBCC359-05	HQ648393	Canada	British Columbia	Biodiversity Institute of Ontario
Eustroma semiatrata	08-JDWBC-0741	LBCG741-09	HQ648401	Canada	British Columbia	Spencer Entomological Museum, UBC
Eustroma semiatrata	08-JDWBC-1835	LBCG1835-09	HQ648403	Canada	British Columbia	Spencer Entomological Museum, UBC
Eustroma semiatrata	08-JDWBC-1836	LBCG1836-09	HQ648402	Canada	British Columbia	Spencer Entomological Museum, UBC
Eustroma semiatrata	08-JDWBC-2116	LBCG2116-09	HQ648405	Canada	British Columbia	Spencer Entomological Museum, UBC
Eustroma semiatrata	08-JDWBC-3691	LBCG3691-09	HQ648404	Canada	British Columbia	Royal British Columbia Museum
Eustroma semiatrata	CNCLEP00033311	GWNC312-07	HQ648397	Canada	Alberta	Canadian National Collection of Insects, Arachnids and Nematodes
Eustroma semiatrata	JECW-07-0135	GWNJ135-07	HQ648398	United States	Washington	James Entomological Collection, Washington State University
Eustroma semiatrata	UASM41389	GWNS310-07	HQ648400	Canada	Alberta	Strickland Museum of Entomology, University of Alberta
Eustroma semiatrata	UASM77890	GWNS311-07	HQ648399	Canada	Alberta	Strickland Museum of Entomology, University of Alberta
Gabriola dyari	08-JDWBC-0201	LBCG201-08	HQ648412	Canada	British Columbia	Spencer Entomological Museum, UBC
Gabriola dyari	08-JDWBC-2316	LBCG2316-09	HQ648413	Canada	British Columbia	Spencer Entomological Museum, UBC
Gabriola dyari	08-JDWBC-2317	LBCG2317-09	HQ648414	Canada	British Columbia	Spencer Entomological Museum, UBC
Gabriola dyari	Dun-08-095	DUNLP095-08	HQ648406	Canada	British Columbia	Pacific Forestry Centre, Canadian Forest Service
Gabriola dyari	ENT996-003894	GWNR218-07	HQ648419	Canada	British Columbia	Royal British Columbia Museum
Gabriola dyari	ENT996-003895	GWNR222-07	HQ648415	Canada	British Columbia	Royal British Columbia Museum
Gabriola dyari	ENT996-003896	GWNR221-07	HQ648416	Canada	British Columbia	Royal British Columbia Museum
Gabriola dyari	ENT996-003898	GWNR219-07	HQ648418	Canada	British Columbia	Royal British Columbia Museum
Gabriola dyari	ENT996-003904	GWNR220-07	HQ648417	Canada	British Columbia	Royal British Columbia Museum
Gabriola dyari	JD1917	GWNS152-07	HQ648408	Canada	British Columbia	Strickland Museum of Entomology, University of Alberta
Gabriola dyari	JECW-07-0074	GWNJ074-07	HQ648407	United States	Washington	James Entomological Collection, Washington State University
Gabriola dyari	PFC-2007-0383	GWNP173-07	HQ648411	Canada	British Columbia	Pacific Forestry Centre, Canadian Forest Service
Gabriola dyari	WFBM-07-0050	GWNW050-07	HQ648410	United States	Idaho	University of Idaho, W. F. Barr Entomological Collection
Gabriola dyari	WFBM-07-0051	GWNW051-07	HQ648409	United States	Idaho	University of Idaho, W. F. Barr Entomological Collection
Glaucina nephos	WFBM-07-0024	GWNW024-07	HQ648420	United States	Idaho	University of Idaho, W. F. Barr Entomological Collection
Glena nigricaria	08-JDWBC-0411	LBCG411-08	HQ648425	Canada	British Columbia	Spencer Entomological Museum, UBC
Glena nigricaria	08-JDWBC-0417	LBCG417-08	HQ648424	Canada	British Columbia	Spencer Entomological Museum, UBC
Glena nigricaria	CGWC-0982	LOWCB042-05	HQ648422	Canada	British Columbia	Biodiversity Institute of Ontario
Glena nigricaria	Dun-08-096	DUNLP096-08	HQ648421	Canada	British Columbia	Pacific Forestry Centre, Canadian Forest Service
Glena nigricaria	ENT996-003448	GWNR093-07	HQ648423	Canada	British Columbia	Royal British Columbia Museum
Gnophos macguffini	CNCLEP00033103	GWNC104-07	HQ648430	Canada	Yukon Territory	Canadian National Collection of Insects, Arachnids and Nematodes
Gnophos macguffini	CNCLEP00033104	GWNC105-07	HQ648429	Canada	Yukon Territory	Canadian National Collection of Insects, Arachnids and Nematodes
Gnophos macguffini	CNCLEP00033105	GWNC106-07	HQ648428	Canada	British Columbia	Canadian National Collection of Insects, Arachnids and Nematodes
Gnophos macguffini	CNCLEP00033308	GWNC309-07	HQ648427	Canada	British Columbia	Canadian National Collection of Insects, Arachnids and Nematodes
Gnophos macguffini	CNCLEP00033309	GWNC310-07	HQ648426	Canada	British Columbia	Canadian National Collection of Insects, Arachnids and Nematodes
Gnophos macguffini	SEM-UBC-GEO-0094	GWNU052-07	HQ648431	Canada	Yukon Territory	Spencer Entomological Museum, UBC
Haematopis grataria	CNCLEP00033334	GWNC335-07	HQ648432	Canada	Alberta	Canadian National Collection of Insects, Arachnids and Nematodes
Haematopis grataria	UASM41553	GWNS249-07	HQ648434	Canada	Alberta	Strickland Museum of Entomology, University of Alberta
Haematopis grataria	UASM58991	GWNS250-07	HQ648433	Canada	Alberta	Strickland Museum of Entomology, University of Alberta
Hemithea aestivaria	CNCLEP00021545	GWNC459-07	HQ648438	Canada	British Columbia	Canadian National Collection of Insects, Arachnids and Nematodes
Hemithea aestivaria	CNCLEP00021546	GWNC460-07	HQ648437	Canada	British Columbia	Canadian National Collection of Insects, Arachnids and Nematodes
Hemithea aestivaria	CNCLEP00021547	GWNC461-07	HQ648436	Canada	British Columbia	Canadian National Collection of Insects, Arachnids and Nematodes

Hemithea aestivaria	Dun-08-097	DUNLP097-08	HQ648435	Canada	British Columbia	Pacific Forestry Centre, Canadian Forest Service
Hemithea aestivaria	ENT006-000228	GWNR350-07	HQ648447	Canada	British Columbia	Royal British Columbia Museum
Hemithea aestivaria	ENT996-004235	GWNR349-07	HQ648448	Canada	British Columbia	Royal British Columbia Museum
Hemithea aestivaria	PFC-2007-0214	GWNP007-07	HQ648446	Canada	British Columbia	Pacific Forestry Centre, Canadian Forest Service
Hemithea aestivaria	PFC-2007-0215	GWNP008-07	HQ648445	Canada	British Columbia	Pacific Forestry Centre, Canadian Forest Service
Hemithea aestivaria	PFC-2007-0216	GWNP009-07	HQ648444	Canada	British Columbia	Pacific Forestry Centre, Canadian Forest Service
Hemithea aestivaria	PFC-2007-0217	GWNP010-07	HQ648443	Canada	British Columbia	Pacific Forestry Centre, Canadian Forest Service
Hemithea aestivaria	PFC-2007-0218	GWNP011-07	HQ648442	Canada	British Columbia	Pacific Forestry Centre, Canadian Forest Service
Hemithea aestivaria	PFC-2007-0220	GWNP013-07	HQ648441	Canada	British Columbia	Pacific Forestry Centre, Canadian Forest Service
Hemithea aestivaria	PFC-2007-0221	GWNP014-07	HQ648440	Canada	British Columbia	Pacific Forestry Centre, Canadian Forest Service
Hemithea aestivaria	UASM59768	GWNS242-07	HQ648439	Canada	British Columbia	Strickland Museum of Entomology, University of Alberta
Hesperumia latipennis	JECW-07-0031	GWNJ031-07	HQ648450	United States	Washington	James Entomological Collection, Washington State University
Hesperumia latipennis	JECW-07-0032	GWNJ032-07	HQ648449	United States	Washington	James Entomological Collection, Washington State University
Hesperumia sulphuraria	08-JDWBC-1097	LBCG1097-09	HQ648457	Canada	British Columbia	Spencer Entomological Museum, UBC
Hesperumia sulphuraria	CNCLEP00033086	GWNC087-07	HQ648451	United States	Washington	Canadian National Collection of Insects, Arachnids and Nematodes
Hesperumia sulphuraria	ENT990-000149	GWNR096-07	HQ648456	Canada	British Columbia	Royal British Columbia Museum
Hesperumia sulphuraria	JECW-07-0033	GWNJ033-07	HQ648453	United States	Washington	James Entomological Collection, Washington State University
Hesperumia sulphuraria	JECW-07-0035	GWNJ035-07	HQ648452	United States	Washington	James Entomological Collection, Washington State University
Hesperumia sulphuraria	UASM41307	GWNS068-07	HQ648455	Canada	Alberta	Strickland Museum of Entomology, University of Alberta
Hesperumia sulphuraria	UASM57100	GWNS069-07	HQ648454	Canada	Alberta	Strickland Museum of Entomology, University of Alberta
Horisme incana	ENT002-001756	GWNR604-07	HQ648462	Canada	British Columbia	Royal British Columbia Museum
Horisme incana	ENT002-001757	GWNR605-07	HQ648461	Canada	British Columbia	Royal British Columbia Museum
Horisme incana	JD0189	GWNS417-07	HQ648458	Canada	Alberta	Strickland Museum of Entomology, University of Alberta
Horisme incana	UASM57012	GWNS416-07	HQ648459	Canada	Alberta	Strickland Museum of Entomology, University of Alberta
Horisme incana	UASM58238	GWNS415-07	HQ648460	Canada	Alberta	Strickland Museum of Entomology, University of Alberta
Horisme intestinata	ENT002-001755	GWNR608-07	HQ648466	Canada	British Columbia	Royal British Columbia Museum
Horisme intestinata	ENT996-005396	GWNR607-07	HQ648467	Canada	British Columbia	Royal British Columbia Museum
Horisme intestinata	UASM58105	GWNS413-07	HQ648464	Canada	Alberta	Strickland Museum of Entomology, University of Alberta
Horisme intestinata	UASM58106	GWNS412-07	HQ648465	Canada	Alberta	Strickland Museum of Entomology, University of Alberta
Horisme intestinata	UASM7307	GWNS414-07	HQ648463	Canada	Alberta	Strickland Museum of Entomology, University of Alberta
Hulstina formosata	JECW-07-0041	GWNJ041-07	HQ648469	United States	Idaho	James Entomological Collection, Washington State University
Hulstina formosata	JECW-07-0042	GWNJ042-07	HQ648468	United States	Idaho	James Entomological Collection, Washington State University
Hulstina xera	JECW-07-0043	GWNJ043-07	HQ648470	United States	Washington	James Entomological Collection, Washington State University
Hydrelia albifera	HLC-20012	LBCA012-05	HQ648472	Canada	British Columbia	Biodiversity Institute of Ontario
Hydrelia albifera	HLC-23472	LBCD652-05	HQ648471	Canada	British Columbia	Biodiversity Institute of Ontario
Hydrelia albifera	PFC-2007-0336	GWNP126-07	HQ648475	Canada	British Columbia	Pacific Forestry Centre, Canadian Forest Service
Hydrelia albifera	PFC-2007-0337	GWNP127-07	HQ648474	Canada	British Columbia	Pacific Forestry Centre, Canadian Forest Service
Hydrelia albifera	UASM58127	GWNS399-07	HQ648473	Canada	Alberta	Strickland Museum of Entomology, University of Alberta
Hydriomena albifasciata	10-GOBCL-03	GOBCL003-10	HQ648478	United States	California	Barcode of Life Data System
Hydriomena albifasciata	10-GOBCL-04	GOBCL004-10	HQ648477	United States	California	Barcode of Life Data System
Hydriomena albifasciata	10-GOBCL-05	GOBCL005-10	HQ648476	United States	California	Barcode of Life Data System
Hydriomena albimontanata	CNCLEP00033312	GWNC313-07	HQ648480	Canada	Alberta	Canadian National Collection of Insects, Arachnids and Nematodes
Hydriomena albimontanata	CNCLEP00033313	GWNC314-07	HQ648479	Canada	British Columbia	Canadian National Collection of Insects, Arachnids and Nematodes
Hydriomena californiata	CNCLEP00033445	GWNC446-07	HQ648482	Canada	British Columbia	Canadian National Collection of Insects, Arachnids and Nematodes
Hydriomena californiata	Dun-08-105	DUNLP105-08	HQ648481	Canada	British Columbia	Pacific Forestry Centre, Canadian Forest Service
Hydriomena californiata	ENT991-065529	GWNR430-07	HQ648484	Canada	British Columbia	Royal British Columbia Museum
Hydriomena californiata	JECW-07-0147	GWNJ147-07	HQ648483	United States	Washington	James Entomological Collection, Washington State University
Hydriomena crokeri	CNCLEP00033446	GWNC447-07	HQ648485	United States	Oregon	Canadian National Collection of Insects, Arachnids and Nematodes
Hydriomena divisaria	CGWC-2734	LOWCC854-05	HQ648487	Canada	British Columbia	Biodiversity Institute of Ontario
Hydriomena divisaria	Dun-08-099	DUNLP099-08	HQ648488	Canada	British Columbia	Pacific Forestry Centre, Canadian Forest Service
Hydriomena divisaria	ENT996-007707	GWNR626-07	HQ648490	Canada	British Columbia	Royal British Columbia Museum
Hydriomena divisaria	HLC-20399	LBCA399-05	HQ648486	Canada	British Columbia	Biodiversity Institute of Ontario
Hydriomena divisaria	UASM78450	GWNS574-07	HQ648489	Canada	Alberta	Strickland Museum of Entomology, University of Alberta
Hydriomena edenata	ENT991-125451	GWNR481-07	HQ648493	Canada	British Columbia	Royal British Columbia Museum
Hydriomena edenata	JECW-07-0139	GWNJ139-07	HQ648492	United States	Washington	James Entomological Collection, Washington State University
Hydriomena edenata	JECW-07-0140	GWNJ140-07	HQ648491	United States	Washington	James Entomological Collection, Washington State University
Hydriomena exculpata	HLC-20291	LBCA291-05	HQ648498	Canada	British Columbia	Biodiversity Institute of Ontario
Hydriomena exculpata	HLC-20314	LBCA314-05	HQ648497	Canada	British Columbia	Biodiversity Institute of Ontario
Hydriomena exculpata	HLC-20721	LBCA721-05	HQ648496	Canada	British Columbia	Biodiversity Institute of Ontario
Hydriomena exculpata	HLC-20866	LBCA866-05	HQ648495	Canada	British Columbia	Biodiversity Institute of Ontario
Hydriomena exculpata	HLC-21372	LBCB432-05	HQ648494	Canada	British Columbia	Biodiversity Institute of Ontario
Hydriomena furcata	08-JDWBC-0774	LBCG774-09	HQ648518	Canada	British Columbia	Spencer Entomological Museum, UBC
Hydriomena furcata	08-JDWBC-0778	LBCG778-09	HQ648517	Canada	British Columbia	Spencer Entomological Museum, UBC
Hydriomena furcata	08-JDWBC-0780	LBCG780-09	HQ648516	Canada	British Columbia	Spencer Entomological Museum, UBC
Hydriomena furcata	08-JDWBC-0781	LBCG781-09	HQ648515	Canada	British Columbia	Spencer Entomological Museum, UBC
Hydriomena furcata	08-JDWBC-0793	LBCG793-09	HQ648514	Canada	British Columbia	Spencer Entomological Museum, UBC
Hydriomena furcata	08-JDWBC-0951	LBCG951-09	HQ648513	Canada	British Columbia	Spencer Entomological Museum, UBC
Hydriomena furcata	08-JDWBC-0992	LBCG992-09	HQ648512	Canada	British Columbia	Spencer Entomological Museum, UBC
Hydriomena furcata	08-JDWBC-1003	LBCG1003-09	HQ648511	Canada	British Columbia	Spencer Entomological Museum, UBC
Hydriomena furcata	08-JDWBC-1005	LBCG1005-09	HQ648510	Canada	British Columbia	Spencer Entomological Museum, UBC
Hydriomena furcata	08-JDWBC-1008	LBCG1008-09	HQ648509	Canada	British Columbia	Spencer Entomological Museum, UBC
Hydriomena furcata	08-JDWBC-2319	LBCG2319-09	HQ648519	Canada	British Columbia	Spencer Entomological Museum, UBC
Hydriomena furcata	08-JDWBC-2320	LBCG2320-09	HQ648520	Canada	British Columbia	Spencer Entomological Museum, UBC
Hydriomena furcata	08-JDWBC-2321	LBCG2321-09	HQ648521	Canada	British Columbia	Spencer Entomological Museum, UBC
Hydriomena furcata	08-JDWBC-2322	LBCG2322-09	HQ648522	Canada	British Columbia	Spencer Entomological Museum, UBC
Hydriomena furcata	08-JDWBC-2327	LBCG2327-09	HQ648523	Canada	British Columbia	Spencer Entomological Museum, UBC
Hydriomena furcata	ENT002-001703	GWNR476-07	HQ648506	Canada	British Columbia	Royal British Columbia Museum
Hydriomena furcata	ENT991-009949	GWNR480-07	HQ648505	Canada	British Columbia	Royal British Columbia Museum
Hydriomena furcata	ENT992-021183	GWNR475-07	HQ648507	Canada	British Columbia	Royal British Columbia Museum
Hydriomena furcata	ENT996-004361	GWNR474-07	HQ648508	Canada	British Columbia	Royal British Columbia Museum
Hydriomena furcata	UASM58896	GWNS346-07	HQ648502	Canada	Alberta	Strickland Museum of Entomology, University of Alberta
Hydriomena furcata	UASM58897	GWNS345-07	HQ648503	Canada	Alberta	Strickland Museum of Entomology, University of Alberta
Hydriomena furcata	UASM7289	GWNS348-07	HQ648500	Canada	Alberta	Strickland Museum of Entomology, University of Alberta
Hydriomena furcata	UASM7331	GWNS347-07	HQ648501	Canada	Alberta	Strickland Museum of Entomology, University of Alberta
Hydriomena furcata	UASM95813	GWNS334-07	HQ648499	Canada	British Columbia	Strickland Museum of Entomology, University of Alberta
Hydriomena furcata	UASM95853	GWNS517-07	HQ648504	Canada	Alberta	Strickland Museum of Entomology, University of Alberta
Hydriomena irata	Dun-08-101	DUNLP101-08	HQ648525	Canada	British Columbia	Pacific Forestry Centre, Canadian Forest Service
Hydriomena irata	Dun-08-102	DUNLP102-08	HQ648524	Canada	British Columbia	Pacific Forestry Centre, Canadian Forest Service
Hydriomena irata	Dun-08-103	DUNLP103-08	HQ648526	Canada	British Columbia	Pacific Forestry Centre, Canadian Forest Service
Hydriomena irata	PFC-2007-0300	GWNP093-07	HQ648530	Canada	British Columbia	Pacific Forestry Centre, Canadian Forest Service
Hydriomena irata	PFC-2007-0301	GWNP094-07	HQ648529	Canada	British Columbia	Pacific Forestry Centre, Canadian Forest Service
Hydriomena irata	PFC-2007-0313	GWNP103-07	HQ648528	Canada	British Columbia	Pacific Forestry Centre, Canadian Forest Service
Hydriomena irata	PFC-2007-0315	GWNP105-07	HQ648527	Canada	British Columbia	Pacific Forestry Centre, Canadian Forest Service
Hydriomena macdunnoughi	CNCLEP00033406	GWNC407-07	HQ648533	Canada	Alberta	Canadian National Collection of Insects, Arachnids and Nematodes
Hydriomena macdunnoughi	CNCLEP00033447	GWNC448-07	HQ648532	Canada	Yukon Territory	Canadian National Collection of Insects, Arachnids and Nematodes
Hydriomena macdunnoughi	CNCLEP00033449	GWNC450-07	HQ648531	Canada	British Columbia	Canadian National Collection of Insects, Arachnids and Nematodes
Hydriomena manzanita	JECW-07-0141	GWNJ141-07	HQ648534	Canada	British Columbia	James Entomological Collection, Washington State University
Hydriomena manzanita	PFC-2007-0298	GWNP091-07	HQ648536	Canada	British Columbia	Pacific Forestry Centre, Canadian Forest Service
Hydriomena manzanita	PFC-2007-0299	GWNP092-07	HQ648535	Canada	British Columbia	Pacific Forestry Centre, Canadian Forest Service
Hydriomena marinata	Dun-08-098	DUNLP098-08	HQ648538	Canada	British Columbia	Pacific Forestry Centre, Canadian Forest Service
Hydriomena marinata	Dun-08-106	DUNLP106-08	HQ648537	Canada	British Columbia	Pacific Forestry Centre, Canadian Forest Service
Hydriomena marinata	UASM59747	GWNS343-07	HQ648539	Canada	British Columbia	Strickland Museum of Entomology, University of Alberta
Hydriomena morosata	CGWC-3108	LOWCD288-06	HQ648540	Canada	British Columbia	Biodiversity Institute of Ontario
Hydriomena morosata	CNCLEP00033407	GWNC408-07	HQ648541	Canada	Alberta	Canadian National Collection of Insects, Arachnids and Nematodes
Hydriomena morosata	ENT996-007729	GWNR630-07	HQ648543	Canada	British Columbia	Royal British Columbia Museum
Hydriomena morosata	ENT996-007733	GWNR629-07	HQ648544	Canada	British Columbia	Royal British Columbia Museum
Hydriomena morosata	UASM95816	GWNS349-07	HQ648542	Canada	Alberta	Strickland Museum of Entomology, University of Alberta

Hydriomena nevadae	CNCLEP00033444	GWNC445-07	HQ648545	United States	Washington	Canadian National Collection of Insects, Arachnids and Nematodes
Hydriomena nubilofasciata	CNCLEP00034036	GWNC474-07	HQ648546	United States	Washington	Canadian National Collection of Insects, Arachnids and Nematodes
Hydriomena nubilofasciata	PFC-2007-0297	GWNP090-07	HQ648547	Canada	British Columbia	Pacific Forestry Centre, Canadian Forest Service
Hydriomena perfracta	CNCLEP00033437	GWNC438-07	HQ648549	Canada	British Columbia	Canadian National Collection of Insects, Arachnids and Nematodes
Hydriomena perfracta	HLC-20242	LBCA242-05	HQ648548	Canada	British Columbia	Biodiversity Institute of Ontario
Hydriomena perfracta	NFRC-P-2007-100056	GWNN056-07	HQ648551	Canada	Alberta	Northern Forestry Centre, Canadian Forest Service
Hydriomena perfracta	UASM7161	GWNS342-07	HQ648550	Canada	Alberta	Strickland Museum of Entomology, University of Alberta
Hydriomena quinquefasciata	CNCLEP00034024	GWNC462-07	HQ648552	Canada	British Columbia	Canadian National Collection of Insects, Arachnids and Nematodes
Hydriomena renunciata	CNCLEP00033299	GWNC300-07	HQ648553	Canada	British Columbia	Canadian National Collection of Insects, Arachnids and Nematodes
Hydriomena renunciata	ENT987-000324	GWNR473-07	HQ648560	Canada	British Columbia	Royal British Columbia Museum
Hydriomena renunciata	ENT996-004488	GWNR477-07	HQ648559	Canada	British Columbia	Royal British Columbia Museum
Hydriomena renunciata	ENT996-004501	GWNR478-07	HQ648558	Canada	British Columbia	Royal British Columbia Museum
Hydriomena renunciata	JECW-07-0144	GWNJ144-07	HQ648554	Canada	British Columbia	James Entomological Collection, Washington State University
Hydriomena renunciata	NFRC-P-2007-100058	GWNN058-07	HQ648556	Canada	Alberta	Northern Forestry Centre, Canadian Forest Service
Hydriomena renunciata	PFC-2007-0306	GWNP096-07	HQ648555	Canada	British Columbia	Pacific Forestry Centre, Canadian Forest Service
Hydriomena renunciata	UASM78451	GWNS575-07	HQ648557	Canada	Alberta	Strickland Museum of Entomology, University of Alberta
Hydriomena ruberata	CNCLEP00033405	GWNC406-07	HQ648563	Canada	Alberta	Canadian National Collection of Insects, Arachnids and Nematodes
Hydriomena ruberata	CNCLEP00033441	GWNC442-07	HQ648561	Canada	British Columbia	Canadian National Collection of Insects, Arachnids and Nematodes
Hydriomena ruberata	CNCLEP00033448	GWNC449-07	HQ648562	Canada	Yukon Territory	Canadian National Collection of Insects, Arachnids and Nematodes
Hydriomena ruberata	ENT996-007708	GWNR627-07	HQ648568	Canada	British Columbia	Royal British Columbia Museum
Hydriomena ruberata	ENT996-007709	GWNR628-07	HQ648567	Canada	British Columbia	Royal British Columbia Museum
Hydriomena ruberata	NFRC-P-2007-100059	GWNN059-07	HQ648566	Canada	Alberta	Northern Forestry Centre, Canadian Forest Service
Hydriomena ruberata	NFRC-P-2007-100060	GWNN060-07	HQ648565	Canada	Alberta	Northern Forestry Centre, Canadian Forest Service
Hydriomena ruberata	NFRC-P-2007-100061	GWNN061-07	HQ648564	Canada	Alberta	Northern Forestry Centre, Canadian Forest Service
Hydriomena sp.	Dun-08-104	DUNLP104-08	HQ648569	Canada	British Columbia	Pacific Forestry Centre, Canadian Forest Service
Hydriomena speciosata	Dun-08-108	DUNLP108-08	HQ648571	Canada	British Columbia	Pacific Forestry Centre, Canadian Forest Service
Hydriomena speciosata	Dun-08-109	DUNLP109-08	HQ648570	Canada	British Columbia	Pacific Forestry Centre, Canadian Forest Service
Hypagyrtis piniata	Dun-08-110	DUNLP110-08	HQ648575	Canada	British Columbia	Pacific Forestry Centre, Canadian Forest Service
Hypagyrtis piniata	HLC-20862	LBCA862-05	HQ648574	Canada	British Columbia	Biodiversity Institute of Ontario
Hypagyrtis piniata	HLC-21202	LBCB262-05	HQ648573	Canada	British Columbia	Biodiversity Institute of Ontario
Hypagyrtis piniata	HLC-22053	LBCC173-05	HQ648572	Canada	British Columbia	Biodiversity Institute of Ontario
Hypagyrtis piniata	UASM95874	GWNS545-07	HQ648576	Canada	Alberta	Strickland Museum of Entomology, University of Alberta
Hypagyrtis unipunctata	CGWC-0744	LOWC744-05	HQ648580	Canada	British Columbia	Biodiversity Institute of Ontario
Hypagyrtis unipunctata	CGWC-0765	LOWC765-05	HQ648579	Canada	British Columbia	Biodiversity Institute of Ontario
Hypagyrtis unipunctata	HLC-20836	LBCA836-05	HQ648578	Canada	British Columbia	Biodiversity Institute of Ontario
Hypagyrtis unipunctata	HLC-21656	LBCB716-05	HQ648577	Canada	British Columbia	Biodiversity Institute of Ontario
Hypagyrtis unipunctata	UASM41631	GWNS096-07	HQ648581	Canada	Alberta	Strickland Museum of Entomology, University of Alberta
Idaea demissaria	ENT996-007631	GWNR619-07	HQ648586	Canada	British Columbia	Royal British Columbia Museum
Idaea demissaria	ENT996-007632	GWNR620-07	HQ648585	Canada	British Columbia	Royal British Columbia Museum
Idaea demissaria	ENT996-007633	GWNR621-07	HQ648584	Canada	British Columbia	Royal British Columbia Museum
Idaea demissaria	JECW-07-0129	GWNJ129-07	HQ648582	United States	Washington	James Entomological Collection, Washington State University
Idaea demissaria	PFC-2007-0223	GWNP016-07	HQ648583	Canada	British Columbia	Pacific Forestry Centre, Canadian Forest Service
Idaea dimidiata	CNCLEP00033260	GWNC261-07	HQ648588	Canada	British Columbia	Canadian National Collection of Insects, Arachnids and Nematodes
Idaea dimidiata	CNCLEP00033261	GWNC262-07	HQ648587	Canada	British Columbia	Canadian National Collection of Insects, Arachnids and Nematodes
Idaea dimidiata	ENT996-004267	GWNR379-07	HQ648590	United States	Washington	Royal British Columbia Museum
Idaea dimidiata	ENT996-004277	GWNR377-07	HQ648592	Canada	British Columbia	Royal British Columbia Museum
Idaea dimidiata	ENT996-004280	GWNR378-07	HQ648591	Canada	British Columbia	Royal British Columbia Museum
Idaea dimidiata	PFC-2007-0222	GWNP015-07	HQ648589	Canada	British Columbia	Pacific Forestry Centre, Canadian Forest Service
Idaea rotundopennata	NFRC-P-2007-100040	GWNN040-07	HQ648595	Canada	Alberta	Northern Forestry Centre, Canadian Forest Service
Idaea rotundopennata	NFRC-P-2007-100041	GWNN041-07	HQ648594	Canada	Alberta	Northern Forestry Centre, Canadian Forest Service
Idaea rotundopennata	NFRC-P-2007-100042	GWNN042-07	HQ648593	Canada	Alberta	Northern Forestry Centre, Canadian Forest Service
Iridopsis clivinaria	CNCLEP00033098	GWNC099-07	HQ648596	Canada	British Columbia	Canadian National Collection of Insects, Arachnids and Nematodes
Iridopsis clivinaria	ENT996-003519	GWNR079-07	HQ648599	United States	Washington	Royal British Columbia Museum
Iridopsis clivinaria	JECW-07-0044	GWNJ044-07	HQ648598	United States	Washington	James Entomological Collection, Washington State University
Iridopsis clivinaria	JECW-07-0045	GWNJ045-07	HQ648597	United States	Washington	James Entomological Collection, Washington State University
Iridopsis larvaria	08-JDWBC-1084	LBCG1084-09	HQ648611	Canada	British Columbia	Spencer Entomological Museum, UBC
Iridopsis larvaria	ENT002-001546	GWNR104-07	HQ648604	Canada	British Columbia	Royal British Columbia Museum
Iridopsis larvaria	ENT996-003559	GWNR098-07	HQ648608	Canada	British Columbia	Royal British Columbia Museum
Iridopsis larvaria	ENT996-003568	GWNR102-07	HQ648606	Canada	British Columbia	Royal British Columbia Museum
Iridopsis larvaria	ENT996-003575	GWNR097-07	HQ648609	United States	Washington	Royal British Columbia Museum
Iridopsis larvaria	ENT996-003580	GWNR101-07	HQ648607	United States	Washington	Royal British Columbia Museum
Iridopsis larvaria	ENT996-007374	GWNR103-07	HQ648605	Canada	British Columbia	Royal British Columbia Museum
Iridopsis larvaria	NFRC-P-2007-100017	GWNN017-07	HQ648603	Canada	Alberta	Northern Forestry Centre, Canadian Forest Service
Iridopsis larvaria	NFRC-P-2007-100018	GWNN018-07	HQ648602	Canada	Alberta	Northern Forestry Centre, Canadian Forest Service
Iridopsis larvaria	PFC-2007-0376	GWNP166-07	HQ648610	Canada	British Columbia	Pacific Forestry Centre, Canadian Forest Service
Iridopsis larvaria	UASM41076	GWNS076-07	HQ648601	Canada	Alberta	Strickland Museum of Entomology, University of Alberta
Iridopsis larvaria	UASM7011	GWNS077-07	HQ648600	Canada	Alberta	Strickland Museum of Entomology, University of Alberta
Ixala desperaria	UASM57510	GWNS109-07	HQ648612	Canada	Alberta	Strickland Museum of Entomology, University of Alberta
Lambdina fiscellaria	CBCC1222	GWNS552-07	HQ648624	Canada	Alberta	Strickland Museum of Entomology, University of Alberta
Lambdina fiscellaria	CBCC1223	GWNS550-07	HQ648625	Canada	Alberta	Strickland Museum of Entomology, University of Alberta
Lambdina fiscellaria	CNCLEP00033209	GWNC210-07	HQ648615	Canada	British Columbia	Canadian National Collection of Insects, Arachnids and Nematodes
Lambdina fiscellaria	CNCLEP00033210	GWNC211-07	HQ648614	Canada	British Columbia	Canadian National Collection of Insects, Arachnids and Nematodes
Lambdina fiscellaria	Dun-08-112	DUNLP112-08	HQ648613	Canada	British Columbia	Pacific Forestry Centre, Canadian Forest Service
Lambdina fiscellaria	ENT991-010424	GWNR290-07	HQ648631	Canada	British Columbia	Royal British Columbia Museum
Lambdina fiscellaria	ENT991-011454	GWNR291-07	HQ648630	Canada	British Columbia	Royal British Columbia Museum
Lambdina fiscellaria	ENT996-004032	GWNR292-07	HQ648629	Canada	British Columbia	Royal British Columbia Museum
Lambdina fiscellaria	ENT996-007540	GWNR366-07	HQ648628	Canada	British Columbia	Royal British Columbia Museum
Lambdina fiscellaria	ENT996-007542	GWNR367-07	HQ648627	Canada	British Columbia	Royal British Columbia Museum
Lambdina fiscellaria	ENT996-007543	GWNR368-07	HQ648626	Canada	British Columbia	Royal British Columbia Museum
Lambdina fiscellaria	PFC-2007-0401	GWNP191-07	HQ648622	Canada	British Columbia	Pacific Forestry Centre, Canadian Forest Service
Lambdina fiscellaria	PFC-2007-0402	GWNP192-07	HQ648621	Canada	British Columbia	Pacific Forestry Centre, Canadian Forest Service
Lambdina fiscellaria	PFC-2007-0405	GWNP195-07	HQ648620	Canada	British Columbia	Pacific Forestry Centre, Canadian Forest Service
Lambdina fiscellaria	PFC-2007-0407	GWNP197-07	HQ648619	Canada	British Columbia	Pacific Forestry Centre, Canadian Forest Service
Lambdina fiscellaria	PFC-2007-0408	GWNP198-07	HQ648618	Canada	British Columbia	Pacific Forestry Centre, Canadian Forest Service
Lambdina fiscellaria	UASM58364	GWNS201-07	HQ648623	Canada	Alberta	Strickland Museum of Entomology, University of Alberta
Lambdina fiscellaria	WFBM-07-0060	GWNW060-07	HQ648617	United States	Idaho	University of Idaho, W. F. Barr Entomological Collection
Lambdina fiscellaria	WFBM-07-0061	GWNW061-07	HQ648616	United States	Idaho	University of Idaho, W. F. Barr Entomological Collection
Lampropteryx suffumata	08-JDWBC-0272	LBCG272-08	HQ648632	Canada	British Columbia	Spencer Entomological Museum, UBC
Lampropteryx suffumata	08-JDWBC-0279	LBCG279-08	HQ648633	Canada	British Columbia	Spencer Entomological Museum, UBC
Lampropteryx suffumata	CNCLEP00033310	GWNC311-07	FJ376639	Canada	Alberta	Canadian National Collection of Insects, Arachnids and Nematodes
Lampropteryx suffumata	ENT991-006550	GWNR470-07	FJ376637	Canada	British Columbia	Royal British Columbia Museum
Lampropteryx suffumata	HLC-20022	LBCA022-05	FJ376638	Canada	British Columbia	Biodiversity Institute of Ontario
Lampropteryx suffumata	HLC-20175	LBCA175-05	FJ376643	Canada	British Columbia	Biodiversity Institute of Ontario
Lampropteryx suffumata	HLC-20320	LBCA320-05	FJ376642	Canada	British Columbia	Biodiversity Institute of Ontario
Lampropteryx suffumata	HLC-20568	LBCA568-05	FJ376636	Canada	British Columbia	Biodiversity Institute of Ontario
Leptostales ferruminaria	CNCLEP00033331	GWNC332-07	HQ648635	Canada	Alberta	Canadian National Collection of Insects, Arachnids and Nematodes
Leptostales ferruminaria	CNCLEP00033332	GWNC333-07	HQ648634	Canada	Alberta	Canadian National Collection of Insects, Arachnids and Nematodes
Leptostales ferruminaria	UASM58026	GWNS281-07	HQ648636	Canada	Alberta	Strickland Museum of Entomology, University of Alberta
Leptostales ferruminaria	UASM58054	GWNS280-07	HQ648637	Canada	Alberta	Strickland Museum of Entomology, University of Alberta
Leptostales rubromarginaria	CNCLEP00033284	GWNC285-07	HQ648639	Canada	British Columbia	Canadian National Collection of Insects, Arachnids and Nematodes
Leptostales rubromarginaria	CNCLEP00033333	GWNC334-07	HQ648638	Canada	British Columbia	Canadian National Collection of Insects, Arachnids and Nematodes
Leptostales rubromarginaria	JECW-07-0131	GWNJ131-07	HQ648640	United States	Washington	James Entomological Collection, Washington State University
Leptostales rubromarginaria	PFC-2007-0213	GWNP006-07	HQ648641	Canada	British Columbia	Pacific Forestry Centre, Canadian Forest Service
Leptostales rubromarginaria	WFBM-07-0077	GWNW077-07	HQ648642	United States	Idaho	University of Idaho, W. F. Barr Entomological Collection
Leucobrephos brephoides	CNCLEP00033002	GWNC003-07	HQ648643	Canada	Quebec	Canadian National Collection of Insects, Arachnids and Nematodes
Lobophora canavestita	JECW-07-0178	GWNJ178-07	HQ648644	United States	California	James Entomological Collection, Washington State University
Lobophora magnoliatoidata	CNCLEP00033402	GWNC403-07	HQ648645	Canada	Alberta	Canadian National Collection of Insects, Arachnids and Nematodes

Lobophora magnoliatoidata	CNCLEP00035469	GWNC703-07	HQ648646	Canada	British Columbia	Canadian National Collection of Insects, Arachnids and Nematodes
Lobophora magnoliatoidata	ENT002-001759	GWNR617-07	HQ648652	Canada	British Columbia	Royal British Columbia Museum
Lobophora magnoliatoidata	ENT002-001760	GWNR618-07	HQ648651	Canada	British Columbia	Royal British Columbia Museum
Lobophora magnoliatoidata	PFC-2007-0232	GWNP025-07	HQ648650	Canada	British Columbia	Pacific Forestry Centre, Canadian Forest Service
Lobophora magnoliatoidata	UASM24860	GWNS485-07	HQ648649	Canada	Alberta	Strickland Museum of Entomology, University of Alberta
Lobophora magnoliatoidata	UASM57988	GWNS483-07	HQ648648	Canada	Alberta	Strickland Museum of Entomology, University of Alberta
Lobophora magnoliatoidata	UASM7028	GWNS484-07	HQ648647	Canada	Alberta	Strickland Museum of Entomology, University of Alberta
Lobophora montanata	CNCLEP00035467	GWNC701-07	HQ648653	United States	Colorado	Canadian National Collection of Insects, Arachnids and Nematodes
Lobophora nivigerata	UASM24861	GWNS487-07	HQ648654	Canada	Alberta	Strickland Museum of Entomology, University of Alberta
Lobophora simsata	ENT996-007857	GWNR653-07	HQ648656	Canada	British Columbia	Royal British Columbia Museum
Lobophora simsata	WFBM-07-0091	GWNW091-07	HQ648655	United States	Idaho	University of Idaho, W. F. Barr Entomological Collection
Lomographa semiclarata	ENT002-001559	GWNR148-07	HQ648662	Canada	British Columbia	Royal British Columbia Museum
Lomographa semiclarata	ENT002-001576	GWNR149-07	HQ648661	Canada	British Columbia	Royal British Columbia Museum
Lomographa semiclarata	ENT996-003705	GWNR147-07	HQ648663	United States	Washington	Royal British Columbia Museum
Lomographa semiclarata	ENT996-003715	GWNR146-07	HQ648664	United States	Washington	Royal British Columbia Museum
Lomographa semiclarata	JECW-07-0049	GWNJ049-07	HQ648657	United States	Idaho	James Entomological Collection, Washington State University
Lomographa semiclarata	UASM34536	GWNS102-07	HQ648658	Canada	Alberta	Strickland Museum of Entomology, University of Alberta
Lomographa semiclarata	UASM57328	GWNS100-07	HQ648660	Canada	Alberta	Strickland Museum of Entomology, University of Alberta
Lomographa semiclarata	UASM58230	GWNS101-07	HQ648659	Canada	Alberta	Strickland Museum of Entomology, University of Alberta
Lycia rachelae	07-JDWBC-0058	GWND047-07	HQ648667	Canada	Alberta	Spencer Entomological Museum, UBC
Lycia rachelae	07-JDWBC-0059	GWND048-07	HQ648666	Canada	Alberta	Spencer Entomological Museum, UBC
Lycia rachelae	07-JDWBC-0060	GWND049-07	HQ648665	Canada	Alberta	Spencer Entomological Museum, UBC
Lycia ursaria	CGWC-3952	LOWCE192-06	HQ648668	Canada	British Columbia	Biodiversity Institute of Ontario
Lycia ursaria	ENT002-001554	GWNR141-07	HQ648672	Canada	British Columbia	Royal British Columbia Museum
Lycia ursaria	ENT002-001555	GWNR142-07	HQ648671	Canada	British Columbia	Royal British Columbia Museum
Lycia ursaria	UASM24878	GWNS094-07	HQ648670	Canada	Alberta	Strickland Museum of Entomology, University of Alberta
Lycia ursaria	UASM59757	GWNS093-07	HQ648669	Canada	British Columbia	Strickland Museum of Entomology, University of Alberta
Macaria adonis	08-JDWBC-1109	LBCG1109-09	HQ648682	Canada	British Columbia	Spencer Entomological Museum, UBC
Macaria adonis	08-JDWBC-1318	LBCG1318-09	HQ648684	Canada	British Columbia	Spencer Entomological Museum, UBC
Macaria adonis	08-JDWBC-1319	LBCG1319-09	HQ648683	Canada	British Columbia	Spencer Entomological Museum, UBC
Macaria adonis	CNCLEP00033058	GWNC059-07	HQ648675	Canada	British Columbia	Canadian National Collection of Insects, Arachnids and Nematodes
Macaria adonis	CNCLEP00033343	GWNC344-07	HQ648674	Canada	British Columbia	Canadian National Collection of Insects, Arachnids and Nematodes
Macaria adonis	Dun-08-115	DUNLP115-08	HQ648673	Canada	British Columbia	Pacific Forestry Centre, Canadian Forest Service
Macaria adonis	ENT996-003267	GWNR035-07	HQ648680	Canada	British Columbia	Royal British Columbia Museum
Macaria adonis	JECW-07-0011	GWNJ011-07	HQ648676	United States	Washington	James Entomological Collection, Washington State University
Macaria adonis	JECW-07-0012	GWNJ012-07	HQ648677	United States	Idaho	James Entomological Collection, Washington State University
Macaria adonis	PFC-2007-0344	GWNP134-07	HQ648679	Canada	British Columbia	Pacific Forestry Centre, Canadian Forest Service
Macaria adonis	PFC-2007-0345	GWNP135-07	HQ648678	Canada	British Columbia	Pacific Forestry Centre, Canadian Forest Service
Macaria adonis	WFBM-07-0006	GWNW006-07	HQ648681	United States	Idaho	University of Idaho, W. F. Barr Entomological Collection
Macaria aemulataria	UASM58112	GWNS027-07	HQ648685	Canada	Alberta	Strickland Museum of Entomology, University of Alberta
Macaria amboflava	CBCC804	GWNS013-07	HQ648688	Canada	Alberta	Strickland Museum of Entomology, University of Alberta
Macaria amboflava	CNCLEP00033019	GWNC020-07	HQ648687	Canada	British Columbia	Canadian National Collection of Insects, Arachnids and Nematodes
Macaria amboflava	CNCLEP00033020	GWNC021-07	HQ648686	Canada	British Columbia	Canadian National Collection of Insects, Arachnids and Nematodes
Macaria amboflava	ENT002-001532	GWNR030-07	HQ648693	Canada	British Columbia	Royal British Columbia Museum
Macaria amboflava	ENT002-001533	GWNR032-07	HQ648691	Canada	British Columbia	Royal British Columbia Museum
Macaria amboflava	ENT002-001534	GWNR031-07	HQ648692	Canada	British Columbia	Royal British Columbia Museum
Macaria amboflava	UASM57938	GWNS012-07	HQ648689	Canada	Alberta	Strickland Museum of Entomology, University of Alberta
Macaria amboflava	UASM58577	GWNS011-07	HQ648690	Canada	Alberta	Strickland Museum of Entomology, University of Alberta
Macaria atrimacularia	09-JDWGEO-482	GNAU182-10	HM392451	United States	Texas	Smithsonian Institution
Macaria bitactata	08-JDWBC-0208	LBCG208-08	HQ648701	Canada	British Columbia	Spencer Entomological Museum, UBC
Macaria bitactata	08-JDWBC-1006	LBCG1006-09	HQ648702	Canada	British Columbia	Spencer Entomological Museum, UBC
Macaria bitactata	CNCLEP00033037	GWNC038-07	HQ648694	Canada	British Columbia	Canadian National Collection of Insects, Arachnids and Nematodes
Macaria bitactata	CNCLEP00033339	GWNC340-07	HQ648696	Canada	Alberta	Canadian National Collection of Insects, Arachnids and Nematodes
Macaria bitactata	CNCLEP00033340	GWNC341-07	HQ648695	Canada	Alberta	Canadian National Collection of Insects, Arachnids and Nematodes
Macaria bitactata	ENT996-003186	GWNR013-07	HQ648700	Canada	British Columbia	Royal British Columbia Museum
Macaria bitactata	NFRC-P-2007-100005	GWNN005-07	HQ648699	Canada	Alberta	Northern Forestry Centre, Canadian Forest Service
Macaria bitactata	NFRC-P-2007-100006	GWNN006-07	HQ648698	Canada	Alberta	Northern Forestry Centre, Canadian Forest Service
Macaria bitactata	NFRC-P-2007-100007	GWNN007-07	HQ648697	Canada	Alberta	Northern Forestry Centre, Canadian Forest Service
Macaria brunneata	CNCLEP00033026	GWNC027-07	HQ648703	Canada	British Columbia	Canadian National Collection of Insects, Arachnids and Nematodes
Macaria brunneata	CNCLEP00033027	GWNC028-07	HQ648704	Canada	Yukon Territory	Canadian National Collection of Insects, Arachnids and Nematodes
Macaria brunneata	ENT002-001535	GWNR016-07	HQ648708	Canada	British Columbia	Royal British Columbia Museum
Macaria brunneata	ENT996-003177	GWNR015-07	HQ648709	United States	Washington	Royal British Columbia Museum
Macaria brunneata	ENT996-003178	GWNR014-07	HQ648710	United States	Washington	Royal British Columbia Museum
Macaria brunneata	JD0841	GWNS554-07	HQ648711	Canada	Alberta	Strickland Museum of Entomology, University of Alberta
Macaria brunneata	UASM34898	GWNS016-07	HQ648705	Canada	Alberta	Strickland Museum of Entomology, University of Alberta
Macaria brunneata	UASM58780	GWNS014-07	HQ648707	Canada	Alberta	Strickland Museum of Entomology, University of Alberta
Macaria brunneata	UASM78365	GWNS015-07	HQ648706	Canada	Alberta	Strickland Museum of Entomology, University of Alberta
Macaria colata	CNCLEP00033040	GWNC041-07	HQ648712	Canada	British Columbia	Canadian National Collection of Insects, Arachnids and Nematodes
Macaria colata	ENT996-003189	GWNR018-07	HQ648718	Canada	British Columbia	Royal British Columbia Museum
Macaria colata	ENT996-003192	GWNR017-07	HQ648719	United States	Washington	Royal British Columbia Museum
Macaria colata	ENT996-003195	GWNR019-07	HQ648717	United States	Washington	Royal British Columbia Museum
Macaria colata	JECW-07-0001	GWNJ001-07	HQ648715	United States	Washington	James Entomological Collection, Washington State University
Macaria colata	JECW-07-0003	GWNJ003-07	HQ648714	United States	Washington	James Entomological Collection, Washington State University
Macaria colata	JECW-07-0004	GWNJ004-07	HQ648713	United States	Washington	James Entomological Collection, Washington State University
Macaria colata	PFC-2007-0359	GWNP149-07	HQ648716	Canada	British Columbia	Pacific Forestry Centre, Canadian Forest Service
Macaria colata	WFBM-07-0001	GWNW001-07	HQ648720	United States	Idaho	University of Idaho, W. F. Barr Entomological Collection
Macaria decorata	08-JDWBC-0200	LBCG200-08	HQ648728	Canada	British Columbia	Spencer Entomological Museum, UBC
Macaria decorata	08-JDWBC-0207	LBCG207-08	HQ648727	Canada	British Columbia	Spencer Entomological Museum, UBC
Macaria decorata	08-JDWBC-1335	LBCG1335-09	HQ648730	Canada	British Columbia	Spencer Entomological Museum, UBC
Macaria decorata	08-JDWBC-1338	LBCG1338-09	HQ648729	Canada	British Columbia	Spencer Entomological Museum, UBC
Macaria decorata	CNCLEP00033039	GWNC040-07	HQ648721	Canada	British Columbia	Canadian National Collection of Insects, Arachnids and Nematodes
Macaria decorata	CNCLEP00033325	GWNC326-07	HQ648723	Canada	Alberta	Canadian National Collection of Insects, Arachnids and Nematodes
Macaria decorata	CNCLEP00033327	GWNC328-07	HQ648722	Canada	Alberta	Canadian National Collection of Insects, Arachnids and Nematodes
Macaria decorata	ENT991-010309	GWNR040-07	HQ648726	Canada	British Columbia	Royal British Columbia Museum
Macaria decorata	UASM41387	GWNS023-07	HQ648724	Canada	Alberta	Strickland Museum of Entomology, University of Alberta
Macaria decorata	UASM59968	GWNS505-07	HQ648725	Canada	Alberta	Strickland Museum of Entomology, University of Alberta
Macaria exauspicata	08-JDWBC-2328	LBCG2328-09	HQ648734	Canada	British Columbia	Spencer Entomological Museum, UBC
Macaria exauspicata	08-JDWBC-2332	LBCG2332-09	HQ648735	Canada	British Columbia	Spencer Entomological Museum, UBC
Macaria exauspicata	Dun-08-111	DUNLP111-08	HQ648731	Canada	British Columbia	Pacific Forestry Centre, Canadian Forest Service
Macaria exauspicata	ENT002-001539	GWNR022-07	HQ648733	Canada	British Columbia	Royal British Columbia Museum
Macaria exauspicata	UASM41341	GWNS021-07	HQ648732	Canada	Alberta	Strickland Museum of Entomology, University of Alberta
Macaria loricaria	CNCLEP00033033	GWNC034-07	HQ648736	Canada	Yukon Territory	Canadian National Collection of Insects, Arachnids and Nematodes
Macaria loricaria	ENT002-001537	GWNR024-07	HQ648742	Canada	British Columbia	Royal British Columbia Museum
Macaria loricaria	ENT002-001538	GWNR023-07	HQ648743	Canada	British Columbia	Royal British Columbia Museum
Macaria loricaria	ENT992-021184	GWNR025-07	HQ648741	Canada	British Columbia	Royal British Columbia Museum
Macaria loricaria	ENT996-003181	GWNR020-07	HQ648744	Canada	British Columbia	Royal British Columbia Museum
Macaria loricaria	PFC-2007-0362	GWNP152-07	HQ648740	Canada	British Columbia	Pacific Forestry Centre, Canadian Forest Service
Macaria loricaria	UASM35893	GWNS020-07	HQ648737	Canada	British Columbia	Strickland Museum of Entomology, University of Alberta
Macaria loricaria	UASM59140	GWNS586-07	HQ648745	Canada	Alberta	Strickland Museum of Entomology, University of Alberta
Macaria loricaria	UASM7008	GWNS018-07	HQ648739	Canada	Alberta	Strickland Museum of Entomology, University of Alberta
Macaria loricaria	UASM95793	GWNS019-07	HQ648738	Canada	Alberta	Strickland Museum of Entomology, University of Alberta
Macaria lorquinaria	CNCLEP00033053	GWNC054-07	HQ648747	Canada	British Columbia	Canadian National Collection of Insects, Arachnids and Nematodes
Macaria lorquinaria	CNCLEP00033054	GWNC055-07	HQ648746	Canada	British Columbia	Canadian National Collection of Insects, Arachnids and Nematodes
Macaria lorquinaria	ENT996-003197	GWNR074-07	HQ648751	Canada	British Columbia	Royal British Columbia Museum
Macaria lorquinaria	ENT996-003264	GWNR073-07	HQ648752	Canada	British Columbia	Royal British Columbia Museum
Macaria lorquinaria	JECW-07-0009	GWNJ009-07	HQ648748	United States	Washington	James Entomological Collection, Washington State University
Macaria lorquinaria	JECW-07-0010	GWNJ010-07	HQ648749	Canada	British Columbia	James Entomological Collection, Washington State University

Macaria lorquinaria	PFC-2007-0363	GWNP153-07	HQ648750	Canada	British Columbia	Pacific Forestry Centre, Canadian Forest Service
Macaria lorquinaria	WFBM-07-0002	GWNW002-07	HQ648753	United States	Idaho	University of Idaho, W. F. Barr Entomological Collection
Macaria masquerata	10-GOBCL-02	GOBCL002-10	HQ648754	United States	California	Barcode of Life Data System
Macaria occiduaria	CNCLEP00033012	GWNC013-07	HQ648755	Canada	British Columbia	Canadian National Collection of Insects, Arachnids and Nematodes
Macaria occiduaria	CNCLEP00033014	GWNC015-07	HQ648757	Canada	Yukon Territory	Canadian National Collection of Insects, Arachnids and Nematodes
Macaria occiduaria	CNCLEP00033029	GWNC030-07	HQ648756	Canada	Yukon Territory	Canadian National Collection of Insects, Arachnids and Nematodes
Macaria occiduaria	ENT002-001530	GWNR029-07	HQ648761	Canada	British Columbia	Royal British Columbia Museum
Macaria occiduaria	ENT002-001531	GWNR028-07	HQ648762	Canada	British Columbia	Royal British Columbia Museum
Macaria occiduaria	ENT992-020794	GWNR010-07	HQ648766	Canada	British Columbia	Royal British Columbia Museum
Macaria occiduaria	ENT992-020795	GWNR011-07	HQ648765	Canada	British Columbia	Royal British Columbia Museum
Macaria occiduaria	ENT996-003175	GWNR027-07	HQ648763	Canada	British Columbia	Royal British Columbia Museum
Macaria occiduaria	ENT996-003176	GWNR026-07	HQ648764	Canada	Alberta	Royal British Columbia Museum
Macaria occiduaria	JECW-07-0006	GWNJ006-07	HQ648758	Canada	British Columbia	James Entomological Collection, Washington State University
Macaria occiduaria	UASM58452	GWNS010-07	HQ648759	Canada	Alberta	Strickland Museum of Entomology, University of Alberta
Macaria occiduaria	UASM77880	GWNS009-07	HQ648760	Canada	Alberta	Strickland Museum of Entomology, University of Alberta
Macaria occiduaria	UASM78401	GWNS563-07	HQ648767	Canada	Alberta	Strickland Museum of Entomology, University of Alberta
Macaria oweni	CNCLEP00033344	GWNC345-07	HQ648769	Canada	Alberta	Canadian National Collection of Insects, Arachnids and Nematodes
Macaria oweni	CNCLEP00033345	GWNC346-07	HQ648768	Canada	Alberta	Canadian National Collection of Insects, Arachnids and Nematodes
Macaria oweni	UASM29098	GWNS590-07	HQ648770	Canada	Alberta	Strickland Museum of Entomology, University of Alberta
Macaria perplexata	WFBM-07-0012	GWNW012-07	HQ648772	United States	Idaho	University of Idaho, W. F. Barr Entomological Collection
Macaria perplexata	WFBM-07-0013	GWNW013-07	HQ648771	United States	Idaho	University of Idaho, W. F. Barr Entomological Collection
Macaria plumosata	08-JDWBC-2117	LBCG2117-09	HQ648777	Canada	British Columbia	Spencer Entomological Museum, UBC
Macaria plumosata	JECW-07-0025	GWNJ025-07	HQ648773	United States	Washington	James Entomological Collection, Washington State University
Macaria plumosata	JECW-07-0026	GWNJ026-07	HQ648775	United States	Washington	James Entomological Collection, Washington State University
Macaria plumosata	UASM58550	GWNS022-07	HQ648774	Canada	Alberta	Strickland Museum of Entomology, University of Alberta
Macaria plumosata	WFBM-07-0004	GWNW004-07	HQ648776	United States	Idaho	University of Idaho, W. F. Barr Entomological Collection
Macaria quadrilinearia	08-JDWBC-3278	LBCG3278-09	HQ648782	Canada	British Columbia	Spencer Entomological Museum, UBC
Macaria quadrilinearia	JECW-07-0007	GWNJ007-07	HQ648779	United States	Washington	James Entomological Collection, Washington State University
Macaria quadrilinearia	JECW-07-0008	GWNJ008-07	HQ648778	United States	Washington	James Entomological Collection, Washington State University
Macaria quadrilinearia	UASM10896	GWNS605-08	HQ648780	Canada	Alberta	Strickland Museum of Entomology, University of Alberta
Macaria quadrilinearia	WFBM-07-0003	GWNW003-07	HQ648781	United States	Idaho	University of Idaho, W. F. Barr Entomological Collection
Macaria ribearia	CNCLEP00033307	GWNC308-07	HQ648783	Canada	Alberta	Canadian National Collection of Insects, Arachnids and Nematodes
Macaria sexmaculata	CNCLEP00033342	GWNC343-07	HQ648785	Canada	British Columbia	Canadian National Collection of Insects, Arachnids and Nematodes
Macaria sexmaculata	Dun-08-118	DUNLP118-08	HQ648784	Canada	British Columbia	Pacific Forestry Centre, Canadian Forest Service
Macaria sexmaculata	ENT996-003275	GWNR066-07	HQ648791	Canada	British Columbia	Royal British Columbia Museum
Macaria sexmaculata	ENT996-003387	GWNR050-07	HQ648792	Canada	British Columbia	Royal British Columbia Museum
Macaria sexmaculata	PFC-2007-0348	GWNP138-07	HQ648790	Canada	British Columbia	Pacific Forestry Centre, Canadian Forest Service
Macaria sexmaculata	PFC-2007-0349	GWNP139-07	HQ648789	Canada	British Columbia	Pacific Forestry Centre, Canadian Forest Service
Macaria sexmaculata	UASM29082	GWNS592-07	HQ648793	Canada	Alberta	Strickland Museum of Entomology, University of Alberta
Macaria sexmaculata	UASM29086	GWNS589-07	HQ648794	Canada	Alberta	Strickland Museum of Entomology, University of Alberta
Macaria sexmaculata	UASM41343	GWNS036-07	HQ648787	Canada	Alberta	Strickland Museum of Entomology, University of Alberta
Macaria sexmaculata	UASM53017	GWNS039-07	HQ648786	Canada	Alberta	Strickland Museum of Entomology, University of Alberta
Macaria sexmaculata	UASM95794	GWNS031-07	HQ648788	Canada	Alberta	Strickland Museum of Entomology, University of Alberta
Macaria signaria	08-JDWBC-0031	LBCG031-08	HQ648827	Canada	British Columbia	Spencer Entomological Museum, UBC
Macaria signaria	08-JDWBC-0111	LBCG111-08	HQ648826	Canada	British Columbia	Spencer Entomological Museum, UBC
Macaria signaria	08-JDWBC-0113	LBCG113-08	HQ648825	Canada	British Columbia	Spencer Entomological Museum, UBC
Macaria signaria	08-JDWBC-0428	LBCG428-08	HQ648828	Canada	British Columbia	Spencer Entomological Museum, UBC
Macaria signaria	CNCLEP00033060	GWNC061-07	HQ648802	Canada	British Columbia	Canadian National Collection of Insects, Arachnids and Nematodes
Macaria signaria	CNCLEP00033061	GWNC062-07	HQ648803	Canada	Yukon Territory	Canadian National Collection of Insects, Arachnids and Nematodes
Macaria signaria	CNCLEP00033062	GWNC063-07	HQ648801	Canada	British Columbia	Canadian National Collection of Insects, Arachnids and Nematodes
Macaria signaria	CNCLEP00033064	GWNC065-07	HQ648805	Canada	Alberta	Canadian National Collection of Insects, Arachnids and Nematodes
Macaria signaria	CNCLEP00033328	GWNC329-07	HQ648804	Canada	Alberta	Canadian National Collection of Insects, Arachnids and Nematodes
Macaria signaria	CNCLEP00033329	GWNC330-07	HQ648800	Canada	British Columbia	Canadian National Collection of Insects, Arachnids and Nematodes
Macaria signaria	CNCLEP00033330	GWNC331-07	HQ648799	Canada	British Columbia	Canadian National Collection of Insects, Arachnids and Nematodes
Macaria signaria	Dun-08-116	DUNLP116-08	HQ648795	Canada	British Columbia	Pacific Forestry Centre, Canadian Forest Service
Macaria signaria	Dun-08-117	DUNLP117-08	HQ648798	Canada	British Columbia	Pacific Forestry Centre, Canadian Forest Service
Macaria signaria	Dun-08-119	DUNLP119-08	HQ648797	Canada	British Columbia	Pacific Forestry Centre, Canadian Forest Service
Macaria signaria	Dun-08-120	DUNLP120-08	HQ648796	Canada	British Columbia	Pacific Forestry Centre, Canadian Forest Service
Macaria signaria	ENT991-005635	GWNR039-07	HQ648818	Canada	British Columbia	Royal British Columbia Museum
Macaria signaria	ENT991-006782	GWNR065-07	HQ648816	Canada	British Columbia	Royal British Columbia Museum
Macaria signaria	ENT996-003311	GWNR070-07	HQ648815	Canada	British Columbia	Royal British Columbia Museum
Macaria signaria	ENT996-003345	GWNR038-07	HQ648819	Canada	British Columbia	Royal British Columbia Museum
Macaria signaria	ENT996-003391	GWNR061-07	HQ648817	Canada	British Columbia	Royal British Columbia Museum
Macaria signaria	JECW-07-0029	GWNJ029-07	HQ648806	United States	Washington	James Entomological Collection, Washington State University
Macaria signaria	NFRC-P-2007-100008	GWNN008-07	HQ648814	Canada	Alberta	Northern Forestry Centre, Canadian Forest Service
Macaria signaria	PFC-2007-0346	GWNP136-07	HQ648813	Canada	British Columbia	Pacific Forestry Centre, Canadian Forest Service
Macaria signaria	UASM29103	GWNS588-07	HQ648821	Canada	Alberta	Strickland Museum of Entomology, University of Alberta
Macaria signaria	UASM29104	GWNS593-07	HQ648820	Canada	Alberta	Strickland Museum of Entomology, University of Alberta
Macaria signaria	UASM57987	GWNS033-07	HQ648811	Canada	Alberta	Strickland Museum of Entomology, University of Alberta
Macaria signaria	UASM58013	GWNS032-07	HQ648812	Canada	Alberta	Strickland Museum of Entomology, University of Alberta
Macaria signaria	UASM78410	GWNS561-07	HQ648823	Canada	Alberta	Strickland Museum of Entomology, University of Alberta
Macaria signaria	UASM78411	GWNS560-07	HQ648824	Canada	Alberta	Strickland Museum of Entomology, University of Alberta
Macaria signaria	UASM78412	GWNS562-07	HQ648822	Canada	Alberta	Strickland Museum of Entomology, University of Alberta
Macaria signaria	UASM95795	GWNS034-07	HQ648810	Canada	Alberta	Strickland Museum of Entomology, University of Alberta
Macaria signaria	UASM95796	GWNS035-07	HQ648809	Canada	Alberta	Strickland Museum of Entomology, University of Alberta
Macaria signaria	UASM95797	GWNS037-07	HQ648808	Canada	Alberta	Strickland Museum of Entomology, University of Alberta
Macaria signaria	UASM99594	GWNS608-08	HQ648807	Canada	Alberta	Strickland Museum of Entomology, University of Alberta
Macaria simplex	CNCLEP00033337	GWNC338-07	HQ648830	Canada	Alberta	Canadian National Collection of Insects, Arachnids and Nematodes
Macaria simplex	CNCLEP00033338	GWNC339-07	HQ648829	Canada	Alberta	Canadian National Collection of Insects, Arachnids and Nematodes
Macaria simplex	JD1136	GWNS506-07	HQ648833	Canada	Alberta	Strickland Museum of Entomology, University of Alberta
Macaria simplex	NFRC-P-2007-100098	GWNN114-07	HQ648832	Canada	Alberta	Northern Forestry Centre, Canadian Forest Service
Macaria simplex	NFRC-P-2007-100099	GWNN115-07	HQ648831	Canada	Alberta	Northern Forestry Centre, Canadian Forest Service
Macaria simplex	WFBM-07-0005	GWNW005-07	HQ648834	United States	Idaho	University of Idaho, W. F. Barr Entomological Collection
Macaria sp.	JECW-07-0005	GWNJ005-07	HQ648835	United States	Washington	James Entomological Collection, Washington State University
Macaria sp. 1	PFC-2007-0355	GWNP145-07	HQ648836	Canada	British Columbia	Pacific Forestry Centre, Canadian Forest Service
Macaria sp. 12	ENT002-001536	GWNR009-07	HQ648837	Canada	British Columbia	Royal British Columbia Museum
Macaria submarmorata	HLC-20854	LBCA854-05	HQ648841	Canada	British Columbia	Biodiversity Institute of Ontario
Macaria submarmorata	HLC-20879	LBCA879-05	HQ648840	Canada	British Columbia	Biodiversity Institute of Ontario
Macaria submarmorata	HLC-21174	LBCB234-05	HQ648839	Canada	British Columbia	Biodiversity Institute of Ontario
Macaria submarmorata	HLC-21592	LBCB652-05	HQ648838	Canada	British Columbia	Biodiversity Institute of Ontario
Macaria submarmorata	UASM29097	GWNS591-07	HQ648842	Canada	Alberta	Strickland Museum of Entomology, University of Alberta
Macaria truncataria	CGWC-4101	LOWCE341-06	HQ648843	Canada	British Columbia	Biodiversity Institute of Ontario
Macaria truncataria	CNCLEP00033051	GWNC052-07	HQ648844	Canada	Yukon Territory	Canadian National Collection of Insects, Arachnids and Nematodes
Macaria truncataria	UASM41704	GWNS025-07	HQ648846	Canada	Alberta	Strickland Museum of Entomology, University of Alberta
Macaria truncataria	UASM41832	GWNS024-07	HQ648847	Canada	Alberta	Strickland Museum of Entomology, University of Alberta
Macaria truncataria	UASM53051	GWNS026-07	HQ648845	Canada	Alberta	Strickland Museum of Entomology, University of Alberta
Macaria truncataria	UASM95870	GWNS541-07	HQ648848	Canada	Alberta	Strickland Museum of Entomology, University of Alberta
Macaria ulsterata	CBCC806	GWNS030-07	HQ648850	Canada	Alberta	Strickland Museum of Entomology, University of Alberta
Macaria ulsterata	CNCLEP00033341	GWNC342-07	HQ648849	Canada	Alberta	Canadian National Collection of Insects, Arachnids and Nematodes
Macaria ulsterata	ENT002-001540	GWNR072-07	HQ648852	Canada	British Columbia	Royal British Columbia Museum
Macaria ulsterata	UASM53011	GWNS028-07	HQ648851	Canada	Alberta	Strickland Museum of Entomology, University of Alberta
Macaria ulsterata	UASM78406	GWNS564-07	HQ648854	Canada	Alberta	Strickland Museum of Entomology, University of Alberta
Macaria ulsterata	UASM78408	GWNS566-07	HQ648853	Canada	Alberta	Strickland Museum of Entomology, University of Alberta
Melanolophia imitata	07-JDWBC-0005	GWND005-07	HQ648871	Canada	British Columbia	Royal British Columbia Museum
Melanolophia imitata	07-JDWBC-0006	GWND006-07	HQ648870	Canada	British Columbia	Royal British Columbia Museum
Melanolophia imitata	07-JDWBC-0009	GWND009-07	HQ648869	Canada	British Columbia	Royal British Columbia Museum
Melanolophia imitata	07-JDWBC-0010	GWND010-07	HQ648868	Canada	British Columbia	Royal British Columbia Museum

Melanolophia imitata	07-JDWBC-0020	GWND013-07	HQ648867	Canada	British Columbia	Royal British Columbia Museum
Melanolophia imitata	07-JDWBC-0026	GWND019-07	HQ648866	Canada	British Columbia	Royal British Columbia Museum
Melanolophia imitata	07-JDWBC-0027	GWND020-07	HQ648865	Canada	British Columbia	Royal British Columbia Museum
Melanolophia imitata	07-JDWBC-0028	GWND021-07	HQ648864	Canada	British Columbia	Royal British Columbia Museum
Melanolophia imitata	07-JDWBC-0030	GWND022-07	HQ648863	Canada	British Columbia	Royal British Columbia Museum
Melanolophia imitata	07-JDWBC-0037	GWND029-07	HQ648862	Canada	British Columbia	Royal British Columbia Museum
Melanolophia imitata	07-JDWBC-0038	GWND030-07	HQ648861	Canada	British Columbia	Royal British Columbia Museum
Melanolophia imitata	07-JDWBC-0041	GWND033-07	HQ648860	Canada	British Columbia	Royal British Columbia Museum
Melanolophia imitata	07-JDWBC-0046	GWND038-07	HQ648859	Canada	British Columbia	Royal British Columbia Museum
Melanolophia imitata	08-JDWBC-0286	LBCG286-08	HQ648876	Canada	British Columbia	Spencer Entomological Museum, UBC
Melanolophia imitata	08-JDWBC-0299	LBCG299-08	HQ648875	Canada	British Columbia	Spencer Entomological Museum, UBC
Melanolophia imitata	CNCLEP00033108	GWNC109-07	HQ648857	Canada	British Columbia	Canadian National Collection of Insects, Arachnids and Nematodes
Melanolophia imitata	CNCLEP00033109	GWNC110-07	HQ648856	Canada	British Columbia	Canadian National Collection of Insects, Arachnids and Nematodes
Melanolophia imitata	CNCLEP00033110	GWNC111-07	HQ648858	United States	Washington	Canadian National Collection of Insects, Arachnids and Nematodes
Melanolophia imitata	Dun-08-121	DUNLP121-08	HQ648855	Canada	British Columbia	Pacific Forestry Centre, Canadian Forest Service
Melanolophia imitata	ENT996-003599	GWNR121-07	HQ648874	United States	Washington	Royal British Columbia Museum
Melanolophia imitata	ENT996-003608	GWNR122-07	HQ648873	Canada	British Columbia	Royal British Columbia Museum
Melanolophia imitata	UASM59773	GWNS085-07	HQ648872	Canada	British Columbia	Strickland Museum of Entomology, University of Alberta
Meris suffusaria	ENT996-007535	GWNR369-07	HQ648878	Canada	British Columbia	Royal British Columbia Museum
Meris suffusaria	ENT996-007537	GWNR370-07	HQ648877	Canada	British Columbia	Royal British Columbia Museum
Mesoleuca gratulata	07-JDWBC-0031	GWND023-07	HQ648881	Canada	British Columbia	Royal British Columbia Museum
Mesoleuca gratulata	07-JDWBC-0032	GWND024-07	HQ648880	Canada	British Columbia	Royal British Columbia Museum
Mesoleuca gratulata	07-JDWBC-0033	GWND025-07	HQ648879	Canada	British Columbia	Royal British Columbia Museum
Mesoleuca gratulata	ENT996-004381	GWNR483-07	HQ648886	Canada	British Columbia	Royal British Columbia Museum
Mesoleuca gratulata	ENT996-004395	GWNR482-07	HQ648887	Canada	British Columbia	Royal British Columbia Museum
Mesoleuca gratulata	NFRC-P-2007-100066	GWNN066-07	HQ648885	Canada	Alberta	Northern Forestry Centre, Canadian Forest Service
Mesoleuca gratulata	PFC-2007-0328	GWNP118-07	HQ648884	Canada	British Columbia	Pacific Forestry Centre, Canadian Forest Service
Mesoleuca gratulata	PFC-2007-0329	GWNP119-07	HQ648883	Canada	British Columbia	Pacific Forestry Centre, Canadian Forest Service
Mesoleuca gratulata	UASM59733	GWNS339-07	HQ648882	Canada	British Columbia	Strickland Museum of Entomology, University of Alberta
Mesoleuca ruficillata	CBCC1027	GWNS337-07	HQ648890	Canada	Alberta	Strickland Museum of Entomology, University of Alberta
Mesoleuca ruficillata	ENT996-007757	GWNR632-07	HQ648895	Canada	British Columbia	Royal British Columbia Museum
Mesoleuca ruficillata	ENT996-007758	GWNR633-07	HQ648894	Canada	British Columbia	Royal British Columbia Museum
Mesoleuca ruficillata	ENT996-007759	GWNR634-07	HQ648893	Canada	British Columbia	Royal British Columbia Museum
Mesoleuca ruficillata	JECW-07-0149	GWNJ149-07	HQ648888	United States	Idaho	James Entomological Collection, Washington State University
Mesoleuca ruficillata	NFRC-P-2007-100065	GWNN065-07	HQ648892	Canada	Alberta	Northern Forestry Centre, Canadian Forest Service
Mesoleuca ruficillata	PFC-2007-0327	GWNP117-07	HQ648891	Canada	British Columbia	Pacific Forestry Centre, Canadian Forest Service
Mesoleuca ruficillata	UASM41839	GWNS338-07	HQ648889	Canada	Alberta	Strickland Museum of Entomology, University of Alberta
Mesothea incertata	ENT002-001647	GWNR352-07	HQ648898	Canada	British Columbia	Royal British Columbia Museum
Mesothea incertata	ENT991-131363	GWNR353-07	HQ648897	Canada	British Columbia	Royal British Columbia Museum
Mesothea incertata	ENT996-007624	GWNR351-07	HQ648899	Canada	British Columbia	Royal British Columbia Museum
Mesothea incertata	JECW-07-0127	GWNJ127-07	HQ648896	United States	Washington	James Entomological Collection, Washington State University
Mesothea incertata	UASM41831	GWNS245-07	HQ648900	Canada	Alberta	Strickland Museum of Entomology, University of Alberta
Mesothea incertata	UASM43220	GWNS244-07	HQ648901	Canada	Alberta	Strickland Museum of Entomology, University of Alberta
Mesothea incertata	UASM53025	GWNS536-07	HQ648903	Canada	Alberta	Strickland Museum of Entomology, University of Alberta
Mesothea incertata	UASM53026	GWNS535-07	HQ648904	Canada	Alberta	Strickland Museum of Entomology, University of Alberta
Mesothea incertata	UASM53028	GWNS534-07	HQ648905	Canada	Alberta	Strickland Museum of Entomology, University of Alberta
Mesothea incertata	UASM58231	GWNS243-07	HQ648902	Canada	Alberta	Strickland Museum of Entomology, University of Alberta
Metanema determinata	CNCLEP00033189	GWNC190-07	HQ648907	Canada	British Columbia	Canadian National Collection of Insects, Arachnids and Nematodes
Metanema determinata	CNCLEP00033191	GWNC192-07	HQ648906	Canada	British Columbia	Canadian National Collection of Insects, Arachnids and Nematodes
Metanema determinata	UASM24858	GWNS171-07	HQ648909	Canada	Alberta	Strickland Museum of Entomology, University of Alberta
Metanema determinata	UASM7068	GWNS170-07	HQ648910	Canada	Alberta	Strickland Museum of Entomology, University of Alberta
Metanema determinata	UASM7356	GWNS172-07	HQ648908	Canada	Alberta	Strickland Museum of Entomology, University of Alberta
Metanema inatomaria	CGWC-0246	LOWC246-05	HQ648911	Canada	British Columbia	Biodiversity Institute of Ontario
Metanema inatomaria	CGWC-0247	LOWC247-05	HQ648913	Canada	British Columbia	Biodiversity Institute of Ontario
Metanema inatomaria	CGWC-0249	LOWC249-05	HQ648912	Canada	British Columbia	Biodiversity Institute of Ontario
Metanema inatomaria	UASM43323	GWNS169-07	HQ648914	Canada	Alberta	Strickland Museum of Entomology, University of Alberta
Metanema inatomaria	UASM44324	GWNS167-07	HQ648915	Canada	Alberta	Strickland Museum of Entomology, University of Alberta
Metarranthis duaria	08-JDWBC-0015	LBCG015-08	HQ648921	Canada	British Columbia	Spencer Entomological Museum, UBC
Metarranthis duaria	CNCLEP00033193	GWNC194-07	HQ648916	Canada	British Columbia	Canadian National Collection of Insects, Arachnids and Nematodes
Metarranthis duaria	ENT002-001616	GWNR257-07	HQ648923	Canada	British Columbia	Royal British Columbia Museum
Metarranthis duaria	ENT002-001620	GWNR258-07	HQ648922	Canada	British Columbia	Royal British Columbia Museum
Metarranthis duaria	UASM57075	GWNS175-07	HQ648917	Canada	Alberta	Strickland Museum of Entomology, University of Alberta
Metarranthis duaria	UASM57418	GWNS173-07	HQ648919	Canada	Alberta	Strickland Museum of Entomology, University of Alberta
Metarranthis duaria	UASM77970	GWNS174-07	HQ648918	Canada	Alberta	Strickland Museum of Entomology, University of Alberta
Metarranthis duaria	WFBM-07-0054	GWNW054-07	HQ648920	United States	Idaho	University of Idaho, W. F. Barr Entomological Collection
Minoa murinata	10-GOBCL-06	GOBCL006-10	HQ648924	Germany	Bavaria	Barcode of Life Data System
Narraga fimetaria	UASM24786	GWNS062-07	HQ648926	Canada	Alberta	Strickland Museum of Entomology, University of Alberta
Narraga fimetaria	UASM41590	GWNS063-07	HQ648925	Canada	Alberta	Strickland Museum of Entomology, University of Alberta
Narraga fimetaria	WFBM-07-0018	GWNW018-07	HQ648927	United States	Idaho	University of Idaho, W. F. Barr Entomological Collection
Nematocampa resistaria	08-JDWBC-3728	LBCG3728-09	HQ648944	Canada	British Columbia	Spencer Entomological Museum, UBC
Nematocampa resistaria	08-JDWBC-3729	LBCG3729-09	HQ648943	Canada	British Columbia	Spencer Entomological Museum, UBC
Nematocampa resistaria	08-JDWBC-3730	LBCG3730-09	HQ648942	Canada	British Columbia	Spencer Entomological Museum, UBC
Nematocampa resistaria	08-JDWBC-3731	LBCG3731-09	HQ648941	Canada	British Columbia	Spencer Entomological Museum, UBC
Nematocampa resistaria	08-JDWBC-3732	LBCG3732-09	HQ648940	Canada	British Columbia	Spencer Entomological Museum, UBC
Nematocampa resistaria	08-JDWBC-3734	LBCG3734-09	HQ648939	Canada	British Columbia	Spencer Entomological Museum, UBC
Nematocampa resistaria	08-JDWBC-3735	LBCG3735-09	HQ648938	Canada	British Columbia	Spencer Entomological Museum, UBC
Nematocampa resistaria	CNCLEP00033236	GWNC237-07	HQ648929	Canada	British Columbia	Canadian National Collection of Insects, Arachnids and Nematodes
Nematocampa resistaria	Dun-08-123	DUNLP123-08	HQ648928	Canada	British Columbia	Pacific Forestry Centre, Canadian Forest Service
Nematocampa resistaria	ENT002-001642	GWNR295-07	HQ648935	Canada	British Columbia	Royal British Columbia Museum
Nematocampa resistaria	ENT991-008446	GWNR293-07	HQ648937	Canada	British Columbia	Royal British Columbia Museum
Nematocampa resistaria	ENT996-004162	GWNR294-07	HQ648936	Canada	British Columbia	Royal British Columbia Museum
Nematocampa resistaria	JECW-07-0113	GWNJ113-07	HQ648931	United States	Idaho	James Entomological Collection, Washington State University
Nematocampa resistaria	JECW-07-0114	GWNJ114-07	HQ648930	United States	Washington	James Entomological Collection, Washington State University
Nematocampa resistaria	UASM41325	GWNS228-07	HQ648933	Canada	Alberta	Strickland Museum of Entomology, University of Alberta
Nematocampa resistaria	UASM43333	GWNS229-07	HQ648932	Canada	Alberta	Strickland Museum of Entomology, University of Alberta
Nematocampa resistaria	UASM7120	GWNS227-07	HQ648934	Canada	Alberta	Strickland Museum of Entomology, University of Alberta
Nemoria darwiniata	CNCLEP00033243	GWNC244-07	HQ648945	Canada	British Columbia	Canadian National Collection of Insects, Arachnids and Nematodes
Nemoria darwiniata	CNCLEP00033244	GWNC245-07	HQ648946	United States	Idaho	Canadian National Collection of Insects, Arachnids and Nematodes
Nemoria darwiniata	ENT996-004179	GWNR355-07	HQ648950	Canada	British Columbia	Royal British Columbia Museum
Nemoria darwiniata	ENT996-004187	GWNR354-07	HQ648951	Canada	British Columbia	Royal British Columbia Museum
Nemoria darwiniata	ENT996-004199	GWNR356-07	HQ648949	Canada	British Columbia	Royal British Columbia Museum
Nemoria darwiniata	JECW-07-0121	GWNJ121-07	HQ648947	United States	Washington	James Entomological Collection, Washington State University
Nemoria darwiniata	UASM38077	GWNS235-07	HQ648948	Canada	British Columbia	Strickland Museum of Entomology, University of Alberta
Nemoria glaucomarginaria	JECW-07-0120	GWNJ120-07	HQ648952	United States	Washington	James Entomological Collection, Washington State University
Nemoria mimosaria	CNCLEP00033368	GWNC369-07	HQ648953	Canada	Alberta	Canadian National Collection of Insects, Arachnids and Nematodes
Nemoria mimosaria	UASM58029	GWNS233-07	HQ648955	Canada	Alberta	Strickland Museum of Entomology, University of Alberta
Nemoria mimosaria	UASM7155	GWNS234-07	HQ648954	Canada	Alberta	Strickland Museum of Entomology, University of Alberta
Nemoria rubrifrontaria	UASM41610	GWNS236-07	HQ648956	Canada	Alberta	Strickland Museum of Entomology, University of Alberta
Nemoria unitaria	08-JDWBC-2138	LBCG2138-09	HQ648965	Canada	British Columbia	Spencer Entomological Museum, UBC
Nemoria unitaria	CNCLEP00033242	GWNC243-07	HQ648957	Canada	British Columbia	Canadian National Collection of Insects, Arachnids and Nematodes
Nemoria unitaria	ENT996-004177	GWNR360-07	HQ648959	Canada	British Columbia	Royal British Columbia Museum
Nemoria unitaria	ENT996-004178	GWNR361-07	HQ648964	Canada	British Columbia	Royal British Columbia Museum
Nemoria unitaria	ENT996-007598	GWNR362-07	HQ648963	Canada	British Columbia	Royal British Columbia Museum
Nemoria unitaria	PFC-2007-0211	GWNP004-07	HQ648958	Canada	British Columbia	Pacific Forestry Centre, Canadian Forest Service
Nemoria unitaria	UASM41585	GWNS231-07	HQ648961	Canada	Alberta	Strickland Museum of Entomology, University of Alberta
Nemoria unitaria	UASM58124	GWNS230-07	HQ648962	Canada	Alberta	Strickland Museum of Entomology, University of Alberta
Nemoria unitaria	UASM58137	GWNS232-07	HQ648960	Canada	Alberta	Strickland Museum of Entomology, University of Alberta

Neoalcis californiaria	Dun-08-124	DUNLP124-08	HQ648966	Canada	British Columbia	Pacific Forestry Centre, Canadian Forest Service
Neoalcis californiaria	ENT996-003421	GWNR105-07	HQ648971	United States	Washington	Royal British Columbia Museum
Neoalcis californiaria	ENT996-003423	GWNR108-07	HQ648970	United States	Washington	Royal British Columbia Museum
Neoalcis californiaria	PFC-2007-0367	GWNP157-07	HQ648969	Canada	British Columbia	Pacific Forestry Centre, Canadian Forest Service
Neoalcis californiaria	PFC-2007-0368	GWNP158-07	HQ648968	Canada	British Columbia	Pacific Forestry Centre, Canadian Forest Service
Neoalcis californiaria	UASM59628	GWNS070-07	HQ648967	Canada	Alberta	Strickland Museum of Entomology, University of Alberta
Neoterpes trianguliferata	CBCC1055	GWNS192-07	HQ648975	Canada	Alberta	Strickland Museum of Entomology, University of Alberta
Neoterpes trianguliferata	CNCLEP00033203	GWNC204-07	HQ648972	Canada	British Columbia	Canadian National Collection of Insects, Arachnids and Nematodes
Neoterpes trianguliferata	ENT996-004022	GWNR297-07	HQ648976	Canada	British Columbia	Royal British Columbia Museum
Neoterpes trianguliferata	ENT996-004024	GWNR296-07	HQ648977	Canada	British Columbia	Royal British Columbia Museum
Neoterpes trianguliferata	UASM24814	GWNS194-07	HQ648973	Canada	Alberta	Strickland Museum of Entomology, University of Alberta
Neoterpes trianguliferata	UASM58783	GWNS193-07	HQ648974	Canada	Alberta	Strickland Museum of Entomology, University of Alberta
Nepytia canosaria	UASM24445	GWNS202-07	HQ648979	Canada	Alberta	Strickland Museum of Entomology, University of Alberta
Nepytia canosaria	UASM41851	GWNS203-07	HQ648978	Canada	Alberta	Strickland Museum of Entomology, University of Alberta
Nepytia freemani	ENT991-067681	GWNR299-07	HQ648985	Canada	British Columbia	Royal British Columbia Museum
Nepytia freemani	ENT996-004045	GWNR301-07	HQ648983	Canada	British Columbia	Royal British Columbia Museum
Nepytia freemani	ENT996-004047	GWNR300-07	HQ648984	Canada	British Columbia	Royal British Columbia Museum
Nepytia freemani	JECW-07-0089	GWNJ089-07	HQ648980	United States	Idaho	James Entomological Collection, Washington State University
Nepytia freemani	PFC-2007-0394	GWNP184-07	HQ648982	Canada	British Columbia	Pacific Forestry Centre, Canadian Forest Service
Nepytia freemani	PFC-2007-0395	GWNP185-07	HQ648981	Canada	British Columbia	Pacific Forestry Centre, Canadian Forest Service
Nepytia phantasmaria	JECW-07-0090	GWNJ090-07	HQ648986	United States	Washington	James Entomological Collection, Washington State University
Nepytia phantasmaria	PFC-2007-0396	GWNP186-07	HQ648989	Canada	British Columbia	Pacific Forestry Centre, Canadian Forest Service
Nepytia phantasmaria	PFC-2007-0397	GWNP187-07	HQ648988	Canada	British Columbia	Pacific Forestry Centre, Canadian Forest Service
Nepytia phantasmaria	PFC-2007-0398	GWNP188-07	HQ648987	Canada	British Columbia	Pacific Forestry Centre, Canadian Forest Service
Nepytia umbrosaria	CNCLEP00033365	GWNC366-07	HQ648991	Canada	British Columbia	Canadian National Collection of Insects, Arachnids and Nematodes
Nepytia umbrosaria	Dun-08-126	DUNLP126-08	HQ648990	Canada	British Columbia	Pacific Forestry Centre, Canadian Forest Service
Nepytia umbrosaria	ENT996-004036	GWNR303-07	HQ648993	United States	Washington	Royal British Columbia Museum
Nepytia umbrosaria	PFC-2007-0392	GWNP182-07	HQ648992	Canada	British Columbia	Pacific Forestry Centre, Canadian Forest Service
Operophtera bruceata	ENT002-001754	GWNR601-07	HQ648999	Canada	British Columbia	Royal British Columbia Museum
Operophtera bruceata	ENT996-005200	GWNR599-07	HQ649000	Canada	British Columbia	Royal British Columbia Museum
Operophtera bruceata	ENT996-005205	GWNR597-07	HQ649001	Canada	British Columbia	Royal British Columbia Museum
Operophtera bruceata	JECW-07-0181	GWNJ181-07	HQ648998	United States	Washington	James Entomological Collection, Washington State University
Operophtera bruceata	PFC-2007-0234	GWNP027-07	HQ648996	Canada	British Columbia	Pacific Forestry Centre, Canadian Forest Service
Operophtera bruceata	UASM24791	GWNS408-07	HQ648994	Canada	Alberta	Strickland Museum of Entomology, University of Alberta
Operophtera bruceata	UASM58424	GWNS407-07	HQ648995	Canada	Alberta	Strickland Museum of Entomology, University of Alberta
Operophtera bruceata	WFBM-07-0086	GWNW086-07	HQ648997	United States	Idaho	University of Idaho, W. F. Barr Entomological Collection
Operophtera bruceata occidentalis	09-JDWGEO-451	GNAU151-10	HM392428	United States	Alaska	Smithsonian Institution
Operophtera bruceata occidentalis	09-JDWGEO-452	GNAU152-10	HM392429	United States	Alaska	Smithsonian Institution
Operophtera bruceata occidentalis	09-JDWGEO-453	GNAU153-10	HM392430	United States	Oregon	Smithsonian Institution
Operophtera bruceata occidentalis	09-JDWGEO-455	GNAU155-10	HM392431	United States	Oregon	Smithsonian Institution
Operophtera brumata	08-JDWBC-3383	LBCG3383-09	HQ649012	Canada	British Columbia	Spencer Entomological Museum, UBC
Operophtera brumata	08-JDWBC-3395	LBCG3395-09	HQ649011	Canada	British Columbia	Spencer Entomological Museum, UBC
Operophtera brumata	08-JDWBC-3429	LBCG3429-09	HQ649010	Canada	British Columbia	Spencer Entomological Museum, UBC
Operophtera brumata	08-JDWBC-3430	LBCG3430-09	HQ649009	Canada	British Columbia	Spencer Entomological Museum, UBC
Operophtera brumata	08-JDWBC-3433	LBCG3433-09	HQ649008	Canada	British Columbia	Spencer Entomological Museum, UBC
Operophtera brumata	08-JDWBC-3434	LBCG3434-09	HQ649007	Canada	British Columbia	Spencer Entomological Museum, UBC
Operophtera brumata	08-JDWBC-3436	LBCG3436-09	HQ649006	Canada	British Columbia	Spencer Entomological Museum, UBC
Operophtera brumata	CNCLEP00020699	GWNC709-07	HQ649002	Canada	British Columbia	Canadian National Collection of Insects, Arachnids and Nematodes
Operophtera brumata	CNCLEP00035473	GWNC707-07	HQ649003	Canada	British Columbia	Canadian National Collection of Insects, Arachnids and Nematodes
Operophtera brumata	PFC-2007-0235	GWNP028-07	HQ649005	Canada	British Columbia	Pacific Forestry Centre, Canadian Forest Service
Operophtera brumata	PFC-2007-0236	GWNP029-07	HQ649004	Canada	British Columbia	Pacific Forestry Centre, Canadian Forest Service
Operophtera danbyi	09-JDWGEO-456	GNAU156-10	HM392432	United States	Oregon	Smithsonian Institution
Operophtera danbyi	09-JDWGEO-458	GNAU158-10	HM392433	United States	Oregon	Smithsonian Institution
Orthofidonia exornata	CNCLEP00033359	GWNC360-07	HQ649013	Canada	British Columbia	Canadian National Collection of Insects, Arachnids and Nematodes
Orthofidonia exornata	ENT002-001543	GWNR110-07	HQ649014	Canada	British Columbia	Royal British Columbia Museum
Orthofidonia exornata	ENT996-003405	GWNR109-07	HQ649015	Canada	British Columbia	Royal British Columbia Museum
Orthofidonia tinctaria	NFRC-P-2007-100014	GWNN014-07	HQ649018	Canada	Alberta	Northern Forestry Centre, Canadian Forest Service
Orthofidonia tinctaria	UASM58368	GWNS067-07	HQ649016	Canada	Alberta	Strickland Museum of Entomology, University of Alberta
Orthofidonia tinctaria	UASM7010	GWNS066-07	HQ649017	Canada	Alberta	Strickland Museum of Entomology, University of Alberta
Orthonama evansi	CNCLEP00033400	GWNC401-07	HQ649019	Canada	Alberta	Canadian National Collection of Insects, Arachnids and Nematodes
Orthonama evansi	NFRC-P-2004-005616	GWNN108-07	HQ649020	Canada	Alberta	Northern Forestry Centre, Canadian Forest Service
Orthonama evansi	UASM19627	GWNS397-07	HQ649022	Canada	Alberta	Strickland Museum of Entomology, University of Alberta
Orthonama evansi	UASM24592	GWNS398-07	HQ649021	Canada	Alberta	Strickland Museum of Entomology, University of Alberta
Orthonama obstipata	JECW-07-0167	GWNJ167-07	HQ649023	United States	Washington	James Entomological Collection, Washington State University
Pasiphila rectangulata	CNCLEP00021542	GWNC710-07	HQ649024	Canada	British Columbia	Canadian National Collection of Insects, Arachnids and Nematodes
Pasiphila rectangulata	JECW-07-0174	GWNJ174-07	HQ649028	United States	Washington	James Entomological Collection, Washington State University
Pasiphila rectangulata	PFC-2007-0279	GWNP072-07	HQ649027	Canada	British Columbia	Pacific Forestry Centre, Canadian Forest Service
Pasiphila rectangulata	PFC-2007-0280	GWNP073-07	HQ649026	Canada	British Columbia	Pacific Forestry Centre, Canadian Forest Service
Pasiphila rectangulata	PFC-2007-0281	GWNP074-07	HQ649025	Canada	British Columbia	Pacific Forestry Centre, Canadian Forest Service
Perizoma basaliata	CNCLEP00033409	GWNC410-07	HQ649029	Canada	Alberta	Canadian National Collection of Insects, Arachnids and Nematodes
Perizoma basaliata	UASM95819	GWNS355-07	HQ649031	Canada	Alberta	Strickland Museum of Entomology, University of Alberta
Perizoma basaliata	UASM95820	GWNS356-07	HQ649030	Canada	Alberta	Strickland Museum of Entomology, University of Alberta
Perizoma costiguttata	ENT996-004581	GWNR486-07	HQ649035	Canada	British Columbia	Royal British Columbia Museum
Perizoma costiguttata	JECW-07-0150	GWNJ150-07	HQ649033	United States	Washington	James Entomological Collection, Washington State University
Perizoma costiguttata	JECW-07-0151	GWNJ151-07	HQ649032	United States	Idaho	James Entomological Collection, Washington State University
Perizoma costiguttata	PFC-2007-0334	GWNP124-07	HQ649034	Canada	British Columbia	Pacific Forestry Centre, Canadian Forest Service
Perizoma curvilinea	ENT996-004570	GWNR487-07	HQ649039	United States	Washington	Royal British Columbia Museum
Perizoma curvilinea	ENT996-004572	GWNR489-07	HQ649038	United States	Washington	Royal British Columbia Museum
Perizoma curvilinea	ENT996-004586	GWNR485-07	HQ649040	United States	Washington	Royal British Columbia Museum
Perizoma curvilinea	HLC-22938	LBCD118-05	HQ649036	Canada	British Columbia	Biodiversity Institute of Ontario
Perizoma curvilinea	JECW-07-0152	GWNJ152-07	HQ649037	United States	Washington	James Entomological Collection, Washington State University
Perizoma custodiata	ENT002-001723	GWNR569-07	HQ649047	Canada	British Columbia	Royal British Columbia Museum
Perizoma custodiata	JD0085	GWNS501-07	HQ649046	Canada	Alberta	Strickland Museum of Entomology, University of Alberta
Perizoma custodiata	JD0122	GWNS503-07	HQ649044	Canada	Alberta	Strickland Museum of Entomology, University of Alberta
Perizoma custodiata	JD0123	GWNS502-07	HQ649045	Canada	Alberta	Strickland Museum of Entomology, University of Alberta
Perizoma custodiata	UASM57478	GWNS360-07	HQ649041	Canada	Alberta	Strickland Museum of Entomology, University of Alberta
Perizoma custodiata	UASM58053	GWNS358-07	HQ649043	Canada	Alberta	Strickland Museum of Entomology, University of Alberta
Perizoma custodiata	UASM58656	GWNS359-07	HQ649042	Canada	Alberta	Strickland Museum of Entomology, University of Alberta
Perizoma grandis	ENT987-000065	GWNR492-07	HQ649051	Canada	British Columbia	Royal British Columbia Museum
Perizoma grandis	ENT996-004549	GWNR491-07	HQ649052	Canada	British Columbia	Royal British Columbia Museum
Perizoma grandis	ENT996-004602	GWNR494-07	HQ649050	United States	Washington	Royal British Columbia Museum
Perizoma grandis	HLC-23028	LBCD208-05	HQ649048	Canada	British Columbia	Biodiversity Institute of Ontario
Perizoma grandis	UASM58892	GWNS357-07	HQ649049	Canada	Alberta	Strickland Museum of Entomology, University of Alberta
Perizoma sp. 1	JECW-07-0158	GWNJ158-07	HQ649053	United States	Washington	James Entomological Collection, Washington State University
Perizoma sp. 2	JECW-07-0153	GWNJ153-07	HQ649055	United States	Washington	James Entomological Collection, Washington State University
Perizoma sp. 2	JECW-07-0154	GWNJ154-07	HQ649054	United States	Washington	James Entomological Collection, Washington State University
Pero behrensaria	Dun-08-127	DUNLP127-08	HQ649056	Canada	British Columbia	Pacific Forestry Centre, Canadian Forest Service
Pero behrensaria	ENT996-003865	GWNR203-07	HQ649060	Canada	British Columbia	Royal British Columbia Museum
Pero behrensaria	ENT996-003868	GWNR204-07	HQ649059	Canada	British Columbia	Royal British Columbia Museum
Pero behrensaria	ENT996-003887	GWNR202-07	HQ649061	United States	Washington	Royal British Columbia Museum
Pero behrensaria	UASM95801	GWNS143-07	HQ649058	Canada	British Columbia	Strickland Museum of Entomology, University of Alberta
Pero behrensaria	UASM95802	GWNS144-07	HQ649057	Canada	British Columbia	Strickland Museum of Entomology, University of Alberta
Pero honestaria	ENT002-001602	GWNR205-07	HQ649065	Canada	British Columbia	Royal British Columbia Museum
Pero honestaria	UASM41324	GWNS134-07	HQ649064	Canada	Alberta	Strickland Museum of Entomology, University of Alberta
Pero honestaria	UASM41790	GWNS136-07	HQ649062	Canada	Alberta	Strickland Museum of Entomology, University of Alberta
Pero honestaria	UASM56984	GWNS135-07	HQ649063	Canada	Alberta	Strickland Museum of Entomology, University of Alberta
Pero mizon	08-JDWBC-0239	LBCG239-08	HQ649075	Canada	British Columbia	Spencer Entomological Museum, UBC

Pero mizon	08-JDWBC-1066	LBCG1066-09	HQ649079	Canada	British Columbia	Spencer Entomological Museum, UBC
Pero mizon	08-JDWBC-1067	LBCG1067-09	HQ649078	Canada	British Columbia	Spencer Entomological Museum, UBC
Pero mizon	08-JDWBC-1072	LBCG1072-09	HQ649077	Canada	British Columbia	Spencer Entomological Museum, UBC
Pero mizon	08-JDWBC-1075	LBCG1075-09	HQ649076	Canada	British Columbia	Spencer Entomological Museum, UBC
Pero mizon	08-JDWBC-2104	LBCG2104-09	HQ649084	Canada	British Columbia	Spencer Entomological Museum, UBC
Pero mizon	CNCLEP00033168	GWNC169-07	HQ649070	Canada	British Columbia	Canadian National Collection of Insects, Arachnids and Nematodes
Pero mizon	CNCLEP00033170	GWNC171-07	HQ649069	Canada	British Columbia	Canadian National Collection of Insects, Arachnids and Nematodes
Pero mizon	CNCLEP00033171	GWNC172-07	HQ649068	Canada	British Columbia	Canadian National Collection of Insects, Arachnids and Nematodes
Pero mizon	CNCLEP00033361	GWNC362-07	HQ649067	Canada	British Columbia	Canadian National Collection of Insects, Arachnids and Nematodes
Pero mizon	Dun-08-128	DUNLP128-08	HQ649066	Canada	British Columbia	Pacific Forestry Centre, Canadian Forest Service
Pero mizon	ENT996-003835	GWNR210-07	HQ649080	United States	Washington	Royal British Columbia Museum
Pero mizon	ENT996-003838	GWNR207-07	HQ649082	United States	Washington	Royal British Columbia Museum
Pero mizon	ENT996-003840	GWNR206-07	HQ649083	United States	Washington	Royal British Columbia Museum
Pero mizon	ENT996-003842	GWNR209-07	HQ649081	Canada	British Columbia	Royal British Columbia Museum
Pero mizon	JECW-07-0068	GWNJ068-07	HQ649074	United States	Idaho	James Entomological Collection, Washington State University
Pero mizon	JECW-07-0069	GWNJ069-07	HQ649073	United States	Washington	James Entomological Collection, Washington State University
Pero mizon	JECW-07-0070	GWNJ070-07	HQ649072	United States	Washington	James Entomological Collection, Washington State University
Pero mizon	JECW-07-0071	GWNJ071-07	HQ649071	United States	Washington	James Entomological Collection, Washington State University
Pero morrisonaria	ENT002-001603	GWNR215-07	HQ649087	Canada	British Columbia	Royal British Columbia Museum
Pero morrisonaria	ENT996-003854	GWNR214-07	HQ649088	Canada	British Columbia	Royal British Columbia Museum
Pero morrisonaria	ENT996-003864	GWNR211-07	HQ649089	United States	Washington	Royal British Columbia Museum
Pero morrisonaria	UASM41994	GWNS140-07	HQ649085	Canada	Alberta	Strickland Museum of Entomology, University of Alberta
Pero morrisonaria	UASM7357	GWNS139-07	HQ649086	Canada	Alberta	Strickland Museum of Entomology, University of Alberta
Pero occidentalis	08-JDWBC-0028	LBCG028-08	HQ649097	Canada	British Columbia	Spencer Entomological Museum, UBC
Pero occidentalis	08-JDWBC-0029	LBCG029-08	HQ649096	Canada	British Columbia	Spencer Entomological Museum, UBC
Pero occidentalis	08-JDWBC-0030	LBCG030-08	HQ649095	Canada	British Columbia	Spencer Entomological Museum, UBC
Pero occidentalis	08-JDWBC-0333	LBCG333-08	HQ649094	Canada	British Columbia	Spencer Entomological Museum, UBC
Pero occidentalis	08-JDWBC-0359	LBCG359-08	HQ649093	Canada	British Columbia	Spencer Entomological Museum, UBC
Pero occidentalis	CBCC1057	GWNS141-07	HQ649090	Canada	Alberta	Strickland Museum of Entomology, University of Alberta
Pero occidentalis	ENT996-003889	GWNR216-07	HQ649098	Canada	British Columbia	Royal British Columbia Museum
Pero occidentalis	UASM41795	GWNS137-07	HQ649092	Canada	Alberta	Strickland Museum of Entomology, University of Alberta
Pero occidentalis	UASM58779	GWNS138-07	HQ649091	Canada	Alberta	Strickland Museum of Entomology, University of Alberta
Phaeoura mexicanaria	08-JDWBC-0410	LBCG410-08	HQ649101	Canada	British Columbia	Spencer Entomological Museum, UBC
Phaeoura mexicanaria	08-JDWBC-1064	LBCG1064-09	HQ649100	Canada	British Columbia	Spencer Entomological Museum, UBC
Phaeoura mexicanaria	Dun-08-122	DUNLP122-08	HQ649099	Canada	British Columbia	Pacific Forestry Centre, Canadian Forest Service
Phaeoura quernaria	UASM24079	GWNS145-07	HQ649102	Canada	Alberta	Strickland Museum of Entomology, University of Alberta
Phigalia plumogeraria	CNCLEP00033122	GWNC123-07	HQ649103	Canada	British Columbia	Canadian National Collection of Insects, Arachnids and Nematodes
Phigalia plumogeraria	JECW-07-0046	GWNJ046-07	HQ649104	United States	Washington	James Entomological Collection, Washington State University
Phigalia plumogeraria	PFC-2007-0377	GWNP167-07	HQ649106	Canada	British Columbia	Pacific Forestry Centre, Canadian Forest Service
Phigalia plumogeraria	PFC-2007-0378	GWNP168-07	HQ649105	Canada	British Columbia	Pacific Forestry Centre, Canadian Forest Service
Philedia punctomacularia	ENT996-003963	GWNR245-07	HQ649110	Canada	British Columbia	Royal British Columbia Museum
Philedia punctomacularia	ENT996-003965	GWNR244-07	HQ649111	Canada	British Columbia	Royal British Columbia Museum
Philedia punctomacularia	JECW-07-0080	GWNJ080-07	HQ649108	Canada	British Columbia	James Entomological Collection, Washington State University
Philedia punctomacularia	JECW-07-0081	GWNJ081-07	HQ649107	Canada	British Columbia	James Entomological Collection, Washington State University
Philedia punctomacularia	PFC-2007-0385	GWNP175-07	HQ649109	Canada	British Columbia	Pacific Forestry Centre, Canadian Forest Service
Plagodis alcoolaria	UASM59681	GWNS189-07	HQ649114	Canada	Alberta	Strickland Museum of Entomology, University of Alberta
Plagodis alcoolaria	UASM7114	GWNS191-07	HQ649112	Canada	Alberta	Strickland Museum of Entomology, University of Alberta
Plagodis alcoolaria	UASM78417	GWNS190-07	HQ649113	Canada	Alberta	Strickland Museum of Entomology, University of Alberta
Plagodis phlogosaria	08-JDWBC-0004	LBCG004-08	HQ649118	Canada	British Columbia	Spencer Entomological Museum, UBC
Plagodis phlogosaria	ENT996-004015	GWNR260-07	HQ649120	Canada	British Columbia	Royal British Columbia Museum
Plagodis phlogosaria	ENT996-004017	GWNR261-07	HQ649119	Canada	British Columbia	Royal British Columbia Museum
Plagodis phlogosaria	ENT996-004018	GWNR259-07	HQ649121	Canada	British Columbia	Royal British Columbia Museum
Plagodis phlogosaria	UASM57998	GWNS187-07	HQ649116	Canada	Alberta	Strickland Museum of Entomology, University of Alberta
Plagodis phlogosaria	UASM58055	GWNS188-07	HQ649115	Canada	Alberta	Strickland Museum of Entomology, University of Alberta
Plagodis phlogosaria	UASM78418	GWNS186-07	HQ649117	Canada	Alberta	Strickland Museum of Entomology, University of Alberta
Plagodis pulveraria	08-JDWBC-0273	LBCG273-08	HQ649126	Canada	British Columbia	Spencer Entomological Museum, UBC
Plagodis pulveraria	CNCLEP00033194	GWNC195-07	HQ649122	Canada	Alberta	Canadian National Collection of Insects, Arachnids and Nematodes
Plagodis pulveraria	ENT002-001622	GWNR252-07	HQ649127	Canada	British Columbia	Royal British Columbia Museum
Plagodis pulveraria	ENT996-003972	GWNR250-07	HQ649129	Canada	British Columbia	Royal British Columbia Museum
Plagodis pulveraria	ENT996-003977	GWNR251-07	HQ649128	Canada	British Columbia	Royal British Columbia Museum
Plagodis pulveraria	UASM43261	GWNS178-07	HQ649123	Canada	Alberta	Strickland Museum of Entomology, University of Alberta
Plagodis pulveraria	UASM7111	GWNS176-07	HQ649125	Canada	Alberta	Strickland Museum of Entomology, University of Alberta
Plagodis pulveraria	UASM78374	GWNS177-07	HQ649124	Canada	Alberta	Strickland Museum of Entomology, University of Alberta
Plataea trilinearia	08-JDWBC-0388	LBCG388-08	HQ649137	Canada	British Columbia	Spencer Entomological Museum, UBC
Plataea trilinearia	ENT991-067013	GWNR306-07	HQ649136	Canada	British Columbia	Royal British Columbia Museum
Plataea trilinearia	ENT996-004114	GWNR308-07	HQ649135	United States	Washington	Royal British Columbia Museum
Plataea trilinearia	JECW-07-0093	GWNJ093-07	HQ649131	United States	Idaho	James Entomological Collection, Washington State University
Plataea trilinearia	JECW-07-0094	GWNJ094-07	HQ649130	United States	Washington	James Entomological Collection, Washington State University
Plataea trilinearia	UASM56974	GWNS209-07	HQ649132	Canada	Alberta	Strickland Museum of Entomology, University of Alberta
Plataea trilinearia	UASM57124	GWNS208-07	HQ649133	Canada	Alberta	Strickland Museum of Entomology, University of Alberta
Plataea trilinearia	UASM57504	GWNS207-07	HQ649134	Canada	Alberta	Strickland Museum of Entomology, University of Alberta
Plemyria georgii	CNCLEP00033381	GWNC382-07	HQ649138	Canada	Alberta	Canadian National Collection of Insects, Arachnids and Nematodes
Plemyria georgii	ENT002-001702	GWNR499-07	HQ649141	Canada	British Columbia	Royal British Columbia Museum
Plemyria georgii	ENT996-004462	GWNR497-07	HQ649143	United States	Washington	Royal British Columbia Museum
Plemyria georgii	ENT996-004463	GWNR498-07	HQ649142	United States	Washington	Royal British Columbia Museum
Plemyria georgii	PFC-2007-0287	GWNP080-07	HQ649139	Canada	British Columbia	Pacific Forestry Centre, Canadian Forest Service
Plemyria georgii	UASM7321	GWNS314-07	HQ649140	Canada	Alberta	Strickland Museum of Entomology, University of Alberta
Probole alienaria	CNCLEP00033197	GWNC198-07	HQ649144	Canada	British Columbia	Canadian National Collection of Insects, Arachnids and Nematodes
Probole alienaria	UASM41552	GWNS603-07	HQ649149	Canada	Alberta	Strickland Museum of Entomology, University of Alberta
Probole alienaria	UASM57021	GWNS184-07	HQ649146	Canada	Alberta	Strickland Museum of Entomology, University of Alberta
Probole alienaria	UASM57023	GWNS183-07	HQ649147	Canada	Alberta	Strickland Museum of Entomology, University of Alberta
Probole alienaria	UASM57255	GWNS182-07	HQ649148	Canada	Alberta	Strickland Museum of Entomology, University of Alberta
Probole alienaria	UASM57961	GWNS185-07	HQ649145	Canada	Alberta	Strickland Museum of Entomology, University of Alberta
Probole amicaria	ENT002-001623	GWNR265-07	HQ649154	Canada	British Columbia	Royal British Columbia Museum
Probole amicaria	ENT002-001624	GWNR264-07	HQ649155	Canada	British Columbia	Royal British Columbia Museum
Probole amicaria	ENT996-003984	GWNR262-07	HQ649156	Canada	British Columbia	Royal British Columbia Museum
Probole amicaria	JECW-07-0083	GWNJ083-07	HQ649150	United States	Washington	James Entomological Collection, Washington State University
Probole amicaria	UASM24040	GWNS179-07	HQ649153	Canada	Alberta	Strickland Museum of Entomology, University of Alberta
Probole amicaria	UASM7354	GWNS180-07	HQ649152	Canada	Alberta	Strickland Museum of Entomology, University of Alberta
Probole amicaria	UASM7400	GWNS181-07	HQ649151	Canada	Alberta	Strickland Museum of Entomology, University of Alberta
Prochoerodes amplicineraria	CNCLEP00033227	GWNC228-07	HQ649160	United States	Oregon	Canadian National Collection of Insects, Arachnids and Nematodes
Prochoerodes amplicineraria	JECW-07-0102	GWNJ102-07	HQ649159	United States	Washington	James Entomological Collection, Washington State University
Prochoerodes amplicineraria	JECW-07-0103	GWNJ103-07	HQ649158	United States	Washington	James Entomological Collection, Washington State University
Prochoerodes amplicineraria	JECW-07-0104	GWNJ104-07	HQ649157	United States	Washington	James Entomological Collection, Washington State University
Prochoerodes forficaria	08-JDWBC-0021	LBCG021-08	HQ649175	Canada	British Columbia	Spencer Entomological Museum, UBC
Prochoerodes forficaria	08-JDWBC-0022	LBCG022-08	HQ649174	Canada	British Columbia	Spencer Entomological Museum, UBC
Prochoerodes forficaria	08-JDWBC-0095	LBCG095-08	HQ649173	Canada	British Columbia	Spencer Entomological Museum, UBC
Prochoerodes forficaria	08-JDWBC-0098	LBCG098-08	HQ649172	Canada	British Columbia	Spencer Entomological Museum, UBC
Prochoerodes forficaria	08-JDWBC-0099	LBCG099-08	HQ649171	Canada	British Columbia	Spencer Entomological Museum, UBC
Prochoerodes forficaria	08-JDWBC-0100	LBCG100-08	HQ649170	Canada	British Columbia	Spencer Entomological Museum, UBC
Prochoerodes forficaria	08-JDWBC-0102	LBCG102-08	HQ649169	Canada	British Columbia	Spencer Entomological Museum, UBC
Prochoerodes forficaria	08-JDWBC-0107	LBCG107-08	HQ649168	Canada	British Columbia	Spencer Entomological Museum, UBC
Prochoerodes forficaria	CNCLEP00033229	GWNC230-07	HQ649161	Canada	British Columbia	Canadian National Collection of Insects, Arachnids and Nematodes
Prochoerodes forficaria	ENT996-004142	GWNR312-07	HQ649166	Canada	British Columbia	Royal British Columbia Museum
Prochoerodes forficaria	ENT996-004144	GWNR313-07	HQ649165	Canada	British Columbia	Royal British Columbia Museum
Prochoerodes forficaria	ENT996-004148	GWNR311-07	HQ649167	United States	Idaho	Royal British Columbia Museum
Prochoerodes forficaria	UASM41902	GWNS222-07	HQ649163	Canada	Alberta	Strickland Museum of Entomology, University of Alberta

Prochoerodes forficaria	UASM59317	GWNS223-07	HQ649162	Canada	Alberta	Strickland Museum of Entomology, University of Alberta
Prochoerodes forficaria	UASM95878	GWNS549-07	HQ649164	Canada	Alberta	Strickland Museum of Entomology, University of Alberta
Prochoerodes lineola	ENT002-001635	GWNR315-07	HQ649180	Canada	British Columbia	Royal British Columbia Museum
Prochoerodes lineola	ENT002-001636	GWNR317-07	HQ649178	Canada	British Columbia	Royal British Columbia Museum
Prochoerodes lineola	ENT002-001638	GWNR316-07	HQ649179	Canada	British Columbia	Royal British Columbia Museum
Prochoerodes lineola	UASM57974	GWNS225-07	HQ649177	Canada	Alberta	Strickland Museum of Entomology, University of Alberta
Prochoerodes lineola	UASM58360	GWNS226-07	HQ649176	Canada	Alberta	Strickland Museum of Entomology, University of Alberta
Prorella leucata	CGWC-1138	LOWCB198-05	HQ649184	Canada	British Columbia	Biodiversity Institute of Ontario
Prorella leucata	CGWC-1150	LOWCB210-05	HQ649183	Canada	British Columbia	Biodiversity Institute of Ontario
Prorella leucata	CGWC-1193	LOWCB253-05	HQ649182	Canada	British Columbia	Biodiversity Institute of Ontario
Prorella leucata	CGWC-1236	LOWCB296-05	HQ649181	Canada	British Columbia	Biodiversity Institute of Ontario
Prorella mellisa	10-GOBCL-01	GOBCL001-10	HQ649185	United States	Colorado	Barcode of Life Data System
Protitame matilda	CNCLEP00033006	GWNC007-07	HQ649186	Canada	British Columbia	Canadian National Collection of Insects, Arachnids and Nematodes
Protitame matilda	ENT991-010195	GWNR002-07	HQ649190	Canada	British Columbia	Royal British Columbia Museum
Protitame matilda	ENT996-003239	GWNR003-07	HQ649189	Canada	British Columbia	Royal British Columbia Museum
Protitame matilda	ENT996-003241	GWNR004-07	HQ649188	Canada	British Columbia	Royal British Columbia Museum
Protitame matilda	UASM59682	GWNS003-07	HQ649187	Canada	Alberta	Strickland Museum of Entomology, University of Alberta
Protitame virginalis	ENT002-001585	GWNR157-07	HQ649198	Canada	British Columbia	Royal British Columbia Museum
Protitame virginalis	ENT002-002417	GWNR007-07	HQ649195	Canada	British Columbia	Royal British Columbia Museum
Protitame virginalis	ENT996-003255	GWNR006-07	HQ649196	Canada	British Columbia	Royal British Columbia Museum
Protitame virginalis	ENT996-003259	GWNR005-07	HQ649197	Canada	British Columbia	Royal British Columbia Museum
Protitame virginalis	PFC-2007-0360	GWNP150-07	HQ649194	Canada	British Columbia	Pacific Forestry Centre, Canadian Forest Service
Protitame virginalis	PFC-2007-0361	GWNP151-07	HQ649193	Canada	British Columbia	Pacific Forestry Centre, Canadian Forest Service
Protitame virginalis	UASM59070	GWNS004-07	HQ649192	Canada	Alberta	Strickland Museum of Entomology, University of Alberta
Protitame virginalis	UASM95792	GWNS005-07	HQ649191	Canada	Alberta	Strickland Museum of Entomology, University of Alberta
Protoboarmia porcelaria	08-JDWBC-1320	LBCG1320-09	HQ649207	Canada	British Columbia	Spencer Entomological Museum, UBC
Protoboarmia porcelaria	08-JDWBC-3279	LBCG3279-09	HQ649206	Canada	British Columbia	Spencer Entomological Museum, UBC
Protoboarmia porcelaria	Dun-08-068	DUNLP068-08	HQ649199	Canada	British Columbia	Pacific Forestry Centre, Canadian Forest Service
Protoboarmia porcelaria	ENT996-003603	GWNR112-07	HQ649203	Canada	British Columbia	Royal British Columbia Museum
Protoboarmia porcelaria	ENT996-003607	GWNR111-07	HQ649204	Canada	British Columbia	Royal British Columbia Museum
Protoboarmia porcelaria	ENT996-007538	GWNR371-07	HQ649205	Canada	British Columbia	Royal British Columbia Museum
Protoboarmia porcelaria	NFRC-P-2007-100019	GWNN019-07	HQ649202	Canada	Alberta	Northern Forestry Centre, Canadian Forest Service
Protoboarmia porcelaria	UASM41078	GWNS082-07	HQ649201	Canada	Alberta	Strickland Museum of Entomology, University of Alberta
Protoboarmia porcelaria	UASM41079	GWNS083-07	HQ649200	Canada	Alberta	Strickland Museum of Entomology, University of Alberta
Psychophora phocata	CNCLEP00034133	GWNC571-07	HQ649209	Canada	Northwest Territories	Canadian National Collection of Insects, Arachnids and Nematodes
Psychophora phocata	CNCLEP00034135	GWNC573-07	HQ649208	Canada	Northwest Territories	Canadian National Collection of Insects, Arachnids and Nematodes
Psychophora sabini	CNCLEP00034127	GWNC565-07	HQ649213	Canada	Yukon Territory	Canadian National Collection of Insects, Arachnids and Nematodes
Psychophora sabini	CNCLEP00034128	GWNC566-07	HQ649212	Canada	Yukon Territory	Canadian National Collection of Insects, Arachnids and Nematodes
Psychophora sabini	CNCLEP00034132	GWNC570-07	HQ649211	Canada	Northwest Territories	Canadian National Collection of Insects, Arachnids and Nematodes
Psychophora sabini	CNCLEP00034134	GWNC572-07	HQ649210	Canada	Northwest Territories	Canadian National Collection of Insects, Arachnids and Nematodes
Psychophora sp. C	CNCLEP00034122	GWNC560-07	HQ649215	Canada	British Columbia	Canadian National Collection of Insects, Arachnids and Nematodes
Psychophora sp. C	CNCLEP00034123	GWNC561-07	HQ649214	Canada	British Columbia	Canadian National Collection of Insects, Arachnids and Nematodes
Psychophora sp. D	CNCLEP00034124	GWNC562-07	HQ649217	Canada	Yukon Territory	Canadian National Collection of Insects, Arachnids and Nematodes
Psychophora sp. D	CNCLEP00034125	GWNC563-07	HQ649216	Canada	Yukon Territory	Canadian National Collection of Insects, Arachnids and Nematodes
Psychophora sp. I	CNCLEP00034130	GWNC568-07	HQ649218	Canada	Yukon Territory	Canadian National Collection of Insects, Arachnids and Nematodes
Psychophora sp. I	CNCLEP00034131	GWNC569-07	HQ649219	Canada	Yukon Territory	Canadian National Collection of Insects, Arachnids and Nematodes
Psychophora suttoni	CNCLEP00033396	GWNC397-07	HQ649223	Canada	Yukon Territory	Canadian National Collection of Insects, Arachnids and Nematodes
Psychophora suttoni	CNCLEP00033397	GWNC398-07	HQ649221	Canada	British Columbia	Canadian National Collection of Insects, Arachnids and Nematodes
Psychophora suttoni	CNCLEP00034120	GWNC558-07	HQ649220	Canada	British Columbia	Canadian National Collection of Insects, Arachnids and Nematodes
Psychophora suttoni	CNCLEP00034121	GWNC559-07	HQ649222	Canada	Yukon Territory	Canadian National Collection of Insects, Arachnids and Nematodes
Rheumaptera hastata	CBCC1002	GWNS327-07	HQ649225	Canada	Alberta	Strickland Museum of Entomology, University of Alberta
Rheumaptera hastata	CBCC1003	GWNS328-07	HQ649224	Canada	Alberta	Strickland Museum of Entomology, University of Alberta
Rheumaptera hastata	ENT002-001704	GWNR503-07	HQ649226	Canada	British Columbia	Royal British Columbia Museum
Rheumaptera hastata	ENT991-010371	GWNR502-07	HQ649227	Canada	British Columbia	Royal British Columbia Museum
Rheumaptera hastata	ENT991-012205	GWNR501-07	HQ649228	Canada	British Columbia	Royal British Columbia Museum
Rheumaptera hastata	ENT993-000269	GWNR500-07	HQ649229	Canada	British Columbia	Royal British Columbia Museum
Rheumaptera subhastata	CNCLEP00034041	GWNC479-07	HQ649230	Canada	Alberta	Canadian National Collection of Insects, Arachnids and Nematodes
Rheumaptera subhastata	ENT991-004569	GWNR507-07	HQ649234	Canada	British Columbia	Royal British Columbia Museum
Rheumaptera subhastata	ENT991-010126	GWNR508-07	HQ649237	Canada	British Columbia	Royal British Columbia Museum
Rheumaptera subhastata	ENT991-012203	GWNR504-07	HQ649235	Canada	British Columbia	Royal British Columbia Museum
Rheumaptera subhastata	ENT991-162271	GWNR509-07	HQ649236	Canada	British Columbia	Royal British Columbia Museum
Rheumaptera subhastata	PFC-2007-0333	GWNP123-07	HQ649233	Canada	British Columbia	Pacific Forestry Centre, Canadian Forest Service
Rheumaptera subhastata	UASM41244	GWNS329-07	HQ649232	Canada	Alberta	Strickland Museum of Entomology, University of Alberta
Rheumaptera subhastata	UASM41248	GWNS330-07	HQ649231	Canada	Alberta	Strickland Museum of Entomology, University of Alberta
Rheumaptera undulata	ENT996-004377	GWNR513-07	HQ649242	Canada	British Columbia	Royal British Columbia Museum
Rheumaptera undulata	HLC-21942	LBCC062-05	HQ649238	Canada	British Columbia	Biodiversity Institute of Ontario
Rheumaptera undulata	PFC-2007-0248	GWNP041-07	HQ649241	Canada	British Columbia	Pacific Forestry Centre, Canadian Forest Service
Rheumaptera undulata	UASM7163	GWNS325-07	HQ649240	Canada	Alberta	Strickland Museum of Entomology, University of Alberta
Rheumaptera undulata	UASM95812	GWNS326-07	HQ649239	Canada	Alberta	Strickland Museum of Entomology, University of Alberta
Sabulodes edwardsata	CNCLEP00033230	GWNC231-07	HQ649249	United States	Washington	Canadian National Collection of Insects, Arachnids and Nematodes
Sabulodes edwardsata	CNCLEP00033366	GWNC367-07	HQ649248	Canada	British Columbia	Canadian National Collection of Insects, Arachnids and Nematodes
Sabulodes edwardsata	Dun-08-129	DUNLP129-08	HQ649245	Canada	British Columbia	Pacific Forestry Centre, Canadian Forest Service
Sabulodes edwardsata	Dun-08-130	DUNLP130-08	HQ649247	Canada	British Columbia	Pacific Forestry Centre, Canadian Forest Service
Sabulodes edwardsata	Dun-08-131	DUNLP131-08	HQ649243	Canada	British Columbia	Pacific Forestry Centre, Canadian Forest Service
Sabulodes edwardsata	Dun-08-132	DUNLP132-08	HQ649246	Canada	British Columbia	Pacific Forestry Centre, Canadian Forest Service
Sabulodes edwardsata	Dun-08-133	DUNLP133-08	HQ649244	Canada	British Columbia	Pacific Forestry Centre, Canadian Forest Service
Sabulodes edwardsata	ENT996-004161	GWNR320-07	HQ649254	Canada	British Columbia	Royal British Columbia Museum
Sabulodes edwardsata	JECW-07-0111	GWNJ111-07	HQ649251	United States	Idaho	James Entomological Collection, Washington State University
Sabulodes edwardsata	JECW-07-0112	GWNJ112-07	HQ649250	United States	Washington	James Entomological Collection, Washington State University
Sabulodes edwardsata	PFC-2007-0388	GWNP178-07	HQ649253	Canada	British Columbia	Pacific Forestry Centre, Canadian Forest Service
Sabulodes edwardsata	WFBM-07-0070	GWNW070-07	HQ649252	United States	Idaho	University of Idaho, W. F. Barr Entomological Collection
Scopula ancellata	CGWC-4358	LOWCE598-06	HQ649255	Canada	British Columbia	Biodiversity Institute of Ontario
Scopula ancellata	ENT002-002421	GWNR392-07	HQ649257	Canada	British Columbia	Royal British Columbia Museum
Scopula ancellata	ENT996-004354	GWNR391-07	HQ649258	Canada	British Columbia	Royal British Columbia Museum
Scopula ancellata	ENT996-004356	GWNR390-07	HQ649259	Canada	British Columbia	Royal British Columbia Museum
Scopula ancellata	UASM58924	GWNS254-07	HQ649256	Canada	Alberta	Strickland Museum of Entomology, University of Alberta
Scopula cacuminaria	UASM41306	GWNS253-07	HQ649260	Canada	Alberta	Strickland Museum of Entomology, University of Alberta
Scopula frigidaria	CNCLEP00033372	GWNC373-07	HQ649261	Canada	Alberta	Canadian National Collection of Insects, Arachnids and Nematodes
Scopula frigidaria	ENT002-002418	GWNR397-07	HQ649268	Canada	British Columbia	Royal British Columbia Museum
Scopula frigidaria	ENT996-004328	GWNR396-07	HQ649269	United States	Washington	Royal British Columbia Museum
Scopula frigidaria	UASM59260	GWNS265-07	HQ649262	Canada	Yukon Territory	Strickland Museum of Entomology, University of Alberta
Scopula frigidaria	UASM78463	GWNS264-07	HQ649263	Canada	Alberta	Strickland Museum of Entomology, University of Alberta
Scopula frigidaria	UASM95866	GWNS538-07	HQ649266	Canada	Alberta	Strickland Museum of Entomology, University of Alberta
Scopula frigidaria	UASM95867	GWNS539-07	HQ649265	Canada	Alberta	Strickland Museum of Entomology, University of Alberta
Scopula frigidaria	UASM95868	GWNS537-07	HQ649267	Canada	Alberta	Strickland Museum of Entomology, University of Alberta
Scopula frigidaria	UASM95869	GWNS540-07	HQ649264	Canada	Alberta	Strickland Museum of Entomology, University of Alberta
Scopula fuscata	WFBM-07-0076	GWNW076-07	HQ649270	United States	Idaho	University of Idaho, W. F. Barr Entomological Collection
Scopula inductata	08-JDWBC-0202	LBCG202-08	HQ649283	Canada	British Columbia	Spencer Entomological Museum, UBC
Scopula inductata	08-JDWBC-1307	LBCG1307-09	HQ649290	Canada	British Columbia	Spencer Entomological Museum, UBC
Scopula inductata	08-JDWBC-1308	LBCG1308-09	HQ649289	Canada	British Columbia	Spencer Entomological Museum, UBC
Scopula inductata	08-JDWBC-1309	LBCG1309-09	HQ649288	Canada	British Columbia	Spencer Entomological Museum, UBC
Scopula inductata	08-JDWBC-1311	LBCG1311-09	HQ649287	Canada	British Columbia	Spencer Entomological Museum, UBC
Scopula inductata	08-JDWBC-1312	LBCG1312-09	HQ649286	Canada	British Columbia	Spencer Entomological Museum, UBC
Scopula inductata	08-JDWBC-1313	LBCG1313-09	HQ649285	Canada	British Columbia	Spencer Entomological Museum, UBC
Scopula inductata	08-JDWBC-1314	LBCG1314-09	HQ649284	Canada	British Columbia	Spencer Entomological Museum, UBC
Scopula inductata	CBCC1021	GWNS271-07	HQ649274	Canada	Alberta	Strickland Museum of Entomology, University of Alberta
Scopula inductata	CNCLEP00033373	GWNC374-07	HQ649271	Canada	Alberta	Canadian National Collection of Insects, Arachnids and Nematodes

Scopula inductata	ENT002-001685	GWNR398-07	HQ649281	Canada	British Columbia	Royal British Columbia Museum
Scopula inductata	ENT002-001686	GWNR400-07	HQ649279	Canada	British Columbia	Royal British Columbia Museum
Scopula inductata	ENT002-001689	GWNR399-07	HQ649280	Canada	British Columbia	Royal British Columbia Museum
Scopula inductata	ENT996-004266	GWNR380-07	HQ649282	United States	Washington	Royal British Columbia Museum
Scopula inductata	JD1798	GWNS507-07	HQ649272	Canada	British Columbia	Strickland Museum of Entomology, University of Alberta
Scopula inductata	UASM41840	GWNS268-07	HQ649277	Canada	Alberta	Strickland Museum of Entomology, University of Alberta
Scopula inductata	UASM57241	GWNS270-07	HQ649275	Canada	Alberta	Strickland Museum of Entomology, University of Alberta
Scopula inductata	UASM58589	GWNS269-07	HQ649276	Canada	Alberta	Strickland Museum of Entomology, University of Alberta
Scopula inductata	UASM58658	GWNS267-07	HQ649278	Canada	Alberta	Strickland Museum of Entomology, University of Alberta
Scopula inductata	UASM59118	GWNS272-07	HQ649273	Canada	Alberta	Strickland Museum of Entomology, University of Alberta
Scopula junctaria	08-JDWBC-0112	LBCG112-08	HQ649304	Canada	British Columbia	Spencer Entomological Museum, UBC
Scopula junctaria	ENT002-001653	GWNR401-07	HQ649303	Canada	British Columbia	Royal British Columbia Museum
Scopula junctaria	ENT002-001660	GWNR403-07	HQ649301	Canada	British Columbia	Royal British Columbia Museum
Scopula junctaria	ENT002-001661	GWNR402-07	HQ649302	Canada	British Columbia	Royal British Columbia Museum
Scopula junctaria	UASM59115	GWNS256-07	HQ649300	Canada	Alberta	Strickland Museum of Entomology, University of Alberta
Scopula junctaria	UASM59143	GWNS259-07	HQ649297	Canada	Alberta	Strickland Museum of Entomology, University of Alberta
Scopula junctaria	UASM7159	GWNS258-07	HQ649298	Canada	Alberta	Strickland Museum of Entomology, University of Alberta
Scopula junctaria	UASM7318	GWNS257-07	HQ649299	Canada	Alberta	Strickland Museum of Entomology, University of Alberta
Scopula junctaria	UASM95805	GWNS260-07	HQ649291	Canada	British Columbia	Strickland Museum of Entomology, University of Alberta
Scopula junctaria	UASM95837	GWNS512-07	HQ649295	Canada	Alberta	Strickland Museum of Entomology, University of Alberta
Scopula junctaria	UASM95848	GWNS513-07	HQ649294	Canada	Alberta	Strickland Museum of Entomology, University of Alberta
Scopula junctaria	UASM95850	GWNS511-07	HQ649296	Canada	Alberta	Strickland Museum of Entomology, University of Alberta
Scopula junctaria	UASM95859	GWNS514-07	HQ649293	Canada	Alberta	Strickland Museum of Entomology, University of Alberta
Scopula junctaria	UASM95860	GWNS516-07	HQ649292	Canada	Alberta	Strickland Museum of Entomology, University of Alberta
Scopula limboundata	CNCLEP00033369	GWNC370-07	HQ649305	Canada	Alberta	Canadian National Collection of Insects, Arachnids and Nematodes
Scopula limboundata	UASM58451	GWNS251-07	HQ649307	Canada	Alberta	Strickland Museum of Entomology, University of Alberta
Scopula limboundata	UASM95803	GWNS252-07	HQ649306	Canada	Alberta	Strickland Museum of Entomology, University of Alberta
Scopula luteolata	08-JDWBC-0009	LBCG009-08	HQ649314	Canada	British Columbia	Spencer Entomological Museum, UBC
Scopula luteolata	08-JDWBC-0109	LBCG109-08	HQ649313	Canada	British Columbia	Spencer Entomological Museum, UBC
Scopula luteolata	08-JDWBC-1306	LBCG1306-09	HQ649319	Canada	British Columbia	Spencer Entomological Museum, UBC
Scopula luteolata	08-JDWBC-1310	LBCG1310-09	HQ649318	Canada	British Columbia	Spencer Entomological Museum, UBC
Scopula luteolata	08-JDWBC-1315	LBCG1315-09	HQ649317	Canada	British Columbia	Spencer Entomological Museum, UBC
Scopula luteolata	08-JDWBC-1316	LBCG1316-09	HQ649316	Canada	British Columbia	Spencer Entomological Museum, UBC
Scopula luteolata	08-JDWBC-1317	LBCG1317-09	HQ649315	Canada	British Columbia	Spencer Entomological Museum, UBC
Scopula luteolata	CNCLEP00033371	GWNC372-07	HQ649308	Canada	British Columbia	Canadian National Collection of Insects, Arachnids and Nematodes
Scopula luteolata	ENT992-020651	GWNR395-07	HQ649311	Canada	British Columbia	Royal British Columbia Museum
Scopula luteolata	ENT996-004257	GWNR381-07	HQ649312	Canada	British Columbia	Royal British Columbia Museum
Scopula luteolata	JECW-07-0128	GWNJ128-07	HQ649309	Canada	British Columbia	James Entomological Collection, Washington State University
Scopula luteolata	UASM95810	GWNS273-07	HQ649310	Canada	British Columbia	Strickland Museum of Entomology, University of Alberta
Scopula quinquelinearia	08-JDWBC-0040	LBCG040-08	HQ649325	Canada	British Columbia	Spencer Entomological Museum, UBC
Scopula quinquelinearia	08-JDWBC-0390	LBCG390-08	HQ649326	Canada	British Columbia	Spencer Entomological Museum, UBC
Scopula quinquelinearia	ENT991-005627	GWNR405-07	HQ649323	Canada	British Columbia	Royal British Columbia Museum
Scopula quinquelinearia	ENT996-004301	GWNR406-07	HQ649322	Canada	British Columbia	Royal British Columbia Museum
Scopula quinquelinearia	ENT996-004318	GWNR404-07	HQ649324	United States	Washington	Royal British Columbia Museum
Scopula quinquelinearia	UASM95807	GWNS262-07	HQ649321	Canada	British Columbia	Strickland Museum of Entomology, University of Alberta
Scopula quinquelinearia	UASM95808	GWNS263-07	HQ649320	Canada	British Columbia	Strickland Museum of Entomology, University of Alberta
Scopula sentinaria	CNCLEP00033282	GWNC283-07	HQ649327	Canada	Yukon Territory	Canadian National Collection of Insects, Arachnids and Nematodes
Scopula sentinaria	CNCLEP00033374	GWNC375-07	HQ649329	Canada	Alberta	Canadian National Collection of Insects, Arachnids and Nematodes
Scopula sentinaria	CNCLEP00033375	GWNC376-07	HQ649328	Canada	Alberta	Canadian National Collection of Insects, Arachnids and Nematodes
Scopula sentinaria	ENT002-001693	GWNR411-07	HQ649335	Canada	British Columbia	Royal British Columbia Museum
Scopula sentinaria	ENT002-002420	GWNR412-07	HQ649334	Canada	British Columbia	Royal British Columbia Museum
Scopula sentinaria	ENT991-164201	GWNR409-07	HQ649337	Canada	Alberta	Royal British Columbia Museum
Scopula sentinaria	ENT996-004342	GWNR410-07	HQ649336	Canada	Yukon Territory	Royal British Columbia Museum
Scopula sentinaria	UASM41060	GWNS277-07	HQ649332	Canada	Alberta	Strickland Museum of Entomology, University of Alberta
Scopula sentinaria	UASM58929	GWNS276-07	HQ649333	Canada	Alberta	Strickland Museum of Entomology, University of Alberta
Scopula sentinaria	UASM59239	GWNS275-07	HQ649331	Canada	Yukon Territory	Strickland Museum of Entomology, University of Alberta
Scopula sentinaria	UASM78376	GWNS017-07	HQ649330	Canada	Alberta	Strickland Museum of Entomology, University of Alberta
Scopula septentrionicola	CNCLEP00033274	GWNC275-07	HQ649338	Canada	Yukon Territory	Canadian National Collection of Insects, Arachnids and Nematodes
Scopula septentrionicola	ENT996-004332	GWNR414-07	HQ649340	Canada	Yukon Territory	Royal British Columbia Museum
Scopula septentrionicola	UASM59203	GWNS266-07	HQ649339	Canada	Yukon Territory	Strickland Museum of Entomology, University of Alberta
Scopula siccata	CNCLEP00033275	GWNC276-07	HQ649341	Canada	Alberta	Canadian National Collection of Insects, Arachnids and Nematodes
Scopula siccata	UASM59837	GWNS279-07	HQ649342	Canada	Alberta	Strickland Museum of Entomology, University of Alberta
Scopula siccata	UASM59838	GWNS278-07	HQ649343	Canada	Alberta	Strickland Museum of Entomology, University of Alberta
Scopula sideraria	ENT996-004340	GWNR416-07	HQ649344	United States	Washington	Royal British Columbia Museum
Scopula sp.	ENT996-004339	GWNR415-07	HQ649346	United States	Washington	Royal British Columbia Museum
Scopula sp.	ENT996-004341	GWNR417-07	HQ649345	United States	Washington	Royal British Columbia Museum
Selenia alciphearia	07-JDWBC-0021	GWND014-07	HQ649350	Canada	British Columbia	Royal British Columbia Museum
Selenia alciphearia	07-JDWBC-0049	GWND041-07	HQ649349	Canada	British Columbia	Royal British Columbia Museum
Selenia alciphearia	08-JDWBC-0317	LBCG317-08	HQ649357	Canada	British Columbia	Spencer Entomological Museum, UBC
Selenia alciphearia	CNCLEP00033186	GWNC187-07	HQ649347	Canada	Yukon Territory	Canadian National Collection of Insects, Arachnids and Nematodes
Selenia alciphearia	Dun-08-365	DUNLP365-08	HQ649348	Canada	British Columbia	Pacific Forestry Centre, Canadian Forest Service
Selenia alciphearia	ENT002-001613	GWNR270-07	HQ649354	Canada	British Columbia	Royal British Columbia Museum
Selenia alciphearia	ENT991-006309	GWNR267-07	HQ649356	Canada	British Columbia	Royal British Columbia Museum
Selenia alciphearia	ENT996-004004	GWNR268-07	HQ649355	Canada	British Columbia	Royal British Columbia Museum
Selenia alciphearia	NFRC-P-2007-100030	GWNN030-07	HQ649353	Canada	Alberta	Northern Forestry Centre, Canadian Forest Service
Selenia alciphearia	UASM24783	GWNS164-07	HQ649351	Canada	Alberta	Strickland Museum of Entomology, University of Alberta
Selenia alciphearia	UASM41835	GWNS163-07	HQ649352	Canada	Alberta	Strickland Museum of Entomology, University of Alberta
Selenia kentaria	CNCLEP00033187	GWNC188-07	HQ649358	Canada	Alberta	Canadian National Collection of Insects, Arachnids and Nematodes
Selenia kentaria	UASM57995	GWNS166-07	HQ649359	Canada	Alberta	Strickland Museum of Entomology, University of Alberta
Selenia kentaria	UASM57996	GWNS165-07	HQ649360	Canada	Alberta	Strickland Museum of Entomology, University of Alberta
Sericosema juturnaria	08-JDWBC-1076	LBCG1076-09	HQ649368	Canada	British Columbia	Spencer Entomological Museum, UBC
Sericosema juturnaria	08-JDWBC-1077	LBCG1077-09	HQ649367	Canada	British Columbia	Spencer Entomological Museum, UBC
Sericosema juturnaria	CNCLEP00033129	GWNC130-07	HQ649362	Canada	British Columbia	Canadian National Collection of Insects, Arachnids and Nematodes
Sericosema juturnaria	CNCLEP00033130	GWNC131-07	HQ649361	Canada	British Columbia	Canadian National Collection of Insects, Arachnids and Nematodes
Sericosema juturnaria	ENT996-003709	GWNR175-07	HQ649369	United States	Washington	Royal British Columbia Museum
Sericosema juturnaria	JECW-07-0051	GWNJ051-07	HQ649363	United States	Washington	James Entomological Collection, Washington State University
Sericosema juturnaria	PFC-2007-0341	GWNP131-07	HQ649364	Canada	British Columbia	Pacific Forestry Centre, Canadian Forest Service
Sericosema juturnaria	SEM-UBC-GEO-0125	GWNU078-07	HQ649366	Canada	British Columbia	Spencer Entomological Museum, UBC
Sericosema juturnaria	WFBM-07-0034	GWNW034-07	HQ649365	United States	Idaho	University of Idaho, W. F. Barr Entomological Collection
Sericosema wilsonensis	CNCLEP00033131	GWNC132-07	HQ649370	United States	Washington	Canadian National Collection of Insects, Arachnids and Nematodes
Sericosema wilsonensis	JECW-07-0052	GWNJ052-07	HQ649371	United States	Idaho	James Entomological Collection, Washington State University
Sericosema wilsonensis	PFC-2007-0342	GWNP132-07	HQ649372	Canada	British Columbia	Pacific Forestry Centre, Canadian Forest Service
Sericosema wilsonensis	WFBM-07-0035	GWNW035-07	HQ649373	United States	Idaho	University of Idaho, W. F. Barr Entomological Collection
Sicya macularia	ENT002-001631	GWNR325-07	HQ649380	Canada	British Columbia	Royal British Columbia Museum
Sicya macularia	ENT996-004103	GWNR322-07	HQ649383	Canada	British Columbia	Royal British Columbia Museum
Sicya macularia	ENT996-004107	GWNR324-07	HQ649381	Canada	British Columbia	Royal British Columbia Museum
Sicya macularia	ENT996-004108	GWNR323-07	HQ649382	Canada	British Columbia	Royal British Columbia Museum
Sicya macularia	ENT996-004112	GWNR321-07	HQ649384	United States	Washington	Royal British Columbia Museum
Sicya macularia	JECW-07-0092	GWNJ092-07	HQ649374	United States	Idaho	James Entomological Collection, Washington State University
Sicya macularia	PFC-2007-0411	GWNP201-07	HQ649376	Canada	British Columbia	Pacific Forestry Centre, Canadian Forest Service
Sicya macularia	PFC-2007-0412	GWNP202-07	HQ649375	Canada	British Columbia	Pacific Forestry Centre, Canadian Forest Service
Sicya macularia	UASM24865	GWNS205-07	HQ649378	Canada	Alberta	Strickland Museum of Entomology, University of Alberta
Sicya macularia	UASM24886	GWNS206-07	HQ649377	Canada	Alberta	Strickland Museum of Entomology, University of Alberta
Sicya macularia	UASM7116	GWNS204-07	HQ649379	Canada	Alberta	Strickland Museum of Entomology, University of Alberta
Spargania luctuata	ENT002-001705	GWNR516-07	HQ649388	Canada	British Columbia	Royal British Columbia Museum
Spargania luctuata	ENT991-004878	GWNR515-07	HQ649389	Canada	British Columbia	Royal British Columbia Museum
Spargania luctuata	ENT991-005586	GWNR514-07	HQ649390	Canada	British Columbia	Royal British Columbia Museum

Spargania luctuata	UASM7164	GWNS352-07	HQ649387	Canada	Alberta	Strickland Museum of Entomology, University of Alberta
Spargania luctuata	UASM7290	GWNS353-07	HQ649386	Canada	Alberta	Strickland Museum of Entomology, University of Alberta
Spargania luctuata	UASM95818	GWNS354-07	HQ649385	Canada	Alberta	Strickland Museum of Entomology, University of Alberta
Spargania magnoliata	08-JDWBC-0003	LBCG003-08	HQ649392	Canada	British Columbia	Spencer Entomological Museum, UBC
Spargania magnoliata	08-JDWBC-1092	LBCG1092-09	HQ649393	Canada	British Columbia	Spencer Entomological Museum, UBC
Spargania magnoliata	08-JDWBC-3277	LBCG3277-09	HQ649394	Canada	British Columbia	Spencer Entomological Museum, UBC
Spargania magnoliata	ENT992-020765	GWNR519-07	HQ649396	Canada	British Columbia	Royal British Columbia Museum
Spargania magnoliata	ENT996-004557	GWNR517-07	HQ649397	Canada	British Columbia	Royal British Columbia Museum
Spargania magnoliata	ENT996-004558	GWNR520-07	HQ649395	United States	Washington	Royal British Columbia Museum
Spargania magnoliata	UASM7334	GWNS351-07	HQ649391	Canada	Alberta	Strickland Museum of Entomology, University of Alberta
Spodolepis danbyi	08-JDWBC-0320	LBCG320-08	HQ649407	Canada	British Columbia	Spencer Entomological Museum, UBC
Spodolepis danbyi	08-JDWBC-0322	LBCG322-08	HQ649406	Canada	British Columbia	Spencer Entomological Museum, UBC
Spodolepis danbyi	08-JDWBC-0323	LBCG323-08	HQ649405	Canada	British Columbia	Spencer Entomological Museum, UBC
Spodolepis danbyi	08-JDWBC-0324	LBCG324-08	HQ649404	Canada	British Columbia	Spencer Entomological Museum, UBC
Spodolepis danbyi	ENT996-003934	GWNR243-07	HQ649408	Canada	British Columbia	Royal British Columbia Museum
Spodolepis danbyi	ENT996-003945	GWNR242-07	HQ649409	Canada	British Columbia	Royal British Columbia Museum
Spodolepis danbyi	JECW-07-0078	GWNJ078-07	HQ649399	United States	Washington	James Entomological Collection, Washington State University
Spodolepis danbyi	JECW-07-0079	GWNJ079-07	HQ649398	United States	Washington	James Entomological Collection, Washington State University
Spodolepis danbyi	UASM24907	GWNS156-07	HQ649403	Canada	Alberta	Strickland Museum of Entomology, University of Alberta
Spodolepis danbyi	UASM24908	GWNS157-07	HQ649402	Canada	Alberta	Strickland Museum of Entomology, University of Alberta
Spodolepis danbyi	UASM41682	GWNS158-07	HQ649401	Canada	Alberta	Strickland Museum of Entomology, University of Alberta
Spodolepis danbyi	UASM59745	GWNS159-07	HQ649400	Canada	British Columbia	Strickland Museum of Entomology, University of Alberta
Stamnoctenis morrisata	08-JDWBC-0199	LBCG199-08	HQ649412	Canada	British Columbia	Spencer Entomological Museum, UBC
Stamnoctenis morrisata	08-JDWBC-1322	LBCG1322-09	HQ649427	Canada	British Columbia	Spencer Entomological Museum, UBC
Stamnoctenis morrisata	08-JDWBC-1323	LBCG1323-09	HQ649426	Canada	British Columbia	Spencer Entomological Museum, UBC
Stamnoctenis morrisata	08-JDWBC-1324	LBCG1324-09	HQ649425	Canada	British Columbia	Spencer Entomological Museum, UBC
Stamnoctenis morrisata	08-JDWBC-1325	LBCG1325-09	HQ649424	Canada	British Columbia	Spencer Entomological Museum, UBC
Stamnoctenis morrisata	08-JDWBC-1326	LBCG1326-09	HQ649423	Canada	British Columbia	Spencer Entomological Museum, UBC
Stamnoctenis morrisata	08-JDWBC-1327	LBCG1327-09	HQ649422	Canada	British Columbia	Spencer Entomological Museum, UBC
Stamnoctenis morrisata	08-JDWBC-1328	LBCG1328-09	HQ649421	Canada	British Columbia	Spencer Entomological Museum, UBC
Stamnoctenis morrisata	08-JDWBC-1329	LBCG1329-09	HQ649420	Canada	British Columbia	Spencer Entomological Museum, UBC
Stamnoctenis morrisata	08-JDWBC-1330	LBCG1330-09	HQ649419	Canada	British Columbia	Spencer Entomological Museum, UBC
Stamnoctenis morrisata	08-JDWBC-1331	LBCG1331-09	HQ649418	Canada	British Columbia	Spencer Entomological Museum, UBC
Stamnoctenis morrisata	08-JDWBC-1332	LBCG1332-09	HQ649417	Canada	British Columbia	Spencer Entomological Museum, UBC
Stamnoctenis morrisata	08-JDWBC-1333	LBCG1333-09	HQ649416	Canada	British Columbia	Spencer Entomological Museum, UBC
Stamnoctenis morrisata	08-JDWBC-1334	LBCG1334-09	HQ649415	Canada	British Columbia	Spencer Entomological Museum, UBC
Stamnoctenis morrisata	08-JDWBC-2118	LBCG2118-09	HQ649428	Canada	British Columbia	Spencer Entomological Museum, UBC
Stamnoctenis morrisata	CNCLEP00033318	GWNC319-07	HQ649411	Canada	British Columbia	Canadian National Collection of Insects, Arachnids and Nematodes
Stamnoctenis morrisata	Dun-08-134	DUNLP134-08	HQ649410	Canada	British Columbia	Pacific Forestry Centre, Canadian Forest Service
Stamnoctenis morrisata	ENT996-007781	GWNR638-07	HQ649414	Canada	British Columbia	Royal British Columbia Museum
Stamnoctenis morrisata	ENT996-007783	GWNR639-07	HQ649413	Canada	British Columbia	Royal British Columbia Museum
Stamnoctenis pearsalli	ENT996-004654	GWNR533-07	HQ649431	Canada	British Columbia	Royal British Columbia Museum
Stamnoctenis pearsalli	JECW-07-0215	GWNJ215-07	HQ649430	United States	Washington	James Entomological Collection, Washington State University
Stamnoctenis pearsalli	JECW-07-0216	GWNJ216-07	HQ649429	United States	Washington	James Entomological Collection, Washington State University
Stamnodes blackmorei	PFC-2007-0294	GWNP087-07	HQ649432	Canada	British Columbia	Pacific Forestry Centre, Canadian Forest Service
Stamnodes marmorata	CNCLEP00033316	GWNC317-07	HQ649434	Canada	British Columbia	Canadian National Collection of Insects, Arachnids and Nematodes
Stamnodes marmorata	CNCLEP00033317	GWNC318-07	HQ649433	Canada	British Columbia	Canadian National Collection of Insects, Arachnids and Nematodes
Stamnodes marmorata	ENT991-012520	GWNR528-07	HQ649437	Canada	British Columbia	Royal British Columbia Museum
Stamnodes marmorata	ENT996-004658	GWNR527-07	HQ649438	United States	Washington	Royal British Columbia Museum
Stamnodes marmorata	JECW-07-0218	GWNJ218-07	HQ649436	United States	Washington	James Entomological Collection, Washington State University
Stamnodes marmorata	JECW-07-0219	GWNJ219-07	HQ649435	United States	Washington	James Entomological Collection, Washington State University
Stamnodes topazata	CGWC-4107	LOWCE347-06	HQ649439	Canada	British Columbia	Biodiversity Institute of Ontario
Stamnodes topazata	CNCLEP00033319	GWNC320-07	HQ649441	Canada	Alberta	Canadian National Collection of Insects, Arachnids and Nematodes
Stamnodes topazata	CNCLEP00033320	GWNC321-07	HQ649440	Canada	Alberta	Canadian National Collection of Insects, Arachnids and Nematodes
Stamnodes topazata	ENT002-001715	GWNR537-07	HQ649443	Canada	British Columbia	Royal British Columbia Museum
Stamnodes topazata	UASM95821	GWNS366-07	HQ649442	Canada	Alberta	Strickland Museum of Entomology, University of Alberta
Stenoporpia excelsaria	08-JDWBC-0016	LBCG016-08	HQ649451	Canada	British Columbia	Spencer Entomological Museum, UBC
Stenoporpia excelsaria	CNCLEP00033092	GWNC093-07	HQ649446	Canada	British Columbia	Canadian National Collection of Insects, Arachnids and Nematodes
Stenoporpia excelsaria	CNCLEP00033362	GWNC363-07	HQ649445	Canada	British Columbia	Canadian National Collection of Insects, Arachnids and Nematodes
Stenoporpia excelsaria	Dun-08-136	DUNLP136-08	HQ649444	Canada	British Columbia	Pacific Forestry Centre, Canadian Forest Service
Stenoporpia excelsaria	ENT996-003489	GWNR113-07	HQ649450	Canada	British Columbia	Royal British Columbia Museum
Stenoporpia excelsaria	ENT996-003497	GWNR114-07	HQ649449	Canada	British Columbia	Royal British Columbia Museum
Stenoporpia excelsaria	ENT996-003499	GWNR115-07	HQ649448	Canada	British Columbia	Royal British Columbia Museum
Stenoporpia excelsaria	JECW-07-0037	GWNJ037-07	HQ649447	Canada	British Columbia	James Entomological Collection, Washington State University
Stenoporpia pulmonaria	CNCLEP00033091	GWNC092-07	HQ649454	Canada	British Columbia	Canadian National Collection of Insects, Arachnids and Nematodes
Stenoporpia pulmonaria	Dun-08-135	DUNLP135-08	HQ649452	Canada	British Columbia	Pacific Forestry Centre, Canadian Forest Service
Stenoporpia pulmonaria	Dun-08-137	DUNLP137-08	HQ649453	Canada	British Columbia	Pacific Forestry Centre, Canadian Forest Service
Stenoporpia pulmonaria	JECW-07-0038	GWNJ038-07	HQ649456	United States	Washington	James Entomological Collection, Washington State University
Stenoporpia pulmonaria	JECW-07-0039	GWNJ039-07	HQ649455	United States	Washington	James Entomological Collection, Washington State University
Stenoporpia pulmonaria	JECW-07-0040	GWNJ040-07	HQ649457	United States	Idaho	James Entomological Collection, Washington State University
Stenoporpia pulmonaria	PFC-2007-0371	GWNP161-07	HQ649460	Canada	British Columbia	Pacific Forestry Centre, Canadian Forest Service
Stenoporpia pulmonaria	PFC-2007-0374	GWNP164-07	HQ649459	Canada	British Columbia	Pacific Forestry Centre, Canadian Forest Service
Stenoporpia pulmonaria	PFC-2007-0375	GWNP165-07	HQ649461	Canada	British Columbia	Pacific Forestry Centre, Canadian Forest Service
Stenoporpia pulmonaria	UASM59366	GWNS071-07	HQ649458	Canada	Alberta	Strickland Museum of Entomology, University of Alberta
Stenoporpia separataria	08-JDWBC-0362	LBCG362-08	HQ649466	Canada	British Columbia	Spencer Entomological Museum, UBC
Stenoporpia separataria	08-JDWBC-0368	LBCG368-08	HQ649465	Canada	British Columbia	Spencer Entomological Museum, UBC
Stenoporpia separataria	BIRD1750	GWNS072-07	HQ649462	Canada	Alberta	Strickland Museum of Entomology, University of Alberta
Stenoporpia separataria	PFC-2007-0370	GWNP160-07	HQ649464	Canada	British Columbia	Pacific Forestry Centre, Canadian Forest Service
Stenoporpia separataria	PFC-2007-0372	GWNP162-07	HQ649463	Canada	British Columbia	Pacific Forestry Centre, Canadian Forest Service
Stergamataea delicatum	WFBM-07-0044	GWNW044-07	HQ649467	United States	Idaho	University of Idaho, W. F. Barr Entomological Collection
Synaxis cervinaria	08-JDWBC-0032	LBCG032-08	HQ649474	Canada	British Columbia	Spencer Entomological Museum, UBC
Synaxis cervinaria	CNCLEP00033222	GWNC223-07	HQ649468	Canada	British Columbia	Canadian National Collection of Insects, Arachnids and Nematodes
Synaxis cervinaria	ENT996-004134	GWNR330-07	HQ649472	Canada	British Columbia	Royal British Columbia Museum
Synaxis cervinaria	ENT996-004139	GWNR327-07	HQ649473	Canada	British Columbia	Royal British Columbia Museum
Synaxis cervinaria	JECW-07-0095	GWNJ095-07	HQ649470	United States	Idaho	James Entomological Collection, Washington State University
Synaxis cervinaria	JECW-07-0096	GWNJ096-07	HQ649469	United States	Washington	James Entomological Collection, Washington State University
Synaxis cervinaria	UASM59738	GWNS213-07	HQ649471	Canada	British Columbia	Strickland Museum of Entomology, University of Alberta
Synaxis jubararia	ENT996-004071	GWNR331-07	HQ649483	Canada	British Columbia	Royal British Columbia Museum
Synaxis jubararia	ENT996-004117	GWNR335-07	HQ649478	Canada	British Columbia	Royal British Columbia Museum
Synaxis jubararia	ENT996-004119	GWNR334-07	HQ649482	Canada	British Columbia	Royal British Columbia Museum
Synaxis jubararia	JECW-07-0100	GWNJ100-07	HQ649475	United States	Washington	James Entomological Collection, Washington State University
Synaxis jubararia	PFC-2007-0415	GWNP205-07	HQ649477	Canada	British Columbia	Pacific Forestry Centre, Canadian Forest Service
Synaxis jubararia	UASM34668	GWNS210-07	HQ649481	Canada	Alberta	Strickland Museum of Entomology, University of Alberta
Synaxis jubararia	UASM58782	GWNS214-07	HQ649479	Canada	Alberta	Strickland Museum of Entomology, University of Alberta
Synaxis jubararia	UASM59739	GWNS212-07	HQ649476	Canada	British Columbia	Strickland Museum of Entomology, University of Alberta
Synaxis jubararia	UASM7117	GWNS211-07	HQ649480	Canada	Alberta	Strickland Museum of Entomology, University of Alberta
Synaxis pallulata	CGWC-0235	LOWC235-05	HQ649486	Canada	British Columbia	Biodiversity Institute of Ontario
Synaxis pallulata	CGWC-0237	LOWC237-05	HQ649485	Canada	British Columbia	Biodiversity Institute of Ontario
Synaxis pallulata	CGWC-0238	LOWC238-05	HQ649484	Canada	British Columbia	Biodiversity Institute of Ontario
Synaxis pallulata	Dun-08-139	DUNLP139-08	HQ649487	Canada	British Columbia	Pacific Forestry Centre, Canadian Forest Service
Synaxis pallulata	PFC-2007-0416	GWNP206-07	HQ649488	Canada	British Columbia	Pacific Forestry Centre, Canadian Forest Service
Synaxis sp.	ENT996-004073	GWNR333-07	HQ649492	Canada	British Columbia	Royal British Columbia Museum
Synaxis sp.	UASM59626	GWNS215-07	HQ649491	Canada	Alberta	Strickland Museum of Entomology, University of Alberta
Synaxis sp.	WFBM-07-0065	GWNW065-07	HQ649490	United States	Idaho	University of Idaho, W. F. Barr Entomological Collection
Synaxis sp.	WFBM-07-0067	GWNW067-07	HQ649489	United States	Idaho	University of Idaho, W. F. Barr Entomological Collection
Synchlora aerata	08-JDWBC-1301	LBCG1301-09	HQ649502	Canada	British Columbia	Spencer Entomological Museum, UBC
Synchlora aerata	CNCLEP00033248	GWNC249-07	HQ649494	Canada	Alberta	Canadian National Collection of Insects, Arachnids and Nematodes

Synchlora aerata	CNCLEP00033249	GWNC250-07	HQ649493	Canada	British Columbia	Canadian National Collection of Insects, Arachnids and Nematodes
Synchlora aerata	ENT002-002424	GWNR364-07	HQ649501	Canada	British Columbia	Royal British Columbia Museum
Synchlora aerata	ENT996-007609	GWNR365-07	HQ649500	Canada	British Columbia	Royal British Columbia Museum
Synchlora aerata	JD1910	GWNS500-07	HQ649496	Canada	British Columbia	Strickland Museum of Entomology, University of Alberta
Synchlora aerata	JECW-07-0123	GWNJ123-07	HQ649495	United States	Washington	James Entomological Collection, Washington State University
Synchlora aerata	UASM24893	GWNS239-07	HQ649497	Canada	Alberta	Strickland Museum of Entomology, University of Alberta
Synchlora aerata	UASM34964	GWNS238-07	HQ649498	Canada	Alberta	Strickland Museum of Entomology, University of Alberta
Synchlora aerata	UASM58440	GWNS237-07	HQ649499	Canada	Alberta	Strickland Museum of Entomology, University of Alberta
Synchlora bistriaria	08-JDWBC-2139	LBCG2139-09	HQ649539	Canada	British Columbia	Spencer Entomological Museum, UBC
Synchlora bistriaria	08-JDWBC-2140	LBCG2140-09	HQ649538	Canada	British Columbia	Spencer Entomological Museum, UBC
Synchlora bistriaria	08-JDWBC-2141	LBCG2141-09	HQ649537	Canada	British Columbia	Spencer Entomological Museum, UBC
Synchlora bistriaria	08-JDWBC-2142	LBCG2142-09	HQ649536	Canada	British Columbia	Spencer Entomological Museum, UBC
Synchlora bistriaria	08-JDWBC-2143	LBCG2143-09	HQ649535	Canada	British Columbia	Spencer Entomological Museum, UBC
Synchlora bistriaria	08-JDWBC-2144	LBCG2144-09	HQ649534	Canada	British Columbia	Spencer Entomological Museum, UBC
Synchlora bistriaria	08-JDWBC-2145	LBCG2145-09	HQ649533	Canada	British Columbia	Spencer Entomological Museum, UBC
Synchlora bistriaria	08-JDWBC-2146	LBCG2146-09	HQ649532	Canada	British Columbia	Spencer Entomological Museum, UBC
Synchlora bistriaria	08-JDWBC-2147	LBCG2147-09	HQ649531	Canada	British Columbia	Spencer Entomological Museum, UBC
Synchlora bistriaria	08-JDWBC-2148	LBCG2148-09	HQ649530	Canada	British Columbia	Spencer Entomological Museum, UBC
Synchlora bistriaria	08-JDWBC-2149	LBCG2149-09	HQ649529	Canada	British Columbia	Spencer Entomological Museum, UBC
Synchlora bistriaria	08-JDWBC-2150	LBCG2150-09	HQ649528	Canada	British Columbia	Spencer Entomological Museum, UBC
Synchlora bistriaria	08-JDWBC-2151	LBCG2151-09	HQ649527	Canada	British Columbia	Spencer Entomological Museum, UBC
Synchlora bistriaria	08-JDWBC-2598	LBCG2598-09	HQ649526	Canada	British Columbia	Spencer Entomological Museum, UBC
Synchlora bistriaria	08-JDWBC-2599	LBCG2599-09	HQ649525	Canada	British Columbia	Spencer Entomological Museum, UBC
Synchlora bistriaria	08-JDWBC-2600	LBCG2600-09	HQ649524	Canada	British Columbia	Spencer Entomological Museum, UBC
Synchlora bistriaria	08-JDWBC-2601	LBCG2601-09	HQ649523	Canada	British Columbia	Spencer Entomological Museum, UBC
Synchlora bistriaria	08-JDWBC-2602	LBCG2602-09	HQ649522	Canada	British Columbia	Spencer Entomological Museum, UBC
Synchlora bistriaria	08-JDWBC-2603	LBCG2603-09	HQ649521	Canada	British Columbia	Spencer Entomological Museum, UBC
Synchlora bistriaria	08-JDWBC-2604	LBCG2604-09	HQ649520	Canada	British Columbia	Spencer Entomological Museum, UBC
Synchlora bistriaria	08-JDWBC-2605	LBCG2605-09	HQ649519	Canada	British Columbia	Spencer Entomological Museum, UBC
Synchlora bistriaria	08-JDWBC-2606	LBCG2606-09	HQ649518	Canada	British Columbia	Spencer Entomological Museum, UBC
Synchlora bistriaria	08-JDWBC-2905	LBCG2905-09	HQ649517	Canada	British Columbia	Spencer Entomological Museum, UBC
Synchlora bistriaria	08-JDWBC-2906	LBCG2906-09	HQ649516	Canada	British Columbia	Spencer Entomological Museum, UBC
Synchlora bistriaria	08-JDWBC-2907	LBCG2907-09	HQ649515	Canada	British Columbia	Spencer Entomological Museum, UBC
Synchlora bistriaria	08-JDWBC-2908	LBCG2908-09	HQ649514	Canada	British Columbia	Spencer Entomological Museum, UBC
Synchlora bistriaria	08-JDWBC-2909	LBCG2909-09	HQ649513	Canada	British Columbia	Spencer Entomological Museum, UBC
Synchlora bistriaria	08-JDWBC-2910	LBCG2910-09	HQ649512	Canada	British Columbia	Spencer Entomological Museum, UBC
Synchlora bistriaria	08-JDWBC-3306	LBCG3306-09	HQ649511	Canada	British Columbia	Spencer Entomological Museum, UBC
Synchlora bistriaria	08-JDWBC-3307	LBCG3307-09	HQ649510	Canada	British Columbia	Spencer Entomological Museum, UBC
Synchlora bistriaria	CNCLEP00033250	GWNC251-07	HQ649503	Canada	Alberta	Canadian National Collection of Insects, Arachnids and Nematodes
Synchlora bistriaria	ENT996-004236	GWNR343-07	HQ649507	United States	Idaho	Royal British Columbia Museum
Synchlora bistriaria	ENT996-004239	GWNR344-07	HQ649506	United States	Idaho	Royal British Columbia Museum
Synchlora bistriaria	JECW-07-0124	GWNJ124-07	HQ649505	United States	Washington	James Entomological Collection, Washington State University
Synchlora bistriaria	JECW-07-0125	GWNJ125-07	HQ649504	United States	Washington	James Entomological Collection, Washington State University
Synchlora bistriaria	UASM24892	GWNS241-07	HQ649508	Canada	Alberta	Strickland Museum of Entomology, University of Alberta
Synchlora bistriaria	UASM41308	GWNS240-07	HQ649509	Canada	Alberta	Strickland Museum of Entomology, University of Alberta
Tetracis cachexiata	UASM10326	GWNS216-07	HQ649542	Canada	Alberta	Strickland Museum of Entomology, University of Alberta
Tetracis cachexiata	UASM41699	GWNS217-07	HQ649541	Canada	Alberta	Strickland Museum of Entomology, University of Alberta
Tetracis cachexiata	UASM57123	GWNS218-07	HQ649540	Canada	Alberta	Strickland Museum of Entomology, University of Alberta
Tetracis crocallata	UASM58367	GWNS219-07	HQ649545	Canada	Alberta	Strickland Museum of Entomology, University of Alberta
Tetracis crocallata	UASM7118	GWNS221-07	HQ649543	Canada	Alberta	Strickland Museum of Entomology, University of Alberta
Tetracis crocallata	UASM7387	GWNS220-07	HQ649544	Canada	Alberta	Strickland Museum of Entomology, University of Alberta
Thallophaga hyperborea	07-JDWBC-0007	GWND007-07	HQ649549	Canada	British Columbia	Royal British Columbia Museum
Thallophaga hyperborea	07-JDWBC-0008	GWND008-07	HQ649548	Canada	British Columbia	Royal British Columbia Museum
Thallophaga hyperborea	07-JDWBC-0011	GWND011-07	HQ649547	Canada	British Columbia	Royal British Columbia Museum
Thallophaga hyperborea	07-JDWBC-0042	GWND034-07	HQ649546	Canada	British Columbia	Royal British Columbia Museum
Thallophaga hyperborea	ENT996-003967	GWNR249-07	HQ649553	United States	Washington	Royal British Columbia Museum
Thallophaga hyperborea	ENT996-003973	GWNR248-07	HQ649554	Canada	British Columbia	Royal British Columbia Museum
Thallophaga hyperborea	PFC-2007-0384	GWNP174-07	HQ649552	Canada	British Columbia	Pacific Forestry Centre, Canadian Forest Service
Thallophaga hyperborea	UASM59627	GWNS161-07	HQ649551	Canada	Alberta	Strickland Museum of Entomology, University of Alberta
Thallophaga hyperborea	UASM59728	GWNS162-07	HQ649550	Canada	British Columbia	Strickland Museum of Entomology, University of Alberta
Thallophaga taylorata	CGWC-0270	LOWC270-05	HQ649559	Canada	British Columbia	Biodiversity Institute of Ontario
Thallophaga taylorata	CGWC-2977	LOWCD157-06	HQ649556	Canada	British Columbia	Biodiversity Institute of Ontario
Thallophaga taylorata	CGWC-4208	LOWCE448-06	HQ649555	Canada	British Columbia	Biodiversity Institute of Ontario
Thallophaga taylorata	CNCLEP00033184	GWNC185-07	HQ649558	Canada	British Columbia	Canadian National Collection of Insects, Arachnids and Nematodes
Thallophaga taylorata	CNCLEP00033185	GWNC186-07	HQ649557	Canada	British Columbia	Canadian National Collection of Insects, Arachnids and Nematodes
Thera juniperata	CNCLEP00021784	GWNC455-07	HQ649562	Canada	Alberta	Canadian National Collection of Insects, Arachnids and Nematodes
Thera juniperata	CNCLEP00021785	GWNC456-07	HQ649560	Canada	British Columbia	Canadian National Collection of Insects, Arachnids and Nematodes
Thera juniperata	CNCLEP00033403	GWNC404-07	HQ649561	Canada	Alberta	Canadian National Collection of Insects, Arachnids and Nematodes
Thera juniperata	UASM41476	GWNS315-07	HQ649565	Canada	Alberta	Strickland Museum of Entomology, University of Alberta
Thera juniperata	UASM41477	GWNS316-07	HQ649564	Canada	Alberta	Strickland Museum of Entomology, University of Alberta
Thera juniperata	UASM58423	GWNS317-07	HQ649563	Canada	Alberta	Strickland Museum of Entomology, University of Alberta
Thera otisi	CNCLEP00033431	GWNC432-07	HQ649566	Canada	Yukon Territory	Canadian National Collection of Insects, Arachnids and Nematodes
Thera otisi	ENT996-007700	GWNR635-07	HQ649575	Canada	British Columbia	Royal British Columbia Museum
Thera otisi	ENT996-007701	GWNR636-07	HQ649574	Canada	British Columbia	Royal British Columbia Museum
Thera otisi	ENT996-007704	GWNR637-07	HQ649573	Canada	British Columbia	Royal British Columbia Museum
Thera otisi	PFC-2007-0295	GWNP088-07	HQ649570	Canada	British Columbia	Pacific Forestry Centre, Canadian Forest Service
Thera otisi	UASM58195	GWNS319-07	HQ649571	Canada	Alberta	Strickland Museum of Entomology, University of Alberta
Thera otisi	UASM58196	GWNS318-07	HQ649572	Canada	Alberta	Strickland Museum of Entomology, University of Alberta
Thera otisi	UASM95844	GWNS494-07	HQ649569	Canada	Alberta	Strickland Museum of Entomology, University of Alberta
Thera otisi	UASM95845	GWNS495-07	HQ649568	Canada	Alberta	Strickland Museum of Entomology, University of Alberta
Thera otisi	UASM95849	GWNS521-07	HQ649567	Canada	Alberta	Strickland Museum of Entomology, University of Alberta
Trichodezia albovittata	ENT987-001244	GWNR582-07	HQ649579	Canada	British Columbia	Royal British Columbia Museum
Trichodezia albovittata	ENT991-068287	GWNR581-07	HQ649580	Canada	British Columbia	Royal British Columbia Museum
Trichodezia albovittata	HLC-20565	LBCA565-05	HQ649576	Canada	British Columbia	Biodiversity Institute of Ontario
Trichodezia albovittata	UASM58553	GWNS404-07	HQ649578	Canada	Alberta	Strickland Museum of Entomology, University of Alberta
Trichodezia albovittata	UASM7023	GWNS405-07	HQ649577	Canada	Alberta	Strickland Museum of Entomology, University of Alberta
Triphosa haesitata	07-JDWBC-0043	GWND035-07	HQ649582	Canada	British Columbia	Royal British Columbia Museum
Triphosa haesitata	07-JDWBC-0048	GWND040-07	HQ649581	Canada	British Columbia	Royal British Columbia Museum
Triphosa haesitata	ENT996-004507	GWNR525-07	HQ649588	United States	Washington	Royal British Columbia Museum
Triphosa haesitata	ENT996-004514	GWNR523-07	HQ649590	Canada	British Columbia	Royal British Columbia Museum
Triphosa haesitata	ENT996-004519	GWNR524-07	HQ649589	Canada	British Columbia	Royal British Columbia Museum
Triphosa haesitata	ENT996-004885	GWNR526-07	HQ649587	Canada	British Columbia	Royal British Columbia Museum
Triphosa haesitata	PFC-2007-0246	GWNP039-07	HQ649585	Canada	British Columbia	Pacific Forestry Centre, Canadian Forest Service
Triphosa haesitata	UASM59731	GWNS321-07	HQ649584	Canada	British Columbia	Strickland Museum of Entomology, University of Alberta
Triphosa haesitata	UASM59732	GWNS322-07	HQ649583	Canada	British Columbia	Strickland Museum of Entomology, University of Alberta
Triphosa haesitata	UASM7162	GWNS320-07	HQ649586	Canada	Alberta	Strickland Museum of Entomology, University of Alberta
Venusia cambrica	ENT996-004860	GWNR585-07	HQ649595	Canada	British Columbia	Royal British Columbia Museum
Venusia cambrica	ENT996-004876	GWNR587-07	HQ649594	Canada	British Columbia	Royal British Columbia Museum
Venusia cambrica	HLC-21719	LBCB779-05	HQ649591	Canada	British Columbia	Biodiversity Institute of Ontario
Venusia cambrica	UASM95832	GWNS400-07	HQ649593	Canada	British Columbia	Strickland Museum of Entomology, University of Alberta
Venusia cambrica	UASM95833	GWNS401-07	HQ649592	Canada	British Columbia	Strickland Museum of Entomology, University of Alberta
Venusia duodecemlineata	PFC-2007-0335	GWNP125-07	HQ649596	Canada	British Columbia	Pacific Forestry Centre, Canadian Forest Service
Venusia obsoleta	07-JDWBC-0002	GWND002-07	HQ649604	Canada	British Columbia	Royal British Columbia Museum
Venusia obsoleta	07-JDWBC-0003	GWND003-07	HQ649603	Canada	British Columbia	Royal British Columbia Museum
Venusia obsoleta	07-JDWBC-0019	GWND012-07	HQ649602	Canada	British Columbia	Royal British Columbia Museum
Venusia obsoleta	07-JDWBC-0035	GWND027-07	HQ649601	Canada	British Columbia	Royal British Columbia Museum
Venusia obsoleta	07-JDWBC-0036	GWND028-07	HQ649600	Canada	British Columbia	Royal British Columbia Museum

Venusia obsoleta	07-JDWBC-0045	GWND037-07	HQ649599	Canada	British Columbia	Royal British Columbia Museum
Venusia obsoleta	CNCLEP00034078	GWNC516-07	HQ649598	Canada	British Columbia	Canadian National Collection of Insects, Arachnids and Nematodes
Venusia obsoleta	CNCLEP00034079	GWNC517-07	HQ649597	Canada	British Columbia	Canadian National Collection of Insects, Arachnids and Nematodes
Venusia obsoleta	ENT996-004921	GWNR590-07	HQ649605	Canada	British Columbia	Royal British Columbia Museum
Venusia pearsalli	08-JDWBC-0010	LBCG010-08	HQ649617	Canada	British Columbia	Spencer Entomological Museum, UBC
Venusia pearsalli	08-JDWBC-0011	LBCG011-08	HQ649616	Canada	British Columbia	Spencer Entomological Museum, UBC
Venusia pearsalli	08-JDWBC-0012	LBCG012-08	HQ649615	Canada	British Columbia	Spencer Entomological Museum, UBC
Venusia pearsalli	08-JDWBC-0277	LBCG277-08	HQ649618	Canada	British Columbia	Spencer Entomological Museum, UBC
Venusia pearsalli	08-JDWBC-0278	LBCG278-08	HQ649619	Canada	British Columbia	Spencer Entomological Museum, UBC
Venusia pearsalli	08-JDWBC-0282	LBCG282-08	HQ649620	Canada	British Columbia	Spencer Entomological Museum, UBC
Venusia pearsalli	08-JDWBC-0283	LBCG283-08	HQ649621	Canada	British Columbia	Spencer Entomological Museum, UBC
Venusia pearsalli	08-JDWBC-0284	LBCG284-08	HQ649622	Canada	British Columbia	Spencer Entomological Museum, UBC
Venusia pearsalli	08-JDWBC-0321	LBCG321-08	HQ649614	Canada	British Columbia	Spencer Entomological Museum, UBC
Venusia pearsalli	08-JDWBC-0328	LBCG328-08	HQ649613	Canada	British Columbia	Spencer Entomological Museum, UBC
Venusia pearsalli	08-JDWBC-0329	LBCG329-08	HQ649612	Canada	British Columbia	Spencer Entomological Museum, UBC
Venusia pearsalli	08-JDWBC-0330	LBCG330-08	HQ649611	Canada	British Columbia	Spencer Entomological Museum, UBC
Venusia pearsalli	CNCLEP00033394	GWNC395-07	HQ649607	Canada	Alberta	Canadian National Collection of Insects, Arachnids and Nematodes
Venusia pearsalli	CNCLEP00033395	GWNC396-07	HQ649606	Canada	British Columbia	Canadian National Collection of Insects, Arachnids and Nematodes
Venusia pearsalli	ENT991-003579	GWNR588-07	HQ649623	Canada	British Columbia	Royal British Columbia Museum
Venusia pearsalli	JD0038	GWNS403-07	HQ649609	Canada	Alberta	Strickland Museum of Entomology, University of Alberta
Venusia pearsalli	NFRC-P-2007-100086	GWNN086-07	HQ649608	Canada	Alberta	Northern Forestry Centre, Canadian Forest Service
Venusia pearsalli	UASM95834	GWNS402-07	HQ649610	Canada	Alberta	Strickland Museum of Entomology, University of Alberta
Xanthorhoe abrasaria	08-JDWBC-0978	LBCG978-09	HQ649630	Canada	British Columbia	Spencer Entomological Museum, UBC
Xanthorhoe abrasaria	08-JDWBC-1112	LBCG1112-09	HQ649629	Canada	British Columbia	Spencer Entomological Museum, UBC
Xanthorhoe abrasaria	08-JDWBC-1809	LBCG1809-09	HQ649634	Canada	British Columbia	Spencer Entomological Museum, UBC
Xanthorhoe abrasaria	08-JDWBC-1812	LBCG1812-09	HQ649633	Canada	British Columbia	Spencer Entomological Museum, UBC
Xanthorhoe abrasaria	CBCC1059	GWNS370-07	HQ649624	Canada	Alberta	Strickland Museum of Entomology, University of Alberta
Xanthorhoe abrasaria	ENT002-001719	GWNR551-07	HQ649631	Canada	British Columbia	Royal British Columbia Museum
Xanthorhoe abrasaria	ENT991-003776	GWNR571-07	HQ649628	Canada	British Columbia	Royal British Columbia Museum
Xanthorhoe abrasaria	ENT991-006399	GWNR575-07	HQ649636	Canada	British Columbia	Royal British Columbia Museum
Xanthorhoe abrasaria	ENT991-006405	GWNR576-07	HQ649635	Canada	British Columbia	Royal British Columbia Museum
Xanthorhoe abrasaria	ENT996-004628	GWNR550-07	HQ649632	United States	Washington	Royal British Columbia Museum
Xanthorhoe abrasaria	UASM24174	GWNS369-07	HQ649625	Canada	Alberta	Strickland Museum of Entomology, University of Alberta
Xanthorhoe abrasaria	UASM95862	GWNS523-07	HQ649626	Canada	Alberta	Strickland Museum of Entomology, University of Alberta
Xanthorhoe abrasaria	UASM95865	GWNS522-07	HQ649627	Canada	Alberta	Strickland Museum of Entomology, University of Alberta
Xanthorhoe algidata	CNCLEP00033414	GWNC415-07	HQ649637	Canada	Alberta	Canadian National Collection of Insects, Arachnids and Nematodes
Xanthorhoe algidata	UASM58362	GWNS313-07	HQ649639	Canada	Alberta	Strickland Museum of Entomology, University of Alberta
Xanthorhoe algidata	UASM95826	GWNS378-07	HQ649638	Canada	Alberta	Strickland Museum of Entomology, University of Alberta
Xanthorhoe alticolata	08-JDWBC-0773	LBCG773-09	HQ649644	Canada	British Columbia	Spencer Entomological Museum, UBC
Xanthorhoe alticolata	CBCC1097_duplicate	GWNS527-07	HQ649642	Canada	Alberta	Strickland Museum of Entomology, University of Alberta
Xanthorhoe alticolata	CNCLEP00034071	GWNC509-07	HQ649640	Canada	Yukon Territory	Canadian National Collection of Insects, Arachnids and Nematodes
Xanthorhoe alticolata	ENT991-003732	GWNR572-07	HQ649643	Canada	British Columbia	Royal British Columbia Museum
Xanthorhoe alticolata	PFC-2007-0318	GWNP108-07	HQ649641	Canada	British Columbia	Pacific Forestry Centre, Canadian Forest Service
Xanthorhoe baffinensis	CNCLEP00033411	GWNC412-07	HQ649646	Canada	Alberta	Canadian National Collection of Insects, Arachnids and Nematodes
Xanthorhoe baffinensis	CNCLEP00034065	GWNC503-07	HQ649645	Canada	Northwest Territories	Canadian National Collection of Insects, Arachnids and Nematodes
Xanthorhoe borealis	CNCLEP00034180	GWNC618-07	HQ649647	United States	Alaska	Canadian National Collection of Insects, Arachnids and Nematodes
Xanthorhoe clarkeata	CNCLEP00034072	GWNC510-07	HQ649649	Canada	British Columbia	Canadian National Collection of Insects, Arachnids and Nematodes
Xanthorhoe clarkeata	CNCLEP00034073	GWNC511-07	HQ649648	Canada	British Columbia	Canadian National Collection of Insects, Arachnids and Nematodes
Xanthorhoe decoloraria	08-JDWBC-1810	LBCG1810-09	HQ649655	Canada	British Columbia	Spencer Entomological Museum, UBC
Xanthorhoe decoloraria	08-JDWBC-1811	LBCG1811-09	HQ649654	Canada	British Columbia	Spencer Entomological Museum, UBC
Xanthorhoe decoloraria	CNCLEP00033415	GWNC416-07	HQ649650	Canada	Alberta	Canadian National Collection of Insects, Arachnids and Nematodes
Xanthorhoe decoloraria	ENT002-001722	GWNR570-07	HQ649653	Canada	British Columbia	Royal British Columbia Museum
Xanthorhoe decoloraria	NFRC-P-2007-100074	GWNN074-07	HQ649651	Canada	Alberta	Northern Forestry Centre, Canadian Forest Service
Xanthorhoe decoloraria	UASM7327	GWNS381-07	HQ649652	Canada	Alberta	Strickland Museum of Entomology, University of Alberta
Xanthorhoe defensaria	08-JDWBC-0006	LBCG006-08	HQ649662	Canada	British Columbia	Spencer Entomological Museum, UBC
Xanthorhoe defensaria	JECW-07-0155	GWNJ155-07	HQ649658	United States	Washington	James Entomological Collection, Washington State University
Xanthorhoe defensaria	JECW-07-0156	GWNJ156-07	HQ649657	United States	Washington	James Entomological Collection, Washington State University
Xanthorhoe defensaria	JECW-07-0157	GWNJ157-07	HQ649656	United States	Idaho	James Entomological Collection, Washington State University
Xanthorhoe defensaria	PFC-2007-0316	GWNP106-07	HQ649661	Canada	British Columbia	Pacific Forestry Centre, Canadian Forest Service
Xanthorhoe defensaria	PFC-2007-0317	GWNP107-07	HQ649660	Canada	British Columbia	Pacific Forestry Centre, Canadian Forest Service
Xanthorhoe defensaria	PFC-2007-0319	GWNP109-07	HQ649659	Canada	British Columbia	Pacific Forestry Centre, Canadian Forest Service
Xanthorhoe ferrugata	CNCLEP00034146	GWNC584-07	HQ649663	Canada	British Columbia	Canadian National Collection of Insects, Arachnids and Nematodes
Xanthorhoe ferrugata	ENT002-001721	GWNR561-07	HQ649668	Canada	British Columbia	Royal British Columbia Museum
Xanthorhoe ferrugata	JD0240	GWNS555-07	HQ649667	Canada	Alberta	Strickland Museum of Entomology, University of Alberta
Xanthorhoe ferrugata	UASM7019	GWNS383-07	HQ649664	Canada	Alberta	Strickland Museum of Entomology, University of Alberta
Xanthorhoe ferrugata	UASM7371	GWNS382-07	HQ649665	Canada	Alberta	Strickland Museum of Entomology, University of Alberta
Xanthorhoe ferrugata	UASM78460	GWNS576-07	HQ649666	Canada	Alberta	Strickland Museum of Entomology, University of Alberta
Xanthorhoe fossaria	CNCLEP00033413	GWNC414-07	HQ649672	Canada	Alberta	Canadian National Collection of Insects, Arachnids and Nematodes
Xanthorhoe fossaria	CNCLEP00034069	GWNC507-07	HQ649671	Canada	Yukon Territory	Canadian National Collection of Insects, Arachnids and Nematodes
Xanthorhoe fossaria	CNCLEP00034070	GWNC508-07	HQ649670	Canada	British Columbia	Canadian National Collection of Insects, Arachnids and Nematodes
Xanthorhoe fossaria	CNCLEP00034156	GWNC594-07	HQ649669	Canada	British Columbia	Canadian National Collection of Insects, Arachnids and Nematodes
Xanthorhoe fossaria	ENT002-002427	GWNR564-07	HQ649678	Canada	British Columbia	Royal British Columbia Museum
Xanthorhoe fossaria	ENT991-162872	GWNR574-07	HQ649675	Canada	British Columbia	Royal British Columbia Museum
Xanthorhoe fossaria	ENT991-163197	GWNR573-07	HQ649676	Canada	British Columbia	Royal British Columbia Museum
Xanthorhoe fossaria	ENT992-020272	GWNR562-07	HQ649679	Canada	British Columbia	Royal British Columbia Museum
Xanthorhoe fossaria	ENT996-004666	GWNR565-07	HQ649677	Canada	Yukon Territory	Royal British Columbia Museum
Xanthorhoe fossaria	NFRC-P-2007-100073	GWNN073-07	HQ649673	Canada	Alberta	Northern Forestry Centre, Canadian Forest Service
Xanthorhoe fossaria	UASM95828	GWNS380-07	HQ649674	Canada	Alberta	Strickland Museum of Entomology, University of Alberta
Xanthorhoe iduata	CNCLEP00034060	GWNC498-07	HQ649680	Canada	British Columbia	Canadian National Collection of Insects, Arachnids and Nematodes
Xanthorhoe iduata	UASM95822	GWNS373-07	HQ649681	Canada	Alberta	Strickland Museum of Entomology, University of Alberta
Xanthorhoe incursata lagganata	UASM99151	MMNA031-08	HQ649684	Canada	Alberta	Strickland Museum of Entomology, University of Alberta
Xanthorhoe incursata lagganata	UASM99152	MMNA032-08	HQ649683	Canada	Alberta	Strickland Museum of Entomology, University of Alberta
Xanthorhoe incursata lagganata	UASM99173	MMNA034-08	HQ649682	Canada	Alberta	Strickland Museum of Entomology, University of Alberta
Xanthorhoe labradorensis	CNCLEP00033410	GWNC411-07	HQ649686	Canada	Alberta	Canadian National Collection of Insects, Arachnids and Nematodes
Xanthorhoe labradorensis	CNCLEP00034056	GWNC494-07	HQ649685	Canada	Yukon Territory	Canadian National Collection of Insects, Arachnids and Nematodes
Xanthorhoe labradorensis	UASM41705	GWNS367-07	HQ649688	Canada	Alberta	Strickland Museum of Entomology, University of Alberta
Xanthorhoe labradorensis	UASM7018	GWNS368-07	HQ649687	Canada	Alberta	Strickland Museum of Entomology, University of Alberta
Xanthorhoe labradorensis	UASM78375	GWNS579-07	HQ649689	Canada	Alberta	Strickland Museum of Entomology, University of Alberta
Xanthorhoe lacustrata	CNCLEP00034145	GWNC583-07	HQ649691	Canada	Alberta	Canadian National Collection of Insects, Arachnids and Nematodes
Xanthorhoe lacustrata	CNCLEP00034147	GWNC585-07	HQ649690	Canada	Alberta	Canadian National Collection of Insects, Arachnids and Nematodes
Xanthorhoe lacustrata	ENT002-001716	GWNR568-07	HQ649694	Canada	British Columbia	Royal British Columbia Museum
Xanthorhoe lacustrata	ENT002-001717	GWNR567-07	HQ649695	Canada	British Columbia	Royal British Columbia Museum
Xanthorhoe lacustrata	ENT002-001718	GWNR566-07	HQ649696	Canada	British Columbia	Royal British Columbia Museum
Xanthorhoe lacustrata	NFRC-P-2007-100077	GWNN077-07	HQ649692	Canada	Alberta	Northern Forestry Centre, Canadian Forest Service
Xanthorhoe lacustrata	UASM7020	GWNS384-07	HQ649693	Canada	Alberta	Strickland Museum of Entomology, University of Alberta
Xanthorhoe macdunnoughi	08-JDWBC-0747	LBCG747-09	HQ649715	Canada	British Columbia	Spencer Entomological Museum, UBC
Xanthorhoe macdunnoughi	08-JDWBC-0748	LBCG748-09	HQ649714	Canada	British Columbia	Spencer Entomological Museum, UBC
Xanthorhoe macdunnoughi	08-JDWBC-0775	LBCG775-09	HQ649713	Canada	British Columbia	Spencer Entomological Museum, UBC
Xanthorhoe macdunnoughi	08-JDWBC-0799	LBCG799-09	HQ649712	Canada	British Columbia	Spencer Entomological Museum, UBC
Xanthorhoe macdunnoughi	08-JDWBC-0977	LBCG977-09	HQ649711	Canada	British Columbia	Spencer Entomological Museum, UBC
Xanthorhoe macdunnoughi	08-JDWBC-1009	LBCG1009-09	HQ649710	Canada	British Columbia	Spencer Entomological Museum, UBC
Xanthorhoe macdunnoughi	08-JDWBC-1013	LBCG1013-09	HQ649709	Canada	British Columbia	Spencer Entomological Museum, UBC
Xanthorhoe macdunnoughi	08-JDWBC-1021	LBCG1021-09	HQ649708	Canada	British Columbia	Spencer Entomological Museum, UBC
Xanthorhoe macdunnoughi	08-JDWBC-1022	LBCG1022-09	HQ649707	Canada	British Columbia	Spencer Entomological Museum, UBC
Xanthorhoe macdunnoughi	08-JDWBC-1023	LBCG1023-09	HQ649706	Canada	British Columbia	Spencer Entomological Museum, UBC
Xanthorhoe macdunnoughi	08-JDWBC-1024	LBCG1024-09	HQ649705	Canada	British Columbia	Spencer Entomological Museum, UBC
Xanthorhoe macdunnoughi	08-JDWBC-1025	LBCG1025-09	HQ649704	Canada	British Columbia	Spencer Entomological Museum, UBC

Xanthorhoe macdunnoughi	08-JDWBC-1026	LBCG1026-09	HQ649703	Canada	British Columbia	Spencer Entomological Museum, UBC
Xanthorhoe macdunnoughi	CNCLEP00034061	GWNC499-07	HQ649698	Canada	Alberta	Canadian National Collection of Insects, Arachnids and Nematodes
Xanthorhoe macdunnoughi	CNCLEP00034062	GWNC500-07	HQ649697	Canada	British Columbia	Canadian National Collection of Insects, Arachnids and Nematodes
Xanthorhoe macdunnoughi	ENT002-001720	GWNR563-07	HQ649702	Canada	British Columbia	Royal British Columbia Museum
Xanthorhoe macdunnoughi	ENT996-004579	GWNR488-07	HQ649701	Canada	British Columbia	Royal British Columbia Museum
Xanthorhoe macdunnoughi	UASM78495	GWNS377-07	HQ649700	Canada	Alberta	Strickland Museum of Entomology, University of Alberta
Xanthorhoe macdunnoughi	UASM95827	GWNS379-07	HQ649699	Canada	Alberta	Strickland Museum of Entomology, University of Alberta
Xanthorhoe packardata	CNCLEP00034152	GWNC590-07	HQ649716	Canada	Alberta	Canadian National Collection of Insects, Arachnids and Nematodes
Xanthorhoe pontiaria	CNCLEP00034068	GWNC506-07	HQ649717	Canada	British Columbia	Canadian National Collection of Insects, Arachnids and Nematodes
Xanthorhoe ramaria	CNCLEP00033412	GWNC413-07	HQ649718	Canada	Alberta	Canadian National Collection of Insects, Arachnids and Nematodes
Xanthorhoe ramaria	JD0844	GWNS556-07	HQ649723	Canada	Alberta	Strickland Museum of Entomology, University of Alberta
Xanthorhoe ramaria	UASM53057	GWNS372-07	HQ649721	Canada	Alberta	Strickland Museum of Entomology, University of Alberta
Xanthorhoe ramaria	UASM59273	GWNS376-07	HQ649722	Canada	Yukon Territory	Strickland Museum of Entomology, University of Alberta
Xanthorhoe ramaria	UASM95823	GWNS374-07	HQ649720	Canada	Alberta	Strickland Museum of Entomology, University of Alberta
Xanthorhoe ramaria	UASM95825	GWNS375-07	HQ649719	Canada	Alberta	Strickland Museum of Entomology, University of Alberta
Xanthorhoe ramaria	UASM95872	GWNS543-07	HQ649725	Canada	Alberta	Strickland Museum of Entomology, University of Alberta
Xanthorhoe ramaria	UASM95873	GWNS544-07	HQ649724	Canada	Alberta	Strickland Museum of Entomology, University of Alberta
Xanthotype sospeta	CNCLEP00034163	GWNC601-07	HQ649726	Canada	Alberta	Canadian National Collection of Insects, Arachnids and Nematodes
Xanthotype sospeta	UASM41955	GWNS131-07	HQ649729	Canada	Alberta	Strickland Museum of Entomology, University of Alberta
Xanthotype sospeta	UASM43228	GWNS133-07	HQ649727	Canada	Alberta	Strickland Museum of Entomology, University of Alberta
Xanthotype sospeta	UASM43230	GWNS132-07	HQ649728	Canada	Alberta	Strickland Museum of Entomology, University of Alberta
Xanthotype urticaria	CNCLEP00033364	GWNC365-07	HQ649730	Canada	Alberta	Canadian National Collection of Insects, Arachnids and Nematodes
Xanthotype urticaria	ENT002-002415	GWNR198-07	HQ649733	Canada	British Columbia	Royal British Columbia Museum
Xanthotype urticaria	ENT002-002416	GWNR199-07	HQ649732	Canada	British Columbia	Royal British Columbia Museum
Xanthotype urticaria	UASM7063	GWNS129-07	HQ649731	Canada	Alberta	Strickland Museum of Entomology, University of Alberta
Zenophleps alpinata	CBCC1251	GWNS553-07	HQ649745	Canada	Alberta	Strickland Museum of Entomology, University of Alberta
Zenophleps alpinata	CNCLEP00033321	GWNC322-07	HQ649737	Canada	Alberta	Canadian National Collection of Insects, Arachnids and Nematodes
Zenophleps alpinata	CNCLEP00033322	GWNC323-07	HQ649736	Canada	Alberta	Canadian National Collection of Insects, Arachnids and Nematodes
Zenophleps alpinata	CNCLEP00033323	GWNC324-07	HQ649735	Canada	Alberta	Canadian National Collection of Insects, Arachnids and Nematodes
Zenophleps alpinata	CNCLEP00033324	GWNC325-07	HQ649734	Canada	Alberta	Canadian National Collection of Insects, Arachnids and Nematodes
Zenophleps alpinata	ENT002-001737	GWNR579-07	HQ649746	Canada	British Columbia	Royal British Columbia Museum
Zenophleps alpinata	JD1790	GWNS504-07	HQ649738	Canada	British Columbia	Strickland Museum of Entomology, University of Alberta
Zenophleps alpinata	JECW-07-0165	GWNJ165-07	HQ649744	United States	Washington	James Entomological Collection, Washington State University
Zenophleps alpinata	JECW-07-0166	GWNJ166-07	HQ649743	United States	Washington	James Entomological Collection, Washington State University
Zenophleps alpinata	UASM41726	GWNS396-07	HQ649739	Canada	Alberta	Strickland Museum of Entomology, University of Alberta
Zenophleps alpinata	UASM57939	GWNS395-07	HQ649740	Canada	Alberta	Strickland Museum of Entomology, University of Alberta
Zenophleps alpinata	UASM58612	GWNS394-07	HQ649741	Canada	Alberta	Strickland Museum of Entomology, University of Alberta
Zenophleps alpinata	UASM58856	GWNS393-07	HQ649742	Canada	Alberta	Strickland Museum of Entomology, University of Alberta
Zenophleps lignicolorata	PFC-2007-0325	GWNP115-07	HQ649747	Canada	British Columbia	Pacific Forestry Centre, Canadian Forest Service
